# The late-evolving salmon and trout join the GnRH1 club

**DOI:** 10.1007/s00418-023-02227-z

**Published:** 2023-08-11

**Authors:** Kristian R. von Schalburg, Brent E. Gowen, Kris A. Christensen, Eric H. Ignatz, Jennifer R. Hall, Matthew L. Rise

**Affiliations:** 1 Department of Biology, Electron Microscopy Laboratory, University of Victoria, Victoria, BC V8W 3N5 Canada; 2 Department of Biology, University of Victoria, Victoria, BC V8W 3N5 Canada; 3 Department of Ocean Sciences, Memorial University of Newfoundland, St. John’s, NL A1C 5S7 Canada; 4 Aquatic Research Cluster, CREAIT Network, Ocean Sciences Centre, Memorial University of Newfoundland, St. John’s, NL A1C 5S7 Canada

**Keywords:** Gamete biology, GnRH, Gene expression, Immunolabeling, Ovary, Testis

## Abstract

Although it is known that the whitefish, an ancient salmonid, expresses three distinct gonadotropin-releasing hormone (GnRH) forms in the brain, it has been thought that the later-evolving salmonids (salmon and trout) had only two types of GnRH: GnRH2 and GnRH3. We now provide evidence for the expression of GnRH1 in the gonads of Atlantic salmon by rapid amplification of cDNA ends, real-time quantitative PCR and immunohistochemistry. We examined six different salmonid genomes and found that each assembly has one gene that likely encodes a viable GnRH1 prepropeptide. In contrast to both functional GnRH2 and GnRH3 paralogs, the GnRH1 homeolog can no longer express the hormone. Furthermore, the viable salmonid GnRH1 mRNA is composed of only three exons, rather than the four exons that build the GnRH2 and GnRH3 mRNAs. Transcribed *gnrh1* is broadly expressed (in 17/18 tissues examined), with relative abundance highest in the ovaries. Expression of the *gnrh2* and *gnrh3* mRNAs is more restricted, primarily to the brain, and not in the gonads. The GnRH1 proximal promoter presents composite binding elements that predict interactions with complexes that contain diverse cell fate and differentiation transcription factors. We provide immunological evidence for GnRH1 peptide in the nucleus of 1-year-old type A spermatogonia and cortical alveoli oocytes. GnRH1 peptide was not detected during other germ cell or reproductive stages. GnRH1 activity in the salmonid gonad may occur only during early stages of development and play a key role in a regulatory network that controls mitotic and/or meiotic processes within the germ cell.

**Supplementary Information:**

The online version contains supplementary material available at 10.1007/s00418-023-02227-z.

## Introduction

Gonadotropin-releasing hormone (GnRH) is best known as the neuropeptide that controls the synthesis and release of gonadotropins from the anterior pituitary gland. Since the early 1970s when mammalian GnRH was first characterized (Amoss et al. 1971; Baba et al. 1971), 19 different vertebrate GnRHs have been discovered. They are divided into three groups based on primary structure and phylogenetic analysis. To date, there are 13 distinct forms of GnRH type 1 (GnRH1), two for GnRH2 and four GnRH3 forms reported (Roch et al. 2014; Gaillard et al. 2018). Mammalian GnRH, chicken-II GnRH and salmon GnRH, respectively, are examples of the older nomenclature for the GnRH1, GnRH2 and GnRH3 types. Differences in the GnRH decapeptides mostly lie in changes to residue sequence in amino acids 5 to 8 (Roch et al. 2014). The conserved N- and C-termini are important for receptor binding and activation (reviewed by Muñoz-Cueto et al. 2020).

Most vertebrates, including mammals, have at least two different forms of GnRH (GnRH1 and GnRH2) in their brains and advanced teleosts usually express all three (Adams et al. 2002; Vickers et al. 2004; Roch et al. 2014; Muñoz-Cueto et al. 2020). During the evolution of the jawed vertebrates, coding for the residues of the active decapeptide portion of the GnRH1 type appears to have been the most amenable to change and has given rise to multiple variant forms (Roch et al. 2014; Gaillard et al. 2018). Most of the differences have occurred in residue positions 7 and 8 in comparison to the originally discovered mammalian GnRH1 QHWSYGLRPG decapeptide. On the other hand, the active hormone residues of the GnRH2 and GnRH3 paralogs have remained very stable in structure (Roch et al. 2014). The GnRH2 hormone is the most conserved peptide sequence (QHWSHGWYPG) remaining identical from cartilaginous fish to humans (Vickers et al. 2004). The GnRH3 form is found only in fishes and the QHWSYGWLPG structure has remained conserved among the salmonids (Roch et al. 2014; Muñoz-Cueto et al. 2020).

GnRH1 has not been detected in the more recently evolved salmonids (salmon or trout) (Vickers et al. 2004). However, the *gnrh2* and *gnrh3* messenger RNAs (mRNAs) (Ashihara et al. 1995; von Schalburg and Sherwood 1999; von Schalburg et al. 1999a; Madigou et al. 2002) and their respective protein expression (von Schalburg et al. 1999b; Gray et al. 2002; Vickers et al. 2004) have been well characterized among the salmonids. The thinking has been that the gene for GnRH1 was no longer viable or had been lost in the salmonids during genome duplication events (Vickers et al. 2004; Muñoz-Cueto et al. 2020).

With the genomes for many salmonids now assembled, we undertook to determine whether a GnRH1 type was present and active in salmon and trout. We found that salmon and trout have five distinct genes that code for GnRH: one for GnRH1, two for GnRH2 and two for GnRH3. The ohnolog for the functional GnRH1 is no longer viable and is disappearing. We discovered four new GnRH1 types among the salmonid family, indicating this peptide is rapidly changing.

We used rapid amplification of complementary DNA (cDNA) ends (RACE) to confirm that transcripts were generated in various tissues and determined the *gnrh1* mRNA framework and 5′- and 3′-termini. We enlarged the study to explore the expression of the *gnrh1* cDNA in comparison to the *gnrh2* and *gnrh3* paralogs in 18 different tissues by real-time quantitative polymerase chain reaction (qPCR). Surprisingly, we found very high *gnrh1* expression levels in the gonads with little or no *gnrh2* or *gnrh3* mRNA detected in these tissues.

The high expression of *gnrh1* in the ovaries led us to examine 1-year-old (immature) and 2.5-year-old (pre-spawn) ovaries and testes for GnRH1 by immunohistochemistry (IHC). We discovered that GnRH1 was expressed in the germ cells of only the immature gonads and not in more mature reproductive stages. We think that this is the first time that the expression of any GnRH has been demonstrated in the nucleus of both the male and female germ cell during immature stages of gonadal development. The promoter of the GnRH1 gene presents recognition elements for members of the PIT-OCT-UNC (POU) and SRY-related box (SOX) families, known regulators of pluripotent and germ cell development transcription programs. We propose GnRH1 may act as a key regulatory factor in an early germ cell-specific steroidogenic network that controls mitotic, self-renewal and/or meiotic processes.

## Materials and methods

### Animals and sampling

For immunohistochemistry, sampling of the 1-year-old male and female Atlantic salmon was conducted as detailed in von Schalburg et al. (2013). The gonads from the older 2.5-year-old salmon were obtained as part of ongoing established aquaculture commercial practices and purposes (Grieg Seafood B.C. Ltd., Canada). The ovaries and testes were removed, dissected into smaller pieces, placed into fixative and stored at 4 °C.

For qPCRs, eight [four male (1135.8 ± 155.6 g; mean ± SE) and four female (1128.3 ± 182.5 g)] adult Atlantic salmon of St. John River origin were euthanized [0.4 g L^−1^ tricaine methanesulfonate (TMS; Syndel Laboratories Ltd., Nanaimo, B.C., Canada)], and 18 different tissues were collected from each fish. A full description of the rearing conditions of these fish prior to sampling and how each tissue was sampled can be found in Crossman et al. (2023). Briefly, blood samples were collected in 300 μL aliquots without heparin into 1.5 mL RNase-free tubes within 2 min of death. Eye, brain, gill, heart, head kidney, posterior kidney, spleen, liver, gonad (i.e., testes or ovaries), stomach, pyloric caecum, midgut, hindgut, skin, muscle and fin were then quickly dissected out with tools that had been treated with RNase AWAY^®^ (Ambion, Thermo Fisher Scientific, Mississauga, ON, Canada), placed into 1.5 mL RNase-free tubes, immediately flash-frozen in liquid nitrogen and then stored at −80 °C until RNA extractions could be performed.

For rainbow trout PCRs, tissues were removed from 16-month-old trout of Pennask Lake origin (Fisheries and Oceans Canada, Pacific Science Enterprise Centre, West Vancouver, BC, Canada). The fish were reared in hatchery conditions on well water (~ 11 °C). The tissues were stored in RNAlater (24 h at 4 °C, then at −20 °C) prior to RNA extraction. Briefly, total RNA was extracted, cDNA synthesized, PCRs performed and amplicons run on agarose gels by standard molecular biology techniques. Purified template was sequenced directly using SeqStudio (Applied Biosystems/Thermo Fisher Scientific).

### Analysis of GnRH genes expressed in six salmonid species

The genome assemblies of grayling (ASM434828v1), whitefish (*Coregonus* sp. “balchen”: AWG_v1), Atlantic salmon (ICSASG_v2), Arctic char (*Salvelinus* spp.: ASM291031v2), rainbow trout (Omyk_1.0) and sockeye salmon (Oner_1.0) were examined. The respective assembly accession designations for each of these genomes are: GCA_004348285.1 (Sävilammi et al. 2019), GCA_902175075.1 (De-Kayne et al. 2020), GCF_000233375.1 (Lien et al. 2016), GCF_002910315.2 (Christensen et al. 2018), GCF_002163495.1 (Pearse et al. 2019) and GCF_006149115.1 (Christensen et al. 2020).

GCF_002910315.2 was originally described for Arctic char, but the species identification was found to be uncertain and therefore updated to *Salvelinus* spp. (Christensen et al. 2021).

Each genome was blasted (https://blast.ncbi.nlm.nih.gov/) (Camacho et al. 2009) with mRNA sequence from each GnRH type in “RefSeq Representative Genomes.” Exon/intron boundaries and the end of each gene were roughly determined by alignments and manual curation. Inspection of promoters for transcription factor recognition motifs was conducted as previously reported (von Schalburg et al. 2013, 2018a).

### Analysis of the GnRH1 homeologs

The NCBI genome viewer was used to identify the homeologous regions surrounding the *gnrh1* gene in Atlantic salmon. Splign (Kapustin et al. 2008) was then used to align the Ssa20 *gnrh1* mRNA (XM_014160183.1) to both Ssa20 and Ssa24 homeologous regions. BLAST was used to align the lake whitefish *gnrh1* mRNA sequence (AY245104.2) to the Atlantic salmon (GCF_000233375.1), *Salvelinus* spp. (GCF_002910315.2), and sockeye salmon (GCF_006149115.1) genome assemblies. BLAST was also used to align the Atlantic salmon homeologous regions surrounding the *gnrh1* gene.

### 5′ and 3′ rapid amplification of cDNA ends (RACE) of *gnrh1* and cloning

The full-length cDNA sequence for Atlantic salmon *gnrh1* was obtained using a commercial kit for 5′ and 3′ rapid amplification of cDNA ends (RACE) [SMARTer^®^ RACE 5′/3′ Kit (Takara Bio, Mountain View, CA, USA)] following the manufacturer's instructions. Briefly, gene-specific primers were designed (Online Resource 1) using Primer3web v.0.4.0 (bioinfo.ut.ee/primer3-0.4.0/) and the predicted mRNA sequence from NCBI GenBank (XM_014160183). A pool of eight brain total RNA samples from the multi-tissue expression qPCR analysis (see below for details) was used as template for the 5′ and 3′ RACE cDNA synthesis reactions. The cDNAs were then diluted by adding 10 µL of Tricine-EDTA buffer prior to their use in the 5′ and 3′ RACE touchdown PCR reactions. The touchdown PCR cycling conditions were: 94 °C for 1 min; 5 cycles of (94 °C for 30 s, 72 °C for 3.5 min); 5 cycles of (94 °C for 30 s, 70 °C for 30 s, 72 °C for 3.5 min); 25 cycles of (94 °C for 30 s, 68 °C for 30 s, 72 °C for 3.5 min) and 1 final extension cycle of 72 °C for 10 min. Five microliters of the primary 5′ and 3′ RACE PCR products were then diluted by adding 245 µL of Tricine-EDTA buffer to generate templates for nested PCR. The nested PCR cycling conditions were: 94 °C for 1 min; 20 cycles of (94 °C for 30 s, 68 °C for 30 s, 72 °C for 3.5 min) and 1 final extension cycle of 72 °C for 10 min.

The nested 5′ and 3′ RACE PCR products were electrophoresed on a 1.0% agarose gel, excised and purified using the NucleoSpin^®^ Gel and PCR Clean-up Kit (Takara Bio). They were then cloned into the linearized pRACE vector using the In-Fusion HD Cloning Kit (Takara Bio), and transformations were performed using One Shot Top10 Chemically Competent *E. coli* cells (Invitrogen/Thermo Fisher Scientific, Burlington, ON, Canada) and standard molecular biology techniques. Plasmid DNA was extracted from individual clones using the QIAprep Spin Miniprep Kit (QIAGEN, Mississauga, ON, Canada). These protocols were all performed following the manufacturer's instructions. Insert sizes were verified by restriction enzyme analysis using *Eco*RI and *Hind*III (Invitrogen/Thermo Fisher Scientific), followed by 1.0% agarose gel electrophoresis with visual comparison to a DNA size marker (1 kb Plus DNA Ladder; Invitrogen/Thermo Fisher Scientific).

Eight clones from each of the 5′ and 3′ RACE PCR products were sequenced at Génome Québec CES (Montréal, QC, Canada). Sanger sequencing was performed in both directions (with M13 primers) using the 3730xl DNA Analyzer (Applied Biosystems/Thermo Fisher Scientific). Vector NTI and AlignX (Vector NTI Advance 11.5; Invitrogen/Thermo Fisher Scientific) were used to analyze and assemble the complete cDNA sequence for *gnrh1* (OQ784171).

### RNA preparation for qPCRs

Total RNA was extracted from the 18 tissues listed above as described in Crossman et al. (2023). In summary, tissue samples were homogenized (TissueLyser II, QIAGEN, Mississauga, ON, Canada) in TRIzol^®^, then frozen on dry ice and stored at −80 °C. Later, the homogenates were thawed on wet ice and centrifuged through QIAshredder (QIAGEN) spin columns following the manufacturer's instructions. Additional TRIzol^®^ was added and total RNA extractions were then completed following the manufacturer's instructions.

Due to either low 260/230 absorbance ratios or high levels of gDNA contamination, RNA subsamples from eight of the tissues (brain, midgut, hindgut, pyloric caecum, eye, gonad, liver and blood) were re-extracted using the phenol–chloroform phase separation method (Xu et al. 2013). All RNA samples were then DNaseI-treated (6.8 Kunitz units added to 30 μg total RNA; RNase-Free DNase Set, QIAGEN) and column-purified using the RNeasy MinElute Kit (QIAGEN) following the manufacturer's instructions. As determined by NanoDrop UV spectrophotometry (Thermo Fisher), all column-purified RNA samples were of high purity (i.e., A260/280 ratios > 2.0 and A260/230 ratios > 1.9). In addition, tight 28S and 18S ribosomal RNA bands (with 28S being approximately twice as intense as 18S) were found using 1.0% agarose gel electrophoresis, confirming RNA quality.

For primer quality testing and for generation of no reverse transcriptase control (NRTC) templates, 1 µg (or 2 µg for ovaries and testes) of each column-purified RNA sample (*n* = 8, or *n* = 4 for ovaries and testes) for a given tissue were combined to generate a tissue specific RNA pool that was then utilized in the cDNA (or mock cDNA) synthesis.

### cDNA synthesis and qPCR parameters

First-strand cDNA templates for qPCR were synthesized in 20 μL reactions from 1 μg of DNaseI-treated, column-purified total RNA using random primers (250 ng), dNTPs (0.5 mM final concentration), M-MLV reverse transcriptase (200 U; Invitrogen/Thermo Fisher Scientific) with the manufacturer's first strand buffer (1× final concentration) and DTT (10 mM final concentration) at 37 °C for 50 min.

To ensure that the RNA samples were free of gDNA contamination, each of the aforementioned 18 RNA tissue pools were subjected to a “mock” cDNA synthesis to generate 18 tissue-specific NRTCs.

PCR amplifications were performed in 13 μL reactions using 1× Power SYBR Green PCR Master Mix (Applied Biosystems/Thermo Fisher Scientific), 50 nM of both the forward and reverse primers, and the indicated cDNA quantity. Amplifications were performed using the QuantStudio 6 Flex Real Time PCR system (Applied Biosystems/Thermo Fisher Scientific). The real-time analysis program consisted of 1 cycle of 50 °C for 2 min, 1 cycle of 95 °C for 10 min and 40 cycles of 95 °C for 15 s and 60 °C for 1 min, with fluorescence detection at the end of each 60 °C step, and was followed by dissociation curve analysis.

### Primer design and quality assurance testing

The sequences of the primer pairs used in qPCR analyses and additional information are presented in Online Resource 1. Primers for the five *gnrh* transcripts were designed using Primer3 (bioinfo.ut.ee/primer3/); whereas the primers for the normalizers had been designed and published previously (Xue et al. 2015; Caballero-Solares et al. 2017). Primer quality testing was performed for the five *gnrh* transcripts using a cDNA template synthesized from the brain RNA pool. Amplification efficiencies (required to be between 90 and 110%; Pfaffl 2001) were calculated by generating standard curves using either a 5- (for *gnrh1*) or 4- (for each *gnrh2* and *gnrh3* paralog) point 1:3 dilution series starting with cDNA representing 10 ng of input total RNA and included a no-template control (NTC).

Testing was also performed to ensure that a single product was amplified (dissociation curve analysis) and that there was no primer-dimer present in the NTC. Amplicons were electrophoretically separated on 2% agarose gels and compared with a 1 kb plus ladder (Invitrogen/Thermo Fisher Scientific) to verify that the correct size fragment was being amplified. Primers for the two normalizers (*rpl32*, *eif3d*) had been previously subjected to quality control testing in four cDNA templates synthesized from the RNA pools for brain, gill, head kidney and muscle (Crossman et al. 2023).

### Endogenous control (normalizer) selection

Expression levels of *gnrh1* and each *gnrh2* and *gnrh3* paralog were normalized to transcript levels of two endogenous controls. These endogenous controls were previously selected from seven candidate normalizers: 60S ribosomal protein L32 (*rpl32*), β-actin (*actb*), elongation factor 1-alpha 1 (*ef1a1*), elongation factor 1-alpha 2 (*ef1a2*), eukaryotic translation initiation factor 3 subunit D (*eif3d*), polyadenylate-binding protein 1 (*pabpc1*) and RNA polymerase 2 (*polr2*). Based on this analysis, *rpl32* and *eif3d* were identified as the most stable normalizers (Crossman et al. 2023).

### Experimental qPCR and data analyses

The experimental qPCR analyses were conducted according to MIQE guidelines (Bustin et al. 2009). cDNA corresponding to 5 ng of input total RNA was used as template in the PCRs and in all cases, technical triplicates were analyzed. As expression levels of the individual transcripts were assessed across multiple plates, a plate linker sample (i.e., a sample that was assessed for each gene on all plates in the study) was also included to ensure there was no plate-to-plate variability. In addition, the aforementioned 18 tissue-specific NRTCs and a NTC were also included. In all cases, there was no amplification in the NRTCs and the NTC for all transcripts tested.

For the *gnrh1* experimental qPCR analysis, for every sample, *gnrh1* and the two endogenous controls were tested on the same plate. The relative quantity (RQ) values were determined using the QuantStudio Real Time PCR Software (version 1.3) (Applied Biosystems/Thermo Fisher Scientific) relative quantification study application, with normalization to both *rpl32* and *eif3d* transcript levels, and with amplification efficiencies incorporated. The sample with the lowest normalized expression (mRNA) level was set as the calibrator sample (i.e., assigned an RQ value = 1.0).

For the *gnrh2* and *gnrh3* experimental qPCR analyses, for every sample, the four *gnrh* transcripts were tested on the same plate (five plates were required to assess all of the samples); however, the two endogenous controls were tested on a different plate than the *gnrh*s. As the QuantStudio Real Time PCR Software (version 1.3) relative quantification study application does not allow calculation of RQ values if, for a given sample, the target gene and endogenous controls are run on different plates, the RQ values were calculated as follows. Within the software, for each individual plate (i.e. “.eds” file), technical replicate outliers were removed. A gene expression study (i.e. “.edm” file) was then created containing the multiple plates for each gene. For each gene, this sets the level at which the fluorescence threshold cycle (*C*_T_) is determined at the same value across all of the plates. The *C*_T_ values for each gene (i.e., the four *gnrh*s and the two normalizers) were then determined for each sample and then exported as an MS-Excel file. These data were then imported into qbase+ (Biogazelle, Ghent, Belgium; Hellemans et al. 2007). Calibrated normalized relative quantities were calculated using normalizer (i.e., *rpl32* and *eif3d*) expression levels, target-specific amplification efficiencies and *C*_T_ values of plate linkers. An RQ value of 1.0 was assigned to the sample with the lowest expression level.

Data were statistically analyzed and graphed using RStudio (R Studio Team, 2015; R version 3.6.3). RQ values were log_10_-transformed to meet normality assumptions before comparisons were made using one-way analysis of variance (ANOVA) followed by Tukey's post hoc test to determine significant (*p* < 0.05) differences between tissues. In addition, *t*-tests were performed to assess differences between sexes within the same tissue type.

### LM processing and embedding

For light microscopy morphology and immunolabeling, ovaries and testes were processed into methyl methacrylate/butyl methacrylate (MBM) as described in von Schalburg et al. (2013). Pieces of each gonad were fixed in freshly prepared 4% formaldehyde at room temperature for 1 h and then stored at 4 °C for at least 24 h. After washing in phosphate-buffered saline (PBS) and dehydrating in a graded ethanol series, the samples were processed into catalyzed MBM over a period of a few days. Gonad pieces were placed into individual embedding capsules, and the MBM UV polymerized within a Pelco UVC2 UV Cryo Chamber (Pelco International, Redding, CA, USA) maintained at 4 °C with dry ice.

### LM immunolabeling

Sectioning and immunolabeling procedures used were as detailed in von Schalburg et al. (2013, 2014). Briefly, 4.0 μm-thick sections were cut using a Sorvall JB-4 microtome with glass knives prepared with a Leica EM KMR2 knife maker (Leica Microsystems, Wetzlar, Germany) and placed on microscope slides.

The MBM plastic was removed using acetone, and the nonspecific sites were blocked with PBS-1% ovalbumin. The sections were incubated for 1 h at room temperature with anti-GnRH GF-6 (1:200), kindly provided by Dr. Nancy Sherwood (University of Victoria, Canada). The RRID for GF-6 is: https://scicrunch.org/resolver/RRID:AB_2818954.

After washing in PBS-1% ovalbumin, the sections were incubated with Alexa Fluor 568 goat anti-rabbit immunoglobulin G (IgG; Invitrogen, Molecular Probes, A11011) diluted 1:100 in PBS-1% ovalbumin for 1 h. After washing in PBS-1% ovalbumin, the sections were incubated with DAPI (Sigma, D9564) diluted in PBS-1% ovalbumin for 10 min. The slides were washed with water, and a cover slip was mounted with Fluoromount-G (Electron Microscopy Sciences).

A Leica DM LB2 compound light microscope with a UV fluorescence EBQ 100 lamp control unit and Leica DFC425 digital camera controlled with Leica Application Suite v3.5.0 software was used to collect images.

### TEM processing and embedding

Pieces of the testes were processed for transmission electron microscopy (TEM) morphology into EMBed 812 (Epon 812 replacement) as described in von Schalburg et al. (2013). After primary fixation in Karnovsky's 0.1 M cacodylate-buffered glutaraldehyde (3%) and formaldehyde (3%) and washing in 0.1 M cacodylate buffer, the pieces were post-fixed in 1% osmium tetroxide in fixation buffer for 1 h. After washing in buffer, the samples were en bloc stained in 5% uranyl acetate in 50% ethanol for 1 h, dehydrated in a graded ethanol series, and infiltrated into Epon using propylene oxide as the transition solvent. Epon blocks containing individual pieces of the samples were polymerized at 60 °C for 48 h.

### TEM immunolabeling of MBM sections

Sectioning and immunolabeling procedures used were as detailed in von Schalburg et al. (2013, 2018b). Ultrathin sections were cut on a Leica UltraCut E ultramicrotome using a Diatome diamond knife (Diatome Ltd., Biel/Bienne, Switzerland) and mounted onto 150 nickel grids containing carbon-coated Formvar™ films.

Sections were blocked using PBS-1% ovalbumin and incubated with anti-GnRH GF-6 for 1 h. After washing with PBS-1% ovalbumin, the grids were incubated with secondary antibody [12 nm colloidal Gold AffiniPure goat anti-rabbit IgG (H + L)] (Jackson ImmunoResearch Laboratories, West Grove, PA, USA) diluted 1:50 with PBS-1% ovalbumin for 1 h. The sections were washed with PBS-1% ovalbumin, deionized water and stained with 5% uranyl acetate in 50% ethanol for 10 min and 5% lead citrate for 1 min.

The sections were viewed at 80 kV in the University of Victoria Biology Department JEOL JEM-1011 TEM. Digital images were captured with a Gatan ES1000W Erlangshen 11 Megapixel CCD camera.

### LM and TEM morphology

MBM-embedded ovaries and testes were cut into 4.0 µm-thick sections and stained with Richardson’s light microscopy stain. Ultrathin Epon-embedded testes sections were cut, placed onto 150 bare mesh copper grids and stained for 10 min in uranyl acetate and 4 min in lead citrate.

### Competition assay immunolabeling

The 3,3-diaminobenzidine (DAB) immunolabeling method was used to test the specificity of the anti-GnRH GF-6 primary for GnRH1 on 4.0 µm-thick MBM-embedded ovary and testes sections. A GnRH1 decapeptide, QHWSYGMNPG, was chemically synthesized (Biomatik Corporation, Kitchener, Ontario, Canada) for a preincubation experiment with GF-6. Aliquots of GF-6 or antibody combined with synthesized peptide were prepared in PBS-bovine serum albumin (BSA) (all final concentrations at 1:1000 for the primary), left in the fridge overnight and warmed to room temperature for 1 h prior to immunolabeling.

The sections were incubated overnight with no GF-6 primary, anti-GnRH GF-6 alone and the GF-6/GnRH1 peptide at 4 °C. Following the incubation period, the sections were washed in PBS-1% BSA, then incubated with biotinylated goat anti-rabbit IgG secondary (H&L) (Abcam ab6720) diluted in 1:500 in PBS-BSA for 1 h. After 3× PBS-BSA washes, the sections were exposed to streptavidin-peroxidase (Abcam ab7403) (1:500) for 30 min, then washed in pure PBS and immersed in DAB solution (DAB tetrahydrochloride, Sigma Chemical) for 7.5 min. The slides were rinsed well in running tap water and counterstained in Harris hematoxylin for 30 s. The slides were rinsed in tap water, 70% ethanol for 1 min, 95% ethanol for 1 min, 100% ethanol twice for 1 min and then xylene twice for 1 min and mounted with Permount (Thermo Fisher Scientific).

A Leica DM LB2 compound light microscope with a UV Fluorescence EBQ 100 Lamp Control Unit and Leica DFC425 digital camera controlled with Leica Application Suite v3.5.0 software was used to collect images.

### Immunolabeling controls

To verify the specificity of GF-6, MBM-embedded sections were incubated with just PBS/ovalbumin (no antibody) and antigen pre-adsorbed antiserum. After testing the GF-6 primary in preliminary immunolabeling runs, 4.0 µm MBM sections from five ovaries and five testes from fish of both age groups were immunolabeled. Both age groups were present as an internal control on the same slides.

## Results

### Confirmation of *gnrh1* mRNA framework

Considering that many annotated, assembled genomes for various salmonid species are now complete, we examined six of them to determine whether a functional GnRH1-encoding gene was present among them. The genomes of grayling, whitefish (*Coregonus* sp. “balchen”), Atlantic salmon, Arctic char (*Salvelinus* spp.), rainbow trout and sockeye salmon were surveyed to determine the framework of the *gnrh1* mRNA. Examination of the exon–intron spanning tracks of RNAseq data for these genomes revealed that the salmonid *gnrh1* gene is composed of three exons. These data were used to initially determine the exon–intron bridges and rough 5′- and 3′-ends of the gene/mRNA. We also found a potential functionally intact open reading frame (ORF) for GnRH1 preprohormone in each species we examined (Online Resource 2).

We present the GnRH1 gene, the mRNA and the preproprotein for each species in Online Resource 2. Similarly, we present both of the viable paralogs (Gene1 and Gene2) that encode the GnRH2 and GnRH3 preproproteins for each species in Online Resource 3 and 4, respectively. The corresponding mRNA/cDNA products of the Atlantic salmon GnRH2 and GnRH3 paralogs, designated Gene1 and Gene2, are presented as *gnrh2a* and *gnrh2b* or *gnrh3a* and *gnrh3b*, respectively (Online Resource 1).

### Determination of 5′- and 3′-ends of each GnRH mRNA

**Fig. 1:**
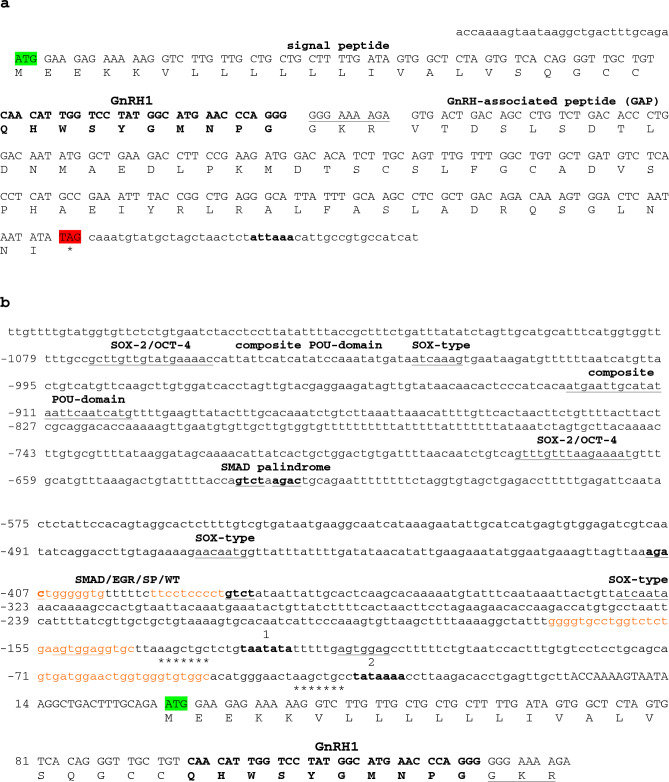
The complete GnRH1 cDNA and proximal promoter of the Atlantic salmon *gnrh1* gene. **a** The full-length GnRH1 cDNA and amino acid sequences. The coding regions for the signal peptide (22 aar), GnRH decapeptide (in bold), amidation/proteolytic cleavage site (underlined) and GnRH-associated peptide (57 aar) are shown. The 5′- and 3′-untranslated regions are shown in lower case. The polyadenylation signal is shown in bold. **b** The proximal promoter and transcription and translation start sites of the *gnrh1* gene. Several different composite regulatory elements are shown. Two composite POU-domain elements could engage multiple members of the diverse POU family of transcription factors (Table 1). An element with motifs that could bind WT-I, EGR-1 and SP-1 (shown in orange) with two flanking SMAD half-sites is presented (−410 to −379). The two potential SMAD half-sites and a SMAD palindromic sequence are underlined and in bold. Two composite elements that could recognize SOX-2 and OCT-4 are underlined. There may be two TATA boxes associated with transcription of *gnrh1*: one that may bind TBP more weakly (1) (−128 to −122) and one with canonical sequences (2) (−29 to −23), respectively. Immediately preceding each TATA box are stretches of DNA that could interact with WT-1, EGR-1 and SP-1 (orange). The motifs AAGCTGC (asterisks below) and AGTGGAG (underlined) in proximity to the TATA boxes may facilitate assembly of the core transcriptional machinery. Translation of preproGnRH1 begins at the start codon (ATG) (green) and the mature hormone processing site (GKR) is underlined. Positions of base pairs (bp) and nucleotides (nt) are based on position relative to the transcription start site

To determine the precise transcription start and termination sites of the Atlantic salmon *gnrh1* mRNA, 5′ and 3′ RACE was performed. The complete *gnrh1* cDNA sequence and the amino acid residues for the preprohormone are shown in Fig. 1. The *gnrh1* mRNA body is composed of only three exons bearing short 5′- and 3′-untranslated regions (utrs). The 5′-utr, the signal peptide, the GnRH decapeptide and the N-terminus of the GnRH-associated peptide (GAP) are encoded on exon 1. Exon 2 encodes the central GAP region and exon 3 encodes the C-terminus of GAP and the 3′-utr (Fig. 1a; Online Resource 2).

The RACE result also provided information necessary to precisely position the 5′ flanking region of the *gnrh1* gene. A canonical TATA box is ~22 bp upstream from the transcription start site in each of the salmonid genomes we examined (Online Resource 2; Fig. 1b). Also, an AUUAAA poly(A) signal is proximal to the translation termination codon for the *gnrh1* mRNAs, with the exception of whitefish.

We determined the 5′- and 3′-utrs of the GnRH2-encoding mRNAs based on RACE results from whitefish (Vickers et al. 2004) and trout (Penlington et al. 1998), respectively (Online Resource 3). The 5′- and 3′-utrs of the GnRH3-encoding mRNAs were determined by primer extension data (Coe et al. 1995; von Schalburg and Sherwood 1999) and RACE results (AB772426.1; Vickers et al. 2004), respectively (Online Resource 4). For consistency, we base the start site for the *gnrh3* mRNAs for each species on our trout data (von Schalburg and Sherwood 1999), but the start sites may begin further upstream as determined in the sockeye (Coe et al. 1995).

### Determination of poly(A) motif in salmonid *gnrh1* mRNAs

Efficient cleavage and polyadenylation of pre-mRNAs requires at least two recognition motifs: usually AAUAAA and a downstream GU-rich element (MacDonald and Redondo 2002). We found a non-AAUAAA motif just 22 nucleotides downstream from the protein termination codon in the Atlantic salmon *gnrh1* mRNA (Fig. 1a). AUUAAA, though “weaker” than AAUAAA, is the second most frequently used poly(A) signal (MacDonald and Redondo 2002). The AUUAAA motif is not present in this location of the whitefish *gnrh1* mRNA, one reason for a much longer 3′-utr in this species. However, this signal is conserved among the other salmonids we examined, indicating that each of the more recently evolved salmonids likely also have very short 3′-utrs in their *gnrh1* mRNAs.

It is unclear whether the grayling mRNA terminates in a position similar to that of the whitefish or of the late-evolving salmonids, but it does present the upstream AUUAAA poly(A) motif unlike the whitefish (Online Resource 2). Further work will be required to unequivocally determine the complete framework of the *gnrh1* mRNA bodies for each of the salmonids examined here.

### Analysis of GnRH gene proximal promoters

Overlapping combinations of different transcription factor (TF) binding motifs are often presented as composite regulatory elements within promoters. We have found a number of interesting examples of composite elements among the different *gnrh* promoters examined here.

We identified a composite element in the *gnrh1* promoter that contains recognition motifs that could interact with two or more different subclasses of POU (PIT-OCT-UNC)-domain TFs (such as PIT-1, BRN-2, BRN-5, OCT-type) (−924 to −900) (Fig. 1b and Table 1) (Rhee et al. 1998; Malik et al. 2018). This element contains non-octameric core binding motifs that could engage BRN-type TFs (ATGAATTGCAT and GCATATAATTCAATCATG) (Rhee et al. 1998). Another element may form an interesting multiprotein complex with TFs of different families, such as SMADs, with other proteins such as Wilms tumor (WT) or the related early growth response (EGR) factors, and with the specificity protein (SP) and Kruppel-like family of TFs (−410 to −379) (Table 1) (Liu et al. 1996; Eisermann et al. 2008; Hill 2016). A SMAD palindromic GTCTnAGAC sequence is positioned ~220 bp upstream from the potential SMAD/EGR/SP/WT composite element. SMAD-3 and SMAD-4 recognize GTCT and AGAC motifs presented in different orientations (Hill 2016; Ramachandran et al. 2018).

**Table 1:**
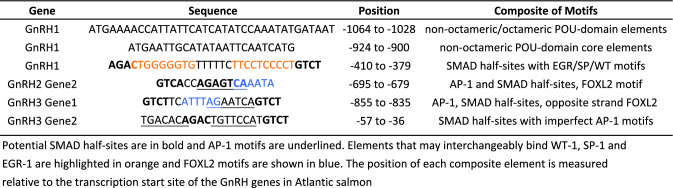
Potential composite regulatory elements in promoters of salmonid GnRH genes

Two elements containing motifs for both SOX-2 and OCT-4 were also identified (Fig. 1b). Although the sequences vary slightly from consensus ((T/C)(A/T)TTGTTnATGCAAAT), both SOX-2 and OCT-4 could bind this element as heterodimers (Kamachi and Kondoh 2013). Extending the OCT-4 co-motif to include the downstream CAT makes each potential octameric motif an imperfect non-octameric palindrome (ATG(A/C)AT(A/T)0-2ATTCAT) (Fig. 1b). This may permit interchange with other POU-factors at these sites since POU TFs adopt different conformations at variable A/T-rich recognition elements through their flexible bipartite binding domains and the “linker” that joins them (Rhee et al. 1998; Malik et al. 2018). In fact, the stretch of DNA between the more upstream SOX-2/OCT-4 element to the single SOX-type motif (ATCAAAG) (from −1064 to −1028) contains several more potential imperfect palindromic non-octameric and octameric POU protein recognition motifs (Rhee et al. 1998; Malik et al. 2018).

We also found a few examples of composite elements in the promoters of the GnRH2 and GnRH3 genes worth mentioning. For the salmonid GnRH2 Gene2, an element that could interact with AP-1, SMAD and/or FoxL2 proteins was detected (Table 1; Online Resource 3). This element is very similar to a regulatory element demonstrated to bind proteins in these families of TFs in the mouse GnRH receptor promoter (Ellsworth et al. 2003). The GnRH3 paralogs also bear elements that potentially could engage with interesting combinations of SMAD and AP-1 proteins (Table 1; Online Resource 4). It is also important to note that the conspicuous G-rich regions that precede the transcription start site of both GnRH2 promoters may interact with proteins such as WT-1, SP-1 and EGR-1, and even with SMADs, as mentioned above for the GnRH1 gene.

We also show that both of the GnRH3 genes and the GnRH2 Gene2 have at least one estrogen response element (ERE) and one cAMP response element (CRE) (TGACNTCA) within 200 bp upstream of the transcription start site for all species we examined (Online Resource 3 and 4). These elements share very similar sequences and positions in each gene. The only exception is that the putative CRE in the GnRH2 Gene2 is further upstream. The GnRH1 and the GnRH2 Gene1 both appear to be devoid of these motifs, at least proximally to the transcription start site. We permitted some variation from ERE consensus in our analysis since imperfect palindromic EREs in the salmon GnRH3 Gene2 have been shown to bind human ER (Klungland et al. 1993).

### Transcription of the GnRH1 homeologs

There are two possible TATA boxes located within the proximal promoter of the salmonid *gnrh1* gene (Fig. 1b). Recognition elements that flank TATA boxes are important for facilitating binding of the TATA-binding protein (TBP) and other associated factors that compose the TFIID complex (Goodrich and Tjian 2010), as well as with components of other TFs that form the core transcriptional machinery. We note that the sequence AAGCTGC, that is present immediately 5′ to each of the TATA boxes (Fig. 1b), is shared among all of the late-evolving salmonid *gnrh1* genes presented here (Online Resource 2). The “weaker” upstream TATA box is flanked by an identical AGTGGAG element (Fig. 1b). The *gnrh1* promoters also possess islands of G- or GC-rich sequences that precede each TATA box. It is not known whether these features contribute to the recruitment and loading of the various TFs that form the core transcription apparatus for processing of the *gnrh1* pre-mRNA.

For each of the *gnrh1* pseudogenes, the TATA boxes and their flanking regions are not conserved positionally, are diminished and/or lost, very likely precluding their ability to transcribe *gnrh1* (Online Resource 2).

### Translation of the GnRH1 homeologs

**Fig. 2:**
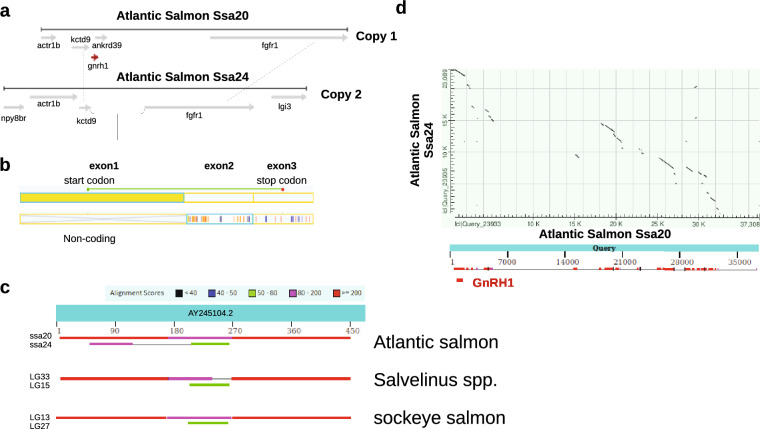
*gnrh1* comparative analysis. **a** Atlantic salmon homeologous regions surrounding *gnrh1* on chromosomes Ssa20 and Ssa24. Only gene symbols are shown. Synteny between the regions is shown with dotted lines. The region on Ssa24 did not have an annotated *gnrh1* gene. **b** Splign alignment of the *gnrh1* mRNA sequence to Ssa20 (top) and Ssa24 (bottom). Based on the Splign alignment, the first exon is non-coding and there has been significant accumulation of mutations in the other two exons for the *gnrh1* copy on Ssa24. **c** Comparison of homeologous copies of *gnrh1* in Atlantic salmon, a *Salvelinus* spp., and sockeye salmon. Each genomic sequence was aligned to the whitefish mRNA (AY245104.2). **d** A dotplot of alignment of homeologous regions Ssa20 and Ssa24 surrounding *gnrh1* (genomic position indicated in **a**). The position of *gnrh1* is shown at the bottom. *npy8br* neuropeptide Y receptor-Y8b, *actr1b* beta-centractin-like, *kctd9* BTB/POZ domain-containing protein KCTD9-like (potassium channel tetramerization domain containing 9a), *gnrh1* gonadotropin releasing hormone 1, *ankrd39* ankyrin repeat domain 39, *fgfr1* fibroblast growth factor receptor 1-A-like, *lgi3* leucine-rich repeat LGI family member 3-like

The salmonid *gnrh2* and *gnrh3* duplicates (Ashihara et al.^1995^; von Schalburg et al. 1999a; von Schalburg and Sherwood 1999; this paper) each encode functional GnRH2 and GnRH3 preprohormones that present identical decapeptides (Online Resource 3 and 4), respectively. However, our analyses indicate that only one active *gnrh1* gene remains in the genomes of salmon and trout species. For example, we show a pseudogene of the homeolog of *gnrh1* remains in the Atlantic salmon genome (Fig. 2a–d). If transcribed, the putative mRNA of this gene would not have a start codon and has accumulated many mutations that would disrupt its function (Fig. 2b).

We also provide evidence that the homeologous *gnrh1* genes for the other species we examined no longer code for a functional GnRH1 preprohormone (Online Resource 2). This is shown in a comparison of the whitefish mRNA to various *gnrh1* homeologs in different salmonids, which reveals that only small regions of moderate identity remain among the *gnrh1* homeologs (Fig. 2c). For Atlantic salmon Ssa20 and Ssa24, the sequence identity was 95% and 76%, with query coverage of 94% and 78%, respectively. A comparison of the surrounding homeologous regions of *gnrh1* shows how little remains of the gene in the corresponding Ssa24 ohnolog (Fig. 2d).

### Disintegration of the GnRH1 homeologs

Each of the salmonid GnRH1 pseudogenes we examined are degenerating in different ways. The grayling and whitefish (*Coregonus* sp. “balchen”) nonviable *gnrh1* homeologs seem to have been disrupted by insertions into their promoter/exon 1 and intron 1 regions, respectively (Online Resource 2). In these two basal salmonids, exon 1 can no longer encode a functional GnRH, but exons 2 and 3 remain relatively intact (i.e., they still theoretically preserve the potential to translate an almost complete GAP). However, this GAP would not be in-frame with GnRH nor would it resemble the GAP of the functional GnRH1 as many indels have occurred within the coding regions of the two exons (Online Resource 2). In the later-evolving salmon and trout, the capacity of exons 2 and 3 to encode GAP is extinguished. None of the salmonid nonviable *gnrh1* ohnologs can synthesize a functional GnRH, with whole in-frame signal peptide and GAP regions (Online Resource 2).

Interestingly, during our examination of the *gnrh1* pseudogenes, we discovered that the whitefish nonviable *gnrh1* gene presented two duplicated copies of the sequence that once encoded the GnRH1 decapeptide. For the more upstream segment, the codons that may have once encoded the signal peptide are intact followed by an in-frame sequence that could encode QHWYHDMSPSTLV (Online Resource 5).

Nevertheless, all known vertebrate GnRH decapeptides have a glycine in position 10 and the residues TLV cannot function as a processing tripeptide (Online Resource 5). Amidation of the mature hormone and splicing of the decapeptide at almost invariable GKR tripeptide sites is an absolute requirement for vertebrate GnRH activity (Roch et al. 2014).

Interestingly, another segment of sequence that could encode a second potential GnRH is immediately downstream of the aforementioned *gnrh1* sequence. Indeed, this appears to be the *bona fide* remnant of exon 1 that remains intact in the viable whitefish *gnrh1* ohnolog. However, translation of the preproprotein cannot occur due to upstream in-frame stop codons (Online Resource 5). Also, a section of sequence is conserved from the GnRH decapeptide coding region to almost the end of exon 1, but the intron 1 sequences past this point are not conserved. In fact, the intron 1 sequences are found approximately 1800 bp further downstream, completely disjointed from exon 1, suggesting a large insertion has occurred between exon 1 and exon 2 in the nonviable gene. Exons 2 and 3 (and intron 2) appear to remain relatively undisturbed (Online Resource 2).

### Comparison of all predicted *gnrh1* mRNAs

Following determination of the full-length Atlantic salmon *gnrh1*, we wondered how this mRNA compared to other salmonid *gnrh1* mRNAs. With the exception of the whitefish (Vickers et al. 2004) and now the Atlantic salmon *gnrh1*, the other salmonid *gnrh1* mRNAs have been automatically generated by the NCBI annotation pipeline (Table 2). This means the generated mRNA transcription start sites and their terminal sequence (for polyadenylation) have not yet been precisely determined. The other salmonid *gnrh1* mRNAs are comparable in structure with the RACE-verified Atlantic salmon mRNA presented here, but have some differences worth noting.

**Table 2:** Computationally derived *gnrh1* mRNAs from NCBI annotation pipeline

mRNA ID	Species name	Common name	GnRH1 peptide	mRNA differences
XM_029677993.1	*Oncorhynchus nerka*	Sockeye	QHW**F**YGLNPG	Very short 3′-utr; unfinished mRNA ends
XM_036935831.1	*Oncorhynchus mykiss*	Trout	QHWSYGLNPG	Stop codon is absent; unfinished ends
XM_024440685.2	*Oncorhynchus tshawytscha*	Chinook	QHWSYGLNPG	Only ORF determined; incomplete mRNA ends
XM_020488559.2	*Oncorhynchus kisutch*	Coho	QHWSYGLNPG	Extra nt in 5′- and 3′-ends of exon 2; incomplete mRNA ends
XM_023978881.1	*Salvelinus* spp.	Char	QHWSY**V**LNPG	Sequence missing in exon 2; unfinished mRNA ends
XM_014160183.1	*Salmo salar*	Atlantic salmon	QHWSYGMNPG	Unfinished mRNA ends
XM_029762814.1	*Salmo trutta*	River trout	QHWSYGMNPG	Unfinished mRNA ends
AY245104.2	*Coregonus clupeaformis*	Whitefish	QHWSYGMNPG	Updated version of 5′-utr accommodates position of upstream TATA box

mRNA differences are based on BLAST comparisons to Atlantic salmon mRNA RACE result. The two amino acid residues in bold highlight change in sockeye and *Salvelinus* spp. GnRH1 from QHWSYGLNPG. The whitefish mRNA information is included to show that the QHWSYGMNPG form is shared with Atlantic salmon and river trout. See text for more details

For example, the rainbow trout mRNA is missing a GACA sequence (as part of a GACAGACA duplicate) in the third exon (nt 17–20 from the beginning of the third exon) that is present in all the salmonids examined here (Online Resource 2). The lack of this sequence presents an ORF that cannot terminate before the RACE-determined mRNA terminus we assume the late-evolving salmonids all share with Atlantic salmon. This predicts an ORF that would run through the AUUAAA poly(A) signal and past the end of the trout mRNA (XM_036935831.1) (Online Resource 2).

To test this we amplified a region of rainbow trout cDNA between the sequence that encodes the signal peptide and a region just downstream of the AUUAAA poly(A) motif by PCR using 5′-GCCTCTAGTGTCACAGGGTT-3′ and 5′-ATGATGGCACGGCAATGTTT-3′ together. Sequencing of the amplicon confirmed that a stop codon was not present in the body of the trout *gnrh1* mRNA. Two other reverse primers targeting more downstream regions of the cDNA were used unsuccessfully in combination with the same forward primer in independent PCRs (5′-AGCCATACCCTGTAATATGCGA-3′ or 5′-GGCCTACCCGTCATTGTATT-3′). Since no amplicons were detected, we assume that the mRNA likely terminates in a position very similar to that shown for the Atlantic salmon mRNA.

These results indicate that the trout mRNA does not code for a preproGnRH1 stop codon. We have previously demonstrated the expression of a correctly spliced GnRH1 decapeptide in precociously mature trout gonads by high-performance liquid chromatography (HPLC)/radioimmunoassay (RIA) (von Schalburg et al. 1999b). The precise mechanism of translation and processing of the trout GnRH1 will need to be investigated further.

A comparison of the *gnrh1* mRNAs predicted for the other salmonids indicates that exon 2 is susceptible to change in some species (Online Resource 6; Table 2). The *Salvelinus* spp. and coho *gnrh1* mRNAs have lost or gained sequence in exon 2, respectively (Online Resource 6). This is interesting considering the mRNA bodies for the paralogs that encode GnRH2 and GnRH3 appear quite stable among the salmonids we examined (Online Resource 3 and 4). In an examination of the *gnrh1* mRNA BLAST results with the coho gene we found that the exon 1 GT donor and exon 2 AG acceptor splice sites were different in comparison to the other salmonids. A more 3′-GT and 5′-AG are selected to bridge the first and second exons, respectively, resulting in the incorporation of an additional 20 nt from the 3′-end of intron 1 into exon 2 (Online Resource 6). For the *Salvelinus* spp. *gnrh1*, the exon 2 GT/AG borders were consistent with the other salmonids, indicating the loss of sequence in exon 2 may be the result of a past deletion event. If these changes in exon 2 are verified, these sequence changes lead to variations in the GAP region of the preprohormone. Nevertheless, each mRNA is predicted to encode a functional preprohormone, both terminating within the first five codons of exon 3 (Online Resource 6).

### Expression levels of GnRH mRNAs across multiple tissues

**Fig. 3:**
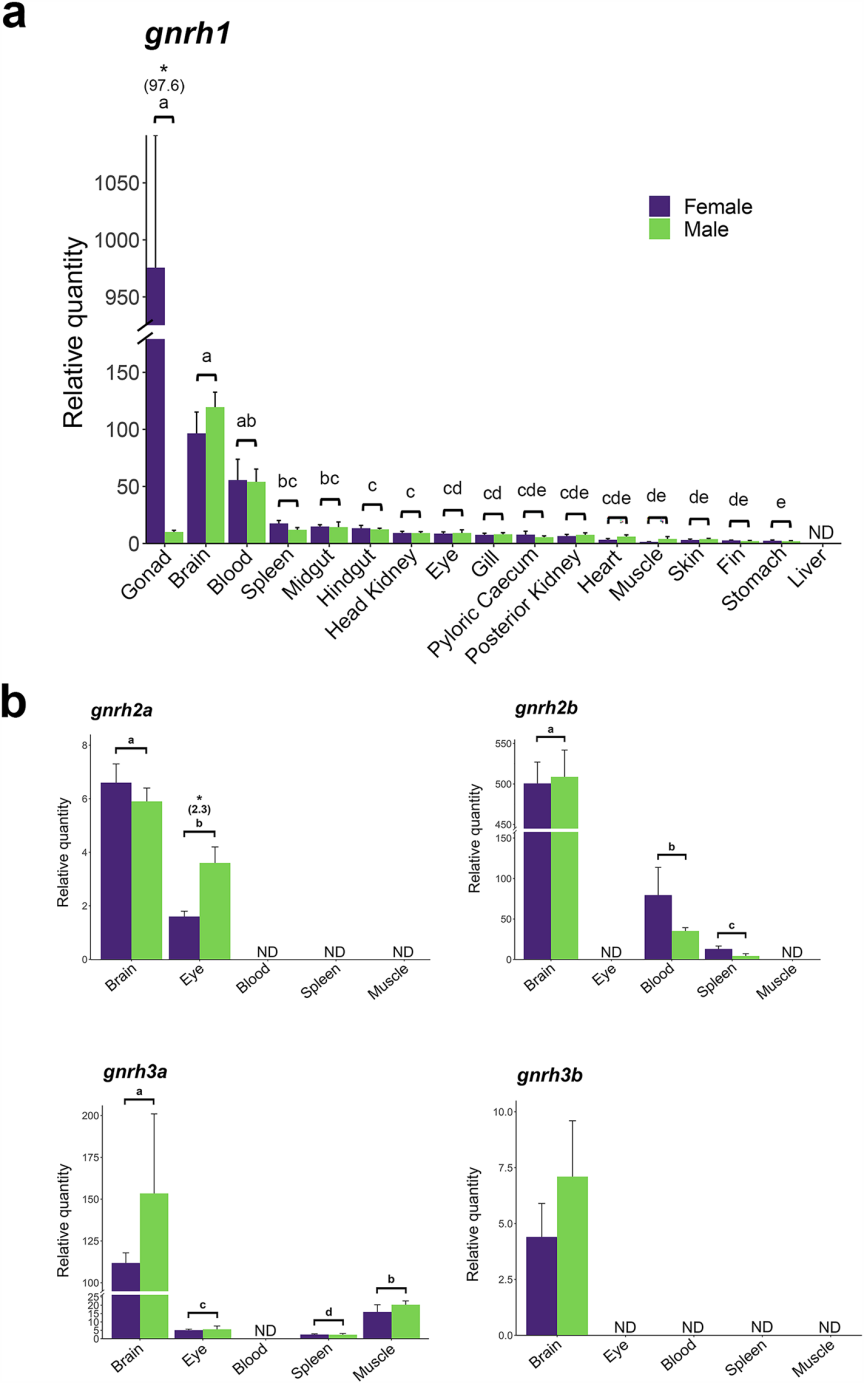
Constitutive expression of five different *gnrh*s in various tissues of adult Atlantic salmon. **a** Constitutive expression of *gnrh1*. Relative quantities are presented as mean ± standard error (*n* = 4 per sex) and are placed in descending order. Different letters denote significant (*p* < 0.05) differences between tissues (1-way ANOVA; data pooled between sexes). The asterisk represents significant (*p* < 0.01) differences between sexes for a given tissue (*t*-test). The number in parentheses below the asterisk is the mean fold-change between sexes. Expression of *gnrh1* was not detected in liver tissue and in two of the muscle samples (one male and one female). **b** Constitutive expression of *gnrh2a*, *gnrh2b*, *gnrh3a* and *gnrh3b.* Relative quantities are presented as mean ± standard error (*n* = 4 per sex). Different letters denote significant (*p* < 0.05) differences between tissues (*gnrh2a*, *t*-test; *gnrh2b* and *gnrh3a*, 1-way ANOVA; data pooled between sexes). The asterisk represents significant (*p* < 0.05) differences between sexes for a given tissue (*t*-test). The number in parentheses below the asterisk is the mean fold-change between sexes. Expression was not detected in gill, heart, head kidney, posterior kidney, liver, gonad, stomach, pyloric caecum, midgut, hindgut, skin or fin tissues for all four transcripts. *ND* not detected

Total RNA/cDNA from 18 different tissues were examined by qPCR to measure the constitutive expression levels of each *gnrh* mRNA. We found that *gnrh1* has the widest distribution among the various tissues examined (Fig. 3a). Expression of the *gnrh2* and *gnrh3* paralogs appear more confined to the brain, eye, blood and spleen (and muscle for *gnrh3a* only) (Fig. 3b).

Levels of *gnrh1* were highest in the ovary, while expression was highest in the brain for the other *gnrh* transcripts. Each *gnrh* type was transcribed in cells of the eye (i.e., *gnrh1*, *gnrh2a*, *gnrh3a*). Significant sex-specific differences were found in expression of *gnrh1* in the gonad (97.6-fold higher in females than males) and in *gnrh2a* in the eye (2.3-fold higher in males than females). Also of interest was the discovery of mRNAs in hematopoietic tissues (i.e., *gnrh1*, *gnrh2b* in blood; *gnrh1*, *gnrh2b*, *gnrh3a* in spleen), pointing to the involvement of this hormone in immune function. Little research has been done in this area for fish, but expression of the GnRH1 peptide has been confirmed in lymphocytes of the red drum (Mohamed and Khan 2006).

We found that only *gnrh1* is expressed in the gonads and at particularly high levels in the ovary, with a mean fold-change 10 times higher than in the brain (Fig. 3a). None of the *gnrh2* or *gnrh3* mRNAs were expressed in the gonads (Fig. 3b). The results for *gnrh3* are supported by earlier transcript analysis done on sockeye salmon (von Schalburg et al. 1999a) and rainbow trout (von Schalburg and Sherwood 1999) that showed *gnrh3* cDNA was not expressed in 2-year-old normal gonads. Similar research on rainbow trout gonads have nevertheless shown the expression of both *gnrh2* (Madigou et al. 2002) or *gnrh3* (Gray et al. 2002; Madigou et al. 2002) transcripts throughout most reproductive stages. However, it has been demonstrated that even during those periods when *gnrh2* and *gnrh3* mRNAs were transcribed, the corresponding peptides were never detected by GF-6 in HPLC/RIAs (von Schalburg et al. 1999b; Gray et al. 2002). In fact, review of these previous studies provide strong evidence that no GnRH peptides, including GnRH1, are synthesized in the gonads of normal, reproductively mature trout during their second and third years of life (von Schalburg et al. 1999b; Gray et al. 2002).

### Specificity of antibody

Antibody GF-6 detects a number of different GnRH forms with different relative cross-reactivities: GnRH1 (e.g., seabream GnRH: 94%; mammalian GnRH: 100%), GnRH2 (10.5%) and GnRH3 (24%) (Quanbeck et al. 1997). GF-6 has been used for HPLC and RIAs to detect immunoreactive GnRHs in salmonids. Various studies primarily examining the brains of salmon or trout have not detected GnRH1. This has led to the long-standing determination that only two GnRHs, GnRH2 and GnRH3, are present in salmon and trout (Vickers et al. 2004 and refs within). However, there has been preliminary evidence for some time that salmon or trout species may possess a third GnRH (Sherwood et al. 1983; von Schalburg et al. 1999b), with a primary structure similar to the whitefish GnRH1 (Adams et al. 2002). We tested the binding specificity of GF-6 for GnRH1 peptide in a preincubation assay, resulting in greatly reduced immunolabeling of 1-year-old Atlantic salmon ovarian and testicular sections (Online Resource 7).

### Ovarian morphology and LM imaging of MBM-embedded sections

**Fig. 4:**
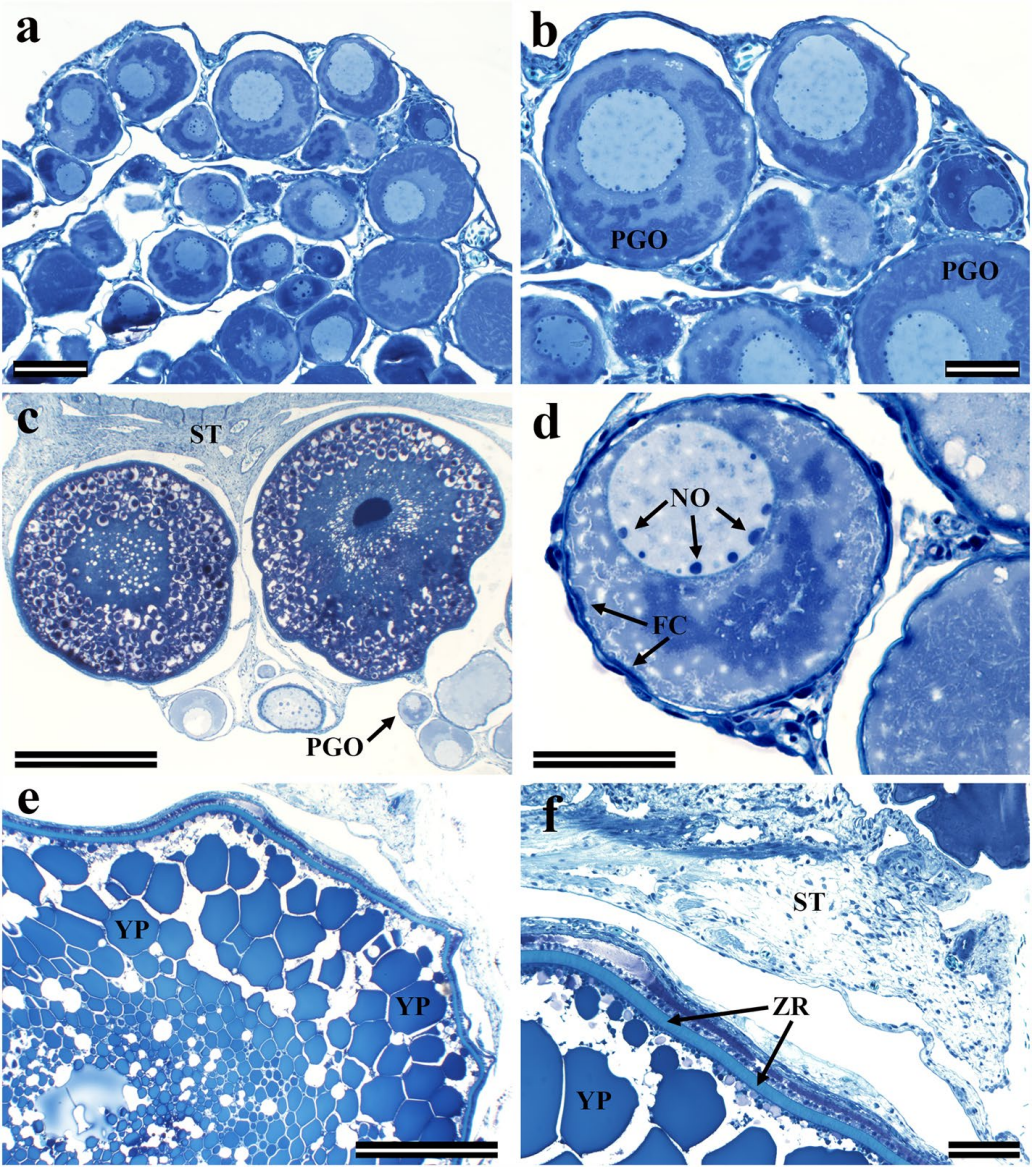
LM images of Richardson-stained MBM-embedded sections showing the general morphology of the ovaries. **a** Low magnification section of a 1-year-old ovary containing oocytes during primary growth stages. Scale bar 100 μm. **b** A higher magnification view of **a** showing more clearly the morphology of the nucleoli near the membranes of the PGO nuclei. Scale bar 50 μm. **c** A section of a 2.5-year-old ovary showing two large oocytes together with a few PGOs. PGO denotes an oocyte examined more closely in image **d**. Scale bar 500 μm. **d** A higher magnification view of the PGO from **c** shows the perinuclear nucleoli and follicle cells much more clearly. Scale bar 50 μm. **e** A section of a large, mature oocyte in comparison to the maturing and smaller oocytes imaged for **c**. In this section, the yolk proteins have filled the ooplasm. Scale bar 500 μm. **f** A magnification of **e** showing the zona radiata surrounding the mature oocyte that is overlaid with follicle cells. Scale bar 100 μm. *PGO* primary growth oocyte, *ST* stroma, *NO* perinuclear nucleoli, *FC* follicle cells, *YP* yolk protein granules, *ZR* zona radiata proteins

The general morphology of the ovaries we examined at the 1-year and 2.5-year-old stages is shown in Fig. 4. We show a low magnification section of a 1-year-old ovary with oocytes during primary growth (Fig. 4a). Most of these Stage I oocytes are in the early cortical alveoli stage as described by Lubzens et al. (2017). A higher magnification view of Fig. 4a clearly shows the morphology of a primary growth oocyte (PGO) with perinuclear nucleoli associated with the nuclei membranes (Fig. 4b). Regions in sections of the 2.5-year-old ovaries were found that contain both large maturing oocytes and oocytes in primary growth stages (Fig. 4c, d). A percentage of Atlantic salmon are iteroparous and it is therefore possible that some of these PGOs would have been viable by the next spawning period. During oocyte maturation, yolk proteins accumulate within the ooplasm (Fig. 4e) and zona radiata proteins form the inner layer of the envelope surrounding the oocyte (Fig. 4f) (Arukwe and Goksøyr 2003). In maturing and growing oocytes, the zona radiata is overlaid with granulosa and theca cells (Arukwe and Goksøyr 2003; Lubzens et al. 2017).

### Testicular morphology and LM imaging of MBM-embedded sections

**Fig. 5:**
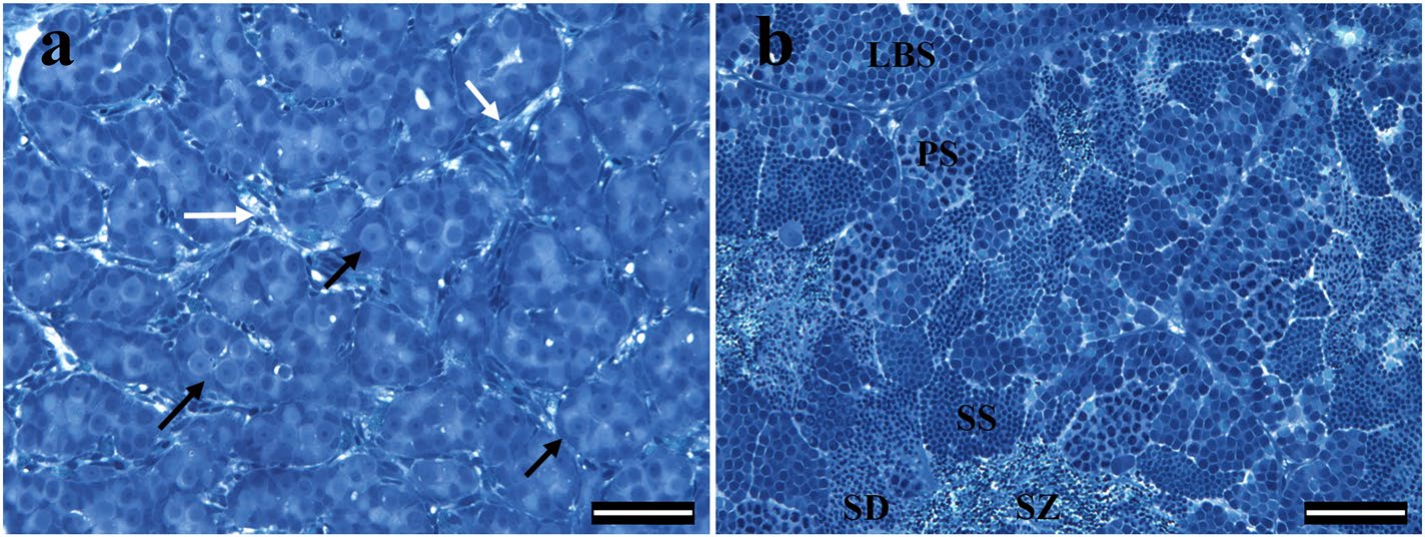
LM images of Richardson-stained MBM-embedded sections showing the general morphology of the testes. **a** A section from a 1-year-old testis shows developing seminiferous tubules containing type A spermatogonia (black arrows) surrounded by Sertoli cells. Leydig cells, blood vessels and red blood cells are shown between the developing tubules (white arrows). **b** A section from a 2.5-year-old testis showing different germ cell types from late type B spermatogonia to spermatozoa within different cysts. Scale bar 50 μm applies to each image. *LBS* late B spermatogonia, *PS* primary spermatocytes, *SS* secondary spermatocytes, *SD* spermatids, *SZ* spermatozoa

The general morphology of the testes we examined at the 1-year and 2.5-year-old stages is shown in Fig. 5. In Fig. 5a we show a low magnification section of a 1-year-old testis with cells organizing into circular seminiferous tubule formations containing type A spermatagonia and Sertoli cells. The Leydig cells are small cells that lie outside of the tubules in the interstitial compartment (Fig. 5a). The spermatogonia are distinguished by their larger nuclei in comparison to the smaller nuclei of the Sertoli cells. A section of a 2.5-year-old testis shows cysts of spermatogonia B (LBS) and primary (PS)/secondary (SS) spermatocytes with spermatids (SD) and spermatozoa (SZ) filling the space within the spermatogenic tubular compartments (Fig. 5b). All the cells within a cyst develop more or less at the same rate, while the various cysts within the testis develop at different rates (Schulz et al. 2010).

### Immunolocalization of GnRH1 in ovaries and testes

**Fig. 6:**
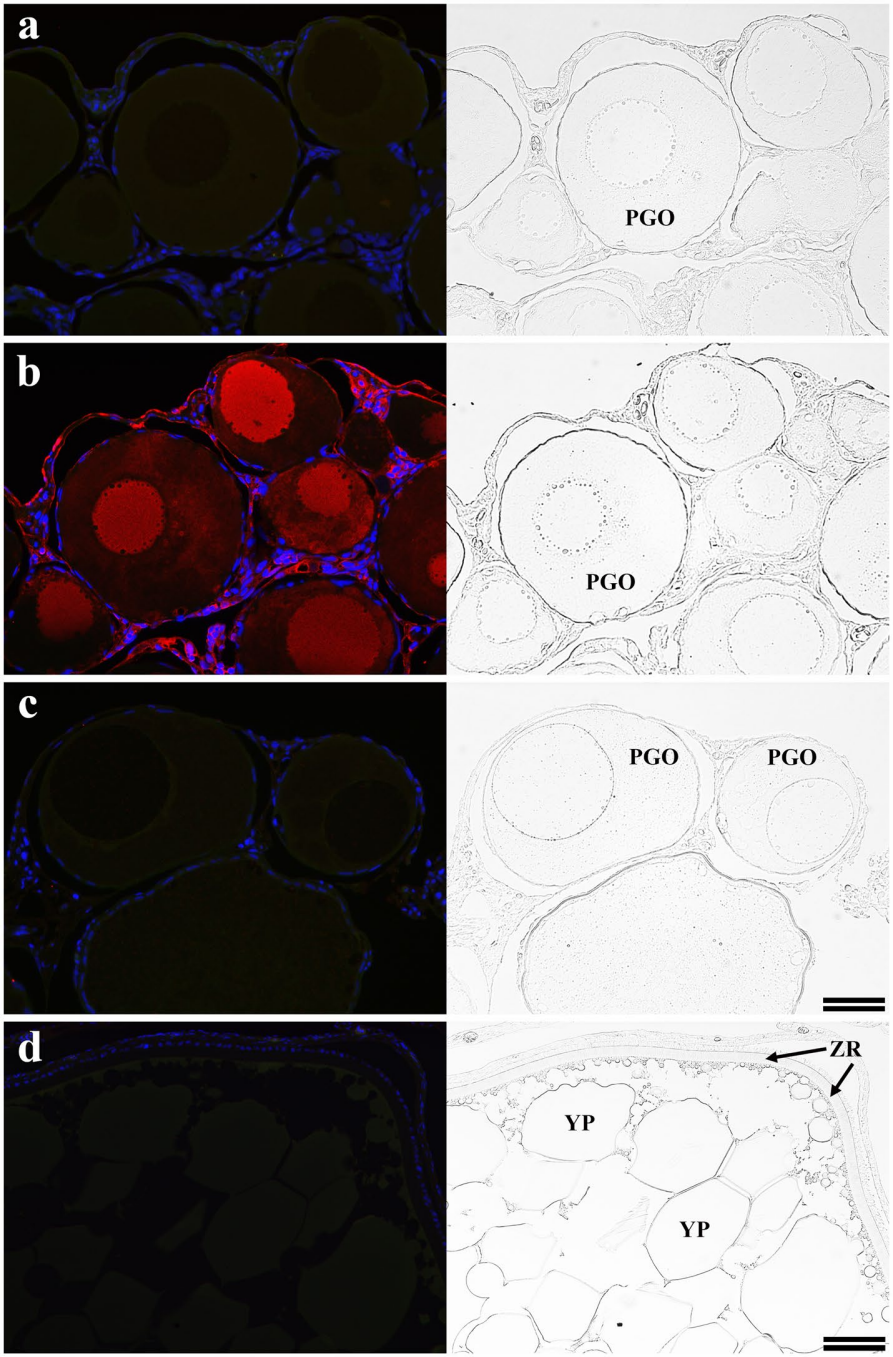
Representative fluorescent and phase-contrast images showing labeling of MBM-embedded ovarian sections. The left sides of each panel **a**–**d** are the fluorescence results while the right sides are their corresponding phase contrast images. **a** One-year-old ovarian section incubated with no primary. **b** One-year old ovarian section incubated with GF-6 primary. **c** Two-and-a-half-year-old ovarian section incubated with GF-6 primary. The scale bar 50 μm in **c** applies to the panels **a**–**c**. **d** Two-and-a-half-year-old ovarian section incubated with GF-6 primary. Scale bar 100 μm. *PGO* primary growth oocytes, *YP* yolk protein granules, *ZR* zona radiata proteins

We examined sections from five individual ovaries and testes obtained from both 1-year-old and 2.5-year-old Atlantic salmon. In Fig. 6, we show representative fluorescent and corresponding phase contrast images of MBM-embedded ovarian sections labeled with GF-6. We demonstrate GnRH immunoreactivity in both the ooplasm and nuclei of oocytes at the early cortical alveoli stage (Fig. 6b). In zebrafish, GnRH immunoreactivity was associated with the ooplasm of primary (perinucleolar) oocytes with diminishing labeling by the cortical alveoli stage (Corchuelo et al. 2017). At the early cortical alveoli stage, we note a large amount of variation in the labeling among the oocytes’ nuclei and ooplasm (Fig. 6b). The nuclei were always labeled, but the ooplasm labeling varied from weak to strong.

In the early zebrafish oocytes, immunoreactive (ir) GnRH was not detected in the follicular cells (Corchuelo et al. 2017). However, we did observe immunolabeling of the thin layer of granulosa cells surrounding the oocytes (Fig. 6b). The cytoplasm of these small cells were well labeled while their nuclei remained unlabelled. We also observed that some regions of the surrounding stroma was heavily labeled. These results may provide an example of blood-borne GnRH positive cells, possibly immune cells, within the surrounding stroma of the 1-year-old ovaries (Fig. 6b). This idea is reinforced by our blood, spleen and head kidney qPCR data that provide evidence for *gnrh1* expression in these hematopoietic tissues (Fig. 3a). No labeling of PGOs or maturing oocytes or any other cell-types associated with the 2.5-year-old ovarian sections was detected (Fig. 6c, d).

**Fig. 7:**
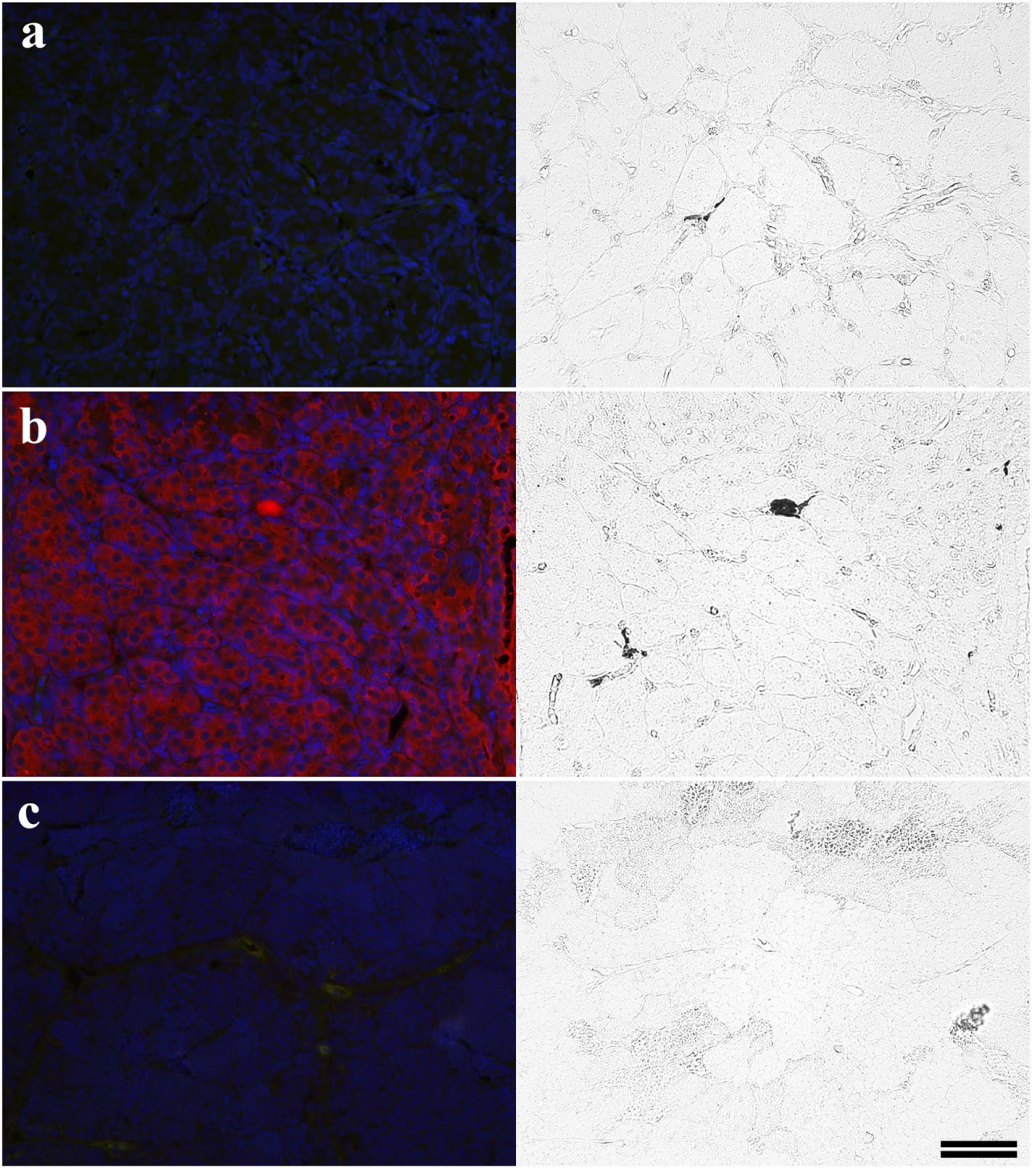
Representative fluorescent and phase-contrast images showing labeling of MBM-embedded testicular sections. The left sides of each panel **a**–**c** are the fluorescence results while the right sides are their corresponding phase contrast images. **a** One-year-old testicular section incubated with no primary **b** One-year-old testicular section incubated with GF-6 primary **c** Two-and-a-half-year-old testicular section incubated with GF-6 primary. The scale bar 50 μm in **c** applies to all panels

In Fig. 7, we show representative fluorescent and corresponding phase contrast images of MBM-embedded testicular sections labeled with GF-6. Labeling of the Sertoli and Leydig germinal support cells, as well as type A spermatogonia, was shown for only the 1-year-old testes (Fig. 7b). In zebrafish, irGnRH was associated with the somatic support cells and was found in most germ cell types, but the labeling was strongest in spermatogonial cells (Fallah et al. 2020). We observed labeling associated with both the spermatogonial nuclei and cytoplasm in some sections, but in other sections only the cytoplasm was labeled. No immunoreactivity of any germ cell type or any other cell-types associated with the 2.5-year-old testicular sections was detected (Fig. 7c).

### Testicular morphology and TEM imaging of Epon-embedded sections

Our general interpretation of the fluorescent LM images of the 1-year-old testes sections was that most of the cells were immunoreactive to GnRH. However, unlike for the ovarian sections, we were unable to precisely evaluate whether GnRH was present in some cellular structures (i.e., nuclei) and cells (i.e., Leydig cells) due to poor membrane resolution and the packed morphological features of the testes. To help visualize the cellular organization more clearly, we examined Epon-embedded sections to provide better morphological quality. Using this approach, our ability to evaluate the organization and the types of cells associated with the developing tubules was improved. This information was then used to help identify more specific localizations of GnRH by TEM.

**Fig. 8:**
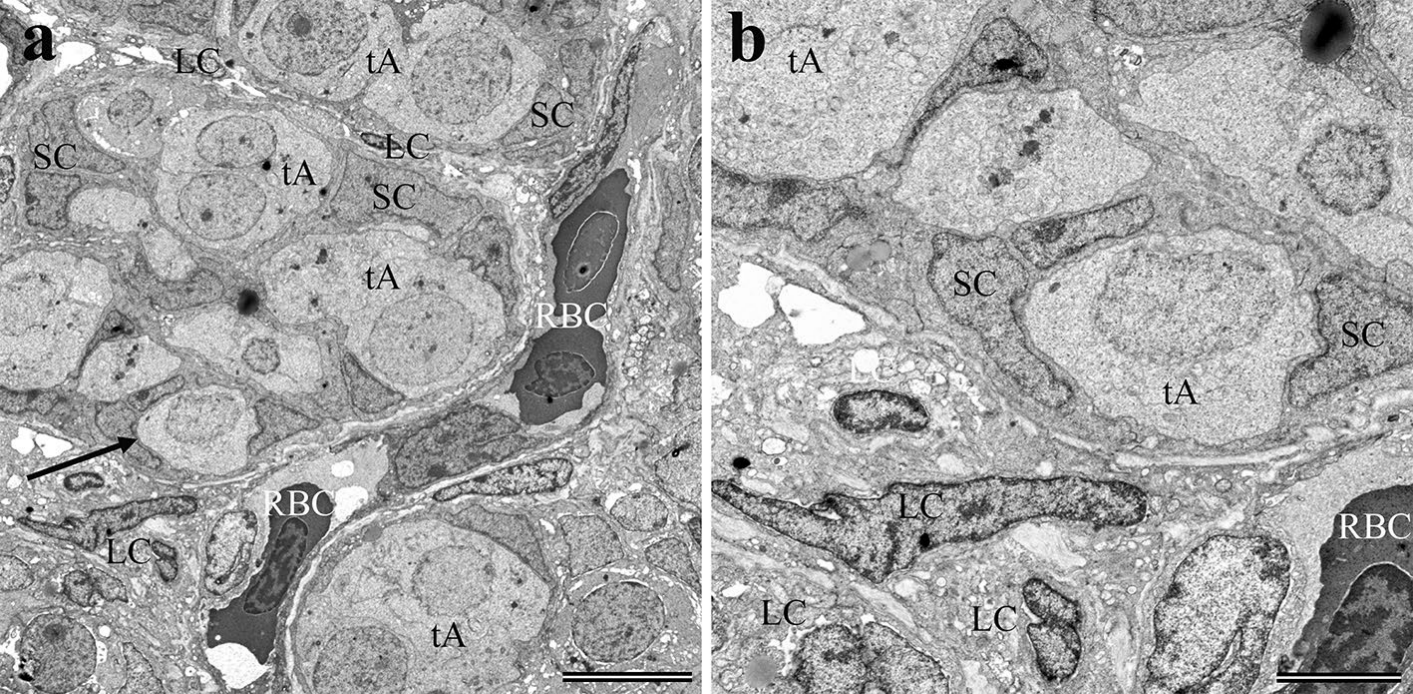
TEM image of stained Epon-embedded sections showing the general morphology of the testes. **a** A section from a 1-year-old testis shows developing seminiferous tubules containing type A spermatogonia surrounded by Sertoli cells. Leydig cells, blood vessels and red blood cells are shown between the developing tubules. Scale bar 10 μm. The arrow points to the area in **a** that is shown at a higher magnification in **b**. Scale bar 4 μm. *tA* type A spermatogonia, *SC* Sertoli cells, *LC* Leydig cells, *RBC* red blood cells

We show examples of differentiated type A spermatogonia (tA) in the 1-year-old Epon-embedded testes sections (Fig. 8a, b). The type A spermatogonia were easily identified by their large round nuclei and accompanying dark compact nucleoli. No examples of type B spermatogonia were observed in any of these sections. Differentiation from late type B spermatogonia to preleptotene spermatocytes is considered the mark of entry into meiosis (Schulz et al. 2010).

The Sertoli cells (SC) and Leydig cells (LC) were distinguished by their darker appearance due to staining of the proteins and lipids that are densely packaged within their cytoplasms. The SC are found in close contact with the spermatogonia and recognized by their rough pyramidal shapes. The amorphous LC were located in the interstitium that lies between the developing seminiferous tubules (Fig. 8a, b).

### Immunolocalization of GnRH1 by TEM imaging of MBM-embedded sections

**Fig. 9:**
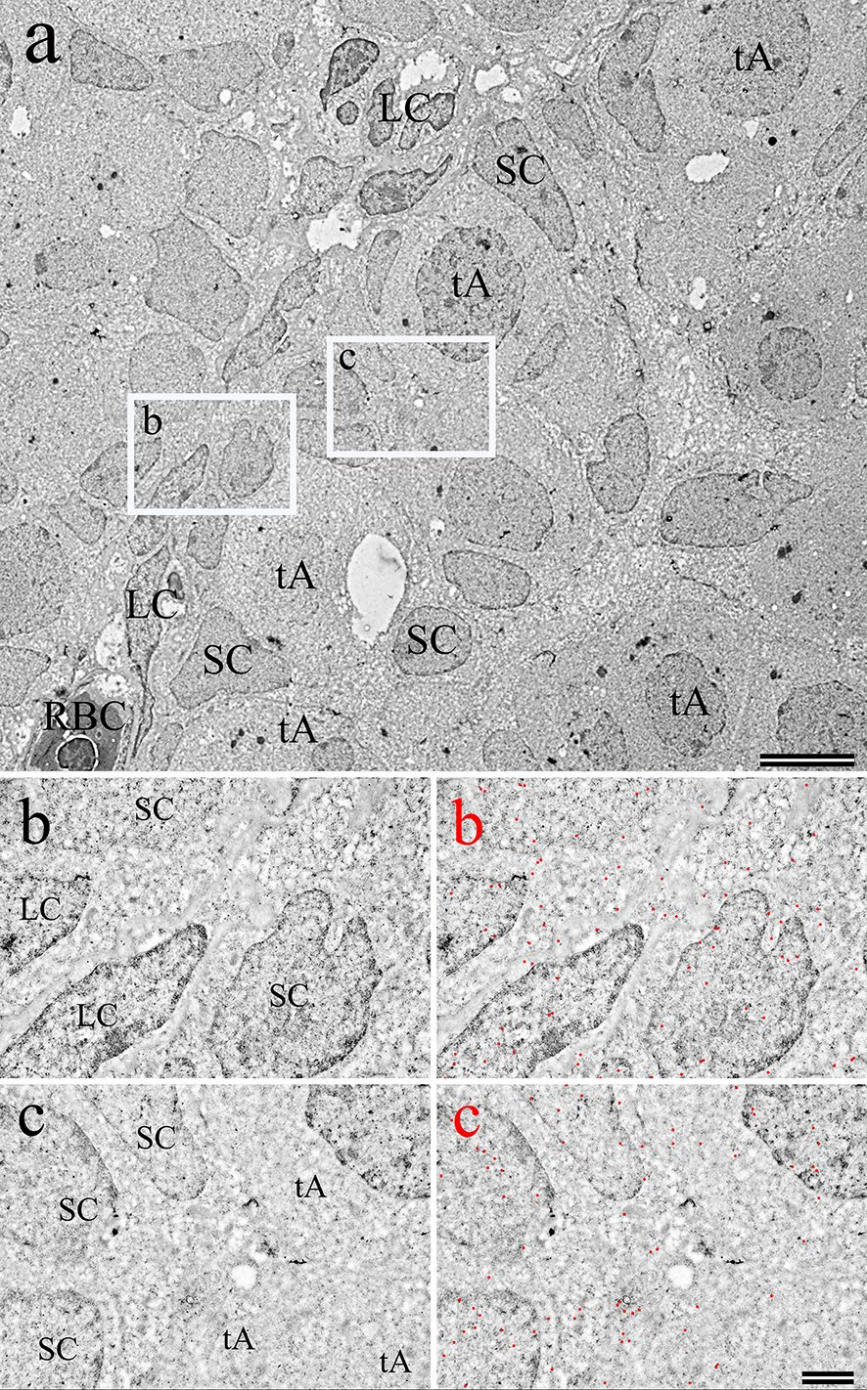
Examination of GF-6 labeling of MBM-embedded testis sections by TEM. **a** Low magnification image showing developing seminiferous tubules. Scale bar 5 μm. The enclosed areas labeled “b” and “c” show the areas highlighted below. **b** Higher magnification of “b” from **a**. The left side shows the raw data and the right side shows positive GnRH immunolocalizations. Electron dense colloidal gold markers have been false colored red to enhance their appearance within the image. Scale bar 1.0 μm. **c** Higher magnification of “c” from **a**. The left side shows the raw data and the right side shows positive GnRH immunolocalizations. The colloidal gold locations have been colored red for clarity. Scale bar 1.0 μm. *tA* type A spermatogonia, *SC* Sertoli cells, *LC* Leydig cells, *RBC* red blood cells

Immunogold labeling of ultrathin MBM-embedded testicular sections for TEM permitted better visualization of GnRH1 localization. Labeling of the cytoplasm of both the SC and LC was detected (Fig. 9b). Both the cytoplasm and nuclei of type A spermatogonia were found to be immunoreactive to GnRH (Fig. 9c). Regions within the cytoplasm of a spermatogonium in Fig. 9c indicates potential accumulation of GnRH1 in association with Golgi apparatus or other subcellular structures.

## Discussion

### General structure of mRNA and translated hormone product

**Fig. 10:**
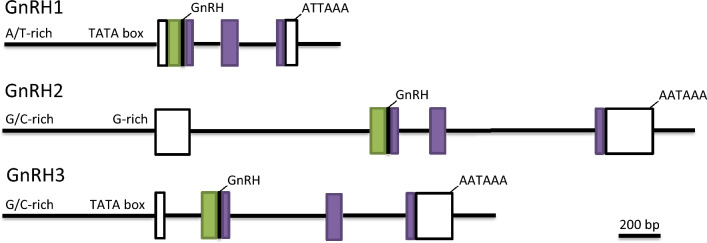
A schematic diagram presenting the organization of the genes encoding the three distinct GnRH forms of the salmonids. The sections that encode the preprohormone are shown by the signal (green box), GnRH (black box) and GnRH-associated peptide (GAP) (purple boxes). Promoter and intronic sequences are represented by horizontal black lines and untranslated regions by white boxes. Some variation exists in the lengths of the sequence that encodes the GnRH1 preprohormone in exon 2 and its terminus in exon 3 among the salmonids (see text for more details). The lengths of each segment of the GnRH2 and GnRH3 genes were averaged and a composite for each paralog is presented

The GnRH1 preproprotein is encoded on three exons, rather than four as for the other *gnrh* types (Fig. 10). However, the fundamental “modules” of the ORF of each GnRH are shared: one exon encodes the signal peptide, GnRH decapeptide and the GnRH-associated peptide (GAP) N-terminal, the next exon the central GAP moiety and the last exon the C-terminal portion of the GAP (Fig. 10). The only difference for the *gnrh2* and *gnrh3* mRNAs is that their 5′-utrs are separately encoded on the first exon.

The functional *gnrh1* homeologs appear more “free” to change within their exonic sequences than do their *gnrh2* and *gnrh3* counterparts (Online Resource 2 and 6). Sequence addition or loss within exons 2 and 3 of the salmonid *gnrh1*s have led to differences in the lengths and the residue composition of the termini of the GAPs (Fig. 10; Online Resource 2 and 6). For example, the length of the complete Atlantic salmon preproGnRH1 is 92 aar, and could exceed 107 aar for rainbow trout, but is only 76 aar for *Salvelinus* spp. (Online Resource 2). For the GnRH2 and GnRH3 hormones, each preproprotein among the species examined here are 86 and 82 aar, respectively [with the exception of the sockeye salmon GnRH3 Gene1 (79 aar)] (Online Resource 3 and 4).

Coding of the residue sequence of the GnRH1 decapeptide also seems more amenable to change among the six salmonids we examined (Table 2) and among vertebrates in general (Roch et al. 2014; Gaillard et al. 2018). Indeed, we report that there are now at least 23 GnRH1 structures distinct from the GnRH first characterized in mammals (Online Resource 8). On the other hand, the salmonid *gnrh2* and *gnrh3* paralogs are more resistant to change, with the GnRH2 and GnRH3 decapeptides remaining identical for each respective paralog (Online Resource 3 and 4).

### Potential regulatory elements of GnRH1 proximal promoter

TFs of one family can interface among themselves and engage with members from other diverse protein families at recognition binding elements within gene promoters. Partnerships between the distinct interactive domains presented by representatives of each of the diverse TF families described here have been well established (Liu et al. 1996; Eisermann et al. 2008; Kamachi and Kondoh 2013; Hill 2016; Malik et al. 2018). Opposing or cooperative interactions by different proteins at composite elements—and formation of multiprotein complexes—may be a common feature in the regulation of specific gene activity (Rhee et al. 1998; Ellsworth et al. 2003; Shi and Massagué 2003; Eisermann et al. 2008; Kamachi and Kondoh 2013; Malik et al. 2018). It is these regulatory elements and the distinct TF compositions that form on them that make transcription of each gene unique to the cell type in which they are expressed.

We have identified at least four different TF families that could play a role in the expression of the salmonid *gnrh1* (Fig. 1b). Interactions of different BRN- and/or OCT-type TF combinations are possible at the composite POU-domain binding elements (−1064 to −1028 and −924 to −900) (Table 1). Members of the POU-domain family of TFs are important in the establishment of specific cell types during embryogenesis and in stem cell maintenance (Malik et al. 2018). We also show the potential for differentiation factors from the EGR, SP and WT families to form complexes in a number of regions of the *gnrh1* proximal promoter (Fig. 1b). Members from these families, including some SMADs, have been shown to bind G + C-rich elements resembling sequence presented in the SMAD/EGR/SP/WT composite element (Liu et al. 1996; Eisermann et al. 2008; Hill 2016). It is possible that members from these different TF families together could integrate signals from various transduction pathways (Beyer et al. 2013; Hill 2016). Also, there are at least three different single binding motifs for SOX factors that could act to bridge with other TF family members during assembly of the transcription machinery.

SOX-2 and OCT-4 regulate pluripotency (Lodato et al. 2013; Malik et al. 2018), and in association with other specific TFs, such as SMADs, direct cellular fate decisions (Beyer et al. 2013). Furthermore, the interchange between SOX and POU factors is central in regulating self-renewal and cell-fate transcription programs (Kamachi and Kondoh 2013; Lodato et al. 2013; Malik et al. 2018). These decisions are directed by different compositions of, and variations in, the SOX/OCT composite motifs presented to the TF complexes that assemble on them (Kamachi and Kondoh 2013; Lodato et al. 2013; Malik et al. 2018). The SOX/OCT elements in the *gnrh1* promoter could therefore be very robust centers for integration of multiple inputs of regulatory activity. Future studies await the determination of the precise combination of different TF family members required to activate GnRH1 expression during early stages of germ cell development.

### Regulation of GnRH peptide expression

Despite research that shows *gnrh* can be transcribed in salmonid gonads during mature reproductive stages (Gray et al. 2002; Madigou et al. 2002), evidence for concomitant GnRH synthesis has been lacking. We think GnRH is functionally active only during naïve/immature gonadal stages, at least in the Atlantic salmon. However, this model may not apply to other fish that have been studied. For example, in the zebrafish, there is evidence for protein expression during all stages of male germ cell development (Fallah et al. 2020). It is also generally accepted that GnRH is expressed throughout the development of the zebrafish oocyte, from pre-vitellogenic to final maturation stages (Fallah and Habibi 2020). However, Corchuelo et al. (2017) found that GnRH expression in the zebrafish ovary was waning by the cortical alveoli stage, even though *gnrh* transcripts were present in later vitellogenic stages. Thus caution should be taken to avoid correlating transcription with translation, particularly in gonadal studies and probably for many of the other salmonid tissues shown to express *gnrh* mRNA (Fig. 3). The post-transcriptional regulatory controls of GnRH synthesis in the germ cell are still unknown.

Our IHC results for the Atlantic salmon, together with collaborating evidence from rainbow trout RIA data, show there may only be a relatively short window of GnRH expression in the ovaries and testes. We know that GnRH1 is expressed during a primary stage roughly associated with gonial proliferation (males) and transition to meiosis (females) (see below). We hypothesize that GnRH synthesis is required during these early stages of gamete development, when decisions for proliferation, self-renewal and entry into meiosis are first being made. The initiation point and the duration of GnRH expression in each germ cell type have not yet been determined.

We also show that GnRH1 peptide is present in the germ cell nucleus during this period in the female (Fig. 6b) and male (Figs. 7b and 9c) germ cell. This indicates that GnRH1 may act directly as a DNA-binding or nuclear regulatory factor—activities that have not been established. Alternatively, GnRH1 may function as a key modulator in a germ-cell-directed steroidogenic network that provides the local hormonal environment required to promote gonial propagation, renewal and meiosis (reviewed by Schulz et al. 2010 and Lubzens et al. 2017).

### Function of GnRH in salmonid gonads

The discovery of GnRH1 in the brains or gonads of salmonids has been elusive. If GnRH1 serves a locally expressed function in later stages of salmonid gonad maturation, during spawning or in the development of the next generation of germ cells, it has never been demonstrated. For example, GnRH peptides have not been detected during examination of gonadal tissues by HPLC/RIAs through either the second or third year of the lives of normal developing trout (von Schalburg et al. 1999b; Gray et al. 2002). Even though the existence of GnRH1 was unknown and the transcript therefore not examined during these previous studies, the antibodies that were used in the trout HPLC/RIA analyses should have detected the third, and in fact all three, GnRH peptides had they been present in the gonads during later stages of normal reproductive development. Furthermore, we did not find immunoreactive GnRH associated with any cell-types of the mature ovaries or testes of pre-spawn Atlantic salmon (Figs. 6c, d and 7c).

We therefore think that the IHC results reported here provide the first evidence that GnRH1 is the only functional GnRH in normal salmonid ovaries and testes. This idea is supported by our expression study that shows an accumulation of *gnrh1* (most strongly in the ovaries) accompanied by very little if any concomitant transcription of *gnrh2* or *gnrh3* products (Fig. 3a, b). Furthermore, the earlier studies mentioned above indicated that transcription of *gnrh2* and *gnrh3*, if it did occur, did not correlate with translation in normal 2-year and 3-year-old rainbow trout gonads (von Schalburg et al. 1999b; Gray et al. 2002). Taken together, we conclude that only GnRH1 is functional in normal salmonid ovaries and testes, and only around the 1-year-old stage, when the gonads are still immature.

For the testes in which GnRH protein was detected, it appears the spermatogonia are in a pre-meiotic, proliferative phase. For the ovaries, the oocytes have entered meiosis and the stage of development corresponds roughly with an early cortical alveoli oocyte stage (Lubzens et al. 2017) (Fig. 4a, b). The testes were less differentiated, with tubules in an early stage of development presenting only primary type A spermatogonia (Figs. 5a and 8). Interestingly, we provide evidence for the translocation of GnRH1 to the nuclei of both the male and female germ cells during these early stages of development (Figs. 6b, 7b, 9c). The mechanisms of the translocation and function of GnRH in the nucleus of the germ cell are unknown.

Nevertheless, there is evidence for a functional GnRH (Corchuelo et al. 2017; Fallah et al. 2020; this paper) gonadotropin (GN) (Wong and Zohar 2004), LH receptor (Chauvigné et al. 2014), estrogen and progestin biosynthetic machinery (von Schalburg et al. 2013, 2018b; Gohin et al. 2011; Caulier et al. 2015; Delalande et al. 2015), ER (von Schalburg et al. 2018b; Zapater et al. 2019) and PR (Zapater et al. 2013a; Chauvigné et al. 2017) network in fish germ cells.

It is therefore conceivable that a GnRH/GN/steroidogenic enzyme signaling axis that is directed by the germ-cell alone could exist early in gametogenesis, at least in some species of fish. Local regulated pathways could lead to the production of steroids, such as estrogens and/or progestins to direct specific processes, such as stem cell renewal, proliferation, differentiation and/or entry into meiosis. The importance of these steroids in guiding these processes during early gamete development has been demonstrated (Amer et al. 2001; Miura et al. 2006, 2007; Chauvigné et al. 2017), but the factor controlling their production has not been elucidated (Lubzens et al. 2017).

We think that glimpses of potential endogenous GN and receptor activity (Zapater et al. 2012; Chauvigné et al. 2014), steroid production (Gohin et al. 2011; Zapater et al. 2012; Caulier et al. 2015; von Schalburg et al. 2018b) and steroid-receptor interactions with functional outcomes (Zapater et al. 2013a, b; Chauvigné et al. 2017) have been reported within fish germ cells. Further work is now required to demonstrate the physiological timing and role of GnRH in this circuitry. A more targeted examination of the primary gonads to determine the earliest expression of GnRH will help us to define the precise stage of germ cell development that may be correlated with native GnRH function.

### Supplementary Information

Below is the link to the electronic supplementary material.

Supplementary file1 (PDF 69 KB)

Supplementary file2 (PDF 170 KB)

Supplementary file3 (PDF 184 KB)

Supplementary file4 (PDF 152 KB)

Supplementary file5 (PDF 90 KB)

Supplementary file6 (PDF 98 KB)

Supplementary file7 (PDF 947 KB)

Supplementary file8 (PDF 72 KB)

## Abbreviations

AP-1: Activator protein-1
cAMP: Cyclic AMP
CRE: cAMP response element
E2: 17-β-Estradiol
ER: Estrogen receptor
ERE: Estrogen response element
FOXL2: Forkhead box L2
GnRH: Gonadotropin-releasing hormone
HPLC: High-performance liquid chromatography
IHC: Immunohistochemistry
MBM plastic: Methyl methacrylate/butyl methacrylate
poly(A): Polyadenylation
PR: Progestin receptor
RIA: Radioimmunoassay
SMAD: Mothers against decapentaplegic
SRY: Sex-determining region Y
SOX: SRY-related high mobility group box
TF: Transcription factor
