# Supplementary material for: The late-evolving salmon and trout join the GnRH1 club
Source: Histochem Cell Biol. 2023 Aug 11;160(6):517–39. doi: 10.1007/s00418-023-02227-z (PMC10700215; doi:10.1007/s00418-023-02227-z)
Supplement: Supplementary file 1 — Supplementary file1 (PDF 69 KB) [file 418_2023_2227_MOESM1_ESM.pdf]

Online Resource 1 Primers used for gene characterization and to assess expression across the multi-tissue panel

Primer Name	Nucleotide sequence (5'-3')	Application	Efficiency (%)	Amplicon size (bp)
GENE CHARACTERIZATION PRIMERS				
gnrh1-GSP	F: TGTCACAGGGTTGCTGTCAACATTGG	3' RACE PCR	N/A	N/A
	R: TCCACTTTGTCTGTCAGCGAGGCTTG	5' RACE PCR		
gnrh1-Nested GSP	F: TGGCTGAAGACCTTCCGAAGATGGA	3' RACE PCR	N/A	N/A
	R: TCAGCCGGTAAATTTCGGCATGAGG	5' RACE PCR		
qPCR PRIMERS				
gnrh1	F: CACAGGGTTGCTGTCAACAT	qPCR	97.3	148
(XM_014160183)	R: GAGACATCAGCACAGCCAAA	qPCR		
gnrh2a	F: GAGTGTGGCTAGGCTGGTGT	qPCR	110.3	75
(NM_001141139)	R: CAGTGTTGGGAGGAGGACAG	qPCR		
gnrh2b	F: CAAGAGGGAGCTGGACTCAT	qPCR	106.5	133
(XM_014132262)	R: GGCCAGGACATCCAAAATAA	qPCR		
gnrh3a	F: ATTGGTCGTATGGGTGGCTA	qPCR	106.2	81
(XM_014206827)	R: CACCTGTGTCCATCATCCTG	qPCR		
gnrh3b	F: GTATGGCTGGCTACCTGGAG	qPCR	91.7	77
(XM_014154459)	R: CACCTCCTGTGTCCATCATCT	qPCR		
eif3d	F: CTCCTCCTCCTCGTCCTCTT	qPCR	97.6	105
(GE777139)	R: GACCCCAACAAGCAAGTGAT	qPCR		
rpl32	F: AGGCGGTTTAAGGGTCAGAT	qPCR	96.1	119
(BT043656)	R: TCGAGCTCCTTGATGTTGTG	qPCR		

GSP gene-specific primers, *gnrh* gonadotropin-releasing hormone, *eif3d* eukaryotic translation initiation factor 3 subunit D (Caballero-Solares et al. 2017), *rpl32* 60S ribosomal protein 32 (Xue et al. 2015). GenBank Accession Numbers are provided below each qPCR primer name

Online Resource 1

The late-evolving salmon and trout join the GnRH1 club

Histochemistry and Cell Biology

Kristian R. von Schalburg, Brent E. Gowen, Kris A. Christensen, Eric H. Ignatz, Jennifer R. Hall, Matthew L. Rise

Corresponding author at: Department of Biology, Electron Microscopy Laboratory, University of Victoria, Victoria, British Columbia, Canada V8W 3N5

E-mail address: krvs@uvic.ca (K.R. von Schalburg)
