# Supplementary material for: The late-evolving salmon and trout join the GnRH1 club
Source: Histochem Cell Biol. 2023 Aug 11;160(6):517–39. doi: 10.1007/s00418-023-02227-z (PMC10700215; doi:10.1007/s00418-023-02227-z)
Supplement: Supplementary file 2 — Supplementary file2 (PDF 170 KB) [file 418_2023_2227_MOESM2_ESM.pdf]

Online Resource 2 Presentation of the GnRH1 genes for grayling, whitefish (*Coregonus* sp. 'balchen'), Atlantic salmon, a *Salvelinus* spp., rainbow trout and sockeye salmon. The 5'-utr, ATG start codons (green), GnRH-encoding sequence, exon/intron GT-AG donor/acceptor sites and poly(A) motifs are highlighted in bold for each gene of interest. The stop codon is highlighted in red for each GnRH1 mRNA. We provide the putative mRNA and protein below each GnRH gene-type presented.

ASM434828v1

Organism name: Thymallus thymallus (grayling)

>gi|1594667058|gb|CM014997.1|:17584065-17586594 Thymallus thymallus isolate TTM2012 chromosome 13B, whole genome shotgun sequence

TAGTTGTATAACAACACTCCCCTCACAATGAATTGCATATAATTCAATCATGTTTTGAA
ATTATACTTTGCACAAAGCTGTCTTAAATGTACATTTTTGTTCATTAACTTGTGTTTTA
CTTACTCGCAGGACACCAAAAGTTAAATGTGGTACTTGTAACAGTGTTTTTTAAATCCA
GTGCTTCCAAAACTATTAGTATAGCAAACCTTTATCATTGCAGGACTGTGATTTTTAAC
AATCTCATGTCAGTTTGTTTAAGAAAATGTCTGCATGTTTAAAGGCTGTATTTTACCAG
TCTTTGACTGGTGAATTTTATCTAGATGTAGCTGAAACCTTTTTGAGATGCAATGTTCT
CTCTCTACACTGCGTGCACAATTATTAGGCAAGTGAGTATTCTGATCTTATCATTATTT
CAATGCACATTTTCCAACTCCAAACCATATAAACTTGAATGCTTTTTGGATTGAATCAT
TTTCAGGTGATATGTATTTGTGTAATGAGGGAGGGTGTGGCGAAAATGAATATCACCTA
TCAAGGTGTGCATAATTATTAGGCAGCTTTATTACCTCTGGTAAAATGGGCCAAAAAAA
TAGATTTAACTGACACTGAAAAGTCAAAAATTGTAAAATGTCTTTCAGACTGATGCAAC
ACTCTTGAAATAGCTAAACTATTGAGGTGTGACCACCGGACAATCAAACGTTTTGTTGC
GAATAGTCAACTGGGGCGCAAAAAACGCATGGAGAAGAAAAGGTGCAAATTAACTGCAA
AAGACTTGAGAAGAATTAAACGTCAAGCTACCAGGAACCCATGATCCTCCAGTGCTCCA
TATTCCAGAACTGTAACCTACCTGGAGTGTCCAGAAGTATTGGGCAATGAAATACCTGA
AGACAGATTTTTCAAAGGTTTTATGGACAGATTAAATGAGAGTGACTCTTGATGGACCA
AATGGATTGGCCCGTGGCTGGATCAGTAATGGACACAGGGCACCACTTCGAGTCAGGCG
CCAGCAAGGTGGAGGAGTGGTACTGTTATGGGCTGCTATCATTAAGGATGAGGTAGTTG
GACCTTTTCGGGTTGAAGATGGACTGAAACTCCCAAACCTACTGCCAGTTTCTGGAAGA
TACTTTCTTCAAGCAGTGCTACAGGAAGAAGTCCTCAGCATTCAAGAAGGCCATGGTCT
TTATGCAGGACAATGCTCCATCACATGCATCCAAGTACTCCACTGCTTGGCTAGCCAGC
AAGGGCCTCAAACATGCCTGAATAATGACCTGGCCCCCTTCCACACCTGACTTAAATCT
TATTGAGAACTTGTGGCCCCTTCTCAAACGTGAGATTTGCTTTGAGGTTAGACAATACA
CCTCTTTGAACAGCATTTGGGAGGCTGTGGTTCCTGCGAAAGTTGATTGTGAACAGATC
AAGAAACTGACAGACTCCATGGATGGAAGGCTCATGGCAGTTATTGAAAAGAAGGGTGG
CTATATTGGTCGCTGAATATTTTTGAAAGGCCAAATTTTGTGTTACTTACACTGTTACA
CTTAGTCTAACAATTGAGAATAAACAAGTGAGTTGGGATAAATTATTTTTGTAATAATT
GTGCACACTTACAGTTGAAGAAGTTTACATACACTTAGGTTGAAGTCATTAAAACTCAT
TTTTTCAACCACTGCACAAATTTCATGTTAACAAGATATCGTTTTGGCAAGTCTGTTAG
GACATCTACTTTGTGCATGACACAAGTAATTCTTCCAACAATTGTTTACAGACAGATTA
TTTCACTTATAATTCACTATATCACAATTGCAGTGAGTCAGAAGTTTACATACACTAAG
TTGACTGTGCCTTTAAACAGCTTGGAGAATTCCAGAAAATTATGTCATGGCTTTAGATG

CTTCTGATAGGATAATTGACATAATTTGAGTAAATTTGAGGTGTAACTGTGGATGTATT
TCTCCAAAAAGTATGATGTAAGATCATACTTTTTGGAGAAATGTCCTCTGGTCTGATGA
AACAAAAATAGAACTGTTTGGCCATAATGACCATCGTTATGTTTGGAGAAAAAGGGGGA
GGCTTGCAAGCCGAAGAACACCATCCCACCCGTGAAGCGTGGCAGCATCATGTTGTGGG
GGTGCTTTGCTGCAGGAGGGGCTGGTGCACTTCACAAAATAGATGGCATCATGAGGAAG
GAAAATTATGTGGATATATTGAAGCAAAATCTCAAGACATCAGTCAGGAAGTTAAAGCT
TGGTTGCAAATGGGTCTTCCAAATGGACAATGACCCCAAGCATACTTCCAAAGTTGTGG
CAAAATGGCTTAAGGACAACAAAGTCAAGGTATTGGAGTGGCCATCACAAAGCCCTGAC
CTCAATCCTATAGAACATTTGTGGGGAGAACTGAAAATGCGTATGCGAACAAGGAGGCC
TACAAACCTGACTCAGTTACACCAGTTCTGTCAGGATGAATGGGCCAAAATTCACACAA
CTTATTGTGGGAAGCTTGTGGAAGGCTACCTGAAACGTTTGACCCAAGTTAAACAATTA
AAGGCAATGCTACCAAATACTAAAAGAGTGAATGTAAACTTCTGACTCTCTGGGAATGT
GATGAAAGACATAAAAGCTGAAATAAATCTGACATTTCACATTCTTAAAATTAAGTGTT
GATCCTAACTGACCTAAAACAGGGAATTTTTACTAGGATTAAATGTCAGGAATTGTGAA
AAACTTTAGTTGAAATGTATTTGGCTAAGGTGTATGTGAACTTCTGACTTCAACTGTAT
ATATTCCCCAAAGAAAGACAAAACTCACTTTTCCTTTGTTAAACATTCAGCTTTGAGGT
TCAATAACATTTTGTATTGACTGAGAGCATTGTGTTCGTTCAACAATAAAATGAATCCT
GAGTAATACAATTTGCCTAATAATTGTGCACGCAGTGTATACTCTATTCCACAGTAGGT
ACTCTTCTGTCATGAAGAATATTGCACCTTGAGTGTGGAGATTGTCAATATTGGAACCA
TGTAGAAAAGAACAATGTTTATTTATTTTGAAATAACTTATTGAAAAATATGGAATGAA
AGTTAGACTGAGTGTTTCTCTTCCACCCCGTCTATAATTATTGCCACTCAAGCACAAAA
CAGTAATTTATTGAAATACATTTATCAATACACAAACGCCACTGTGTAGAAGAACACCA
AGACCTTGTGCCTAATTCATTTCATCGTTGCTGCTGTAAAAGTGCCCAATCATTCCCAA
ACTATTGTTAAGCTTTTAAAAGGCTCTTGGGGTGCCCGGTGCCTGAAGTGGAGGTGCTT
AGTGCTGCTCTGTAATATATTTTCTGAGTGGAGCCTTTTTCTGTTATCCACTTTGTGAC
CTCCTGTGGCGAGGTGATGGGACTTGTGGTGGTGGGGCACATGGGAACCAAGCTGCCTA
TAAAACCTCAGACATGAGTTGCTC**CACTAGAGTTATAAGGCTGACTTTGTAGAATG**GAA
GAGAAAAAAGTACTGTTGTTGCTGCTGCTCTTGGCGGTGGCTCTAGTGTCACAGGGTTG
CTGT**CAACATTGGTCCTATGGCATGAACCCAGGG**GGGAAAAGAGCGACTGGCAGCCTGT
CTGACACCCTGGACAAT**GT**AAGTACTTTACCTATTCATAATGTTGAAGCAGAAGTAGCT
AGGTATATTTACTAAAGGTTGTGATGGTAAAGTATTAAATGATGGGAGGGATGTTCCTA
TATTTGCATCGTAAAATCTAGCTAAATGATCATTCAGACCTGGAACTGTTAGGGTGTTA
GGCCCTAGTTTAACAATTATGCATCCATCCGTGAGTAACTGGTCTGTAATCCCTCATTC
ACTCAGGACTTATTTATTCATTCAAGTCTGTGTATACATATTCATGTTCAGTTGTTATA
TCACGTGCAATATTGGAAGTAATTATTGGACTGAACTTACATAGTCATTAACTCAGGAA
TGCATATTAGTATCCTGTTAGTTGTATCTTTTTTTTTTTCATTGCTTTTAGTATATCGAA
GGTGCAATACGTGCATTTATCTGTAATTAAACCCTGCACTAAGGACTGTATTTTAAGGG
AATTACTATTATTACCCTAAATTATATAATACACACATTTAGCTATTTAAGTATCTCTT
CCTGTCATTTACCTGTACAGTTAATGTAACCATGCAAATATTCACTTACCTGTAAATGT
AACTGTATGTTTACCTGTACAGTTAATTTAACCATGCAAAATATTCACTTACCTGTTCA
TTTAACCAGACTGTATATTAAGTAAACTATTACTACCCTCACTTATATCATACGCACTT
TTAGCTATTTAAGTATTTCTCTGCCATCGACCTGTATAATATAGTTAACCATGCAAATA
CTCCTTATTTATCTATCAACTTGCAATATTTTAATCATTTTTTTCACCTAACGCTCTGT
AACAATGTATAGTGTACATATTCAGCCATCCACCTATACTATGCAATGTCTTTTTATAG
CTTTTTGCACTCCAGACTTTTTCATACTGCCGCTCTATGTATCGTGTATCTGTCTTTTG
TGTATTTTCATTCTGGCATTTTGCACCTGCCCTGTTTTTTCGTAGCTGCTTGACAAAGA
AATTTCCCCTCTGGGATCAATAAAGTATATATCTATCTAATCTATATATTGTATCTATT
CTA**CAG**ATGGCTGAAGACCTTCCAAAGATAGACACATCTTGCAGTTTGTTTGGCTGTGC

TGATGTCTCACCTCACGCCAAAATGTACAGGCTGAGGGCATTACTT**GT**GAGTGACATTT
ACAATTTGCATATACTGTACATTTCCCTTCAAAAAATGTGTTTTTTCTCAATGTTTTTA
ACAAATTTTATCCAAATTGTAAATTATGTTTTTCCATGTACTTTTTTTCT**AG**GCAAGCC
TCGCTGACAGACAAAGTGGACTCAATAATAATAATAATGCTAGCTAACTCT**ATTAAA**CA
TTGCAGTGCCATCATTGTGTTTTGTGGTGGTCTTTGTTGGGGGCCCATTTTTGTTGATT
TGCATATTATATAGTATGGCTTTAAAACAGTGGAACACGATGTCAA**AATAAA**AGTGACA
ACGAATTATTGCGACTGAGTTACGTTGTTCCTGCAAACCTTTATTTTGAAAATGTCGAC
AGCACGGCTTTTATGTCGAGTTCCTGTGTACACATGTTTATCACGGGTTAGTTTTAAAC
AAGGATTTGTGTTCATTCTGCGGTGGTTGTTCAAGATTATTAGCTCTGAGTCCAGAATT
TCCAGCTAATTAGCTAAACTCGAGCTCAGTTACTTTGAGGGCACGTTTGCAGACAATCG
TCTGGTTTTGCTAGCAACTGTGTTAGCTAGTTTCGAGAGCTATCTAACGTTAGCTGTAG

mRNA:

ACTAGAGTTATAAGGCTGACTTTGTAGAATGGAAGAGAAAAAAGTACTGTTGTTGCTGC
TGCTCTTGGCGGTGGCTCTAGTGTCACAGGGTTGCTGT**CAACATTGGTCCTATGGCATG**
AACCCAGGGGGGAAAAGAGCGACTGGCAGCCTGTCTGACACCCTGGACAATATGGCTGA
AGACCTTCCAAAGATAGACACATCTTGCAGTTTGTTTGGCTGTGCTGATGTCTCACCTC
ACGCCAAAATGTACAGGCTGAGGGCATTACTTGCAAGCCTCGCTGACAGACAAAGTGGA
CTCAATAATAATAATAATGCTAGCTAACTCT**ATTAAA**CATTGCAGTGCCATCATTGTGT
TTTGTGGTGGTCTTTGTTGGGGGCCCATTTTTGTTGATTTGCATATTATATAGTATGGC
TTTAAAACAGTGGAACACGATGTCAA**AATAAA**AGTGACAACGAATTATTGCG

protein:

MEEKKVLLLLLLLAVALVSQGCC**QHWSYGMNPG**GKRATGSLSDTLDNMAEDLPKIDTSC
SLFGCADVSPHAKMYRLRALLASLADRQSGLNNNNNAS

It is not clear if the end of the protein is correct since there is a potential sequence problem that leads to the string of asparagines (Ns) at the protein terminus. There may be a small segment of DNA (TAATAA) that has been assembled erroneously, just upstream of the stop codon. This additional segment is not present in the other salmonids.

Also, unlike for the whitefish (*Coregonus* sp. 'balchen'), the grayling gnrh1 mRNA does present a poly(A) motif (ATTAAA) similar to that presented in the late evolving salmonids. The whitefish gnrh1 uses the more downstream AATAAA poly(A) motif (Vickers et al. 2004). However, its protein terminates in roughly the same position as shown for the late evolving salmonids.

AWG_v1

Organism name: Coregonus sp. 'balchen' (whitefish)

>gi|1711366635|emb|LR664361.1|:23120119-23121777 Coregonus sp. 'balchen' genome assembly, chromosome: 18

TCGTTTTGTTTGGATGTTCTCTGTGAATCTACCTCCTTATTGTTTTTACCCCTTTCTGA
TGTATCTCTAGTTGCATGCATTTCACAGTGGTTTTTGCTTCTTGTATGAAATCCATTAT
TCATCATATCCAAATATGATAAGCAAATAATCAAAGTGAATAAGATGTTTTTTTAATAG
TGTTACTGTCATGTTCAAGCTTGTGGATCACCTAGTTGTACGAGGAAGATAGTTGTATA
ACAACACTCCCCTCACAATGAATTGCATATAATTCAATCATGTTTTGAAATTATACTTT
GCACAAATCTGTCTTAAATTAAAAATGTTGTTCACCGTCTTCTGTTATACTCACTCGCA
GGTCACCAACAAGTAGAATGTGGTTCTTGTAACAATGTTTTTTATTTTTTTTAATCCAG
TGCTTCCAAAACGTGTAAATTATTAGGATAGCAAAACATTATCACTGCTGGACTGTGAT
TTTAACAATCTCATGTCAGTTTGTTTAAGAAAATGTCTGCATGTTTAAAGACTGTATTT
TACCAGTCTTCGACTGGTGAATTTCCTCTAGGTGTATCTTTTTGAGATGCAATATTCTC
TCTCTATACTCTATTCCACAGTAGGTACTCTTCTGTCGCAATAATGAAGGCAATCATAA
AGAATATTGCACCTTGAGTGTGGAGATCGTCAATATCAGAACCTTGTAGAAAAGAACAA
TGGTTATTTATTTTGATATAACATATTGAAGAAATATGGAATGAAAGTTAGTTAAAGAC
TGGGTGTGTTTCTCTTCCTCCCCTGTCTATAATTATTGCACTCAAGCACAAAATTGTAT
TTCAATAAATTACTGTTCTCAATAAACAAAAGCCACTGTGTAATTACTTGTGCAAATGA
AATACTGGTATCCTTTCACTAACTTCCCAGAAGAACACCAAGACCCTGTGCCTAATTCA
TTTCATTGTTGCTGCTGTAAAAGTGCACAATCGTTCCCAAAGTATTGTTAAGCTTTTAA
AAGGCTCTTTGCGGTGCCTGAAGTGGAGGTGCTTAGTGCTGCTCTGTAATATATTTTTG
AGTGGAGCCTTTTTCTGTAATCCACTTTGTAACCTCCTGCAACTAGGTGATGGGGCTGG
GGGGTGGGGCACATGGGAACCAAGCTGCCTATAAAACCTCAAAGACACATGAGTTGCTC
AGCTAGAGTAATAAGGCTGACTTTGCAGAATGGAAGAGAAAAAGGTCCTGTTGTTGCTG
CTGCTTTTGGTGGTGGCTCTAGTGTCACAGGGTTGCTGT**CAACATTGGTCCTATGGCAT**
GAACCCAGGGGGGAAAAGAGCGACTGGCAGCCTGTCTGACACCCAGGACAAT**GT**AAGTA
CTTTACCTATTCATAATGTGGAAGCAGAACTAGCTAGGTCTATTTACTAAAGGTTGAGA
TGGTAAAGTATACAATGATGGAAGGGATGTTCCTATATTTGCATAGTATAATCTGGCTA
AATGACAATTCTA**CAG**ATGGCTGAAGACCTTCTGAAGATAGACCCTTGCAGTTTGTTTG
GCTGTGCTGATGTCTCACCTCATGCCAAAATGTACAGGCTGAGGGCATTACTT**GT**GAGT
GACATTTACAATTTTGCATATACATTTCCCTTCAAGAAGCTACCCAGAAGTTGAATATA
AAGCGAAATTGTGTTGGTAGTTCAATATTGCTACTTTATTTAATGTATGTGTTTTTTTC
TAAATTTCTTCAATTTTTATCCAAATTGTAAATTATGTTTTTCCTTTACTTTTTTGT**AG**
GCAAGCCTCGCTGACAGACAAAGTGGACTCAATAATATATAGCAAATGTATGCTAGCTA
ACTCAATGAAACATTGCAGTGCCATCATTGTGTTTTATGGTGGTCTTTGTTGGGGGTCC
CATTTTTGTTGGTTTGCATATTACATAGTATGGCTTTAAAACAGTGGAACACGATGTCA
A**AATAAA**AGTGCCAACACATGATTGCGAGAATGAGTTACGTTGTCGACTCTTGTAGCAA
ACCTTTATTTTGAAAATGTCGACAGCATGGCTTTTATGTCGAGTTCA

mRNA:

AGCTAGAGTAATAAGGCTGACTTTGCAGAATGGAAGAGAAAAAGGTCCTGTTGTTGCTG
CTGCTTTTGGTGGTGGCTCTAGTGTCACAGGGTTGCTGT**CAACATTGGTCCTATGGCAT**
GAACCCAGGGGGGAAAAGAGCGACTGGCAGCCTGTCTGACACCCAGGACAATATGGCTG
AAGACCTTCTGAAGATAGACCCTTGCAGTTTGTTTGGCTGTGCTGATGTCTCACCTCAT
GCCAAAATGTACAGGCTGAGGGCATTACTTGCAAGCCTCGCTGACAGACAAAGTGGACT

CAATAATATATAGCAAATGTATGCTAGCTAACTCAATGAAACATTGCAGTGCCATCATT
GTGTTTTATGGTGGTCTTTGTTGGGGGTCCCATTTTTGTTGGTTTGCATATTACATAGT
ATGGCTTTAAAACAGTGGAACACGATGTCAA**AATAAA**AGTGCCAACACATGATTGCG

protein:

MEEKKVLLLLLLLVVALVSQGCC**QHWSYGMNPG**GKRATGSLSDTQDNMAEDLLKIDPCS
LFGCADVSPHAKMYRLRALLASLADRQSGLNNI

ICSASGv2

Organism name: Salmo salar (Atlantic salmon)

>gi|925216702|ref|NC_027319.1|:23134738-23136448 Salmo salar isolate Sally breed double haploid chromosome ssa20, ICSASG_v2, whole genome shotgun sequence

TTGTTTTGTATGGTGTTCTCTGTGAATCTACCTCCTTATATTTTACCGCTTTCTGATTT
ATATCTAGTTGCATGCATTTCATGGTGGTTTTTGCCGCTTGTTGTATGAAAACCATTAT
TCATCATATCCAAATATGATAATCAAAGTGAATAAGATGTTTTTTAATCATGTTACTGT
CATGTTCAAGCTTGTGGATCACCTAGTTGTACGAGGAAGATAGTTGTATAACAACACTC
CCATCACAATGAATTGCATATAATTCAATCATGTTTTGAAGTTATACTTTGCACAAATC
TGTCTTAAATTAAACATTTTGTTCACTAACTTCTGTTTTACTTACTCGCAGGACACCAA
AAAGTTGAATGTGTTGCTTGTGGTGTTTTTTTTTTATTTTTATTTTTTTTATAAATCTAG
TGCTTACAAAACTTGTGCGTTTTATAAGGATAGCAAAACATTATCACTGCTGGACTGTG
ATTTTAACAATCTGTCAGTTTGTTTAAGAAAATGTTTGCATGTTTAAAGACTGTATTTT
ACCAGTCTAAGACTGCAGAATTTTTTTTCTAGGTGTAGCTGAGACCTTTTTGAGATTCA
ATACTCTATTCCACAGTAGGCACTCTTTTGTCGTGATAATGAAGGCAATCATAAAGAAT
ATTGCATCATGAGTGTGGAGATCGTCAATATCAGGACCTTGTAGAAAAGAACAATGGTT
ATTTATTTTGATATAACATATTGAAGAAATATGGAATGAAAGTTAGTTAAAGACTGGGG
GTGTTTTTCTTCCTCCCCTGTCTATAATTATTGCACTCAAGCACAAAAATGTATTTCAA
TAAATTACTGTTATCAATAAACAAAAGCCACTGTAATTACAAATGAAATACTGTTATCT
TTTCACTAACTTCCTAGAAGAACACCAAGACCATGTGCCTAATTCATTTTATCGTTGCT
GCTGTAAAAGTGCACAATCATTCCCAAAGTGTTAAGCTTTTAAAAGGCTATTTGGGGTG
CCTGGTCTCTGAAGTGGAGGTGCTTAAAGCTGCTCTGTAATATATTTTTGAGTGGAGCC
TTTTTCTGTAATCCACTTTGTGTCCTCCTGCAGCAGTGATGGAACTGGTGGGTGTGGCA
CATGGGAACTAAGCTGCCTATAAAACCTTAAGACACCTGAGTTGCTT**ACCAAAAGTAAT**
AAGGCTGACTTTGCAGAATGGAAGAGAAAAAGGTCTTGTTGCTGCTGCTTTTGATAGTG
GCTCTAGTGTCACAGGGTTGCTGT**CAACATTGGTCCTATGGCATGAACCCAGGG**GGGAA
AAGAGTGACTGACAGCCTGTCTGACACCCTGGACAAT**GT**AAGTACTTTACCTATTCATA
ATATGGAAGCAGAAGTAGCTAGGTCTTTTACTAAAGGTTGTGATGGTAAAGTATCCAAT
GATGGAAGGGATGTTCCTATATTTGCATTAAGTAATCTAGTTTAAAAAATCTAGTTAAA
ATGACAATTCTA**CAG**ATGGCTGAAGACCTTCCGAAGATGGACACATCTTGCAGTTTGTT
TGGCTGTGCTGATGTCTCACCTCATGCCGAAATTTACCGGCTGAGGGCATTATTT**GT**GA
GTGACATTTACAATTTTGAATATTCATTTCCCTCCAAGAAGTTACCCAGAAGTTGAATA
TAAAGGGAAATTGTGTTGGTAGTTAAATATTGCAACTTTTTTTTAATGCATGTTTTTTT
CTCTCAATGTTTTAAACAATTTGTATCCAAATTGTAAGTAATGTTTTTCATTTACTTTT
TTCT**AG**GCAAGCCTCGCTGACAGACAAAGTGGACTCAATAATATATAGCAAATGTATGC

TAGCTAACTCT**ATTAAA**CATTGCCGTGCCATCATTGTCTTTTGTGGTGTCTTTGTTGGG
GGACATTTTTGCTACATCGCATATTACATGGTATGGCTTTAAAACAATTTAACACGATG
TCAAAATACAATGACGAATAGGCCATCATTGA**AAATAA**GAATTTGTTCTTAACTGACTT
GCCTAGTTAAAAAAAAAAAAAAGCCAACAAATGATTGCCTGTATGAGTTACGTTGTTGAC
TCTTGTAGCAAACCTTTATTTTGAAAATATTGACAGCATGGCTTTAATGTCGAGTTCCT
GTGTACACATGTTCATCACGGGTTAGTTATACAAGTCTTTGTGTACATTCTGTGGTGGT
TGTTCAAGATTATTAGCTTTGAGCCCAGAATGTCCAGATAAATAGCTCAACTCAAGCTC
AGTTACTTTGAGGGACACGTTTGCAGACAATCGTCCGGTTTTCGCTAGCATCTGTGTTA
GCTACTTATGAGAGCTAGCTAGCTGTAGCTAACAACATTAGCTAGCTAACGTTAGCAAG
GTACAGTAGTGGGACTTGGAAGCCAACTACTGAGTGCAACGTTTCCTCTATTTTCGTGC
TAGCATTACAAACTTCGGCAGACATCGAGATATAAGAAATACCACATTTGTTTTTCAAA
TGCGTTATATATATTTTTTTTTACAAGAACATAAACAACTGCACTGAAGACTGCAATGTC
GTCTGGAGGCCATCAATGTAACGTTAGGTGTTGTTCCCACCAACTTGCGTCCCCCAGTG

mRNA:

ACCAAAAGTAATAAGGCTGACTTTGCAGAATGGAAGAGAAAAAGGTCTTGTTGCTGCTG
CTTTTGATAGTGGCTCTAGTGTCACAGGGTTGCTGT**CAACATTGGTCCTATGGCATGAA**
CCCAGGGGGGAAAAGAGTGACTGACAGCCTGTCTGACACCCTGGACAATATGGCTGAAG
ACCTTCCGAAGATGGACACATCTTGCAGTTTGTTTGGCTGTGCTGATGTCTCACCTCAT
GCCGAAATTTACCGGCTGAGGGCATTATTTGCAAGCCTCGCTGACAGACAAAGTGGACT
CAATAATATATAGCAAATGTATGCTAGCTAACTCT**ATTAAA**CATTGCCGTGCCATCAT

protein:

MEEKKVLLLLLLIVALVSQGCC**QHWSYGMNPG**GKRVTDSLSDTLDNMAEDLPKMDTSCS
LFGCADVSPHAEIYRLRALFASLADRQSGLNNI

ASM291031v2

Organism name: Salvelinus spp.

>gi|1340979591|ref|NC_036872.1|:16608763-16610800
Salvelinus spp. isolate IW2-2015 linkage group LG33,
ASM291031v2, whole genome shotgun sequence

ATTTGCACTAGAGCACATTCAGGGATATTTGTTTCTGTTATGTAGTGGACGCTTTTACC
TGCCTTTCCTTATTTATCCCCAAAACCAAAATATTTCATGTATGTTTACTATTATGTCT
GGATTCAATCCTACCTTATTACTATTTTTTTTTAATTCTAGATGTATACACACTTGATTA
TTTAACGCAACATCTCTGAAAGTCAAAGATTATTAAACTAGTAAGTGCATGAAGGTTTA
AAGTAACAATATAAGGGCATGGTGGGACTGTACATTCTTATCAATGTACTTAGTTTTAT
GCCAGATATGCTTTATGATTAGGATAATGTTTTTTTTTTTCTAAGCCAAAGCCATACTGT
AGCATGATAAAAATGGCAAAAGAGTTTGCTGTTATTGCACCACCTAATTATGCTGTGGA
TTTCATTTTTCTTATAATACTGTGGCTGTTGAAACAGAATTTCAAGAATGCAATGTACT
CTTCCTTGATAATCACTGAAGTCAAAAGCTGCCTCTTGTGGTGTTTGGTGTTCTCTGTG
AATCTACCTCCTTATTTTTTTACCGCTTTCTGATTTATATCTAGTTGCATGCATTTCATG

GTGGTTTTTGCCTCTTGTTGTATGAAAACCATTATTCATCATATCCAAATATGATAATC
AAAGTGAATAAGATATTTTTTAATAGTGTTACTGTCATGTTCAAGCTTGTGGATCACCT
ACTTGTATGAGGAAGATGGTTGTATAACAAAACTCACACCACAATGAATTGCATATAAT
TCAATCATGTTTTGAAGTTATACTTTGCACAAATCTGTCTTAAATTAAACATTTTGTTC
ACTAACTTCTGTTTTATTTACTCGCAGGACACCAAAAAGTTGAATGTGGTGCTTGTAGT
GTTTTTTTTTTTTTTTATCCAGTGCTTACAAAACTTGTGCGTTTTATAAGGATAGCAAA
ACATTAGCACTGCTGGACTGTGATTTTAACAATCTGTCAGTTTGTTTTTGCATGTTTAA
AGACTGTATTTTACCAGTCTACGACTGCAGAATTGTTTTCTAGGTGTAGCTGAAACCTT
TTTGAGATGCAATATTCTCTCTCTATACTCTATTCCACAGTAGGTACTCTTTTGTCATG
ATAATGAAGGCAATCATAAAGAATATAGCACCTTGAGTGTGGAGATCGTCAGTATCAAG
ACCTTGTAGAAAAGAACAATGGTTATTTATTTTGATATAACATATTGAAGAAATATGGA
ATGAAAGTTAGTTCAAGACTGGGGTGTTTCTCTTCCTCCCCTGTCTATAATTATTGCAC
TCAAGCACAAAAATGTATTTCAATAAACAAAAGCCACTGTGTAATTACAAATGAAATAC
TGTTTTCTTTTCACTAACTTCCCAGAAGAACACAAAGACCATGTGCCTAATTCATTTTA
TCGTTGCTGCTGTAAAAGTGCACAATCATTCCCAAAGTGTTAAGCTTTTAAAAGGCTAT
TTGTGGTGCCTGAAGTGGAGGTGCTTAAAGCTGCTCTGTAATATATTTTTGAGTGGAGC
CTTTTTCTGTAATCCACTTCGTGTCCTCCTGCAGCAGTGATGGAACTGGTGGGTGTGGC
ACATGGGAACTAAGCTGCCTATAAAACCTCAGACACCTGAGTTGCTT**ACCGAAAGTAAT**
AAGGCTGACTTTGCAGAATGGAAGAGAAAAAGGTCTTGTTGCTGCTGCTTTTGGTAGCG
GCTCTAGTGTCACAGGGTTGCTGT**CAACATTGGTCCTATGTCTTGAACCCAGGG**GGGAA
AAGAGTGACTGACAGCCTGTCTGACACCCTGGACAAT**GT**AAGTACTTTACCTATTCATA
ATATGGAAGCAGAAGTAGCTAGGTCTTTTACTAAAGGTTGTGATGGTAAAGTATCCAAT
GATGGAAGGGATGTTCCTATATTTGCATTGTAAAATCTAGTTAAAATGACAATTCTA**CA**
GATGGCTGAAGACCTTCCGAAGATAGACACATCTTGCAGTTTGTTTGGCTGTGCTGATG
TCTCACCTCATCCTCTTACTT**GT**GAGTGACATTTACAATTTTGAATATACATTTCCCTT
CAAGAAGCTACCCAGAAGTTGAATATAAAGGGACATTGTGTTGGTAGTTAAATATTGCA
ACTTTTTTAATGTATGTTTTTTTTCTCTCAATGTTTTAAACAATTTGTATCCAAATTGT
AAGTCATGTTTTTAATTTGCTTTTTTCT**AG**GCAAACCTCGCTGACAGACAAAGTGGACT
CAATAATATATAGCAAATGTATGCTAGCTAACTCT**ATTAAA**CATTGCGGTGCCATCATT
GTGTTTTGTGGTGTCTTTGTTGGGTGACAATTTTTGCTAGATCGCATATTACATTGTAT
GGCTTTAAAGCAGTGTAACACGATGTCAAAATACAATGACGGGTAGGCAATCATTGT**AA**
ATAAGAATTTGTTCTTAACTGACTTGCCTAGTTAAAACAAAACAAAAAACTAAATGCCA
ACAAATGATTGCCAGTATGAGTTACGTTGTTGACTCTTGTAGCAAACCTTTATTTTGAA
AATGTTAACAGCATGGCTTTAATGTCGAGTTCCTGTGTACACATGTTCATCACGGGTTA

mRNA:

ACCGAAAGTAATAAGGCTGACTTTGCAGAATGGAAGAGAAAAAGGTCTTGTTGCTGCTG
CTTTTGGTAGCGGCTCTAGTGTCACAGGGTTGCTGT**CAACATTGGTCCTATGTCTTGAA**
CCCAGGGGGGAAAAGAGTGACTGACAGCCTGTCTGACACCCTGGACAATATGGCTGAAG
ACCTTCCGAAGATAGACACATCTTGCAGTTTGTTTGGCTGTGCTGATGTCTCACCTCAT
CCTCTTACTTGCAAACCTCGCTGACAGACAAAGTGGACTCAATAATATATAGCAAATGT
ATGCTAGCTAACTCT**ATTAAA**CATTGCGGTGCCATCAT

protein:

MEEKKVLLLLLLVAALVSQGCC**QHWSYVLNPG**GKRVTDSLSDTLDNMAEDLPKIDTSCS
LFGCADVSPHPLTCKPR

Omyk_1.0

Organism name: Oncorhynchus mykiss (rainbow trout)

>gi|1207596189|ref|NC_035087.1|:52759250-52760928
Oncorhynchus mykiss isolate Swanson chromosome 11,
Omyk_1.0, whole genome shotgun sequence

TCTAGATGTATACATACTTTCTTGGTTATTTAACGCAACATCTCTGAAAGTCAGATTAT
TAAACTATAAGTGCATGAAGGTTTAAAGTAACAATATAAGGGCATGGTGTGACTGTACA
TTCTTATCAATGTACTTAGTTTTATGTCAGATAGGCTTTATGATTAGGATAATGTTTTT
TCTACGCCGAAGCCATACTGTAGCATGATAAAAATGGCAAAAGAATTTGCTGTTATTGC
ACCACCTAATTATGCTGTGTATTTCATTTTTCTTATAATAATGTGGCTTTTGAAACATA
ATTTCAAGAATGCAATGTAATCTTCATTGATAATCACTGAAGTCAGAAGCTGCCTGTTG
TTTTGTTTGGTGTTCTCTGTGAATCTACCTCCTTATTTTTTTTTACCACTTTCTGATTT
ATATCTAGTTCCATGCATTTCATGGTGGTTTTTGTCTCTTGTTGTATGAAAACCATTAT
TCATAATATCCAAATATGATAAGTAAATAATCAAAGTGAATAAGCTGTTTTTTAATAGT
GTTACTGTCATTTTCCAGCTTGTGGATCACCTAGTTGTATGAGGAAGATGGTTGTATAA
CAACACACCCATCACAATGAATTGCATATAATTCAATCATGTTTTGAAGTTTGCACAAA
TCTGTCTTAAATTAAACATTTTGTTCACTAACTTCTGTTTTACTTACTCACAGGACACC
AAAAAGTTGATTGTGGTGCTTGTAGTGTTTTTTGAAATATATTTTTTTTATCCAGTGCT
TACAAAACTTGTGTGTTTTATAAGGATAGCAAAACATCACTGCTGGACTGTGATTGTTA
AAATCTGTCAGTTTGTTTAAGAAAATGTTTGCATGTTTAAAGACTGTATTTTACCAGTC
TAAGGCTGCTGAATTTTTTTCTAGGTGTAGCTGAAACCTTTTTTGAGATGCAATATTCT
CTCTCTATACTCTATTCCATAGTAGGTACTCTTTTGTCGTGGTAATGAAGGCAGTCATA
AAGAATATTGCACCTTGAGTGTGGAGATCGTCAGTATCAGGACCTTGTAGAAAAGAACA
ATGGTTATTTATTTTGATATAACATATCGAAGAAATATGGAATGAAAGTTAGTTAAAGA
CTGGGGGTGTTTCTCTTCCTCCCCTGTCTATAATTATTGCACTCAAGCACAAAAATGTA
TTTTAATAAATTACTGTTATCAATAAACAAAAGCCACTGTGTAATTAAAAATGAAATAC
TGTTTTCACTAACTTTCCAGAAGAACACCAACACCATGTGCCTAATTAATTTTATTGTT
GCTGCTGTAAAAGTGCACAATCATTCCCAAAGTGTTAATCTTTTAAAAGGCTATTAGGG
GTGCCCGGTGCCTGGAGTGGAGGTGCTTAAAGCTGCTCTGTAATATATTTTTGAGTGGA
GCCTTTTTCTGTAATCCACTTTGTGTCCTCCTGCAGCAGTGATGGAACTGGTGGGTATG
GCATATGGGAACTAAGCTGCCTATAGAACCTCAGACACCTGAGTTGCTT**ACCTAAAGTA**
ATAAGGCTTACTTTGCAGAATGGAAGAGAAAAAGTTATTGTTGCTGCTGCTTTTGGTCG
CGCCTCTAGTGTCACAGGGTTGTTGT**CAACATTGGTCCTATGGCTTGAACCCAGGG**GGG
GAAAGAGTTACTGACAGCCTGTCTGACACCCTGGACAAT**GT**AAGTACTTTACCTATTCA
TAATATGGAAGCAAAAGTAGCTAGGTCTTTTCCTAAAGGTTGTGATGGTAAAGTATCCA
ATGATGGCAGGGATGTTCCTATAGTTGCATTGTAAAATCTAGTTAAAATGACAATTCTA
CAGCTGGCTGAAGACCTTCGAAGATAGACACATCTTGCAGTTTGTTTGGCTGTGCTGAT
GTCTCACCTCATCCCGAAATTTACCGGCTGAGGGTATTACTT**GT**GAGTGACATTTACAA
TTTTGAATATACATTTCCCTTCAAGAAGCTACCCAGAAGTTGAATATAAAGGGAGGTTG
TTTTGGTAGTTAAATATTGCAATTTGTTATGTATGTTTTTTTTCTCAATGTTTTAAACT
ATTTGTATCCAAATTGTAAGTAATGTTTTTCATGTACTTTTTTCT**AG**GTAAGCCTCGCT

GACAAAGTGGACTCAATAATATATAGCAAATGTATGCTAGCTAACTCT**ATTAAA**CATTG
CCGTGCCATCATTGTGTTTCGTGGTGTCTTTTGTGGGACAATTTTTGCTAGATCGCATA
TTACAGGGTATGGCTTTAAAACAGTGTAACACGATGTCAAAATACAATGACGGGTAGGC
CATCGT**AAATAA**GAATTTGTTCTTAATTAATTGACTTGCCTAGTTAAAACTAAATTCAA
ACAAATGATTGCCAGTATGAGTTACGTTGTCTACTCTTGTAGCGAACCTTTATTTTGAA
AATGTTGACAGCATGGCTTTAATGTCGAGTTCCTGTGTACACGTGTTCATCACAGGTTA
GTTAAACAAGTATTTGTATACATTCTGTGGTGGTGGTTCAAGATTATTAGCTTTGAGTC
CAGAATGTCCAGATAAATAGCTCAACTCAAGCTCGGTTACTTTAGGGACACGTTTGCAG

There is a C missing in exon 2 of the genomic sequence that we have added to the coding region presented below (in bold).

mRNA:

ACCTAAAGTAATAAGGCTTACTTTGCAGAATGGAAGAGAAAAAGTTATTGTTGCTGCTG
CTTTTGGTCGCGCCTCTAGTGTCACAGGGTTGTTGT**CAACATTGGTCCTATGGCTTGAA**
CCCAGGGGGGGAAAGAGTTACTGACAGCCTGTCTGACACCCTGGACAATCTGGCTGAAG
ACCTTC**C**GAAGATAGACACATCTTGCAGTTTGTTTGGCTGTGCTGATGTCTCACCTCAT
CCCGAAATTTACCGGCTGAGGGTATTACTTGTAAGCCTCGCTGACAAAGTGGACTCAAT
AATATATAGCAAATGTATGCTAGCTAACTCT**ATTAAA**CATTGCCGTGCCATCAT

protein:

MEEKKLLLLLLLVAPLVSQGCC**QHWSYGLNPG**GERVTDSLSDTLDNLAEDLPKIDTSCS
LFGCADVSPHPEIYRLRVLLVSLADKVDSIIYSKCMLANSIKHCRAIIKKKKK

Oner_1.0

Organism name: Oncorhynchus nerka (sockeye salmon)

>gi|1681300725|ref|NC_042547.1|:49325254-49326964
Oncorhynchus nerka isolate On170113-E2 linkage group LG13, Oner_1.0, whole genome shotgun sequence

ACTTTCTTGATTATTTAACGCAACATCTCTGAAAGTCAGATTATTAAACTATAAGTGCA
TGAAGGTTTAAGGTAACAATATAAGGGCATGGTTTGACTGTACATTCTTATCAATGTAC
TTAGTTTTATGTCAGAAAGGCTTTATGATTAGGATAATGTTTTTTCTACGCCAAAGCCA
TACTGTAGCATGATAAAAATGGCAAAAGAATTTGCTGTTATTGCACCACCTAATTATGC
TGTGGATTTCATTTTTCTTATAATAATGTGGCTGATGAAACAGAATTTCAAGAATGCAA
TGTACTCTTCATTGATAATCACTGAAGTCAGAAGCTGCCTGTTGTTTTGTTTGGTGTTC
TCTGTGAATCTACCTCGTTTTTTTTTTACCACTTTCTGATTTATATCTAGTTCCATGCA
TTTCATGGTGGTTTTTGTCTCTTGTTTTATGAAAACCATTATTCATCATATCCAAATAT
GATAAGCAAATAATCAAAGTGAATAAGCTGTTTTTTAATAGTGTTTACTGTCATTTTCA
AGCTTGTGGATTACCTAGTTGTATGAGGAAGATGGTTGTATAACAACACTCCCATCACA
ATGAATTGCATATAATTCAATCATGTTTTGAAGTTTGCACAAATCTGTCTTAAATTAAA
CATTTTGTTCACTAACTTCTGTTTTACTTACTCGCAGGACACCAAAAAGTTGAATGTGG

TGCTTGTAATGTTTTTTATTTTATTTTTTAAATCCAGTGCTTACAAAACTTGTGTGTTT
TATAAGGATAGCAAAATATCACTGCTGGACTGTGATTGTTAAAATCTGTCAGTTTGTTT
AAGAAAATGTTTGCATGTTTAAAGACTGTATTTTACCAGTCTAAGGCTGCTGAATTTTT
TTCTAGGTGTAGCTGAAACCTTTTTGAGATGCAATATTCTCTCTCTATACTCTATTCCA
TAGTAGGTACTCTTTTGTCGTGATAATGAAGGCAATCATAAAGAATATTGCACCTTGCG
TGTGGAGATCGTCAGTATCAGGACCTTGTAGAAAATAACAATGGTTATTTATTTTGATA
TAACATATCGACGAAATATGGAATGAAAGTTAGTTAAAGACTGGGGGTGTTTCTCTTCC
TCCCCTGTCTATAATTATTGCACTCAAGCACAAAAATGTATTTCAATAAATTACTGTTA
TCAATAAACAAATGCCACTGTGTAATTAAAAATGAAATACTGTTTTCACTAACTTTCCA
GAAGAACACCAAGACCATGTGGCTAATTAATTGTATCGTTGCTGCTGTAAAAGTGCACA
ATCATTCCCAAAGTGTTAATCTTTTAAAAGGCTATTAGGGGTGGAGGTGCTTAAAGCTG
CTCTGTAATATATTTTTGAGTGGAGCCTTTTTCTGTAATCCACTTTGTCCTCCTGCAGC
AGTGATGGAACTGGTGGGTATGGCATATGGGAACTAAGCTGCCTATAAAACCTCAGACA
CCTGAGTTGCTT**ACCTAAAGTAATAAGGCTTACTTTGCAGAATG**GAAGAGAAAAAGGTC
TTGTTGCTGCTGCTTTTGGTAGCGCCTCTAGTGTCACAGGGTTGCTGT**CAACATTGGTT**
CTATGGCTTGAACCCAGGGGGGAAAAGAGTTACTGACAGCCTGTCTGACACCCTGGACA
AT**GT**AAGTACTTTACCTATTCATAATATGGAAGCAAAAGTAGCTAGGTCTTTTCCTAAA
GGTTGTGATGGTAAAGTATCCAATGATGGCAGGGATGTTCCTATAGTTGCATTGTAAAA
TCTAGTTAAAATGACAATTCTA**CAG**CTGGCTGAAGACCTTCCGAAGATAGACACATCTT
GCAGTTTGTTTGGCTGTGCTGATGTCTCACCTCATCCCGAAATGTACTGGCTGAGGGCA
TTACTT**GT**GAGTGACATTTACCATTTTTAAAATACATTTCCCTTCAAGAAGCTACCCAG
AAGTTGAATATAAAGGGAGGTTGTTTTGGTAGTTAAATATTGAAACTTTTTATGTGTTT
TTTTTTTCTCTCAATGTTTTAAACAATTTGTATCCAAATTGTAAGTAATGTTTTTCATT
TACTTTTTTCT**AG**GTAAGCCTCGCTGACAGACAAAGTGGACTCAATAATATATATCAAC
TGTATGCTAGCTAACTCT**ATTAAA**CATTGCCGTGCCATCATTGTGTTTTGTGGTGTCTG
TTGGGGGACAATTTTTGCAAGATCGCATATTACAGGGTATGGCTTTAAAACAGTGTAAC
ACGATGTCAAAATACAATGACGGGTAGGCCATCAT**AAATAA**GAATTTTTGATTAATTAA
CTGACTTGCCTAGTTAACTAAATTCCAACAAATGATTGCCAGTATGAGTTACGTTGTCT
ACTCTTGTAGCAAACCTTTATTTTGAAAATGTTTACAGCATGGCTGATGTCGAGTTCCT
GTGTACACGTGTTCATCACGGGTTAGTTACACAAGTATTTGTATACATTCTGTGATGGT
GGTTCAAGATTATTAGCTTTGAGTCCAGATAAATAGCTAAACTCAAGCTCGGTTACTTT
AGGGACACGTTTGCAGACAATCGTCCGGTTTTCGCTAGCATCT

mRNA:

ACCTAAAGTAATAAGGCTTACTTTGCAGAATGGAAGAGAAAAAGGTCTTGTTGCTGCTG
CTTTTGGTAGCGCCTCTAGTGTCACAGGGTTGCTGT**CAACATTGGTTCTATGGCTTGAA**
CCCAGGGGGGAAAAGAGTTACTGACAGCCTGTCTGACACCCTGGACAATCTGGCTGAAG
ACCTTCCGAAGATAGACACATCTTGCAGTTTGTTTGGCTGTGCTGATGTCTCACCTCAT
CCCGAAATGTACTGGCTGAGGGCATTACTTGTAAGCCTCGCTGACAGACAAAGTGGACT
CAATAATATATATCAACTGTATGCTAGCTAACTCT**ATTAAA**CATTGCCGTGCCATCAT

protein:

MEEKKVLLLLLLVAPLVSQGCC**QHWFYGLNPG**GKRVTDSLSDTLDNLAEDLPKIDTSCS
LFGCADVSPHPEMYWLRALLVSLADRQSGLNNIYQLYAS

Nonviable GnRH1 Homeologs

Organism name: Thymallus thymallus (grayling)

>gi|1594667059|gb|CM014996.1|:9213248-9215800 Thymallus thymallus isolate TTM2012 chromosome 13A, whole genome shotgun sequence

GCATAAATGGGAGGTGGCATGGTGTGACTACATTCTTATCAATGTACTTAGTTTAATTG
CCTGATATGCTTTGTGATTAGGATCATGTCAAAGCCATACTGTAGCATGGTGAAAATGG
CACCACACAATTATTCTGTGGATTTCATTTTTCCTAAGACTTTGTGTTGAACTGTATTC
AACAGAATTTCAAGTATGCAATGTACTCTTTTTCTTTGCTACCTGTCGTTTTTGGTGTT
CTCTGTGAATCTACCTCCTTATTTTCTACCCCTGTCTGGTTGATCTCTAGTCTAGTTGC
ATTTCACTGGTTTTTGCCTCTTGTTGTATGACATCCATTATTCATCATATCCAAATATG
TTAAGAAATTATCAAAGTGGATGTGTGTTTTAATTTTGATACTGTCTTAAAGCTTGTGC
AGCACCTAGTTGTAAGAGGAAGATAGTTGTATGACAACACTCCCCTCAAGGAGACAACA
ACCAATGAACAGGGCATATCATTCACTCATTATTTTGCACAAATATGTCTAAAAAGTAT
GTGTCTGTCACTAACTTCTGTTTTACTCACTCATAGGACACCAAAAAGTTAAATGGTTA
CATAATGTATTTCAAATCCAATGCTTCCAAATCGTGTAAGTTATTAGGATAGCAAAATG
TTATCACTGCTGGACTGTGATTTTCACAATCTCACATCAGTTCTTTTAGAACAATGTCT
GCATGTTTAAAGACTGTATTTTCTCTGACTGTAGCTGAAAGATTTTGAGATGCAATATT
TTCTCTATACTCTTTCACAGTAGGTATGTTGTGATAATGAAGGCAATCGTAAATAATAT
TGCACCTTGCGTGTGGAGATCGTCAATATCAGAACTTTGTAAGAAAGAGCAAGGGTTAT
TTATCTTAATATAACATATTGTATTCAAGAAATATAGAATGAAACTTAAGTTCTTTAAA
GACGGTGTCTCTTCTTCCCCTGTCTATAATTATTGCACTCAAACACAAAAATGTAATTC
AATAAATTACTTTTATGAATTTTAAAAAAGGGTCTGTAATTACTTGTGCAAATGAAATA
CTGTTGTTTATTTTTACTAACTTTCCAAAATAACTCTTAAGACCCTGTGCCTAATTAAT
TTAATTGTTGCTGCTGTAAAAGTGCACAAACATAGCCAAAGATTTTTAGGCTTTTACAA
TACATTTGGGGTGCCTGAAGTGGAGGTGCTTAGTGCTGCTCTGCAATATATTTTTGAGT
GGATCCTTTTTTCTTTAATCCCACTTTTTTGTGACCTGCAGCGAGGTGATGGGACTAAG
GGGCCATATGGGAACCAAGCTGCCAATAAAACCTCAGACACATTAACTGCTCAGACAGA
GTAAGGCTGACTTTGTAGAATGGAAGAGAAAAAATATCCTGTTGTTGCTGCTGCTTTTGG
TGGTGGAACAAGTGTCACAGGGTTGCTGTCAACCTTGAACTAGTGTCACAGGGTTGCTG
TCAACATTGAACTAGTGTCACAGGGTTGCTGTCAACATGGAACTAGTGTCACAGGGTTG
CTGTCAACATTGAACTAGTGTCACAGGGTTGCTGTCAACATTGAACTAGTGTCACAGGG
TTGCTGTCAACATAGAACTAGTGTCACAGGGTTGCTGTCAACATTGAACTAGTGTCACA
GGGTTGCTGTCAACATTGAACTAGTGTCACAGGGTTGCTGTCAAC**ATG**GAACTAGTGTC
AAAGGGTTTCTGTCAACATTGAACTAGTGTCACAGGGTTGCTGTCAACATTGAACTAGT
GTCACAGGGTTGCTGT**CAACATTGGTCCTATGAGCCCAAGGG**GGAAGAGAGAGACTGGC
AACCTGTCTGACACTGTGGGAAAT**GT**AAGTACTTTACCTATTCATAATGTGGAAGTAGG
TATATTTACTTAAGGTTGTGATGGTGCTTTTCAAATGATGGGAGGGATGTTCCTATATT
TGCATAGTAAAATCTAGCTAAATTACAATTCTA**CAG**ATGGCTGAAAACCTACTGAAGAT
GGACACATCCTGCAGTTTGTTTGGCTGTGATGTCTCACGTCATGCCAAAATGTACAGGC
TGAGGGTATTT**GT**GAGTGACGTTTACAATTTTGCCAATAGAGCAGTGTCTCTTCAAGAA
GCTACCCAGAAGTTGAAAATATGGAAATTGTGTTGGTAGTTACTTTTTTCATTGATGTG
TTTTTTCTCAATGTTTTTTATCCAAATTGTAAACCCTATTTTCTATTTAGTTTTTTTG**T**
AGGCAACCCTCGCTGACAGAGCGTGGATTCAAAAATATATAGCAAATGTATTGTAACTC
TAATAAACATTACAGTGCCATCACTGTGTTTTGTGGTGATCTTTGTTGGGGGTGCTGTT

TTGGTGGGTTGCATATTACAGAGTGTGGCTTTAAAATATTGGAACACACCCATTCAAAG
TTCTATAGAACACAATTTCTCCGCCATCAACCTGTATAGTATAGTTAACCATACAAATA
CTCCTTATTTATCTATATCAACTTGCAATATTTTAATCTTTTTTATTTTTTTCACCTGA
CACTGTAACAATGTATAGCCATCCACCCATACTATGCAATGTCTGTCTGTCTATCTATC
TATCTCAAGTGGTTAGCCACTTGGTGTCCTGTAACTGAAAGGTTGCAGGATCAAATCCC
CGAGCTGACAAGGTAAAAATCCCCTGAACAAGGCAGTTAACCCACTGATCCTAGGCTGT
CATTGTAAATAAGAATTAGTTCTTAACTGACTTGCCTAGTAAAATAAAGGTACAAAGTA
AATGCTATCTATATAAGAGATTGGTGATGCAGTATGTAATAATGATTCACTGACTAAGT
GTCACGATGTGGGTGTGGGAATGACCAAGGCGCAGCGCAGGGATAGTAGTTCGACATAT
TTATTATACTATGTAAGAATGTATATTACTATGTATAACAAGACAAAAGACGAACTACT
GAAAACACAAAAAACACGAATTGATAAAACGTCACGCTCACAAAACAAAACACGAATGA
ACAGACGTAAATCACGCACACTTACTTCTCACCGTGACATGCGAAATATACGACAACGA
CCCACACCAGAGTGAAGGACAGACTCAACATATATAGGATCCCTAATTAGAGACAATGC
GGTGCAGCTGCCTCTAATTAGGGATCCAACCCAAACCCCACACAGACACAGAAAAGAAC
GATACTGTCAGACTCTGACCATAGCTACATAGAGAAGCAAATAGGCTTCTCTATGGCCA
GAGCCTGACACTAAGATACCGAAGAATAGTGTACAGTTTAAACATATGAGATGTG

It seems that an insertion of a repetitive element has occurred in the promoter and exon 1 region of the grayling GnRH1 ohnolog. Due to the insertion, a region containing remnants of the TATA box has been shifted over 300 bp upstream from exon 1, completely disrupting the orientation of the proximal promoter of the nonviable gene.

There is one in-frame stop codon in the region that would encode the signal peptide (underlined) and the in-frame sequence does not encode a functional GnRH decapeptide. The vestiges of sequence that once possibly encoded GnRH are highlighted in bold. If this gene was somehow transcribed and exon 1 translated (despite no obvious in-frame ATG start codon), the amino acid composition would be QHWSYEPKGEERDWQPV and then terminate. Exons 2 and 3 (alone, not in-frame with exon 1) theoretically could encode part of the GAP but would contain many amino acid substitutions compared to the viable GnRH1 protein (see below). The end of the third exon is roughly underlined.

>exons 2 and 3

ATGGCTGAAAACCTACTGAAGATGGACACATCCTGCAGTTTGTTTGGCTGTGATGTCTC
ACGTCATGCCAAAATGTACAGGCTGAGGGTATTTGCAACCCTCGCTGACAGAGCGTGGA
TTCAAAAATATATAGCAAATGTATTGTAACTCTAATAAACATTACAGTGCCATCACTGT
GTTTTGTGGTGATCTTTGTTGGGGGTGCTGTTTTGGTGGGTTGCATATTACAGAGTGTG
GCTTTAAAATATTGGAACACACCCATTCAAAGTTCTATA

MAENLLKMDTSCSLFGCDVSRHAKMYRLRVFATLADRAWIQKYIANVL

Organism name: Coregonus sp. 'balchen' (whitefish)

>gi|1711366460|emb|LR664359.1|:27878420-27882670 Coregonus sp. 'balchen' genome assembly, chromosome: 16

GGGTTTACCCAAATCAGTCTTTTTTGTGTGACAATAACTCTGTTTTCCTTCATTAGGTT
GTAGCTGTGTATGGGACCTTGGCTGACCTGTTGTCTGTAGCAAGCAATAAATTGGGAAT
CAAAGCCTGTAATTTATACAATGGAAAAGGTGGTCTTATAGATGACATAGCACTCATCA
GGTAGGGTACATTTCCTTCAACACTTTGTAGTTTCATGTACTTGGAATGTCCTTACTCA
GGCCAATATTACATATTCATTTCTCTACTATTCCTTATTATAGGGATGATGATGTTTTG
TATGTCTCAGAGGGAGAACCCTTTGTTGGTGAGTTTGTGTCCTCTAAATCTTTCCTAGT
CTTTGCAAACCATTTGGTTCTGAAGAATGAGGTGGTGAGTTATCCCTCTGAATAATGTT
ATTATCTTAGACCCACAGAATGATGTCAGGGCTACAGACGGCCTGCCTAGGGCACACAC
AGATTGGCTGACCCTTAATATTGGGGGGCGTCTCTTCACCACCACACGGTAAGCAATGG
AGGAGTTGTTTCTAATGTCTGTGAACATCAAAAGTCACATAGGAATATCTCTGTTAGTA
TTATTGCCATCCACTTTATTATTTGGTCACGTTTTTCCTCAAAATTATGGTCCTTTTTA
CAGGAGCACCTTGGTCAGCAAGGAGCCAGAGAGCATGCTTGCTCATATGTTCCGGGAGA
AGGGTAAACAGTAATTAAGTTTGTCACATCTAACACTGCTGACCTTATACAAGATACCT
TTTCTGTTGTAATTTACCCATAATGATGGCCTCTCTTTGCTCTGCAGATGTATGGGGTA
ACAAGCAGGATGAACGTGGGGCTTACCTGATTGACCGCAGCCCAGAATACTTCGAGCCT
ATTCTTAATTACCTGCGACATGGCCAGCTCATTATCAATGAAGGAATCAATTTACTTGG
TATGTATGGCATTTACAGGCGGCGCATTATCTTTATTCTACAATAAAGCTCACAGATTA
ATGATATCACTTCAGTTGTACATGTTGACTACAGTATTTATATTTCTGAAATCTTGACA
ACATATTGGCTTTAATCAGTAAATCTAATCTGGGTTTCAGGGGTTTTGGAGGAGGCTAA
ATTCTTTGGAATTGAGCAGCTGGCTGAGCAGTTGGAAGTAGCAATAAAGGTGAAGAACT
CATGGCCTTATGGATGACAAGCAAGCTCCATATTTCAAATGATTTATTTAATTTCGTCA
CTGGATATTAATCCATGTCACCACATGCTTTTCTCCAGAATAACCAGCCACCTGAGGAC
CACTCTCCCATCTCTCGCAAGGAGTTTGTTCGGTTTCTGCTGGCTACACCCACCAAATC
AGAGCTCCGCTGTCAGGTAAAACAACACATTTTCTACAAGGCCAACACCCTGTTGAACA
TTAGATACAGTTTACCTAACATATGTGAACGTGATAAATAATACCTCTCTCCATCCAAA
AGGGACTTAATTTCAGTGGTGCTGATCTCTCTCGCCTTGACTTGCGCTACATCAACTTC
AAGATGGCCAACCTGAGTCGCTGCAACCTGACACATGCCAACCTATGCTGTTCAAACCT
GGAGCGGGCTGACCTCTCTGGGGCCAACCTCGATGTAAATCCATTAGCTCCTTCATTAG
CTTATGAAACCTACCTGTTACAGCGTGTCAACATTTGAGAAATTAGCAGTATTTTGAGG
GGACTGATTTTGTCTTTCTGTTTTAGGGTGCTAACTTGCAGGGTGTAAAAATGCTCTGT
TCAAATGCTGAGGGGGCGTCTCTCAAAGGATGCAATTTTGAGGACCCGTCTGGACTAAA
GGCCAACTTGGAGGGTAAACTGTAATTTATTTGTATTATAAGAGGGATGAGAAATATGG
AACTGGAAACACTTGTATAACCTCTGGTGTTGAACATCTAATGTCTGTCTCATTAGGTG
CCAATCTGAAAGGGGTCGACATGGAGGGAAGTCAGATGACTGGAATTAATCTGCGTGTG
TCCACTCTAAAAAATGCTAAACTGAAGAACTGTAACCTACGGGGTGCCACTTTGGCAGG
AACTGATCTTGAGGTAAGCAGAGGGCATTTAAGAGGATTAACCTTGAGACAAAAATATC
AAGTAAATCTGAAACAAATGAGATCAGACAGCTCTTGTAATCATTTTGTTTGTTGTTGG
AGGTTTATCAGATACCATAAAGTTGCATCCATCCTAAGATAACTGTTTTATTACTGTGA
CATCTAAGGTTATTGTCAAGAATACTGACTGGTTAGTACCAAGAATACTGACAGTTTTG
TTTTACTCCTCACAGAATTGTGATCTGTCTGGCTGTGACTTACAAGAGGCCAACTTGAG
AGGGTCCAATGTGAAGGGGGCCATTTTTGAAGAAATGCTGACTGCATTGCACATGTCTC
AGAGTGTCAGATGACTCTCCTGCACAACCCTGCACATTCCACCTGGATTCAGGCTGGTT
AATCTCATGGATGCCTGGCAACGATGTTATTATGCCAGCAAATCCCACCCACCCCTACT

ATCAAGCTTTCTGGACCCTTATGCAATACATACATTTTTATGCACATCCCACATCCCTT
CCACCTGATATACTAGCTTTAAATCTCTTGCACTAGAGCACATCCAGGGTTATCCATTT
ATGTTATGTAGTGGAAGGTTTTAAATGTCTGACTGTCTTTCCTTATTTTTTTCCCAAAA
CCAAAATATTGAATGTTCTTTATGTATGTTTACTATTACGTTTTTTTTTTTTTATTATT
TCAAGATGTGTGTATGTATATATATTTCTTGATTATTTAACACGGTTCTGTAAGTCAAA
GATTATCAAACTATAAGTCCATGAAAGTTGAAAGTAAAAATGTAAGGGCCTCAACCAAG
ACTGTGGTATAGCATAAGTGGGAGGTGGCATGGTGTGACTGTACATTCTTATCAATGTA
CTTAGTTTAATTGCCAGATATGCTTTATGTTTAGGATGTTTATTTTCTAAGTCAAAGCC
ATACCGTAGCATGGTGAAAATGGCATAATAATTTGCTGTTATTGCACCACACAATTATG
CTGTGGATTTCATTTTTCATATAAGACTGTGTGTGTTGAACTGTATTCAACAGAATTTC
AAGTATGCAATGTACTCTTTCCTTGCTGCCTGTTTTTGGTGTTCTCTGTGAATCTACCT
CCTTATTTTTACCACTGTCTGATTGATCTCTAGTCTAGTTGCATGCATTTCACTGTGGT
TTTTGCCTCTTGTTGTATGAAATCCATTATTCATCATATCCAAATATGATAAGCAATTT
ATCGAAGTGAATGTGTGTTTTAATTGTGTTACTGTCATGTTCAAGCTTGTGTAGCACCT
AGTTGTAAGAGGAAGATAGTTGTATAACAACACTCCCGTCAAGGAGACAACAACCAATG
AACAGGGCATTTCATTCAGTCATGTATTGACATTTTGCTTTGCAACTTCTGTTTTACTC
ACTCATAGGACACCAAAAAGTTAAATGTGGTGGTTGTAACAATGTATTTCAAATCCAGT
GCTTCCAAAACGTGTAAGTTATTAGGATAGCAAAATGTTATCACTGCTGGACTGTGATT
GTCACAATCTTACATCAGTTATTTTAGAACGATGTCTGCATGTTTAAAGACTGCATTTT
TTCTGGCTGTAGCTGAAAGATTTTGAGATGCAATATTCTCTCTATACTCTATCACAGTA
GGTAGGTACTCATCTTTCGCGATAATGAAGGCAATCATAAAGAATATTGCACCTTGAGT
GTGGAGATCGTCAATATCTGAACCTTGTAAGAAAGAACAATGGTTATTTATTTTGATAT
AACATATTGTATTAAAGAAATATGGAATGAAAGTTAGTTCTTTAAAGACCGGGTGTGTT
TCTCGGCTTTTGCTGTCTATAATTATTGCACTCAACCACAAAAATGTAATTCAATAAAT
TACTGTTATGAATTAAAAAATAAATTACTTGTGCAAATGAAATACTGGTATGTTTTCGC
TAACTTTCCAACTAATAACTCTTAAGACCCTGTGCCTAATTCATATAATAGTTGCTGCT
GTAAAAGTGCACAATCATACCCAAAGTATTTTAGGCTTTTAAAAGGCTCTTTGGGGTGC
CTGAAGTGGAGGTGCTTAGTGCTGCTCTGCAAAATATTTTTGAGTGGATTATTTTTTCT
GTAATCCCACTTTTTTGTGACCTGCAGCGAGGTGATGGGACTAAGGGGCCACATGGAAA
CCAAGCTTCCAATAAAACCTCAGAGACAAATGAACTGCTCAGATAGAGTAAGGCTGACT
ATTGGTAGA**ATG**GAAGAGAACAATGTCCTGTTGTTGCTGCTGCTCTTGGTGGTGGTACT
AGTGTCACAGGGTTGCTGT**CAACATTGGTACCATGACATGAGCCCGTCA**ACATTGGTCC
TATGACATGAGACCAGGGGAGAAGAGAGAGACTGGCAGCCTGTCTGACACTGTGGGAAA
T**GT**AAGTACACTGCTCAAAAAAATAAAGGGAACACTTAAACAACACAATGTAACTCCAA
GTCAATCACACTTCTGTGAAATCAAACTGTCCACTTAGGAAGCAACACTGATTGACAAT
ACATTTCACATGCTGTTGTGCAAAAGACACCCCCAATAAAGGACTGGTTTTGCATGTGG
TGACCACAGACCACTTCTCAGTTCCTATGCTTCCTGGCTGATGTTTTGGTCACTTTTGA
ATGCTGGCGGTGCTTTCACTCTAGTGGTAGCATGAGACGGAGTCTATAACCCACACAAG
TGGCTCAGGTAGTGCAGCTCATCCAGGATGGCACATCAATGCGAGCTGTGGCAAGAAGG
TTTGCTGTGTCTGTCAGCGTAGTGTCCAGAGCATGGAGGCGCTACCAGGAGACAGGCCA
GTACATCAGGAGACGTGGAGGAGGCCGTAGGAGGGCAACAACCCAGCAGCAGGACTGCT
ACCTCCGCCTTTGTGCAAGGAGGAGCAGGAGGAGCACTGCCAGAGCCCTGCAAAATGAC
CTCCAGCAGGCCACAAATGTGCATGTGTCTGCTCAAACGGTCAGAAACAGACTCCATGA
GGGTGGTTTGAGGGCCCGACGTCCACAGGTGGGGGTTGTGCTTACAGCCCAACACCGTG
CAGGACGTTTGGCATTTGCCAGAGAACACCAAGATTGACAAATTCACCACTGGCGCCCT
GTGCTCTTCACAGATGAAAGCAGGTTCATACTGAGCACGTGACAGACGTGACAGAGTCT
GGAGACGCTGTGGAGAATGTTCTGCTGCCTGCAACATCCTCCAGCATGACCGGTTTGGC
GGTGGGTCAGTCATGGTGTGGGGTGGCATTTCTCTGGGGGGCCGCACAGCCCTCCATGT

GCTCTCCAGAGGTAGCCTGACTGCCATTAGGTACCGAGATGAGATCCTCAGACCCCTTG
TGAGACCATATGCTGGTGCGGTTGGCCCTGGGTTCTCCTAATGCAAGACAATGCTAGAC
CTAATGTGGCTGGAGTGTGTCAGCAGTTCCTGCAAGAGGAAGGCATTGATGCTATGGAC
TGGCCCGCCCGTTCCCCAGACCTGAATCCAATTGAGCACATCTGGGACATCATGTCTCG
CTCCATGCACCAACGCCACGTTGCACCACAGACTGTCCAGGAGTTGGCGGATGCTTTAG
TCCAGGTCTGGGAGGAGATCCCTGAGGAGACCATCCGCCACCTCATCAGGAGCATGCCC
AGGCATTGTAGGGAGGTCATACAGGCCACACACACTACTGAGCCTCATTTTGACTTGTT
TTAAGGACATTTTACATTTTAGTCATTTAGCAGACACTCTTATCCAGAGCGACTTACAG
TTAGTGAATACATATTTTTTTATACTGGCCCCCCGTGGGAATCGAACCCACAACCCTGG
CGTTGCAAACGCCATGCTCTATCAACTGAGCTACATCCCTGCCCTACCCTGGACAACTG
TGCGCCGCCCATGAGTCTCCCGGCTGCGACAGAGCCTGGATTCGAACCAGGATCTCTAG
TGGCACAGTTAGCTTAGACCACTGCGCCACTCAGGAGTGGGGACATTACATCAAAGTTG
GATCAGCCTGTAGTGTGGTTTTCCACTTTAATTTTGAGGGTGACTCCAAATCCAGACCT
CCATGGGTTGATAAATTTGATTTCCATTGATAATTTTTGTGTGATTTTGTTGTCAGCAC
ATTCAACTATGTAAAGAAAAAAGTATTTAATAAGATTATTTCATTCATTTAGATCTAGG
ATGTGTTATTTTAGTGTTCCCTTTATTTATTTGAGCAGTGTACTTTACCCATTCATAAT
GTGGAAGTAGGTCTATTTACTAAAGGTTGTGATGGTGCTTATCCAATGATGGGAGGCAC
GTTCCTATATTTGCATCGTAAAATTTAGCTAAATGACAATTCTA**CAG**ATGGCTGAAGAC
CTTCTGAAGATGGACACATCCTGCAGTTTGCTTGGCTGTGATGTCTCACCTCATGCCAA
AATGTACAGGCTAAGGGCATTT**GT**GAGTGACATTTACAGTTTAGCAAATAGGGCAGTTT
CCCTTCAAGAAGCTACCCAGAAGTTGAAAATAAAAGGAAATTGTGATGGTAGTTACTTT
TTTCATTGATGTGTTTTTTCTCAATGTTTTATATCCAAATTGTAAACTATATTTTTTAT
TTAGTTTGTTTG**TAG**GCAACCCTCACTGACAGAGAAAGTGGACTAAACAATATATAGCA
AATGTATGCTAACTCTAATAAACATTGCAGTGCCATCACTGTGTTTTGTGGTGGTCTTT
GTTGGGGGCCCTGTTTTGGTCGTTTGCATATTACAGAGTCTGGCTTTAAAATAGTGGAA
CACACCCATTCAAACTTTTGTAAAGCACGATGTCAA**AATAAA**AGTGCCAACCAATGATT
GTGCTCAGCTGGTAGAGCACGGCGCTTGTAACGCCAAGGTAGTGGGTTCGATCCCCGCG
ACCACCCATACACAAAAAAAAAAAAAATGTATGCACGCATGACTGTAAGTCGCTTTGGAT
AAAAGCGTCTGCTAAATGGCATATTATTATTATTATTATTATTATTGTGAGTGTGAGTT
ACGTTGTCGGCTCTTGTAGCAAACCTTTATTTAGAAAATGTTGACAGAATGGCTTTTAT
GGCATTTGTTCATTTGGTTGTGTTTGTTAAAGATTATTATCTTCGAGTACAGAATTTCC
AAGTAATTAGCAAGCTAAATAGCTAAACTCAAGCAGTTACTTTGAGGAGCAAATTAACG
TTTGCAGACAAGCGTCCTGTTTTGCTAGCAGCTGTGTTAGTTATGGGCTAGCTGTAGCT
AACAACATTAGCTAGCTAGCTACACTATATATACAAAAGTATGTGGACACCCCTTCATA
TTAGTGGATTCGGCAATTACAGCCACTCCCATTACTGAGAGGTGTACAAAATCGAGCAT

It is unlikely that this GnRH1 pseudogene is transcribed since the proximal promoter has lost many of the features that are present in the viable gnrh1 promoter. For example, the two potential TATA boxes are missing in the *gnrh1* pseudogene. Nevertheless, the remnants of GnRH1-encoding sequences exist in the *Coregonus* sp. ‘balchen’ nonviable *gnrh1* ohnolog. Interestingly, sometime in the past, the GnRH1 decapeptide-encoding sequence was duplicated and then diverged. See text for further detail.

Organism name: Salmo salar (Atlantic salmon)

>gi|925216694|ref|NC_027323.1|:20445606-20447847 Salmo salar isolate Sally breed double haploid chromosome ssa24, ICSASG_v2, whole genome shotgun sequence

TTTTCTTAATTTTTTTCCCCCAAAACCAAAATATTGAATGTTCTTTATGTATGTTTACT
ATTATCTGGATTCAATCCTAACTTATTATTTTTATGATTTCTAGGTGTGTGTTTTCTTG
ATTATTTAATGTCTGTAAATCAAAGATTATCAAACTATAAGTGCATGAAAGTAAAAAAG
GGCCTCCATCAAGACTGTGGTAAAGTATAAATGGAGATGACATTGTGTGACTAAGTAC
ATTGATAAGAATGTACAAAGTGCCAGATATGCTTTATGATTAAGATGTTTATTTTTTAA
GTCAAAGCCATACCGTAGCATGGTGAAAATGGCAAAAATAATTTGCTGTTATTGTGCCA
CACAATTATGCTGTGGAATTCATTTTTCATAGACTGGGTGTTGAACTGTATTCAACAGA
ATTTCAAGTATGCAATGTACTCTTTTTCCTTGCTGCCAGTTGTTTTTGGTGTTCTCTGT
GAATCTACCTCCTTATTTTCTACCCCTGTCTGATATCTAGTCTAGTTGCATGCATTTCA
CAAGTTTTTGGCTCTTGTTGTATGAAATCCATTATTCATCATATCCAAATATAAGTAAA
TTATCAAAGTGAATGTGTTTTAATTGTTACTGTCATGTTCAAGCTTGTGTAGCACTTAG
TTGTATAACACTCCCCTCAAGGAGACAACAACCAATGAACAGGGCATATCGTTCAGTCA
TGTCTTGAAATTTTGCTTTGCACAAATGTCTAAATGGTATGTTTTGGTCAATAACTTCT
GTTTTACTCACTCATAGGACACCAACAAGTTAAATGTGCTTGTAACATAATGTATTTCA
AATCTAGTGCTTCCAAAATGTTATTAGGATATCAGAATGTTATCACTGCTGGACTGTGA
TTGTAACAATCTCACATCAGTTCTTTTAGAACAATGTCTGCATGTTTAAAGACTGCATT
TTCTTTGGCTGTAGCTGAAATATTTTGAGATGCCATATTCTCTCATACTCTATTTCATA
GTAGGTAGGTACTCATCTGTCATGATAATGAAGGCAATCATAAAGAATATTGCTCCTTG
AGTGTGGAGATCGTCAATATCAGAACCTTAACAATGGTTATTTATTTTGGTATAACATT
GTATTCAAGAAATATTGGATGAAAGTTCTTTAAATATCGGGTGTGTTTCTCTTCTTCCC
CTGTCTATAATTATTGCACTCAACCACAATGTAATTCAATAAATTACTGTGATGAATTG
TTTAAGTATCTGTGTAATTAATTACTTGTGCAAGTGAAATAATTATCTTTTCACTAACT
TTCCAAAATAACTCTATGCCCTGTGCCTAATTAATTTCATTGTTGCTGCTGTAAAATGC
ACAATCATACCCAAAGTATTTTAGGCTTTTAAAAGGCTCTTTGGGTTCCTGAAGTGGAG
ATGCTTAGTGCTGCTCTGCAATATATTTTTGAGTGGATGCTTTTTTTCTTTAATTCCAC
TTTTTTTGTGACCTCTGGTGAAGTGATGGGACTAGGGCCAATAAAACCTCAGACACATT
AACTGCTCAGATAGAGTAAGGTTGACTATTTGTAGAAAAGAAGACAAACGTCCTGTTGC
TGCTGCTCTTGGTGGTGGAACTACTGTCACAGGGTTGCTGT**CAACATTGGTCCTATGAC**
AGTCTGGCAGCCTGTCTGAAACCATGGGAAAT**GT**AAGTATTTTACCTATTCATAATGTA
GAAGTGGAAGTAGGTCTATTCACTAAAGGTTGTGATGGTGCTTATCCAATGATGGGAGG
GATGTTCCTATATTTGCATAGTAAGTTCTAGCTAAATGACAATTCTA**CAG**ATGGCTGAA
GATGGAGTTTGTTTGGCTGTGATGTCTCACCTCATGCCAAAATGTACAGGCTAAGGGAA
TTT**GT**GAGTGACATTTACAATTTTGCAAATAGGGTAGTTTCCCTTCAAGAAGCTACCCA
GAAGTTGAAAATAAATGGAAATTGTGTTGGTTGTTACTTTTTTCACTGATGTGTTTTTT
TTTCTCAAGGTTTTATCCAAATTGTAAACGATATTTTTAATTTAGTTTTTTG**TAG**GCAA
CCCTCGCTGACAGAAAGTGGACTCAAAAAATATATTGCAAATGTATGCTAACACTAAAAA
TTGCAGTGTCATCACTGTTTTGTGGTGGTCTTTTTTGGTGGTTTACATATTACGGAGTG
TGGCTTTAAAATAGTGGAAAAACACCCATTCAAACTTCTGTAGAGCACGATGTCAAAAC
AAAAGTGCCAACCAATGATTGCGAGTATGAGTTACGTTGACAACTCTTGTAGCAAACCT
TGATTTAGAAAATGTCGACAGAATGGCTTTTATGGCATTTGTTCATTATGTTGTGTTTG
TTAAAGACCAGTATCTTCAAGTATAGAATTGACAAGTAATTAGCAAGCTAAATAGCTAA
ACTCAAGCAGTTACTTTGAGGAGCACGTTAACGTTTGCAGACAAGCGTCCTGTTTGGCT

AACAGCTGTTTAGTTGTAGCTAACAACATTAGCTAGCTAGCAACACTGTATATACAAAA
GTATGTGGACACCCCTTCAAATTAGTGGATTTGGCTATGACAGCCACACCCGTTGCTGA
CAGGTGTATAAAATCGAGCACACAGCCATGCAATCTCCGTAGAGAAACATTTGCAGTAG
AATGGCCTTACTGAAGAGCTAAGTGACTTTCAACGTGGCACCGTCATAGGATTCCACCT
TTCTGCCCTGCTAGAGATGCCCAGGGCACTTTAAGTGTTGTTATTGTGAAGTGGAAACA
TCTAGGAGCAACAATAGCTCAGCCGCAAAGTGGTAGGCCACACAAGCTCACAGAATGGG
ACCAGTAGCGCGTAAAAATCTGACCTCGGTTGCAACACTCACCCCTGAGTT

It is unlikely a viable mRNA can form since there are no TATA boxes or poly(A) motifs in required positions. The start codon in exon 1 has been lost and there is no viable ORF for synthesis of a preproGnRH. The regions where exon 1 begins and where the end of the gene should be are roughly underlined.

Organism name: Salvelinus spp.

>gi|1340979674|ref|NC_036855.1|:20968917-20970863 Salvelinus spp. isolate IW2-2015 linkage group LG15, ASM291031v2, whole genome shotgun sequence

TTGCACCACACAATTATGCTGTGGAATTAATTTTTCATATAAGACTGTGGGTGTTGAAC
TGTATTCAACAGAATTTCAAGTATGCAATGTACTCTTTCCTTGCTGCCTGTCGTTTTTG
GTGTTCTCCGTGAATCTACCTCCTTATTTCTACCCCTGTCTGATATCTAGTTGCATGC
ATTTCACTGTAGTTTTGGCTCTTGTTGTATGAAATTCATCATATCCAAATATTATCAAA
GTGAATGTGTGTTTTAATTGTGTTACTGTCATGTTCAAGCTTGTGTAGCACTTAGTTGT
ATAACACTCCCCTCAAGGAGACCACAACCAATGAACAGGGCATATCATTCAGTCATGTC
TTGAAATTTTGCTTTGCACAAATGTCTAAATGGTATGTTTTGGTCAGTAACTTCAGTTT
TACTCACTCATAGGACACCAAGTTAAATGTGCTTGTAACATAATGTATTTCAAATCTAG
TGCTTCCAAAACATTATTAGGATAGCAAAATGTTATCACTGCTGGACTGTGATTGTTAC
AATCTCAGTTCTTTTAGAACAATGTCTGCATGTTTAAAGACTGCATTTTCTCTGGCTGT
AGCTGAAAGAGTTTGAGATGCAATATTCTCTCTATACTCTATTTCACAGTAGGTAGGTA
CTCATCTGTCATGATAATGAACACAATCATAAAGAATATTGCACCTTGAGTGTGGAGAT
CGTCAATATCAGAACCTTGAAGGAACAATGGTTATTTATTTTGATATAACATTGTATTC
AAGAAATATTGGATGAAAGTTCTTTAAATATCGGGTGTGTTTCTCTTTTTTCCCTGTCT
ATAATTATTGCACTCAACCACAAAAATGTAATTCAATAAATTACTGTAATTTTTTAAAA
AGTGTCTGTGTAATGAATTACTTGTGCAAATTAAATAATGGTATCTTTTCACTAACTTT
CCAAAATAACTGCCTTGTGCCTAATTAATTTCATTGTTGCTGCTGTAAAAGTGCACAAT
CATACCTGAAGTATTTTAGGCTTTTAAAAGGCTCTTTGGGTTCCTGAAGTGGAGATACT
TAGTGCTGCTCTGCAATATATGTTTGAGTGGATCCTTTTTTCTTTAATTCCACTTTTTT
TGTGACCTCTGGTGAAGTGATGGGACTAAGGGGCCAATAAAACCTCAGACACATTAACT
GCTCAGAGTAAGGCTGACTATTTGAAGAATAGAAGACAAACATTTCCGGTTGTTGCTGC
TGCTCTTGGTGGTGGAACTACTGT**CAACATTGGTCCTATGACATGAGCCGAGGG**GGGAA
GAGACAGTCTGGCAGCCTGTCTGAAACCATGGGAAAT**GT**AAGTATTTTACCTATTCATA
ATGTGAAAGTGGAAGTAGGTCTATTCACTAAAGGTTGTGATGGTGCTTATCCAATGATG
GGAGGGATGTTCCTATAGTTGCATAGTAAATTCTAGCTAAATGACAATTCTA**CAG**ATGG

CTGAAGATGGAGTTTGTTTGGTTGTGATGTCTCACCTCATGCCAAAATGTACAGACTAA
GGGAATTT**GT**GAGTGACATTTACAATTTTGCAAATAGGGCAGTTTCCCTTCAAGAAGCT
ACCCAGAAGTTGAAAATAAATGGAAATTGTGTTGGTAGTTACTTTTTTCATTGATGTGT
TTTTTTTCAAGGTTTTATCCAAATTGTAAACTATATTTTTAATTTAGTTTTTTTG**TAG**G
CAACCCTCGCTGACAGAAAGTGGACTCAAAAATATATTGCAAATGTATGCTAACGCTAA
TAAAAATTGCAGTGCCATCACTGTGTTTTGTGGTGGTCTTTGTTGGTGGTTTGCATATT
ACGGAGTGTGGCTTTAAAATAGTAGAAAAACACCCATTCAAATTTCTGTAGAGCACGGT
GTCAAAACAAGAGTGCCAACCAATGACTGCGAGTATGTGTTACGTTGTCAACTCTTGTA
GTAAACCTTGATTTAGAAATGTCGACAGAATGGCTTTTATGGCATTTGTTCATTATGTT
GTGTTCGTTAAAGACCATTATCTTCGAGTACAGAATTTACAAGTAATTAGCAAGCTAAA
TAGCTAAACTTGAGCAGTTACTTTGAGGAGCACGTTAACGTTTGCAGACAAGCGTCCTG
TTTGGCTAACAGCTGGGTTAGCTGTAGCTAACAACATTAGCTTGCTAGCTACACTGTAT
ATACAAAAGTATGTGGACACCCCTTCAAATTAGTGGATTCGGCTATGACAGCCACACCC
GTTGCTGACAGGTGTATAAAATTGAGCACACAGCCATGCAATCTCCATAGACAAACATT
TGCAGTAGAATGGCCTCACTGAAGAGCTAAGTGACTTTCAACGTGGCACCGTCATTGGA
TGCCATCTTTCTGCCCTGCTAGAGATGCCCCGGGCAACTTTATGTGTTGTTATTGTGAA
GTGGAAACATCTAGGAGCAACAACAGCTCAGCTGCAAAGTGGTAGGCCACACAAGCTCA
CAGAACGGGAGTGCTGAAGCACGTAGCGCGTAAAAATCTGACCTCAGTTGCAACACTCA
CCCACGAATTACAAATTGCATCTGGAAGCAATGTCAGTACAAGAACTGTTGGG

There are sequences in the remnant of the first exon that could encode QHWSYDMSRGGKR (in bold). However, translation cannot occur due to the lack of any upstream in-frame ATG codons in exon 1. We show the sequence that may have once been the ATG start codon (underlined). As well, no vertebrate GnRHs exist that do not have a proline in position 9. Finally, any potential translation of the GAP would be interrupted by stop codons and the open reading frame is degenerating, although the exon/intron boundaries seem relatively intact.

Organism name: Oncorhynchus mykiss (rainbow trout)

>gi|1207596390|ref|NC_035082.1|:23777153-23780620
Oncorhynchus mykiss isolate Swanson chromosome 6, Omyk_1.0, whole genome shotgun sequence

ACTAGCTTTAAATCTCTTGCACTAGAGCACATCCAGGGTTATCCGTTTCTGTTATGTAG
TGGAATATTTTAAATGTCTGACTGTCTTTCCTTATTTTATTTCCCAAAACCAAAATATT
GAATGTTCTTTATGTATGTTTACTATTATATCTGGATTCAATCCTAACTTATTTTTATT
ATTTCTAGATGTGCATTTTCTTGATTATTTAATGTCTGTAAATCAAAGATTATCAAACT
ATATAATTGCATGAAAGTAAAAGGGCCTCAATCAAGACTGTGGTAAAGCATAAATGGGA
GATGACATTGTGACTGTACATTCTTATCAATGTACTTAAAGTGCCAGATATGCTTTATG
ATTAGGATGTTTATTTTCTAAGTCAAAGCCATACCGTAGCATGGTGAAAATGGCAAATA
ATTTGCTGTTATTGCACCACACAATTATGCTGTGGAATTCATTTTTCATATAAGACTGT
GGGTGTTGACCTGTATTCAACCGAATTTCAAGTATGCAATGTACTCTTTTTCCTTGCTG
CCTGTTGTTTTTGGTGTTCTCTCTGAATCTACCTCCTTATTTTCTACCCCTGTCTGATA

TCTAGTCTAGTTGCATGCATTTCACTGTAGTTTTTGGCTCTTCTTGTATGAACTTCATA
TCCAAATATGATAAGTTATCAAAGTGAATGTGTGTTTTAATTGTGTTACTGTCATGTTC
AAGCTTGTGTAGCACTTAGTTGTATAACACTCCCCTCAAGGAGACAACAACCAATGAAC
AGGGCATCTCATTCAGTCATGTCTTGAAATTTTGCTTTGCACAAATGTCTAAATGGTAT
GTTTTGGTCAATAACTTTGGTTTTATTCACTCATAGGACACCAAGTTAAATGTGATTGT
AACAATGTATTTCAAATCTAGTGCTTCCAAAACGTTATTAGGATAGCAAAATGTTATCA
CTGCTGGACTGTGATTTTTACAATCTCACATCAGTTCCTTTAGAACAATGTCTGCATGT
TTAAAGACGGCATTTTCTCTGGCTGAGGCTGAAAGATTGAGACGCAATATTCCCTCTAT
ACTCTTTCACTGTCAATAGGTACTCATCTGTCATAATGAAGGCAATCATAAAGAATATT
GCACCTTGAGTGTGGTGATCCTCATTATCAGAACCTTGAAAGAACAATGGTTATTTATT
TTGCTATAACATTGTATTCAAGAAATATTGGATGAAATAGGTTTAAATAGGTGTTTCAC
TTTCCCTGTCTATAATTATTGCACTCAACCACAAAAATGTAATTCAATAAATGACTGTT
TTTTTTTTTTTAACTATCTGTGTAATTAATTATTTGTGCAAATGAAATAATGGTATCTT
TTCACCAACTTCCCAAAATAAGTCTATGCCCTGTGCCTAATTAATTTAATTGTTGCTGC
TGTAAATGTGCACAATCATGCCCAAAGTATTTTAGGCTTTTAAAATGCCCTTTGGGTTC
CTGAAGTGGAGATACTTCGTGCTGCTCTGCAATATATTCTTGAGTGGATCCTTTATTCT
TTAATTCCACTTTTTTTGTGACCTCTGACGTGATGGGACTAAGGAGCCAATAAAACCTC
AGACACATTAACTGCTCAGATAGAGTAAGGCTGACTATTTGTCGAATAGAAGACAAACA
TGCCCTGTTGTTGCTGCTGCTCTTGGTGGTGGAACTACTGTCACAGGGTTGCCGT**CAGG**
ATTGGTCCTATGACATGAGCCCAGGGGGGAAGAGACAGTCTGGCAGCCTGTCTGAAACC
ATGGGAAAT**GT**AAGTATTTTACCTATTCATAATGTGGAAGTAGGTCTATTCACTAAAGG
TTGTGATGGTGCTTATCCAATGATGGGAGGGATGTTCCTATAGTTGCATAGTAAATTCT
AGCTAAATTATAATTCTA**CAG**ATGGCTGAAGATGGAGTTTGTTTGGCTGTGATGCCTCG
CCTCATGCCAAAATGTACAGGCTAAGGGAATTT**GT**GAGTGACATTTACAATTTTGCAAA
TAGGGCAGTTTCCCTTCAAGAAGCTACCCCATAAGTTGAAAATAAATGGAAATTGACAC
ATTTATACACTGATACATTGGCGGCTAAAGAAATAATAGCAAAGAACTCAGAGATAGCC
TATAGGCAAATGCAGCAGCCAATGGTGACCCATCATTCAAGGCAGATGGGGCAGAGCCC
CACATATCAATTTGCAAACAATGTAAAACATTTTTTATCATTGAGTTAATAAAGCCACA
TACAACTTGGGTCTCTTTTTTCATTCTTGAGTAAGGCAGCTCCAAAATTCAGGTGTTTT
AGCCTAGCTCAGTGCTTTCTGTCGTGGTGTAGCAGCCAGTGGAAAATACGGAACGTAGG
GGTTGGTAATGTTCTCTAGCTGCGCCATGATTGCCTCAATGTTCTGTCACTCATTGGGA
AACTATGTCACCACAAAATCTACGGGTAGAGCTTGAAAATCCAAGCCCCTTGGGTGCTG
CCATAGAGTTACACTAGAAGTGCCCATCCAACAAGGCTCAAAGTCATTGGCCACAGATC
AAATGATGTCAAATCACGTTATACAGTGGGGCAAAAAAGTATTTAGTCAGCCACCAATT
GTGAAAGTTCTCCCACTTAAAAAGATGAGAGAGGCCTGTAATTTTCATCATAGGTACAC
TTCAACTATGACAGACGAAATGAGAGAAAAAAAAAAATACAGAAAATCACATTGTAGGAT
CTTTTATGAATTGATTTGCAAATTATGGTGGAAAATAAGTATTTGGTCACCTACAAACA
AGCAAGATTTCTGGCTCTCACAGACCTGTAATTTCTTCTTTAAGAGGCTCCTTTGTCCT
CCTCTCGTTACCTGTATTAATGGCACCTGTTTGAACTTGTTATCAGTATAAAAGACACC
TGTCCACAACCTCAAACAGTCACACTCCAAACTCCACTATGGCCAAGACCAAAGAGCTG
TCAAAGGACACCAGAAACAAAATTGTAGACCTGCACCAGGCTGGGAAGACTGAATCTGC
AATAGGTAAGCAGCTTGGTTTGAAGAAATCAACTGTGGGAGCAATTATTAGGAAATGGA
AGACATACAAGACCACTGATAATCTCCCTCGATCTGGGGCTCCACGCAAGATCTCACCC
CGTGGGGTCAAAATGATCACAAGAACGGTGAGCAAAAATCCCAGAACCACACGGGGGA
CCTAGTGAACGACCTGCAGAGAGCTGGGACCAAAGTAACAAACGCCTACCATCAGTAAC
ACACTACGCCGCCAGGGACTCAAATCCTGCAGTGCCAGACGTGTCCCCCTGCTTAAGCC
AGTACATGTCCAGGCCCGTCTGAAGTTTTCTAGAGAGCATTTGGATGATCCAGAAGAAG
ATTGGGAGAATGTCATATGGTCAGATGAAACCAAAATATAACTTTTTGGTAAAAACTCA

ACTTGTCGTGTTTGGAAGACAAAGAATGCTGAGTTGCATCCAAAGAACACCATACCTAC
TGTGAAGCATGGGGGTGGAAACATCATGCTTTGGGGCTGTTTTTCTGCAAAGGGACCAG
GATGACTGATCCGTGTAAAGGAAAGAATGAATGGGGCCATGTATCGTGAGATTTTGAGT

The remnants of exons 1, 2 and 3 cannot encode a functional signal peptide or GAP. There are no obvious start codons and the GnRH1 decapeptide would resemble no other known GnRH (QDWSYDMSPG if encoded). Exon 3 is no longer easily detectable but a potential diminishing remnant of it is shown (underlined).

Organism name: Oncorhynchus nerka (sockeye salmon)

>gi|1681300710|ref|NC_042561.1|:16777400-16780350
Oncorhynchus nerka isolate On170113-E2 linkage group LG27, Oner_1.0, whole genome shotgun sequence

TTGCATGAAAGGGCCTCAATCAAGACTGTGGTAAAGCATAAATGGGAGATGCCATTGTG
ACTACATTCTTATCAATGTACTTAAAGTGCCAGATATGCTTTATGATTAGGATGTTTAT
TTTCTAAGTCAAAGCCATACCGTAGCATGGTGAAAATGGCAAATAATTTGCTGTTATTG
CACCACACAATTATGCCGTGGAATTCATTTTTCATAGAAGACTGTGTGTTGAACTGTAT
TCAACAGAATTTCAAGTATGCAATGTACTCTTCTTTTTTCTTTTTTTTTGCCTGTCGTT
TTTTGGTGTTCTCTGTGAATCTACCTCCTTATTTTCTACCCCTGTCTGATATCTAGTTG
CATGCATTTCACTGTAGTTTTTGGCTCTTGTTGTATGAACTTCATATCCAAATATGATA
AGTTATCAAAGTGACTGTGTGTTTTAATTGTGTTACTGTCATGTTCAAGCTTGTGTAGC
ACTTAGTTGTATAACACTCCCCTCAAGGAGACAACAACCAATGGACAGTTAATCTCATT
CAGTCATGTCTTGAAATTTTGCTTTGCACAAATGTCTAAATAGTATGTTTTGGTCAATA
ACTTCAGTTTTATTCACTCATAGGACACCAAGTTAAATGTGATTGTAACAAAGTATTTC
AAATCTAGTGCTTCCAAAATGTTATTAGGATAGCAAAATGTTATCACTGCTGGACTGTG
ATTTTTACAATCTCACATCAGTTCTTTTAGAACAATGTCTGCATGTTTAAAGATGGCAT
TTTCTCTGGCTGAGGCTGAAAGATTTTGAGATGCAATATTCCCTCTATACTCTATTTCA
CTGTCAATAGGTACTGATCTGTCATGATAATGAAGGCAATCATAAAGAATATTGCACCT
TGAGTGTGGCGATCCTCATTCTCAGAACCTTGAAAGAACAATGGTTATTTATTTTGATA
TAACATTGTATTCAAGAAATATTGGATGAAAGTTAGTTCTTTAAATAGGTGTGTTGCTC
TTCTTTCCCTGTCTATAATTATTGCACTCAACCACAAAAAGGTAATTCAATAAATTACT
GTTATTATATATTTTTTTTAACTATATGTGTAATTAATTACTTGTGCAAATGAAATAAT
GGTATCTTTTCACTAACTTTCCAAAATAAGTCTATGCCCTGTGCCTAATTAATTTAATT
GGTGCTGCTGTAAAAGTGCACAATCATGCCTAAAGTATTTTAGGCTTTTAAAATGGCCT
TTGGGTTCCTGAAGTGGAGATACTTCGTGCTGCTCTGCAATATATTTTTGAGTGGATCC
TTTATTCTTTAATTCCACTTTTTTTGTGACCTCTGATGTGATGGGACTAAGGAGCCAAT
AAAACCTCAGACACATTACCTGCTCAGATAGAGTAAGGTTGACTATTTGAATAGAAGAC
AAACATGTCCTGTTGTTACTGCTGCTCTTGGTGGTGGAACTACTGTCACAGGGTTGCTG
TCAGCTTTGGT**CCTATGACATGAGCCCAGGG**GGGAAGAGACAGTCTGTCTGAAACCATG
GGAAAT**GT**AAGTATTTTACCTATTCATAATGTGGAAGTAGGTCTATTCACTAAAGGTTG
TGATGGTGCTTATCCAATGGTGGGAGGGATGTTCCTATAGTTGCATAGTAAATTCTAGC
TAAATGACAATTCTGAAGATGGAGTTTGTTTGGCTGTGATGTCTCACCTCATGCCAAAA
TGTACAGGCTAAGGGAATTT**GT**GAGTGACATTTACAATTTTGCAAATAGGGCAGTTTCC

CTTCAAGAAGCTACCCCATAAGTTGAAAATAAATGGAAATTGACACATTTATACACTGT
GATACATTGGCGGCTAAAGAAATAAGAGCAAAGGACTCAGAGATAGCCTATAGGCAAAT
GCAGCAGCCAATGGTGACCCATCATTCAAGGCAGATGAGCCCCACATATCAATTTGCAA
ACAATGTAAAACATTTTTTATCATTGAGTTAATAAAGCCACATTCAACTTGGGTCTCTT
TTTTGCTTTCTTGAGTAAGGCAGCTCCAAAATTCAGGTGTTTTAGCCTAGCTCAGTGCT
TTCTGTAGCAGCCAGTGGAAAATACGAAACGTAAGGGGTTGGTCATGTTCTCTAGCTGC
GCCATGATTGCCTCAATGTTCTGTCACTCATTGGGAAACTATGTCACCACAAAATCTAC
GGGGTAGAGCTTGAAAATCCAAGCCCCTTGGGTGCTGCCATAGAGTTACACTAGAAGTG
CCCATCCAACAAGGCTCAAAGTCATTGGCCACAGATCAAATGATGTCAAATCACGTTAT
ACAGTGGGGCAAAAAAGTATTTAGTCAGCCACCAATTGTGCAAGATCTCCCACTTAAAA
AGATGAGAGAGGCCTGTAATTTTCATCATAAGTACACTTCAACTATGACAGACAAAATG
AGAAAAAAAATCCAGAAAATCACATTGTAGGATTTTTAATGAATTTATTTGCAAATTAT
GGTGAAAAATAAGTATTTGGTCACCTACAAACAAGCAAGATTTCTGGCTCTCACAGACC
TGTAACTTCTTCTTTAAGAGGCTCCTTTGTCCTCCACTCGTTACCTGTATTAATGGCAC
CTGTTTGAACTTGTTATTAGTATAAAAGACACCTGTCCACAACCTCAAACAGTCACACT
CCAAACTCCACTATGGCCAAGACCAAAGAGCTGTCAAAGGACACCAGAAACAAAATTGT
AGACCTGCACCAGGCTGGGAAGACTGAATCTGCAA**TAG**GTAAGCAGCTTGGTTTGAAGA
AATCAACTGTGGGAGCAATTATTAGGAAATGGAAGACATACAAGACCACTGATAATCTC
CCTCGATCTGGGGATCCACGCAAGATCTCACCCTGAGGGTTCAAAATGATCACAAGAAT
GGTGAGCAAAAATCCCAGAACCACACGGGGGACCTAGTGAATGACCTGCAGAGAGCTGG
GACCAAAGTAACAAAGCCTACCATCAGTAACACACTACGCCGCCAGGGACTCAAATCCT
GCAATGCCAGACGTGTCCCCCTGCTTAAGCCAGTACATGTCCAGGCCCGTCTGAAGTTT
GCTAGAGAGCATTTGGATGATCCAGAAGAAGATTGGGAGAATGTCATAT

Exons 1, 2 and 3 are degenerating and could not encode a functional preproGnRH. Both exons 2 and 3 are not easily identified. A portion of exon 2 is intact (underlined) but is incomplete. We have selected several stretches of DNA that may represent remnants of exon 3 (underlined regions downstream from **TAG**).

Online Resource 2

The late-evolving salmon and trout join the GnRH1 club

Histochemistry and Cell Biology

Kristian R. von Schalburg, Brent E. Gowen, Kris A. Christensen, Eric H. Ignatz, Jennifer R. Hall, Matthew L. Rise

Corresponding author at: Department of Biology, Electron Microscopy Laboratory, University of Victoria, Victoria, British Columbia, Canada V8W 3N5

E-mail address: krvs@uvic.ca (K.R. von Schalburg)
