# Supplementary material for: The late-evolving salmon and trout join the GnRH1 club
Source: Histochem Cell Biol. 2023 Aug 11;160(6):517–39. doi: 10.1007/s00418-023-02227-z (PMC10700215; doi:10.1007/s00418-023-02227-z)
Supplement: Supplementary file 3 — Supplementary file3 (PDF 184 KB) [file 418_2023_2227_MOESM3_ESM.pdf]

Online Resource 3 Presentation of the GnRH2 genes for grayling, whitefish (*Coregonus* sp. 'balchen'), Atlantic salmon, a *Salvelinus* spp., rainbow trout and sockeye salmon. The 5'-utr, ATG start codons (green), GnRH-encoding sequence, exon/intron GT-AG donor/acceptor sites and poly(A) motifs are highlighted in bold for each gene of interest. Putative CREs and EREs in the proximal promoter of the GnRH2 Gene2 are underlined. We provide the putative mRNA and protein below each GnRH gene-type presented.

GnRH2 Gene1

Organism name: Thymallus thymallus (grayling)

>gi|1594667049|gb|CM015006.1|:33161836-33165434 Thymallus thymallus isolate TTM2012 chromosome 17B, whole genome shotgun sequence

TATTTGTCCGGTCATAAAGGTTCAGCGTTGTTTTGTCATTAGAACATAAGGAAAGCCCAC
AGTGTGTAGAAAAATAACTGCACAACCCCTACAGTTCAAGAGTCGGAATGGATTGCTTTA
GCTTCTCCCTTTATATATTTTTATAGGCTGAAAGGAAAGAGCACAATCCAATTCTGTTGT
GCGTGAATCATATGGGATCAGGAAATTAGAAATAGTCTCTGAAACTACAAAAAAAAGCAC
CATTATTGAGAAATTGTTGTTATTGTTTATTGTTTCGAATGCTTGAGCTAGTTGCACCCA
AAAGAATATCCGAACCAAGAGATAGTAAATTGGCAACTAGGGTTGCAAAATTATTTTATA
TTTTTTTATCCTGGTTAAAGAATTCCAGGTGGGAGGATTCCCTCGCAGCTTATTCCCTGC
TGATTTCAGAAATCCTCCAACCTTTAAATAAAATAAATGATAATAATTGCCACCCCAATG
TCAACATTGAAGAAGCCTTGTAAAAGACTCTGGTTATGGCCTCTGGTTCTGACTGGTTCT
CGTAGGTTAAACCACAGGACATGGATACCTAGGTACTTAGACCAGTCACTTTTCAGACCG
TGCCCGTGTGTTACGATTGATGCCATTTTGTTTTCCTTCCTGGATCTTTGTAATCGTAAA
GGTGGGCGTTTGTGTGTGGAGATGCGAGGCTTTTACTCTTGTCTTGAGCCAGAAGGTTTG
ATGAATGTAATCACCTGTCCACAATGACACTGTGCCAAAACCCAACCCTAGTGAATTAAG
GCTTTACTTTAGAAAGTGCCTCACGGGTTGATTTATATGGAAAATAAGTTCACTGTACCT
TTCCCTCAGTGAGTTGATTGAGTGTTGGTGTATAGATGCTTGGTAAACCAGTATTACCCT
ACAGATTAGAACATTATATTTGTCCCCTCCCCACTATTTTTGAGCCGTTGTAGATAACTT
TGTCGACAAAGCACCTCTGTACAAAGCCGTAGCTGTTGCTTGGCTTTGTAAAACTGTGAC
CAAGATCAGGGCCTTTTATTCTCCTGTTCGAGTTCATGCTTCACCAGCCTCCTTTCCATT
TCATGCTGTAATGCCGGGTTCCATTCCAATCCAACTCCATTCTTTTGTTTTGAACCAGCT
TTGTCTTGGCTCCATGTCTTAATTCCATTCCACTGAAGTGCTGGACCATTGACTATTCCT
TTATTGTGGCACCTCGTTGGGTCGTCTACCTAACCTTCACCCGCTATACTGGGTAAAATA
CATTGCCCTAAGTTACTCCAACTACAAATCACAATGACCATGGCCATTACAGAGGATTAT
TAGAGAGCACAGCCAGGAGCATCTGGGCCCTTGTTCCACACCCCTGCTGGCTGCCGATAG
AAATGTGACGCAACACCCCAGTAATGTAATGTCTGAGCAACAGGGCACACCTTTCTTCAG
AGACTGAGAAAGAGAGCGAGAAAGACAGGAGGAGAAATACAGGTTTAAGAGGTGGGAGAG
CCAGAGAGACCGGTTTAAGAGGTGGGAGAGACCGGTTTAAGAGGTGAGAGAAACAGGTTT
AAGAGGTGGAGGAGTGGGGCTTTTTAGGGTTCAACCAGTCTCAGTCTCTCCCACCTCTAA
ACCTCTCTCTCCCACCCTTAAACCTCTCTCGCTCATAAACCTGTCTCTCCCACCTCTTAA
ACCTGTCTCTGTCTCTCCCACCTCTTAAACCTGTCTCTCCCACCTCTTAAACCTGTCTCT
GTCTCTCCCACCTCTTAAACCTGTCTCTTCCACCTCTTAAACCTCTCTCTGTCTCTCCCA
CCTCTTAAACCTGTCTTTCCCACCTCTTAAACCTGTCTCTCCCACCTCTTAAACCTGTCT

TTCTCCTCCTGATCCTGTTTAGCTGTCCCTTGGATGCGCCCAAAGATCTCTCTCTCGCTC
TCTCTCTCTTTTTCCCTTAACTCCTATCTCTTTCTTCTCCCTCTCTTGTTCTATTTGCCC
CTTGGGCTGGTTTGGTCTCCCCTATAACCGGTCACTGCCCTACTCCCTTCTGTACATAAT
GTAGGGTTATTTATATTTCATTCCTGTCTGTTGCCTTGCTTTTTAGTTGTTTTTTGTAGA
AAATGTACATTAACTGTACTGGGACCATTTAGAAATTTATGAGATATTTTGTGTTTTTCT
TGCTTTTTTAAATTTGGTATTATTGTCTTAAGAAAATCCTATTTTTTATTTGCATCTGTT
TTGTTTTCTAGTGTTTTGTTATAAATATGTATATATAATTATATATATATGATACAATTA
TATATAATTATATAATGCCAAATGGCCTTTCTAAGCAAGATATTGTTTAAACCTAATAAA
AAATATTTCAAAAACCTATTTGTTTATTACCTACAGCATTATCTGGTGAAATCTGCTGTA
ATTACTGATGTTCAGTGGTAACACCAATCCCAACATCAAAACTATAATCTTCAATACTGG
AATAGTTTCACTCTAATACTACTAGCCACCTAGCAATTTTATGAAGTTGGCTTTAGCTAG
CCCAGATAGGTTCCCAATCTCCTAACCTCAGAAATAGCTATCAAGAAGCCATTTCAGGCT
ATCATTCAAGTTAGAGTAGCTAGCTTGTCTATTTTAGCTGGCATGCCTGCTGGCAAGGTT
TGTAGACTTTAGAAAAGCAAGTAATTACTAAATATACTGAATAAGACTCGCATTCCTTTC
AATCTCTTACTCAGATTTTTGCAGAGATGCAGAGAATCATATTTAGTTTCTTTAAAAAAA
TAACCATCAGTCAGTAGGATAGACAGCTCGAGGTATGCTAAGATATCCAGAAAAATAAAC
ATGTTTTTTTTTTACATAGAAGTAAGCATAATGATTATGGCTCTAGGTTGAAGGAAAAAA
CAGTTTCAGTTGTTAGAAAAAAATAATATTCTTCAACTTCCACCACCCAGCCATCCTTAC
GTATTTTGTGCCCCCTCAGATTTTTGGGGTGCAGGCTTTTGTTCCAGCACTAACATACCT
GATCCAACTAATCATGGTATAATCTGAAAATCAGGATTAGTAATTAGGTGTGTTAGCGCT
GGGCTGGAACAAAAGACTACACATCCATCTCTGCGAGATCCATCTTGACTGTCCCATACC
CTTGATAAGGTCCAAGGCCCCTATTCACAGCGTCTAAGAGTAGGAGGGCTGATCTAGGAT
CAGGTCCCCCCTCTTCATTATGGTTTAAAAGGCAAAACTGATCCTAGATCAACTGATCCT
ACATCCTACTCTAAGACGTTTTGTGAATACGGCCCAGGTTCTCCGCTTCCGTTGGCGGGC
CAGGTCTCAGGAAGGTGTGGCGGGTGAGAGGAATTATCCTCGGTGACTCATGCAGCGCTC
CATCATGTCCTCCTGCTTCCTGCGCCCCCCTCGCACTGGCAGCCCCATGTCAGTCAGCAG
ATTAACTAAGTCAATGGCGAGAAGACAAGCTGTCAATCAACCGCCCGATAGGAACTCCCC
CTGCGCATTTTAAACATGAACATTTTCCAGTCCCCAATTGATACCCCCTCACCCAAGCCA
AAAGGCCTGTCAGACTGGAACTGAGAGAGATGGTGGCGGCTGGTGTGTTTGCCCGTGCTC
GTGTTTGCTTGTCATCCGGTGTCCCCATGGTCAAAGTGTCCTGGCGGTCACTCGGGTGTG
ATGGAGGGCGCGTGACAGGTCACGGCACCACTTTAAGCAGGAGCAAATACAGAGGAAATG
GGATTTGGCCTAGGTCAGTGATCCTCAACCCCTGGTCTGCAGACCAACACTGGACCGGTC
CCACAGATTGTTAGTGAAATTACACCAATTTGTCAGTTCTCAAGTGCTAGTTGTAGGTGT
CCCTCTAAGCAGGAAGGGGACAACCGGTTGCCAGTCCCTGGTAAAGTAAGGACCATAGCT
TGCCGGTCCCTGATATAACAGGGTTGAGAAACATTGAAGATTCGATGTGTTCAATCTTCG
TTAGTCTCAGGTTTTGTCAGATTGTCTATGTGTCCTCCACAGTTCAAAAATCTGAACGCA
ACTGACAAAATCTGAACCAGTCCAGTTAAAATTTTCCTTCCAATACATTTTTCCTTGAAC
GTGATGTGTGAAGAAGATGAGAGAACGTCATGAAGTGACAGAACAGGATTCATCCAGCCA
CATGATGTTTATAGTAAACGACCTCAACGTATTGATTTTGGTGGCTGGGAGCAGGGGAGC
GGGGTGTATTAGGTGCAGACAGGACAAACATCCCAGGGGAGGTCAGGACTATTTGGAGAT
GATAATTGGAATTGAGCGGATCTTACGCACACCCCGCGTCTCTAAACACCACTGAGCTGA
CCGCTGGGAGGGAAAGGAGCAATGGGGTCAGCGATGGGCGGGGCGAGGTTGGGTCATAAA
TTGAGGAGGAAGAGGAGGCTTC**AGAGATATCATCAGAGAGCAGCTCTACAACACTTATCG**
TCGCCCCAGGAAAAGTGATTTACCACTGAGTTGCTGTATAACAACAGGTTAATAATAGGC
AGACGTGAGCAGCGGGCACACATAGACAGACTTGGTGGACATCAACAGTGATTACCGATT
CCATACAACAGGTATTGTCAGACTGAGGATCTAAGGTAAGGATTGCTGTGATTTTTGCTA
ATGGGTCTTGGAGAGTCTTTGATGCTTTCGATGAAGTATACATTAAACCCATAGACAGCT
ACATTTATTGGTTCCATTGTATGGTGAATGAGTGGTGGCAGTACCAGTATATATTTGAAG

TTATGAGCTGCACACAAAAAAACTTTAAGGCTTGTTGCTTTTTGCTGTTTGCCTCTCCAA
TCGTGCCTCTTGAATAATTACCCTAATTTGCCTGGCAGATCAGACACCGTCATCATACTT
CTGACACCAATGTAGCGACCATGTAGCAGTAGGTACGGCCACAGGGGATGTTCAACCCCT
GGTTGTCTAACCCCAAGGCAAGCATAGTCTCCTTAGGCAGACGGTTGCCTCCCACAATGT
ATCCAATGCTACTGTTGCCTAGGGTCGGGTTTACGAGACTAAGGCAAGCATACAGTTCCA
AGAGAATTAACTCCCTTGAGAGCTCTAGGGTCATCACAGTTTTCTCCCCACTCTCTCATT
CCCGAGGCATGAATTGTATAACGATCCCCCATCCCTCCCTTTCTTTTCTTGTGAAAGGTG
TGTAAGCCTGACAGGTGTCTCATGCTCTCTTACCCACACCCTCCCTCTCCTTATATCTCT
CCATCCCTCTCTCTCCAG**CTCCAGTGATG**GTGAGTGTGGCTAGGCTGGTGTTGATGCTGG
GGCTGCTGCTGTGTCTGGGAGCCCAGCTGTCCTCCACC**CAGCACTGGTCCCATGGCTGGT**
ACCCAGGAGGCAAGAGGGAATTGGACTCATTTACCACCTCTGAG**GT**GGGTCACAGTACTA
CAATCGCAATGACTCAAATGGATATTAAGTCTGCGTAAAATACACAGTAGCCATTTGTGT
CACCATCATTCTATTATCCCTCTCTCTCTCT**CAG**ATTTCAGAGGAGATTAAACTCTGTGA
GGCAGGAGAATGCAGCTACCTGAGACCACAGCGAAGGAACATCCTTAAGAACATTCTT**GT**
GAGTGCAGGGGCTTCCTTACGAAAAAAGATTGCAGTCAGAGCGCATTAAAACTGCAGTTA
GAGTGCAGTATAACTGCAGTATGCAGCAAATACTACGTCCAAAATAACACCGTTTTTTTT
TACTGCAGTAATTTTGCAGTGTAACTGCAGTTAGAGTGCTGTATAACTGCAGTACACTGC
AGTTATTCTGTATCCAAAATACCACAGTCGACTGCAGTTACTGCACTTTTACTGCAGTTT
CAAAACTGCAATCTATTTTTGTAAGGGCGGGAAGGCAGTAGTAAATTAATGTAATGATAA
TGTATCAGGTGGCATTCATATAGGCCACTGATACGGTAGCTCAAATCTGGGAGGCCGGAA
TACTGCTTGTTTAAATTTCACTGATTGGTGGAGGAAAAATGTATATTGCACCTGTAATTA
ATGTGGTGTTACTCTTATTG**CAG**TTGGATTCCCTTGCCAGAGAATTCGAGAAAAGAAAGT
AACCTATCAACTGTGGCTGACTTAAATCCTTCCTTGTCTATCTGTTTATTTCATATGACC
TCTATTCTAATCTTATGTCCTTTGATCCAATTTTGTGGTTTATCCATTTTGAT**AATA**GTG
TAGTGTCACTCCTTTACTGTCCCTCATTATTGTCCCTTTTTTCTCCTATAGACTCAATTA
CAGATGTAACATCATGCACATGACATTTCCTGT**AATAAA**GTCTCTATTTTGTTATTAATG
TCTTCATATTTGTCCTCTTCAATTAATTTGATTCAAATAAAGAATAGGTATAGAAATATA
CTGAACAGAAATATAAAAGCACCCTGCAAAGTGTTGGTCCGATGTTTCATGAGCTGACAT
AAAAGATCCCAGAAAGACTCCAGTCAACCTAGTCATAGACTGTTCTCTCTGCTCCCGCAT
GGCAAGCGGTACCGGAGCACCAGGTCTAGGTCCAAAAGGCTTCTTAACAGCTTCTACCCA
CAAGCCATAAGACTTCTGAACAGCTAATCAAATGGCTACCCGGACTATTTGCATTGTTGT
CGTCATACGTAACACTTTCTTAAAACTAGGGTTTGTAAGTAAGCATTTCACTGCAAGGT

mRNA:

AGAGATATCATCAGAGAGCAGCTCTACAACACTTATCGTCGCCCCAGGAAAAGTGATTTA
CCACTGAGTTGCTGTATAACAACAGGTTAATAATAGGCAGACGTGAGCAGCGGGCACACA
TAGACAGACTTGGTGGACATCAACAGTGATTACCGATTCCATACAACAGGTATTGTCAGA
CTGAGGATCTAAGCTCCAGTGATGGTGAGTGTGGCTAGGCTGGTGTTGATGCTGGGGCTG
CTGCTGTGTCTGGGAGCCCAGCTGTCCTCCACC**CAGCACTGGTCCCATGGCTGGTACCCA**
GGAGGCAAGAGGGAATTGGACTCATTTACCACCTCTGAGATTTCAGAGGAGATTAAACTC
TGTGAGGCAGGAGAATGCAGCTACCTGAGACCACAGCGAAGGAACATCCTTAAGAACATT
CTTTTGGATTCCCTTGCCAGAGAATTCGAGAAAAGAAAGTAACCTATCAACTGTGGCTGA
CTTAAATCCTTCCTTGTCTATCTGTTTATTTCATATGACCTCTATTCTAATCTTATGTCC
TTTGATCCAATTTTGTGGTTTATCCATTTTGAT**AATAG**TGTAGTGTCACTCCTTTACTGT
CCCTCATTATTGTCCCTTTTTTCTCCTATAGACTCAATTACAGATGTAACATCATGCACA
TGACATTTCCTGT**AATAAA**GTCTCTATTTTGTTATTAATGTC

protein:

MVSVARLVLMLGLLLCLGAQLSST**QHWSHGWYPG**GKRELDSFTTSEISEEIKLCEAGECS
YLRPQRRNILKNILLDSLAREFEKRK

Organism name: Coregonus sp. 'balchen' (whitefish)

>gi|1711366354|emb|LR664355.1|:45754800-45759000 Coregonus sp. 'balchen' genome assembly, chromosome: 12

GGGTTAAAAAGTTAGCTCTTGGAAAGGATTTTAGCATTTCCTTTAGGTTTGATGCTTTGT
ATGAAGTATACATTAAACCCATAGACAGCTACATTTATTGGTTCCATTGTATGGTGAATG
AGTGGTGGCAGTACCAGTATATATTTTGGAGTTAGACGTTTTTAAAACATCCTAACATTA
CACTGTTAACGTGCCTTTCTAAGTGGAATCTTGAGAAGAAATTACCACAACAATTGCCAA
TGAGAAACTGATCTTTGAAACCCACTGTGAACTTTACTGTATATCTTATTTTATTTTAAT
TTTAAAAAAAATTAGGGGGTAGATCAGCTTTAATATTGCAGATAGATTGTAACTTCCATC
AATGTAATTGTCTGCATCACTTCCAATCCCCCATATATTTTTTTCGCTAATATATATGTA
CATACATACATACATACATACATACATACATACATACATACATATACACATACATATACA
TACATATATATACACATCCTTCTTTTTTAATATAGTTTCCTTAATTACTTTCCAACCCCA
CCACCCCTCCCCTAATTGGAGTAAACTAGTGAACAACAACGCTTAGGCCTCTACTTCCAG
CATATACATACTATATACATTTTATGGACAGTCAATTTTACAATAGTTCTATTTTGTCTG
TTTTTACTCCTGAACTTCCTCTACCCTCAACCTCTCCGATCATTTTCATGATGTCCATCC
GGTTTGCTTCTATATGCCATATCTTTCAAACTGTGCTCTTTCACAAAAGTTCTCAACCTA
TAACCTATTTTTATTTATTTTTATTTCACCTTTATTTAACCAGGTAAGCCAGTTGAGAAC
AAGTTCTCATTTACAACTGCGACCTGGCCAAGATAAAGCAAAGCAGTGCGATAAAAACAA
CACAGAGTTACATATGGGGTAAAACAAAACATAAACATGTATATACTTATTATGGACACA
GTATGTTTTACATGAGTTATCTTGTTGTTATTAGTTGTTATTAGTCCCAACCCCCAACCC
CTCCCATCTATCTCTTAACACTTAATATTGGATTTCTATTTGCCATATATTTTTTAACTG
GACTGTGATGTTTCACAAAAGTTCTGAACCGTTCTATTCTCATTGTTACTACAGATTGTA
AATTTAAAAAATAAACATTTTTGCTAAAAGTATTATTATATTATTGATCAATTGATCACC
CAGTAGTGCTATCTGCATTCCTGGACCTGTGACCAAAAACAAGCTACAAATGGACAGTAC
CAAAATAAATTATCTAATGATTCTGTCTCTTCGCAGCAAAATCTGCAGAGCTGGGAAGAT
TGTATTCCCCATATAAATAACATTCTATTGGTAGCAAGAATTTTGTATAATAATTTAAAT
TGAAAAATTCAAGTTTTGAATCCGGCTTCGTTTTACGTATCAGTTCATAAACCACTTGCC
ATCGAATCGGTACGTCAAAAATCTCTTCCTAACTATTTTGCAATCTATATGGGACTGCTG
TCAATCCTTTGGTCCTTAAATGAAACTGGTATACTTTTTTATTTATCACAATTTTCTTTA
ACCAATGATGGTCTATGAACTTTACTGTATTTGTTTGTGGAGATTTTTTTCCCTGTGTAT
AAATATGATTAATGTATTGGATTAAATATTTATCGTGCATAAAATGAAGTTATGAGCTGC
ACACAAAAAAAACTTTAAGGCTTGTTGTTGCTTTTTGCTGT**TTGCCTCTCCAATCGTGCCT**
CTTCACTAAGATTTGAATAATTACCCTCATTTGCCTGGCAGATCAGACGCCGCCGTCACA
CTTTTGACACCAATGTAGCGACCATGTAGCAGTAGGTACGGCCACAGGGGATGTTCAACC
CCCGGTTGTCTAACCCCAAGGCAAGCAGACAGACGGTTGCCTCCCACAAGGTATCCAATG
CTACTGTTGCCCAGTGGTGTAAAGTACTTAAGTAAAAATACTTTAAAGTACTACTTAAGT
CGTTTTTATGGGGTATCTGTACTTTTCTGTTGATATTTTTGACAACTTTTACTTTTACTT
CACTACATTCCTAAAGAAAATAATGTACTTTTTACTCCATACAATTTCCCTGACACCCAA

AAGTACTAGTTACATTTTGACAGGAAAATGGTCCAATTCACACACTTATCAAGACAACAT
CCCTTGTCATCCCTACTGTCTCTGATGTGGTGGACTCACTAAACACAAATGCTTCGCTTG
TAAATGATGTCTGAGTGTTGGAGTGTGCCCCTGGCTATCCGTCAATAAAATTTAAAAAAA
GAAGGCATTTGAAATGGTTTATGCTTTTACTTTTGATACTTAAGTACATTTTATCAATTA
CATTTACTTTTGATACTTAAGTATATTTACTTTACTTTTACTCAAGTAGTATTTTACTGG
GTGACTTTCACTTTTACTTGAGTCATTTTCTATTAAAGTATGACAATTTAGTACTTTTTC
CACCACTGCTGTTGCCTAGGGTCAGGTTTACGAGACTAAGGCAAGCATGTAGTTCCAAGT
GAATTAATTCCCTTGAGAGCTCTAGGGTCTTCACAGTATTCTCCCCCCTCTCTATCACTT
TCTCTCGTTCCCGAGGCATGAATAATGATCCCCCATCCCTCCCTTTCTTTCCTTGTGAAA
GGTGTGCAAGCCTGACAGGTGTCTCATGCTCTCTTTCCCACACCCTCCCGCTCCTTACAT
CTCTCCATCCCTCTCTCTCCAG**CTCCAGTGATG**GTGAGTGTGGCTAGGCTGGTGTTGATG
CTGGGGCTGCTGCTGTGTCTGGGAGCCCAGCTGTCCTCCTCC**CAGCACTGGTCCCATGGC**
TGGTACCCAGGAGGCAAGAGGGAACTGGACTCATTTACCACCTCTGAG**GT**GGGTCACAAT
ACTACAATCGCAATGACTCAAATAGATATTACATCTGCGTAAAACACACAGTAGCCATTT
GTGTCACAATCATTCTATTATCTCTCTCTCT**CAG**ATTTCAGAGGAGATTAAACTCTGTGA
GGCAGGAGAATGCAGCTACCTGAGACCACAGCGAAGGAACATCCTTAAGAACATTCTT**GT**
GAGTGCAGGGGCGCCCTTACGAAAAAAAGTCAAAGTGAAGTATAACTGCAGTTAGAGTGC
AGTATAACTGCAGTATGCAGCAAATACTACGTCCAAAATAACACCATTTTTTTACTGCAG
TAATTTTGCAGTGTAACTGCAGTTAGAGTGCAGTTATTCTGTATCCAAAATACCACAGTT
GACTGCAGTTACTGCACTTTTACAGCAGTTTCAAAACGGCAAGCTATTTTTGTAAGGGCG
GGAAGGCAGTAGTACAGTAATGTAATGATAATGTATCAGGTGGCATTCATATAGGCCAGG
GAATTTGAATCCTTATTCAATGTGTCAGAGAGGCATGTTTATTGTACATAATGTATTTCT
ATCTGAACGTTCCATAATAATATGTTTCGTTGTCACATACAGCGAAATGTGTTGTTTTAC
AGGGTCAGCCATAGTAGTATGGCACCCCTGGAGCAAATTAGGGCTAAGTGCCTTGTTCAA
GGACACATGGACATCTTTTTCACCTTGACTGGTCGGGTATTTGAACCAGTGACCTTTCAG
TTACTGGCCCAATGCTCCAACCGCTGGACTACCTGCCACCCTTTAATGTTGTGCCCTACT
GAATGCCTGTGTCAAAGGAGAATAATAAAAGGCCAGTAGTGTGTGAGCATAGTATTTATT
TGTATAAATGTGTATTGTATCTACAGGTGTTACAATCATGCTGCATAGAGGTGGAAATGG
GGGGGGGGGGGGGGGGGGGGGGGGGGGGGGGGTGAATAGTGGAGGAAAAATGTATATTGC
ACATGTAATTCATGTCTTCATGTGGTGTTACTCTCTTTG**CAG**TTGGATGCTCTGGCCAGA
GAATTCGAGAAGAGAAAGTAACCTATCAACTGTGGTTGACTTAAATCCTTCCTTGTCTAT
CTGTTTATTTCATATGACCTCTATTCTAATCTTATGTCCTTTGATCCAATTTCGTGGTTT
ATCCATTTTGAT**AATAA**TGTAGCATCACTCCTTTACCCTTGCACCCTCTTTATTGTCCCT
TTTTTCTGCTGTAGACTCAATTACAGATGTAACATCATACACATGCCATTTCCTGT**AATA**
AAGTATCTATTTTGTTATTAATGTCTTCATCTTTGTCCTCTTCAATTGATTTGATTCAAA
TAAAATAATAGGTGTGGTCCTCTGTAGCTCAATTGGTAGAGCATGGCGCTTGTAACGCCA
GGGTAGTGGGTTCGATCCCCGGGACCACCCATACGTAAAAATTAATGCACACATGACTGT
AAGTCGCTTTGGATAAAAGCGTCTGCTAAATGGCATAATATATTATATTACTGAACAAAA
ATATAAACGCACCATGCAAAGTGTTGGTCCCATGTTTCATGAGCTGAAATAAAAGATCCC
AGAAATTTTCCACACAAACAAAAAGCTTATTTCACTAAAATGTTGTGCACAAATTGGTTT
ACTTCCCTGTTAGTGAGCATTTCTCCTTTGCCAAGATAATCCATCCACCTGACAGGTGTG
GCATATCAAAAAGCTGATTAAACAGCATGATCATTACACAGGTGCACCTTGTGCTTGGGA
AAATAAAAGGCCTCTCTAAAATGTGCAATTTTGTCACACAACACAATGCCACAGATGTCT
GGAGTTTTGAGGGAGTGTGCAATTGGCATGCTGACTGCCGGAATGTCCACCAGAGCTGTT
GCCAGAGAATTGAAAGTTCATATCTCCACCATAAGGAACCGCCAATGTCTTTTTAGAGAA
TTTGGCAGTACGTCCAACCGGCCTCACAACTGAAGACCACGGGATTGGGGTGCTGAGGAG
TATTTCTGTCTGTAATAAAGCCCTTTTGTGGGAAAAACTCATTCTGATTGGCTGGGCCT

GGCTCCCCATGGATCAGCCTAGCTCCAACGTGGGTGGGCCTATGGCCTCCCAGACCCACC
ATTGCTGCGCCCCTGCCCAGTGATGTGAAATCCAT

The exon 1 presented here is only presumptive. We have placed it in the equivalent position to exon 1 of the *Coregonus sp*. GnRH2 Gene2. The promoter region and exon 1 of the GnRH2 Gene1 are quite different in comparison to their counterparts in the other species examined here. Also, an unlikely poly(A) motif (AATAA) is shown here in the position equivalent to that presented by Vickers et al. (2004) for the GnRH2 gene in *Coregonus clupeaformis*. However, a more canonical signal (AATAAA) is found approx. 100 bps downstream, which has been selected as the bona fide poly(A) site for both GnRH2 genes. See our note on the poly(A) motif and end of the mRNA in the description provided for Gene2.

mRNA:

TTGCCTCTCCAATCGTGCCTCTTCACTAAGATTTGAATAATTACCCTCATTTGCCTGGCA
GATCAGACGCCGCCGTCACACTTTTGACACCAATGTAGCGACCATGTAGCAGTAGGTACG
GCCACAGGGGATGTTCAACCCCCGGTTGTCTAACCCCAAGGCAAGCAGACAGACGGTTGC
CTCCCACAAGCTCCAGTGATGGTGAGTGTGGCTAGGCTGGTGTTGATGCTGGGGCTGCTG
CTGTGTCTGGGAGCCCAGCTGTCCTCCTCC**CAGCACTGGTCCCATGGCTGGTACCCAGGA**
GGCAAGAGGGAACTGGACTCATTTACCACCTCTGAGATTTCAGAGGAGATTAAACTCTGT
GAGGCAGGAGAATGCAGCTACCTGAGACCACAGCGAAGGAACATCCTTAAGAACATTCTT
TTGGATGCTCTGGCCAGAGAATTCGAGAAGAGAAAGTAACCTATCAACTGTGGTTGACTT
AAATCCTTCCTTGTCTATCTGTTTATTTCATATGACCTCTATTCTAATCTTATGTCCTTT
GATCCAATTTCGTGGTTTATCCATTTTGAT**AATAA**TGTAGCATCACTCCTTTACCCTTGC
ACCCTCTTTATTGTCCCTTTTTTCTGCTGTAGACTCAATTACAGATGTAACATCATACAC
ATGCCATTTCCTGT**AATAAA**GTATCTATTTTGTTATTAATGTC

protein:

MVSVARLVLMLGLLLCLGAQLSSS**QHWSHGWYPG**GKRELDSFTTSEISEEIKLCEAGECS
YLRPQRRNILKNILLDALAREFEKRK

Organism name: Salmo salar (Atlantic salmon)

>gi|925216698|ref|NC_027321.1|:43720468-43724243 Salmo salar isolate Sally breed double haploid chromosome ssa22, ICSASG_v2, whole genome shotgun sequence

TCCTAGATTAACTGATCCTACATTCTACTCTAAGAAGTTTTGTGTAGCCCAAGTTCTCCG
CTTCCGTTGGCGGGCCGCGTCTCAGGAAGGTTTGGCACGCGTGATGAATTCTCCTCTGTG
ACTCATGCAGCGCTCTGTCATGTCCTCCTGCTTCCTGCGCCCTCCTCGCGCTGGCAGCCC

CGTGTCAGTCAGCAGATTAACTAAGTCAACGGCGAGAAGACATGCTGTCAATCAACCGCC
AGATAGGAACTCCCTATGCATTTTAAACATGAAAATTTGCCAGTCCCCAATTGATACCCC
CTCACCCAAGCCAAAAGGCCTGTCAGACTGGAATTAAGAGAGATGGTGGTGGCTGGTGTG
TTTGCCCATGCTCGTGTTTGCTTGTCATCCGGTGTCCCCATGGTCAAAGTGTTCTGACGG
TCACTCGGGTGTGATGGAGGGTGCGAGACAGGCCACTGCACCACTTTAAGCAGGGGCAAA
TACAGAGGAAATTAGATTTGGCCTTGGTCAGTGATCCTCAACCCCCGGTCTGCTGACCAA
CAGTGGATCCTGGAATGTTGTTGACCAGTCCCACAGACTGTTAACGAAATTACAGTTCT**C**
AAGTGCTAGTTGTAGGTGTCCCTCTAAGCAGGAAGGGCAACCGGTTGCCAGTCCCTGGTA
AAGTAAGGACCGTAGCTTGCCGGTCCCTGATATAACAAGGTTGAGAAACATTGAAGATTC
AATGTGTTCAATCTTTGTTAGTTTCAGGTTTTGTCAGGTTGTCTATGGGTCCTACACAGT
TCTGAAATCTCAATGCAACTGACAAAATCTGAACCAGTCCAGTTGAAATTTTTCTTCCAA
TCCATTTTTCCATGAACAGAACTGGATTCATCCATCCACATGATGTTTATAGTAAACCAT
GTCAACCTATTGATTTTGGTGGCTGGGAGCAGGGTGGCTGGGGAGTGTAATGGAAGGGTA
CAGACAGGATGAACATCCCAGGGGAGGTCAGGACTATTTAGAGATGATAATTGGAATTGA
GTGGATCTTACGCACACCCTGCGTCCCTTAACACCTCTGAGCTGACCGCTGGGAGGGAAA
GGAGCAATGGGGGCGAGGTGGGGTAGGGTGAGGGAGGGAGGGAGGTGGCGGGGGGCATTT
GAGGTGGGGACATAAATTGAGGAGGAAGAGGAGGCTTC**AGAGATATCATCAGAGAGCAGC**
TCTACAACACTCATCGTCGCCTCGGGAAAGTGATTTGACACTGAGTTGCTGTATACCAAC
AGGTTAGAAATTAATAGAAGTGAACAGTGCACATAGACAGACTTGGTGGACATCAACAGT
GATAACCGATTCCATACAACAAGTATTATCAGACAGGATCTAAGGTAAGGATTGCTATGA
TTTTTGCTATTGGGTATCAATGGGTTAAAAAGTGAGCTCTTTGAGAGAATTTTTGCATTT
GCTTTAGGTTTTATGCTTTCCATGAAGTATACATTAAACCCATAGACATATACATTTTTT
GGTTCCATTGTATGGTGGCAGTACCAGTATATATTTTGGAATTAGATCTTTAAAAAAAAA
AAATTACGCTTAACATTATTTTCAAGCCTGCCTCTCTAAGTGGAATCTTGAGAAGAAATT
ACCACAACAATTGCCTATGAGAAATGGATTTTTGAAACCCACTGTAAACGTACTGTTTTT
GTTTGTGGAGATTTTTTCCCCTGTGTATAAATATTATTAATGTATTGTATTAAATATTTA
TCCTGCATAAAATTAAGTTCTGAGCTGCCCACAAAAAAAAACTTTAAGGCTTGTTGCTTTT
TGCTGTTTGCTTCTCCAATCGTGCCTCTTCACCAAGATTTGAATAATTATCCTAATTTGC
TTGGCAGATCAGACGCCGCCATCGCACTTCTAACACCAATGTAGCGACCGTGTAGCAGTA
GGTACGGCCACAAGGGATGTTCAACCCCAGTTGTCTAACCCCAAGGCAAGCATAATCTCC
CTAGGCAGTCGGTTGCCTCCCACAATGTGCTACTGTTGCCTAGGGTCGGGTTTACGAGAC
TAAGGCAAGCATGCAGTCCCAAGAGAATTAACTCCCTTGAGAGCTCTAGGGTCATCACAG
TATTCTCCCCCTTCTCGCACTTTCTCTCGTTCCCGTGGCCTGAATTGTATAACGATCCCC
CATCCCTCCCTTTCTTTCCTTGTGAAAGGTGTGCAAGCCTGACAGGTGTCTCATGCTCTC
TTACCCACACCCTCCCTCTCATTACATCTCTCCAG**CTCCAGTGATG**GTGAGTGTGGCTAG
GCTGGTGTTGATGCTGGGGCTGCTGCTGTGTCTGGGAGCCCAGCTGTCCTCCTCC**CAACA**
CTGGTCCCATGGCTGGTACCCAGGAGGCAAGCGGGAACTGGACTCATTTACCACCTCTGA
G**GT**GGGTCACAGTACTACAATCACAATGACTCAAATGGATATTATGCATGTGTAAAATAC
ACAGTAGCCATTTGTGTCACCATCATTCTATTATCTCTCTCTCTCTCT**CAG**ATTTCAGAG
GAGATTAAACTCTGTGAGGCAGGAGAATGCAGCTACCTGAGACCACAGCGAAGGAACATC
CTTAGGAACATTCTT**GT**GAGTGCAGGGGCGCCCTTACGAAAAAATATTGCAGTCAGAGTG
CAGTATAACTGCAGTATGCAGCAAATACTACATCTAAAATAACACCGTTTTTTTTACTGC
AATAATTTTGCAAAGTAACTGCAGTTAGAGTGCAATATAACACTGCAGTTATTCTGCATC
CAAAATAACACAGTCGACTGCAGTTACTGCACTTTTACTGCAGTTTCAAAACTGCAATTT
ATTTGTGTAAGAACAGTAGTACATTAATGTAATGATAATGTATCAGGTGGCATTCACATA
GGCCAGGGATACAGTACCTCAAATCTGAGCCTGGGAGGCCGGAATACTGCTAGTTTAATT
TTCACTGATTGGCCACTCAGAGCCTGACAGCCATGTACCGTAGCCAACCATTGTTGAACA
TTGCAGATTTTAATCCTTATTCAATGTGTCAGAGAGGCATGTTTGTTGTACGTAATGTAT

TTCTATCTGAACATTGCATAATAATATGTTTCATTGTCACATACACCGGATAGGTGCAGT
TAAATGTGTTGTTTTCCATGGTCAGCCATAGTAGTACTGCACCCCTGGAGCAAATTAGGG
CTAAGTACCTTGTTCAAGGGCATATGGACATATTTTTCACCTTAACTGCTCAGGTATTTT
AACCAGTGACCTTTCAGTTACTGGTCCAATGCTCTAACTAACCGCTAGGCTACCTGCCAC
CCTTTAATGTTGTGCCCTGCTGAATGCAGCACTGGTGTCCCAGGTCTAAATCAGTCCCTG
ATTAAAGGAGAAGAATAAAAGCCCACTTGTGTGTGAGCATATGAGTGTATTGTATCTACA
TGTGTTACAAGCATGCTGCATAGAGGTGGAAAAGGGGGGGGTATATTGCACCTGTAATT
CATATGGTGTTACTCTCATTG**CAG**TTGGATGCCCTGGCCAGAGAATTTGAGAAGATAAAG
TAACCTATCAACTGAAGCTGACTTAAATCCTTTCTTGTCTATCTGTTTATTTCAAATGAC
CTCTATTCTAATCTTATGTCCTTTGATCCAATTTTATGGTTTATCAATTTTGAT**AATAA**T
GTCGCATCCCTTCTTTACCCTTGCACCCTCTTTATTGTCCCTTTAGACTAAATTACAGAT
GTAACATCATGCACATTCCATTTCCTGT**AATAAA**GTATCTATTTTGTTATTAATGTCTTC
ATCTTTGTCCTCTTCATTTAATTTGATCCAAAGAAAGAATAGGTATAGAAATATACTGAA
CAAAAATATAAACGTTTAATGCAAAGTGTTGGTCCCATGTTTCATGCCAAGATAATCCAT
CCACCTGACAGGTGTGGCATATCAAGAAGCTGATTAAACAGCGTTACACAGGTGCACCTT
GTGCTTGGGACAATAAAAGTCCACTCTAAAATGTGCCATTTTGTCACACATCACAATGCT
AGAGATGTAATTGGCATGCTGAAGGT

mRNA:

AGAGATATCATCAGAGAGCAGCTCTACAACACTCATCGTCGCCTCGGGAAAGTGATTTGA
CACTGAGTTGCTGTATACCAACAGGTTAGAAATTAATAGAAGTGAACAGTGCACATAGAC
AGACTTGGTGGACATCAACAGTGATAACCGATTCCATACAACAAGTATTATCAGACAGGA
TCTAAGCTCCAGTGATGGTGAGTGTGGCTAGGCTGGTGTTGATGCTGGGGCTGCTGCTGT
GTCTGGGAGCCCAGCTGTCCTCCTCC**CAACACTGGTCCCATGGCTGGTACCCAGGA**GGCA
AGCGGGAACTGGACTCATTTACCACCTCTGAGATTTCAGAGGAGATTAAACTCTGTGAGG
CAGGAGAATGCAGCTACCTGAGACCACAGCGAAGGAACATCCTTAGGAACATTCTTTTGG
ATGCCCTGGCCAGAGAATTTGAGAAGATAAAGTAACCTATCAACTGAAGCTGACTTAAAT
CCTTTCTTGTCTATCTGTTTATTTCAAATGACCTCTATTCTAATCTTATGTCCTTTGATC
CAATTTTATGGTTTATCAATTTTGAT**AATAA**TGTCGCATCCCTTCTTTACCCTTGCACCC
TCTTTATTGTCCCTTTAGACTAAATTACAGATGTAACATCATGCACATTCCATTTCCTGT
AATAAAGTATCTATTTTGTTATTAATGTC

protein:

MVSVARLVLMLGLLLCLGAQLSSS**QHWSHGWYPG**GKRELDSFTTSEISEEIKLCEAGECS
YLRPQRRNILRNILLDALAREFEKIK

Organism name: Salvelinus spp.

>gi|1340979802|ref|NC_036851.1|:41836621-41839584 Salvelinus spp. isolate IW2-2015 linkage group LG11, ASM291031v2, whole genome shotgun sequence

TATTTTAGCTTGCATGCCTGCTGGCAAGCAATTACTAAATGTATTGAATAAGACTCGCAT
TCCTTTCCATCTTTTACCCAGATTTTAGCAGAGATGCAAAGAATCATATTTATTTTCTTT
AAAAAAGAACCACCAGTCAGTAGGATAGACAGCTTTAGGTATGCTTAGATATGCAGAAAA

ATAAACATAAGCATAATGATTATGGCTCTAGTTGCAGGAAGAAAAAAAAATGCAGGTGTTT
CAAATTCTCCAACTTCCACCACCCAGCTATCCTTACGTACTTTGTGCCCCCACAGATTTT
TGGGGTGCATGACACCCCTTCTGGAAGGCCACTCTCAGTGCAGGATTTTGTTCCAGCACT
AACACACCTGATCCAACTAATCATGGTCTAATCTGAAGATCAGGATTAGTGATTAGCTAA
ATCAGGTGTGTTAGCGCTGGGCTGGAACAAAAGCCTGCACACCCTGCATAGCTCTCTCCG
GGACTGGAATTGTCCATCTCTGCGAGATCCAACTTGACGGTCCCACACCCTTGATAAGGT
CCAAGGCCCCTATTCACATCGTCTAAGAGTAGGAGTGCTGATCTAGGATCAGGTCCCCCC
TCTTCATTATGGTTTAAAAGGCAAAACTATCCTAGATTAACTGATCCTACATTCTACTCT
CAGACGTTTTGTGAATAAGTTATCCGCTTCCGTGGCGGGCCGTGTCTCAGGAAGGTTTGG
CAGGCGAGATGAATTCTCCTCTGTGACTCATGCAGCGCTCCGTCATGTCCTCCTGCGCCC
TCCTCGCATTGGCAGCCCCATGTCAGTCAGCAGATTAACTAAGTCAACGGCGAGAAGACA
TGCTGTCAATCAACCGCCAGATAGGAACTCCCCCTATGCATTTTAAACATGAATTTGCAG
TCCCCAATTGATACCCCCTCACCCGAGCCAAAAGGCCTGTCAGACTGGAATTAAGAGAGA
TGGTGGCGGCTGGTGTGTTTGCCGTGCTCGTGTTTGCTTGTCATCCGGTGTCCCCATGGT
CAAAGTGTTCTGGCGGTCACTCGGGTGTGAGGGAGGGTGCGAGACAGGCCACTGCACCAC
TTTAAGCAGGGGCAAATACAGAGGAAATTAGATTTGGCCTAGGTCAGTGATCCTCAACCC
CCAGTCTGCAGACCAACACTGGATCCTGGAATGTTGTTGACCGTCCCACAGACTGTTAGC
GAAATTACAGTTCTCGAGTGCTAGTTGTAGGTGTCCCTCTAAGCAGGAAGGACAACCGGT
TGCCAGTCCCTGGTAAAGTAAGGACGGTAGCTTGCCGGTCCCTGATATAACAAGGTTGAG
AAACATTGAAGATTCAATGTGTTCAATCTTTGTTAGTTTCAGGTTTTGTCAGGTTGTCTA
TGTGTCCTCCACAGTTCTGAAATCTCAACGCAACTGACAAAATCTGAACCAGTCCAGTTG
AAATTTGTCTTCCAATACATTTTTCCATGAACGTGATGTGTGAAGAAATGTAAGAACATC
ATGAAGTGACCGAACTTGATTCATCCAGCCACATGATGTTTATAGTAAACCATGTCAACC
TATTGATTTTGTTGGCTGGGAGCAGGTTGGCTGGGGAGTGTAATGGAAGGGGGTGGGTAT
TAGGTACAGACAGGATGAACATCCCAGGGGAGTCAGGACTATTTAGAGATGATAATTGGA
ATTGAGTGGATCTTACGCACACCCCGCTCGCTAAACACCTCTGAGCTGACCACTGGGAGG
GAAAGGAGCAATGGGGGCGAGGAGGGGTAGAGTGAGGGAGGGAGGTGGCGGGGGGCATGG
TGGGGACATAAATTGAGGAGGAAGAAGAGACTTC**AGAGATATCATCAGAGAGCAGCTCTA**
CAACACTCATCGTCGCCTTGGGAAAGTGATTTGATACTGAGTTGCTGTATACCAACAGCT
TAGAAAGTATAGAAGTGAACAGCGCACATAGACAGACTTGGTGGACATCAACAGTGATAA
CGATCCCATAGAACAAGTATTATCAGACTGAGGATCTAAGGTAAGGATTGCTACGATTTT
TGCTATTGGGTATCAATGGGTAAAAAGTGAGCTCTGAGAGAATTTTTGCATTTGCTTTAG
GTTTTACGCTTTCTATGAAGTATACATTAAACCCATAGACAGCTAAATGTTTTGGTTCCA
TTGTATGGTGAATGAATGGTGGCAGTACCAGTATATATTTTGGAATTAGATGTTTTAAAA
AAGAAACTAAAATTAAGCTTAACATGCTCTTTATTTTCAATCCTGCCTTTCTAAGTGGAA
TCCTGAGAAGAAATTCCCACAACAATTGCCTATGAGAAACGGATCTTTGAAACCCACTGT
AAACGTACTGTTTTTGTTTGTGGAGATTTTTTTCCCTGTGTATAAATATTAATAATGTAT
TGTATTAAATATTTATCCTGCATAAAAATAAGTTATGAGCTGCACACCAAAAAAACTTTA
AGGCTTGTTGTTAAGGCTTGTTGCTTTTTGCTCTTTGCTTCTCCAATCGTGCTCTTCACC
AAGATTTGAATAATTATCCTCATTTGCCTGGCAGATCAGACGCCGCCATCACACTTCTAA
CAACAATGTAGCGACCATGTAGCAGTAGGTACGGCCACAGGGATGTTCAACCCCAGTTGT
CTAACCCCAAGGCAAGCATAGTCTCCCTAGGCAGTCGGTTGCCTCCCACAATGTATCCAA
TGCTACTGTTGCCTAGGGTCGGGTTATGAGACTAAGCAAGCATGCAGTCCCAAGACAATT
AACTCCCTTGAGAGCTCTAGGGTCATCACAGTATTCTCCCCCTTCTCGCACATTCTCTCG
TTCCCGTGGCTGAATTGTATAACGATCCCCCATCCCTCCCTTTCTTTCCTTGTGAAAGGT
GTGCAAGCCTGACAGGTGTCTCATGCTCTCTTACCCACACCCTCCCTCTCCTTACATCTC
TCCAG**CTCCAGTGATG**GTGAGTGTGGCTAGGCTGGTGTTGATGATGGGGCTGCTGCTGTG
TCTGGGAGCCCAGCTGTCCTCCTCC**CAACACTGGTCCCATGGCTGGTACCCAGGA**GGCAA

GCGGGAACTGGACTCATTTACCACCTCTGAG**GT**GGGTCACAGTACTACAATCACAATGAC
TCAAATGGATATTATGCATGTGTAAAATACACAGTAGCCATTTGTGTCACCATCATTCTA
TTATCTCTCTCT**CAG**ATTTCAGAGGAGATTAAACTCTGTGAGGCAGGAGAATGCAGCTAC
CTGAGACCACAGCGAAGGAACATCCTTAGGAACATTCTT**GT**GAGTGCAGGGGCACTCTTA
CGAAAAAAAATTGCAATCAGAGTGCAGTATAACTGCAGTATGCAGCAAATACTACATCCA
AAATAACACCGTTTTTTTTACTGCAGTCATTTTGCAGTGTAACTGCAGTTAGAGTGCAAT
ATAATACTGCAGTTATTCTGCATCCAAAATAACACAGTCGACTGCAGTTACTGCACTTTT
ACTGCAATTTCAAAACTGCAATCTATTTGTGTAAGGGCAGGAAGGCAGTAGTACATTCAT
GTAATGATAATGTATCAGGGGGGGTATAGTGCACCTGTAATTCATATGGTGTTACTCTCT
TTG**CAG**TTGGATGCCCTGGCCAGAGAATTCGAGAAGATAAAGTAACCTAAGCTGACTTAA
ATCCTTCCTTGTCTATCTGTTTATTTAAATGACCTCTATTCTAATCTTATGTCCTTTGAT
CCAATTTTATTGTTTATCAATTTTGAT**AATAA**TGTCGCATCCCTCCTTTACCTTGCACCC
TCTTTATTGTCCCTTTAGACTCAATTACAGATGTAACATCATGCACATGCCATTTCCTGT
AATAAAGTATCTATTTTGTTATTAATGTCTTCATCTTTGTCCTCTTCATTTAATTTGATC
CAAAGAAAGAATAGGTATAGAAATATACTGAACAAAAATATAAACGTATAATGCAAAGTG
TTGGTCCCATGTTTCATGAGCTGAAATGCCATACATTTTCCACACGCACAAAATGTTATT
TC

mRNA:

**AGAGATATCATCAGAGAGCAGCTCTACAACACTCATCGTCGCCTTGGGAAAGTGATTTGA
TACTGAGTTGCTGTATACCAACAGCTTAGAAAGTATAGAAGTGAACAGCGCACATAGACA
GACTTGGTGGACATCAACAGTGATAACGATCCCATAGAACAAGTATTATCAGACTGAGGA
TCTAAGCTCCAGTG**ATGGTGAGTGTGGCTAGGCTGGTGTTGATGATGGGGCTGCTGCTGT
GTCTGGGAGCCCAGCTGTCCTCCTCC**CAACACTGGTCCCATGGCTGGTACCCAGGA**GGCA
AGCGGGAACTGGACTCATTTACCACCTCTGAGATTTCAGAGGAGATTAAACTCTGTGAGG
CAGGAGAATGCAGCTACCTGAGACCACAGCGAAGGAACATCCTTAGGAACATTCTTTTGG
ATGCCCTGGCCAGAGAATTCGAGAAGATAAAGTAACCTAAGCTGACTTAAATCCTTCCTT
GTCTATCTGTTTATTTAAATGACCTCTATTCTAATCTTATGTCCTTTGATCCAATTTTAT
TGTTTATCAATTTTGAT**AATAA**TGTCGCATCCCTCCTTTACCTTGCACCCTCTTTATTGT
CCCTTTAGACTCAATTACAGATGTAACATCATGCACATGCCATTTCCTGT**AATAAA**GTAT
CTATTTTGTTATTAATGTC

protein:

MVSVARLVLMMGLLLCLGAQLSSS**QHWSHGWYPG**GKRELDSFTTSEISEEIKLCEAGECS
YLRPQRRNILRNILLDALAREFEKIK

Organism name: Oncorhynchus mykiss (rainbow trout)

>gi|1207596268|ref|NC_035083.1|:64441322-64444570
Oncorhynchus mykiss isolate Swanson chromosome 7, Omyk_1.0, whole genome shotgun sequence

CTGGCGACCCCACCTCTTTAACCTGTCTCTCTGGTGACCCCACCTCTTTAACCTGTCTCT
CTTTGGCGACCCCACCTCTTTAACCTGTCTCTCTCCTCCTGATCCTGTTTAGCTGTCCCT
TGGATGTCTCTCTCTCTCTCTGTCGGTCTGTCTATTTCACTCTGTCGGTCTGTCTCTTTC

TCTCTCCTCTCTCTCCTTTCTTTCTTTCTTTCTTTCCTTCCTTCCCTTAATTAACTCATA
TTTCCTTCCTCTCTTGTTCAATTTGCCCCTTGGGCTGGATTTGTCTCATCGATAACCGGT
CACTGCCCCATTCCCTTCTGTACATAATGTAGGGTTATTTATATTTAATTCCTGTCTGCT
GCCTTGCTTTTTTTTCTTTGCCTTTTTTAAGAAAATGTACATGAGCTGTACTGGGACAAT
TTAGAAATGTATTAGATATTTTGTTTTTCTTGCTATTGTTTTTATTTGTTATTATTATTG
TCTTAAGAAAATCCTATTTTTTATTTGCATCTGTTTTGTTTTCTAGTGTTTTATTAAAAT
GTTGAATATATGTATATATAATTATATATACGATAAAATGATATATAATTATATAATGCC
AAATGGCCTTTCTAAGCAAGATATTGTTTAAACCTAATAAAATGCTTGAGATTTTGTGTT
ATTTGTTTGTTAGCTACAGCATTATCTGATGACATGTGCAATAGTTACAAATGTTCAGTG
TTAACACCAATCCCAACATCAATACTATAATCTTCAATACTGGAATTGAACCAAGTTCTC
TAACCTGCATTCTGTTCAGTAGGGCAATCCACAGCTAAACTTTTTATAATGGAAAATGGA
AATCTGTGTTTTGATAAGTAATGTTACAGGCTTCTCCTTTTTAATTCCATTCCGAAACGT
TTTTTCCCCACTGAACACAACCCTCTTCTGAGACTATTTACACTGCATGATGATGATTAC
GTGCCCCAATGCACATCCAGGTCATGCACTGTTATTGAGTTTGTCTGGACTGGCTTTTCA
GTTAAGAGTTTAACCCTTACACTCAATCAAATTTGTATTTTTTTTATATATTTTTTTATT
GAACCTTTATTTAACTAGGCAAGTCAGTTAAGAACAAATTCTTATTTACAATGACGGCCT
ACCAAAAGGCCGCAGGGGCGGGGGCTGGGATAAAAAATATAGGACAAAACACACATTACA
TTACACACTGCAGCAGGGGTTGTGGTGGAATGTGTGTGAAGCAATATGTTTATTTGTATT
TGAACATTATAGTAGTCCATAATAGTTGAGGCTGATGAGGGGAAGACTTCACCTAATAAT
AGCTGGAACAGAGTGAATGGAATGGCATCAAACACATGGAAACCATGTGTTTTATGTATT
TGATACCATTCCACTAATTCCGCTCAAGCCATTACCACGAGCCTATCCTCCCAAATGAAG
ATTCCACCAACCTCCTGTGGTCCATAACCAGATAATACATTATCTAGAATCTACAGCTTG
GAGTAAGCACTTGATTGAAGCTACTCCTGCAGGAATGGGAGGATTCTGGACCTGGAAGCA
GAGTGTCATGCCCACAGGGGCAGGTGCCCCATCATATTTTTAAACAGGACAAATCTATGT
TTTGATTTTGTTTCTCTCTAATACTACTAGCCACCTAGCAATTTTATGATGTTGGCTTTA
GCGAGCCCAGATAGGTTCCCAATCTCCTAACCTCATAACTAGCTACCAAGCAGCCATTTC
ATGCTATCAAGTTAGAGTAGCTAGCTTGTCTATTTTAGCTGGCATGCCTGCTGGTAAGCA
ATTGCTACATGTATTGAATAAGACTTGCATTCCTTTCCATCTCTTACCCAGATTTGAGCA
GAGATGCAAAGAATCATATTTATTTTCTTTAAAAAAATAACCAGTCAGTAAGATAGACAG
CTCGAGGTATGCTTAGATATGCAGAAAAATAAACATATAAGCATAATGAGTATGGCTCTA
GGTTGCAGGAAGAAAAACATTTCAGGTGTTAGAAAATTTCCAAATTGTCCAACTTCCACC
ACCCAGCTATCCTAACGTACTTTGTGCCCCCACAGATTTTTGGGGTTCATGACGCCCCTT
CCTGGAAGTCCACTCAGTGCAGGATTTTGTTCCAGCACTAACACACCTGATCCAACTAAT
CATGGTCTAATCTGAAGATCAGTATTAGTGATTAGCTAAATCAGGTGTGTTAGTGCTGGG
CTGGAACAAAAGCCTGCACAATCTGCTATAGCTCTCTCCAGGACTGCAATTTTCCATCTC
TGCAAGATCCAAATTGACGGTCCCACACCCTTGATAAGATCCAAGGACCCTATTCACTTC
GTCTAAGAGTAGGAGTGCTGATCTAGGATCAGGTCCCCCCTCTTCATTATGGTTTAAAAG
GCTAAATTGATCCTAGATTAACTGATCCTACATTCTACTCTAAGACGTTTTGTGGATACG
GCCCAAGTTCTCCGCTTCCGTTGGCGGGCCGCGTCTCAGGAAGGTTTGGCAGGCAAGATG
AATTCTCCTCGGTGACTCATGCAGCGTTCCGTCATGTCCTCCTGCTTCCTGCGCCCTCCT
CGCGCTGGTAGCCCCGTGTCAGTCAGGAGATTAACTAAGTCAACAGCGAGAAGACATGCT
GTCAATCAACCGCCAGATAGGAACTCCCCCTATGCATTTTAAACATTAAAATTTGCCAGT
CCCCAATTGACACCCCCTCACCCATGCCAAAAGGCCTGTCAGACTGGAATTAAGAGAGAT
GGTGGCAGCTGGTGTGTTTGCCCGTGCTCGTGTTTGCTTGTCATCCAGTGTCCCCATGGT
CAAAGTGATCTGGCGGTGACTCGGGTGTGATGGAGGGTTTGAGACAGGCCACTGCACCAC
TTTAAGCAGGGGAAAATACAGAGGAAATGAGATTTTGCCTAGGTCAGTGATCCTCAACCC
CGGTCTGCAGACCAACACAGGATCCTGGAATGTTGTTGACCCACAGACTGTTAGCGAAAT
TACAGCTCTCAAGTGCTAGTTGTAGGTGTCCCTCTAAGCAGGAAGGACAACCGGTTGCCA

GTCCTTGGTAAAGTAAGGACCGTAGCATGCCGGTCCCTGACATAACAAGGTTGAGAAACA
TTGAAGATGTGTTCAATCTTTGTTAGTTTCATGTTTTGTCAGGTTGTCTATGTGTCCTCC
ACAGTTCTGAAATCTCAACGCAACTGACCAAATCTGAACCAGTCCAGTTGAAATTTGTCT
TCCAATACATTTTTCCATGAACGTGATGTGTGAAGAAATGTAAGAACATCATGAAGTGAC
AGATCTGGATTCATCCAGCCACAGGATGTTTATAGTAAACCATGTCAACCTATTGATTTT
GGTGGCTGGGAGCAGGGTGGCTGGGGAGTGTAATGGAAGGGGGTGGGTATTAGGTACAGA
CAGGATGAACATCCCAGGGGAGGTCAGGACTATTTAGAGATGATAATTGGAATTGAGTGG
ATCTTACGCACACCCCGCGTCCCTAAACACCTCTGAGCTGACTGCTGGGAGGGAAAGGAG
CAATGAGGACGAGGTGGGGTAGGGTGAGGGAGGTGAGGTGGCGGGGGGCATTTGAGGTGG
GGACATAAATTGAGGAGGAAGAGGAGGCTTC**AGAGATGTCATCAGAAAGCAGCTCTACAA**
CACTCATCGTCGCCTTGGGAAAGGGATTTGACACTGAGTTGCTGTATACCAACAGGTTTG
AAAGTAATAGAAGTGAGCAGCGCACATAGACTGACTTGGTGGACATCAACAGTGATAACC
GATTCCATACAACAAGTATTATCAGACTGAGGATCTACGGTAAGAATTGCTATGATTTTT
GCTATTGGATATCAATAGGTAAAAAGTGAGCTCTGAGAGAATTTTTGCATTTGCTTTAGG
TTTTATGCTTTCTATGAAGTATACATTAAACCCATAGATAGCTACATTTTTTGGTTCCAT
TGTATGGTGAATGAATGGTGGCAGTACCAGTATATATTTAGGAATTAACAACCTAAAATT
ACACTTAATAAGCTCTTCATTTTCAATCCTGCCTTTCTAAGTGGGATCTTGAGAAGAAAT
TACCACAACAATTGCCTATGAGAAACGGATCTTTGAAGCCCACTGTAAACGTACTGTTTT
TGTTTGTGGAGATTTTTCCCTGTGTATAAATATTATTAATATATTGTATTAAATATTTAT
CCTGCATAAAATTAAGTTATGAGCTGAACACAAAAAAAAACGTTCAGGCTTATTGCTTTTT
GTTTCTCCAATTGTGCCTCTTCACCAATATTTGAATAATTACCCTCATTTGCCTGGCAGA
TCAGACGCCGCCATCACACTTCTAACACCAATGTAGCGACCATGTAGCAGTAGGTATGGC
CACATCGGGATGTTCAACCCCAGTTGTCTAACCCCAAGGCAAGCATAGTCTCCCTAGGCA
GTCAGTTGCCTCCCACAATGTATCCAATGCTACTGTTGCCTAGGGTCGGGTTTACGAGAC
TAAGGCAAGCATGCAATCCCAAGAGAATGAACTCTCTAGAGAGCTCTAGCGTCATCACAG
TATTCTCCCCCTTCTCGCACTTTCTCTCGTTCCCGTGGCGTGAATTGTATAACGATCCCC
CATCCCACCCTTTCTTTCCTTGTGAAAGGTGTGCAAGCCTGACAGGTGTCTCATGCTCTC
TTACCCACACCCTCCCTTTCCTTACATCTCTCCAG**CTCCAGTGATG**GTGAGTGTGGCTAG
GCTGGTGTTGATGCTGGGGCTGCTGCTGTGTCTGGGAGCCCAGCTGTCCTTCTCC**CAACA**
CTGGTCCCATGGCTGGTACCCAGGAGGCAAGCGGGAACTGGACTCATTTACCACCTCTGA
G**GT**GGGTCACAGTACTACAATCACAATGACTCAAATGGATATTATGCATGTGTAAAATAC
ACAGTAGCCATTTGTGTCACCATCATTCTATTATCTCTCTCTCTCTCTCT**CAG**ATTTCAG
AGGAGATTAAACTCTGTGAGGCAGGAGAATGCAGCTACCTGAGACCACAGCGAAGGAACA
TCCTTAGGGACATTCTT**GT**GAGTGCAGGGGCGCCCTTATGAAAAATATTGCAGTCAGAGT
GCAGTATAACTGCAGTATGCAGCAAATACTACATCCAAAATAACACCGTTTTTTTTTACT
GCAGTAATTTTGCAGTGTAACTGCAGTTAGAGTGCAATATAATACTGCAGTTATGCTGCA
TCCAAAATAACACAGTCGATGTATCAGGTGGCATTCACATAGGCCAGGGATACAGTACCT
CAAATCTGAGCCTGGGAGGCCGGAATACTGATAGTTTAATTTTCAATGATTGGCCACTCA
GAGCCTGAGAGCCATGTTCCGTAGCCAACCGTTGTGGAACATTGCAGATTCTAATCCTTA
TTCAATGTGTCAGAGAGGCATGTTTGTTGTACATAATGTATTTTTATATGAACGTTCCAT
AATAATGTTTCATTGTCACATACACCGGATAGGTGCAGTTAAATGTGTTGTTTTCAAGGG
CATATGGACATATTTTTCACCTTAACTGCTCAGGTATTTTAACCAGTTACTGGTCCAATG
CTCTAACTAACTGCTAGGCTACCTGCCACCCTTTAATGTTGTGCCCTGCTGAATGCAGCA
CTGGTGTCCCAGGTCTAAATCAGTCCCTGATTAAAGGAGAAGAATAAAAACCCACTTGTA
TGTGAGCATAGGAGTGTATTGTATCTACATGTGTTACAAGCATGCTGCATAGAGGTGGAA
ATGGGGGNNNNNNNNNNNNNNNNNNNNNNNNNNNNNNNNNNNCTGTCTAATAGACAGGTTA
GTACAGCCTATATCATTCTGTCTAATAGACAGGTTAGTACAGCCTATCTCATTCTGTCTT
ATAGACAGGTTAGTACAGCCTATGTCACACTGTCTAATAGACAGGTTAGTACTGCCTAAG

CACACTTTCAAATAGGATGGTTAGTACAGCCTATATCACTCTGTAATAGCAAGGTCAGTA
CAGCCTATATCACACTGTCTAATAGACAGGTTAGTACTGCCTATATCACACTGTCTGATA
TACAGGTTAGTATAGCCTATATCACGCTGTAATTGACAGGTTAGTAATGCCTATATCACA
CTGTCTAATAGACAGGTTAGTACNNNNNNNNNNNNNNNNNNNGTAATTCATATGGTGTTA
CTCTCTTCG**CAG**TTGGATGCCCTGGCCAGAGAATTCGAGAAGATAAAGTAACCTTACAAC
TGAAGCTGACTTAAATCCTTCCTTGTCTATCTGTTTATTTCAAATAACCGCTATTCTAAT
CTTATGTCCTTTGATCCAATTTGATGGTTTATCAATTTTGAT**AATA**TGTCACATCCCTCC
TTTACCCTTGCACCCCCTTTATTGTCCCTTTGGACTCAATTACAGATGTAGCATCATGCA
CAAGCCATTTCCTGT**AATAAA**GTATCTATTTTGTTATTAATGTCTTCATCTTTGTCCTCT
TCATTTAATTTGATCCAAAGAAAGAATAGGTATAGAAATATACTGAACAAAAATATAAAC
GTATAATGCAAAGTGTTGGTCCCATGTTTCATGAGCTGAAATACCATACATTTTCCACAC
GCACAAAAATGTTATTTCTCTAAAATGTTGTACACAAATTTGTTTACATCCCTGTTAGTG
AGAATTTCTCCTTTGCCAACATAATCCATCCACCTGACAGGTGTGGCATATCATGAAGCT
GATTAAACAGTGTTACACAGGTGCACCTTGTGCTTGGAAAAATAAAAGTCCACTCCAAAA
TGTGC

mRNA:

AGAGATGTCATCAGAAAGCAGCTCTACAACACTCATCGTCGCCTTGGGAAAGGGATTTGA
CACTGAGTTGCTGTATACCAACAGGTTTGAAAGTAATAGAAGTGAGCAGCGCACATAGAC
TGACTTGGTGGACATCAACAGTGATAACCGATTCCATACAACAAGTATTATCAGACTGAG
GATCTACGCTCCAGTGATGGTGAGTGTGGCTAGGCTGGTGTTGATGCTGGGGCTGCTGCT
GTGTCTGGGAGCCCAGCTGTCCTTCTCC**CAACACTGGTCCCATGGCTGGTACCCAGGA**GG
CAAGCGGGAACTGGACTCATTTACCACCTCTGAGATTTCAGAGGAGATTAAACTCTGTGA
GGCAGGAGAATGCAGCTACCTGAGACCACAGCGAAGGAACATCCTTAGGGACATTCTTTT
GGATGCCCTGGCCAGAGAATTCGAGAAGATAAAGTAACCTTACAACTGAAGCTGACTTAA
ATCCTTCCTTGTCTATCTGTTTATTTCAAATAACCGCTATTCTAATCTTATGTCCTTTGA
TCCAATTTGATGGTTTATCAATTTTGAT**AATA**TGTCACATCCCTCCTTTACCCTTGCACC
CCCTTTATTGTCCCTTTGGACTCAATTACAGATGTAGCATCATGCACAAGCCATTTCCTG
T**AATAAA**GTATCTATTTTGTTATTAATGTC

protein:

MVSVARLVLMLGLLLCLGAQLSFS**QHWSHGWYPG**GKRELDSFTTSEISEEIKLCEAGECS
YLRPQRRNILRDILLDALAREFEKIK

Organism name: Oncorhynchus nerka (sockeye salmon)

>gi|1681300741|ref|NC_042536.1|:54369680-54373159
Oncorhynchus nerka isolate On170113-E2 linkage group LG2,
Oner_1.0, whole genome shotgun sequence

CCCCTGGTCCGATAGCCAATCACAGCTACTAATTGCCCTCAAAGGATTGTAATAAACAGC
GTGTGCGTAAGAGATTCTCACCCACTTCGCCACAAACCCAACCTCCCCACTCACTTGATA
TCTGCAAGGTTTGAGTGTATGAGGGGTAACTGACATGAGGTTTTAGCTGTCAATTGTGAT
CAGGGATTATTGGTCAATTATAAGAGCTGCCATTTTGATCCCAGTTTCTTCATTGGTCTG

TGGAGTTGACAGCACAGGAGGCTGCTGATGGGAGGAGGGCTCATAATAATGGCCGGAATG
GAGCAAATGGAACGGCATCAAACACCTGTGAACCATGTATTAAATACCATTCCCCCAATT
CCGCTCCAGCCATTCCCACGAGCCCGTCCTCCCCAATTTAGGTGCCACCAACCTCCTGTG
GTTGATGGGTATGCTAGACAGCCATGAAGATCTGCCAAGCCAAGTTGATGCCAAACGCAT
TCTCTTATACACAGATGGCCTATCTGTCCGGTCATAAAGGTGAACCGTTGTTTTGTCATT
AGGATATAAGGAAAGTCCACAGTCTGTAGAAAAATACCTGCACAACCTATACTGTTCAGG
AGTTAGAATGGATTGCTTTAGCTTCTCCCTTTTCTATATTGTTATAGGTCGAAAGGAAGG
AGCACAATTCAACTCCGTCGTGATTGAATCATATGGGATCAGGAAATTAGAAATAGTATC
TGAAACTCTACCAAAAAAAGCACCAGTATTGACAAATTGTTGCTATCGTTGATTGTTCGA
ATGCTTGTACTATTTGCGCCAAAATAAAAATCCGAACCGGTGATGCAAAGGCCAGATGGG
TAACATGACCCTGAGATAGTAAATGTGAGGATTCCCTCACAGCTTATTCCCTTCTGATTA
CAGGATTTCTGGGGGGAAAAAAACAGATTTTAGTCTTTAAAAAAAATATTTTGCAACCCT
GATGTCAGCATTGAAGAGGCCTTTTTAAAGACTGGTTATGGCCTCTAGTTCTGACTAGTT
CTATTAGGTTAAACCACAGGACATAGATAGCTAGGTAGAGATCTTCAAACCGGATGTCTC
ACTTAGACCAGTCACTTTAAAAAATACTGTCAATGTCATTTCAGACCGTGTTCCATTCCA
AAGCATCTCAAAGGCCCGTGTGTTACAATTGGTGCCATTTTATTTTCCTTCCGGATGTTT
GGTTGTGTTGGATCTTAGTAATCGTCAGGGTGGGCGTTGGGGAGGAGAGCCTTTAAGTCT
TCTTTTGAGCCAGAAGGTTTGCTGAATGTAATCACCTGTCCCCAACAACACTGTGCCAAC
CCCCAACCCTAGTGACTTAAGGCTTTACTCTAGAAAGTGCCTCACAGGTTGATTTATATA
GAAAATATGTTCACTGCACCTCTCCCTCAGTGAGTTGATTGAGTGTAGGTGTATAGACTC
TTGGTAAACCAGTATCATCCTACAGATTAGTACATCTATTTGGCCCCTTCCCACTACTAT
TTTTTAGCCATTGTAGATAACTTTATCGACAAAGCACCTCTGTACAAAGCCCCAAAGCTG
TAGCTGACCCTTGGCTTTGTAACACTGAGACCAAGATCAGGGCCTTTTATTATCCTGTTC
AAGTTCATAATCCACCAGCCTCCTTTCCATTTCATGCTGTAACGCCGGGTTCAATTCCAA
TCAAACTCCATTGCTTTGTTTTGAACCAGCTTTGTCTTGGCTCCATGTCTTCATTCCATT
CCACTGAAGTGCTGGACCCTTGACTATTCCTTCATTATGTCCCCTAGGCAGGTCCTCTAC
CTAACCTTCACCTACTATAGTGGGTAAAATACATTGCCCTGAGTTATTCCAACCACAAAT
CACAATGACATTGTTTACTGGTTATTACAGAGGACAGCCAGGTGCATCCGGGCCCTTGTT
CCACACCCCTGCTGGCTGACGATAGAAATGTGATGCAACATCCCAGTAATGTAATGTCCC
TGTCTGAGCAACAGGGCACACCTTCCTTCAGAGATGGAGAAAGAGAGCTAGACAGAGTGG
GGCTTCTTAGGGTTCAACCAGTCTCTCTGTCTCTCCCACATCTAAACCTGTCTCTCCCAG
CCTTAAACCAGTCTCTCTTGCCCACCCCTAAAACTCTCTCACCCCTAAACCTGTCTCTCT
GGCGACCCCACCTCTTTAACCTGTCTCTCTGGCGACCCCACCTCTTTAACCTGTCTCTCT
CTGGCGACCCCACCTCTTTAACCTGTCTCTCTGGCGACCCCACCTCTTTAACCTGTCTCT
CTCTAGCGACCCCACCTCTTTAACCTGTCTCTCTCTGGCGACCCCACCTCTTTAACCTGT
CTCTCTCTGGCGACCCCACCTCTTTAACCTGTCTCTCTGGCGACCCCACCTCTTTAACCT
GTCTCTCTGGCGACCCCACCTCTTAAACATGTCTCTCTGGCGACCCCACCTCTTTAACCT
GTCTCTCTGGCGACCCCACCTCTTTAACCTGTCTCTCTATAGCGACCCCACCTCTTAAAC
CTGTCTCTCTCTGGCGATCCCACCTCTTAAACCTGTCTCTCTCTGGCGACCCCACCTCTT
AAACCTGTCTCTCTCTGGCGACCCCACCTCTTTAACCTGTCTCTCTCTAGGGACCCCACC
TCTTTAACCTGTCTCTCTCCTCCTGATCCTGTTTAGCTGTCCCTTGGATGTCTGTCTCTC
TGTCTATTTCACTCTGTAGGTCTGTCTCTTTCTCTCTCCTCTCTCTCCTTTCTTTCTTTT
TTTCTTTCCTTCCTTCCCTTAATTAACTCATATTTCCTTCCTCTCTTGTTCAATTTGCCC
CTTGGGCTGGATTTGTCTCATCGATAACCGGTCACTGCCCCATTCCCTTCTGTACATAAT
GTAGGGTTATTTATATTTAATTCCTGTCTGCTGCCTTGCTTTTTTTTCTTTGCCTTTTTT
AAGAAAATGTACATGAGCTGTACTGGGACAATTTAGAAATGTATTACATAATTTGTTTTT
CTTGCTATTGTTTTTATTTGTTATTATTATTGTCTTAAGAAAATCCTATTTTTTATTTGC
ATCTGTTTTGTTTTCTAGTGTTTTATTAAAATGTTGAATATATGTATATATAATTATATA

TATGATAAAATGATATATAATTATATAATGCCAAATGGCCTTTCTAAGCAAGATATTGTT
TAAACCTAATAAAATGCTTGAGATTTTGTGTTATTTGTTTGTAAGCTACAACATTATCTG
AGTACATTTGCAATAGTTACAAATGTTCAGTGTTAACACCAATCCCAACATCAATACTAT
AATCTTCAATACTGGAATTGAACCAAGTTCTCTAACCTGCATTCTGTTCAGTAGGGCAAT
CCACAGCTAAAATGTTATAATGGAAAATGGAAATCTGTGTTTTGATAAGTAACGTTACAG
GCCTCTCCTTTTTAATTCCATTTCGAAACGTTTTCTCCCCACTGAATACAACCCTCTTCT
GATACTATTTACACTGCATGATGATGATTATGTGCCCCAATGCACATCCAGGTCATGCAC
TGTTATTGAGTTTGTCTGGACTGGCTTTTCAGTTAAGAGTTTAACCCTTACCCTCAATCA
AATTTTTCTTTTTTTATATATTTTTTATTTATCCTTTATTTAGCGAGATAACGGTATGAG
CGCATGGATGTTCTTCACAGCCACACTTACATCTATCAACAGAAGCGCAAAGAGGCGGGG
ACCCGCCTGAGCGCCTGCAATAGGCTGAGCAAGATTCGAATTCTTATTTACAATGACGGC
CTACCAAAAGGCAAAGGCCTCCTGCAGGGATGGGGGCTGGGATTAAAAATATAGGACAAA
ACACCCATTACATTACACACTGCAGTAGGGGTTGTGGTGAAATGTGTGTGAAGCAATATG
TTTATTTGTATTTGAACATTATAGTAGTCCATAATAGTTGAGGCTGCTGAGGGGAAGAAT
TCACCTAATAATGGCTGGAACAGTGAATGGAATGGCATCAAACACATGGAAACCATGTGT
TTTATGTATTTGATACCATTCCACTAATTCCGCTCAAGCCATTACCACGAGCCCATCCTC
CCAAATGAAGATTCCACCAACCTCCTGTGGTCCATAACCAGATAATACATTATCTAGAAT
CTACAGCTTGGAGTAAGCACTTCATTGAAGCTACTCCTGCAGGAATGGGAGGATTCTGGA
CCTGGAAGCAGAGTGTCATGCCCCATCATATTTTTAAACAGGACAAATCTATGTTTTGAT
TTTGTTTCTCGCTAATACTACTAGCCACCTAGCAATTTTATGATGTTGGCTTTAGCGAGC
CCAGATAGGTTCCCAATCTCCTAACCTATAACTAGCTACCAAGCAGCCATTTCATGCTAT
CAAGTTAGAGTAGCTAGCTTGTCTATTTTAGCTGGCATGCCTGCTGGCAAGCAATTGCTA
CATGTATTGAATAAGACTTGCATTCCTTTCCATCTCTTATCCAGATTTTAGCAGAGATGC
AAAGAATCATATTTCTTTTCTTTAAAAAAAAATAACCAGTCAGTAAGATAGACAGCTCGAG
GTATGCTTAGATATGCAGAAAAATAAACATATAAGCATAATGATTATGGCTCTAGGTTGC
AGGAAGAAAAACATTTCAGGTGTTAGAAAATTTCCAAATTGTCCAACTTCACCACCCAGC
TATCCTAACGTACTTTGTGCCCCCACAGATTTTTGGGGTTCATGACGCCCCTTCCTGGAA
GTCCACTCAGTGCAGGATTTTGTTCCAGCACTAACACACCTGATCCAACTAATCATGGTC
TAATCTGAAGATCAGGATTAGTGATTAGCTAAATCAGGTGTGTTAGTGCTGGGGTGGAAC
AAAAGCCTGCACAATCTGCTATAGCTCTCTCCAGGACTGCAATTTTCCATCTCTGCGAGA
TCCAAATTGACGGTCCCACACCCTTGATAAGATCCAAGGTCCCTATTCACATCGTCTAAG
AGTAGGAGTGCTGATCTAGGATCAGGTCCCCCCTCTTCATTATGGTTTAAAAGGCTAAAT
TGATCCTAGATTAACTGATCCTACATTCTACTCTAAAGACGTTTTGTGGATACGGCCCAA
GTTCTCCGCTTCCGTTGGCGGGCCGTGTCTCAGGAAGGTTTGGCAGGCGAGATGAATTCT
CCTAGGTGACTCATGCAGCGTTCCGTCATGTCCTCCTGCTTCCTGCGCCCTCCTCGCGCT
GGTAGCCCCATGTCAGTCAGCAGATTAACTAAGTCAACAGCGAGAAGACATGCTGTCAAT
CAACCGCCAGATAGGAACTCCCCCTATGCATTTTAAACATGAAAATGTGCCAGTCCCCAA
TTAATACCCCCTCACCCAAGCCAAAAGGCCTGTCAGACTGGAATTAAGAGAGATGGTGGC
AGCTGGTGTGTTTGCCCGTGCTCGTGTTTGCTTGTCATCCAGTGTCCCCATGGTCAAAGT
GTTCTGGCGGTCACTCGGGTGTGATGGAGGGTTTGAGACAGGCCACTGCACCACTTTAAG
GAGGGGAAAATACAGAGGAAATGAGATTTTGCCTAGGTCCGTGATCCTCAACCCTGGTCT
GCAGACCAACACAGGATCCTGGAATGTTGTTGACCCACAGACTGTTAGCGAAATTACAGC
TCTCAAGTGCTAGTTGTAGGTGTCCCTCTAAGCGGGAAGGACAACCGGTTGCCAGTCCTT
GGTAAAGTAAGGACCGTAGCATGCCGGTCCCTGATATAACAAGGTTGAGAAACATTGAAG
ATTCAATGTGTTCAATATTTGTTAGTTTCAGGTTTTGTCAGGTTGTCTATGTGTCCTCCA
CAGTTCTGAAATCTCAACGCAACTGACAAAATCTGAACCAGTCCAGTTGAAATTTGTCTT
CCAATACATTTTTCCATGAACGTGATGTGTGAAGAAATGTAAGAACATCATGAAGTGACA
GAACTGGATTCATCCAGCCACAGGATGTTTATAGTAAACCATGTCAACCTATTGATTTTG

GTGGCTGGGAGCAGGGTGGCTGGGGAGTGTAATGGAAGGGGGTGGGTATTAGGCACAGAC
AGGATGAACATCCCAGGGGAGGTCAGGACTATTTAGAGATGATAATTGGAATTGAGTGGA
TCTTACGCACACCCCGCGTCCCTAAACACCTCTGAGCTGACCGCTGGGAGGGAAAGGAGC
AATGAGGACGAGGTGAGGGAGGGAGGTGGCGGGGGGCATTTGAGGTGGGGACATAAATAG
AGGAGGCTTC**AGAGATATCATCAGAAAGCAGCTCTACAACACTCATCGTCGCCTTGGGAA**
AGTGATTTGACACTGAGTTGCTGTATACCAACAGGTTTGAAAGTAATAGAAGTGAGCAGC
GCACATAGACTGACTTGGTGGACATCAACAGTGATAACCGATTCCATACAACAAGTATTA
TCAGACTGAGGATCTAAGGTAAGAATTGCTATGATTTTTGCTATTGGATATCAATGGGTT
AAAAAGTGAGGGCTGAGAGAATTTTTGCATTTGCTTTAGGTTTTATGCTTTCCTATGAAG
TATACATTTAACCCATAGATAGCTACATTTTTTGGTTCCATTGTATGGTGAATGAAGGGT
GGCAGTACCAGTATATATTTTGGAATTAACAACCTAAAATTACGCTTAATATGCTCTTTA
TTTTCAATCCTGCCTTTCTAAGTGGAATCTTGAGAAGAAATGACCACAACAATTGCCTAT
GAGACCCACTGTAAACGTACTGTTTTTGTCTGTGGAGATTTTTCCCTGTGTATAAATAGT
ATTCATGTATTGTATTAAATATTTATCCTACATAAAATTAAGTTATGAGCTGAACACAAA
AAAAACTTGAAGGTTTATTGCTTTTTGTTTCTCCAATTGTGCCTCTTCACCAATATTTGA
ATAATTACCCTCATTTGCCTGGCAGATCAGACGCCGCCATCACACTTCTAACACCAATGT
AGCGACCATGTAGCAGTAGGTATGGCCACATGGGGATGTTCAACCCCAGTTGTCTAACCC
CAAGGCAAGCATAGTCTCCCTAGGCAGTCGGTTGCCTCCCACAATGTATCCAATGCTACT
GTTGCCTAGGGTCGGGTTTACGAGACTAAGGCAAGCATGCAATCCCAAGAGAATGAACTC
CCTTGAGAGCTCTAGGGTCATCACAGTATTCTCCCCCTTCTCGCACTTTCTCTCGTTCCC
GTGGCGTGAATTGTATAACGACCCCCCATCCCACCCTTTCTTTCCTTGTGAAAGGTGTGC
AAGCCTGACAGGTGTCTCATGCTCTCTTACCCACACCCTCCCTCTCCTTACATTTCTCCA
G**CTCCAGTGATG**GTGAGTGTGGCTAGGCTGGTGTTGATGCTGGGGCTGCTGCTGTGTCTG
GGAGCCCAGCTGTCCTCCTCC**CAACACTGGTCCCATGGCTGGTACCCAGGA**GGCAAGCGG
GAACTGGACTCATTTACCACGTCTGAG**GT**GGGTCACAGTACTACAATCACAATGACTCAA
ATGGATATTATGCATGTGTAAAATACACAGCAGCCATTTGTGTCACCATCATTATCTATC
TATCTATCTCTCTCTCTCTCTCTCTCTCTCTCTCTCTCTCTCTCTCTCTCCTCTCTCTCTCT
CTCTCTCTCTTCTCCTCTCTCTCGCAACTACTCCTCGCGTCTTCTCTCTCTCATCTCTCT
T**CAG**ATTTCAGAGGAGATTAAACTCTGTGAGGCAGGAGAATGCAGCTACCTGAGACCACA
GCGAAGGAACATCCTTAGGAACATTCTT**GT**GAGTGCAGGGGCACCCTTATTAAAAATATT
GCAGTCAGAGTGCAGTATGCAGCAAATACTACATCCAACTGCAAAATTACTGCAGTAATT
TTGCAGTGTAACTGCAGTTAGAGTGCAATATAATACTGCAGTTATGCTGCATCCAAAATA
ACACAGTCGATGTATCAGGTGGCATTCACGTAGGCCAGGGATACAGTACCTCAAATCTGA
GCCTGGGAGGCCGGAATACTGCTAGTTTAATTTTCACTGATTGGCCACTCAGAGCCAGAG
AGCCATGTTCCGTAGCCAACCGTTGTGGAACATTGCAGATTCTAATCCTTATTCAATGTG
TCAGAGAGGCATGTTTGTTGTACATAATGTATTTTTATATGAACGTTCCATAATAATGTT
TCATTGTCACATACACCGGATAGGTGCAGTTAAATGTGTTGTTTTCAAGGGCATATGGAC
ATATTTGTCACCTTAACTGCTCAGGTATTTTAACCAGTGACCTTACAGTTACTGGTCCAA
TGCTCTAACTAACCGCTAGACTACCTGCCACCCTTTAATGTTGTGCCCTGCTGAATGCAG
CACTGGTGTCCCAGGTCTAAATCAGTCCCTGATTAAAGGAGAAGAATAAAAACCCACTTG
TGTGTGAGCATAGGAGTGTATTGTGTTACATGTGTTACAAGCATGCTGCATAGAGGTGGA
AATGGGGAGGGGGGGGGGGGGTATATTGCACCTGTAATTCATATGGTGTTACTCTCTTTG
CAGTTGGATGCCCTGGCCAGAGAATTTGAGAAGATAAAGTAACCTTACAACTGAAGCTGA
CTTAAATCCTTCCTTGTCTATTTGTTTATTTCAAATGACCGCTATTCTAATCTTATGTCC
TTTGATCCAATTTGATTGTTTATCAATTTTG**ATAA**TGTCACATCCCTCCTTTACCCTTTG
CACCCCCTTTATTGTCCCTTTAGACTCAATTACAGATGTAGCATCATGCACATGCCATTT
CCTGT**AATAAA**GTATCTATTTTGTTATTAATGTCTTCATCTTTGTCCTCTTCATTTAATT
TGATCCAAAGAAAGAATAGGTATAGAAATATACTGAACAAAAATATAAACGTATAATGCC

AAGTGTTGGTCCCATGTTTCATGAGCTGAAATTTTTGTGCGTGTGGAAAATGTATGGTAT
ATACATTTTCCACACGCACAAAAATGTTATTTCTCTAAAATGTTGTGCACAAATTTGTTT
ACATCCCTGTTAGTGAGAGTTTCTCCTTTGCCAACATAATCCATCCACCTGACAGGTGTG
GCATATCATGAAGCTGATTAAACAGCGTTACACAGGTGCACCTTGTGCTTGGAAAAATAA
AAGTCCACTCCAAAATGTGCTGTTTTGTTACACAACACAATGCTAGACATGTAATTGGCA
TGCTGAAGGTCTACCAGAACTGTTCCCAGAAAATGTAATGTTCATATCTCCACTATTTTG
AGGTGCTGAGGAGTATTTCTGTCTGTAATAAAGCCCTTTTGTGGGGAAAAACTCATCCTG
ATTGGCTGGGCCTGGCTCCCCGTGGATCGACCTGGCTGCCAAGTGGGTGGGCCTATGCCC
TCCCAGGCCCATCCATGGCTGTGCCACCACCCAGTCATGTGAAATCCATAGACTAGGCCT
TAATACATTTATTTAAATTGACCGATTTCCTTATATGAACTATAACTCAGTAAACATTTG
GAAATTGTTGCATGTTGCCTTTATATTTTTGTTCAGATTTGAAAATACAATTGAGCAGAA
AAACATAAACAATACAGTAAAAGGTTCTCAGTTATTATATGCAAGTTGAGCACACAGAAT
TCCCAACCAAGTCTAAACAAAACTTTAACTTTACAGAGAGTAAATAATGTTATGTTTCAC
TATATTGGGATTGGAG

mRNA:

**AGAGATATCATCAGAAAGCAGCTCTACAACACTCATCGTCGCCTTGGGAAAGTGATTTGA
CACTGAGTTGCTGTATACCAACAGGTTTGAAAGTAATAGAAGTGAGCAGCGCACATAGAC
TGACTTGGTGGACATCAACAGTGATAACCGATTCCATACAACAAGTATTATCAGACTGAG
GATCTAAGCTCCAGTGATG**GTGAGTGTGGCTAGGCTGGTGTTGATGCTGGGGCTGCTGCT
GTGTCTGGGAGCCCAGCTGTCCTCCTCC**CAACACTGGTCCCATGGCTGGTACCCAGGA**GG
CAAGCGGGAACTGGACTCATTTACCACGTCTGAGATTTCAGAGGAGATTAAACTCTGTGA
GGCAGGAGAATGCAGCTACCTGAGACCACAGCGAAGGAACATCCTTAGGAACATTCTTTT
GGATGCCCTGGCCAGAGAATTTGAGAAGATAAAGTAACCTTACAACTGAAGCTGACTTAA
ATCCTTCCTTGTCTATTTGTTTATTTCAAATGACCGCTATTCTAATCTTATGTCCTTTGA
TCCAATTTGATTGTTTATCAATTTTG**ATAA**TGTCACATCCCTCCTTTACCCTTTGCACCC
CCTTTATTGTCCCTTTAGACTCAATTACAGATGTAGCATCATGCACATGCCATTTCCTGT
AATAAAGTATCTATTTTGTTATTAATGTC

protein:

MVSVARLVLMLGLLLCLGAQLSSS**QHWSHGWYPG**GKRELDSFTTSEISEEIKLCEAGECS
YLRPQRRNILRNILLDALAREFEKIK

GnRH2 Gene2

Organism name: Thymallus thymallus (grayling)

>gi|1594667050|gb|CM015005.1|:16481692-16485470 Thymallus thymallus isolate TTM2012 chromosome 17A, whole genome shotgun sequence

ACCTGGCACCATCCCTACGGTGAAGCATGGTGGTGGCAGCATCATGCTGTGGGGATGTTT
TTCAACGGCAGTGACTGGGAGACAAGTTAGGATTGAGGGAAGCCAAGCTTGTAGAATCAT
ACCCAAGAAGACTCGATGCTGTAATCGCTGCCAAAGGTGCTGTAACGTAACAAAAAGTGA
AAGGGTCTGAATACTTTCCAACTGCAGTGTATGTTGGAGTTGACATTTCTTCCAATTATT

ATGTGGGGAGGCCAAAATAATGTTAAACTATATACCAGGTCTATTCAATATTGGAAATAA
TGTTGGTTACCTGTCCTTGCCATTTATTATTCCGCAGATGAGACAGGACGTGGGAAAAA
ACTTGTTGCTAACATATGATGGGGATGCTTGTTTACCTGGGTGGGGGTCCTTGGGCAAG
AAAACTTTGAAGACCTATGTTTTAATCCATAACCAGAAAATACATTATCTAAATTCTACA
GCTTGTAGTAAGCACTTGATCGAAGCTACTCATGCAGTGTTGTGGAATTCTGGACCTGGA
AGGCAACTGGGTGTGCAGGCTTTTGTTCACTATCATACCTGATCCAACTAATCATGGTCT
AATCCAGGGGTGGGCAAACAAACAAACTCAATATACCCTGCTTTAAAATGACTAAAACTA
AATCAAAACTGTAGAAATGATAATGGACCTACATTCATAGAGATCTTTGACGGTGTCCAA
CTTGCTAATAATCATCAAAATGAAAGCTAGACAGTCAGGGAGTATCGGAATTTTCTCGAA
ACGATAGATGGAGGGAAAGTTTCTTGCAAATTTCAGCGGTGAGGAAATATAACCAATTTT
CAGTGCAGCCCTCCAGACATCGTTGAAGACCAAATGCGTCCCCCAGGTCAAAATTACACC
ACTAGTCTAAGGATCAGGTCCCCCCTGTTATTCATTATGGTTTAAAAGGCTAAACTGATC
CTACATCCTACTCTAACATGTTTTGTGAATACTTGCCCAGGTACTCCGGTCCCGTCTCAG
AAATGTTTGGCGGGCAAGGCGATGTCTCATCGGTGACTCATGCAGCACTCTGTCATGTCC
TCCTGCTTCCTGCACTCCCTTCGTGCTAGCAGCGCCGTGTCAGTCAGCAGATAAAGTATT
TCTGTCTAAGTCAACAGAGAGAAGACACACTGTCAAACAACCGCCCGATAGGAACTCCCC
CTATGCATTTTAAACATGAAAATTTGTCACTTTCTAATTGATACCCCTTACCCCAAGCAA
AAAGGCCCATCAGACCGGAATTGAGGGTGATTGTGGCGGCTGGAATGTTTGATTGTGCTC
GTGTTTGCTTGTCTGGTGTCCCCATTGTCACCGTGTCCTGGCGGTCACTTCAATGTGATG
GAGGCTGTGTGACAGGCCACGGCACCACTTTAAGCATGGGCAAATAGAGGAAATGGGGTC
AGTGATCCTCAACCCCCAGACTGCAGACCAACACTGATCCCTGGGATGTTGTTGACTGGT
CCCACATGCTGTTCGCTAAATGACACCAATTTGGTCAGTTCTCAAGTGATAGTTGTACTT
GTAAGTGGCCCCCACAGCAGGAAGGACAGCTGCTTGCCAGTCCCTGGTAAAGGAAGGAGC
ATAGCTATAGCTTGCTGGTGACAGCCAGTCCGTGTTATAGAAAGGTTGAGACACATAAAT
TTGTTCAACCTTGATTAGTCTTAGGTTTTGCTAGTTTCCCCAGTTTGAAATCAGAACACA
ACTGACAAAATCTGAACCAGTCCAGTTGATATTTGCCTACTATGGAATTTTTCCATGTGG
TGAGAAAATATGTGAACATGCTGTGAATGAAACCAGAAAGACCTTATCCCTCAACAATTT
AAATTACTTTTATTGTCACCAGAGTCAAATACAAAATAACAAATACAAAATAAACTTATT
TAGTGATCATATACCTGTATGGTTATGAGTATACAGTAGCTAGCTAGGATTTTTTTTGTT
GGAGAGGAATATTTTTCTTTGCTAGCTTTTTCTATCAAGCTAGCTGAGAGCAGATTTGAT
GAAATTGAACATTTGATCCTATTTAGAAAATGAATTTCTGTTAAGAAAACATAACAAACG
TATTTCATAATATAGTTGTATTTTTTTTATGCATTAAAATGGCCTAGAGGAGTGTCCCAG
TTTGAGAGTAGGCGATTGGCATACTGGAACACTGTAAAAATGTAAATATTCTAAAAAGGG
GAGAGAAGTAAGTAAAGCCATTTACAGTCATTTTGATTTACAATTACATCCCATTACAAA
GTATGTTGTAATGAGAGACTGAATATCCTGTTTTTTATATCCTTAACATTATTTACTTTA
GAACCCTATGTCATTTTATTTACCATTATGTCTCTAGAGACACGTGTCATTAAACGGGAT
ACCACGGCCACAATGACGATGCCATACCCATTACATTTTTTTATTTTTTTTATTTAACTA
GGCAAGTCACTTAAGAACAAATTCTTTTTTACAATGACGGCCATTGAACTCATGTTTTAT
CATCACATACTTTCTCACAAACACACAGGCATTGATTGTTGGTTATGTTATTGTACACAT
GTAAAGATATCCATTACAAAGCTTAAGTTTCCCACATTACCAGTTGTCCCACTTCACCAA
TACTCACCCTACGTCACTAAATGTGTTGATTTTGGTGGAAGGGGGGCAGGGAGGGAGGGA
GGGAGGGAGGGTGAAATGGGGGTATTAGATAGAGGACGGACGGACATCCCAAGGGAGGTC
AGGACTGTTTGGAGATGATAATTGGAATTGAGCGGATCTTACGCACAACCCGTGTCCCTA
AACACCGCTCAGCTGACCGTTGGGAAGGGAAGGAGCAAAGGGTTCAACGATGGGCAGGGC
GAAGTGGCGTGGGGGGAAGCATTAGAGGTGGGGACATAAATTGAGGAGGAAGAGGAGGCT
TC**AGAGATATCAACAGAGACCAGCTCAACGACACTCATGTTGCCTCAGGAAAAGTGATGA**
GCCACTGAGTTGCTGTATACCAACAGGTTAATAGTAGGCATAAGTGAACAGCGGGCACAC
AGACGTACCTGGGGGACATACCACAGTGATTATCAGACTAAGGGTCTAAGGTAAGAATTG

CTATGATTTTTGCTCTTGGATATTAATGGGTCTGAAGTGAGATCTTGGAAAGGATTATAG
CATTTGCTTTGGGTTTGATGCTGTATATGAATAATTCAACGTAAAAAAAAAAAAATCCTAT
GTATTTATTGTCATTTTACAATGAAGATTAAGCTGTTAACATGTTCTTTATTTTCAGTCC
TGCCTTGGTAAGTGGAATCTTGAAGAAATTACCTCAACAATTGTCTTTGACAAATTGATC
TTTGAAACCCACTGCAAACTTGACTGTATTTGTTTGTGGAGATTTTTTTCCGCCTGCAGA
AATATTATTTGATTGTATCAGATATTTCTCCAGCATTGGATAACTTGTCATCCTGACTGT
ATCACAACCGGACGTGATTGGGAGTCCCATAGGGCGGCCACAATTGGCCCAGCGTCGTCC
GAGTTTTCCGGTGTAGGCCGTCATTGTAAATAAGAATGTGTTCTTAACTGAATTGCCTAG
TTAAATTGAGGTTCAATAAAATTATGAGCGGCACAAGAAAAACACATTTTAAAATGTAAG
GCATGTTAAGTCTTGTTGTTGCTGTTTGTTTGTCCAACCGTGCCTCTTCAACAACATTTG
GATGTCTGGCAGATCAGAAGGCGCCATCACACTTCTGACACCAATGTCACTACCAGCAGT
AGTAGGACAGGCCACATGGGCTGCATTTACTCAGGCAGCTTAATTCTGAGCTGTTGCCCA
ATTATTGGAAAAAGAGCTGATCGGATTGGTTAAAAGACACATTTTGTGAGAAAACATTTT
TTGAATTTTTGGCAGCCTGTGTTAATGCAGCCTCTTGCACTTTCTCGTTCCAGAGGCATT
CCTTGTATAACCAGCCCCCATCCCTCCCTTTAATTCCTTGTGAAAGGTGTGCCCTACTGC
ACAGACAAGTGTCACATGCTCTCTTAACTCTCCATACATCTCTCCATCCCTCTCCACCCA
G**CTCCAGTGATG**GTGAGTGTGGCTAGACTGGTGTTTATGCTGGGGCTGCTGCTGTGTCTG
GGAGCCCAGCTGTCCTCCTCC**CAGCACTGGTCCCATGGCTGGTACCCAGGA**GGCAAGAGA
GAGCTGGACTCATTCGCCACCTCTGAG**GT**GGGTCACAGTACTACAATCTCAATGACTCAA
ATGGATATTACATACAGCACTTTTCATCAGGAGTCAAGACAGGTGTTTAGCACCTGTCTG
GGTAACACACAGTAGCCATTTGTGTCACCATCATGCTGTTATCTCTCTCTCTCTCTCTCT
CT**CAG**ATTTCAGAGGAGATTAAACTCTGTGAGGCAGGAGAATGCAGCTACCTGAGACCCC
AGCGAAGGAACATCCTTAAAAACATTATT**GT**GAGTGCAGGGGCGGGAAGGCAGTAGTGCA
TTCATGTAATGATACTGTATCAGGTGGTAATCATGTAGGCTGGGGATAGCTCAAATCTGA
GCCTGAGGGGCTGGAATATTTCTTGTTTTCTTTTCTACATGGTCACTGATTAGCCACTCA
CCGGGGCTGTGACAAAATCTGAACCAGTCCAGTTGATATTTGCCTACTATGGAATTTTTC
CATGTGGTGAGAAAATATGTGAACATGCTGTGAATTGCTATGATTCCTTATTGTGTGTCA
GAGGCATGTTTGTTCTACATAATGTATTTCTATCTGACATATCCATAAAGTTGTGCCCTG
ATGAATGTAGCCCTGGTGTCCCAAGTCTAAATCAGTCCCTCATTCAGGATGAATAATGAA
ATGCTACTAGTGTGTAAGCATAGTATTATTACTTTTCTACAGGTGTTACAAGCATGTTGC
ATATCAAGGGGTGGAAATGGTGGGGGGGAACCAGGTTCCGGGAGTAAAATTATTTTGACA
TGTAAAATTAATTGAAGGATTTTACATTGGTGGGTTCCCTGTGTGACTTTCCTCAGTCAT
CTCCACCTTTGGTTGGTCCCTTTCATTAAAACATGTCTATTTTGTGGGATAAATTAAGTC
TATATTACTGCACCTGTAATTCATGTCGTGTTAACTCTCTTTG**CAG**TTGGATGCCCTGGT
CAGAGAATTTCAGAAGAGAAAGTAACCTTAGAAAACCTATCAACCGTGGCTGATTTAAAT
CCTTCCTTGTCTCTGTCTGTTTCCTTCATATGACCTCTATTCTAATCTCATGTGATTTGG
TCCAATTTTGTGTTTTATCCACATTGAT**AATCA**TGTAGCATCACTCTTTACCCTTGCATC
CTCTTTCTGTTCCTTTTTGCTGCTGTAGACTATTACAGATGTAACATCACGCACGTGTCA
CTTCCTGT**AATAAA**GTATCTATTTTGTTATTCATGTCTTCATCATTGTCCTCTCTTTAAT
TGATTTGAGGCAAAGAAAGAATAGGTCAAGAAAAAATATCATGAGAGCAACGTGATCTTTT
TTTGAAAATATGATTTAAAAATAAAAACATAAACACTAGCATCAACAATAGAATAAAATA
TTGTCAGTTATTATTGACAAAATTAAGTAGTAAATAAGATATCTCAAAAAGCAGAGACGA
AGAGGCGAAGCGAGAGGGTTTATTCGCCCAAAATCTGTCCACCAAAATAAGACCACTAAG
TGAGGAATATTTGTACGGTGGTCAATTACCTTAACCGTTTTAAATGTCAACTTCAACGGA
GTAAAATTAGAGTTGGGATGTCCCAAGGATCCCAGATAGCAAGGACCATTCTGCAACACA
TTTTCATTTACCTACGCAATGCGCATGCTCTGCTCAGATAGGAAAATAACATTATTTCTA
CTCATAAAGTGGGTTCTGTATCAGATACTGACACTGTCATAAACACTAACAATACCAATG
TTTCACAGATTCAAAGTGAGTTCAATAAGAATGTGAAATTACAATTTGTAGCTAGCAAAT

TAGGTAGCAATGCTAATGTCGTTAGCCAAGGCAAGGGCGTTCTCAAATTAGCGAGCTAAG
GTTAGTTCACTAACTGTTAAATGTTGAATAAGGCACTGTAGCCTATATGGTCAACTTTAG
CTAGCCAGCTATCCGA

mRNA:

AGAGATATCAACAGAGACCAGCTCAACGACACTCATGTTGCCTCAGGAAAAGTGATGAGC
CACTGAGTTGCTGTATACCAACAGGTTAATAGTAGGCATAAGTGAACAGCGGGCACACAG
ACGTACCTGGGGGACATACCACAGTGATTATCAGACTAAGGGTCTAAGCTCCAGTGATGG
TGAGTGTGGCTAGACTGGTGTTTATGCTGGGGCTGCTGCTGTGTCTGGGAGCCCAGCTGT
CCTCCTCC**CAGCACTGGTCCCATGGCTGGTACCCAGGA**GGCAAGAGAGAGCTGGACTCAT
TCGCCACCTCTGAGATTTCAGAGGAGATTAAACTCTGTGAGGCAGGAGAATGCAGCTACC
TGAGACCCCAGCGAAGGAACATCCTTAAAAACATTATTTGGATGCCCTGGTCAGAGAAT
TTCAGAAGAGAAAGTAACCTTAGAAAACCTATCAACCGTGGCTGATTTAAATCCTTCCTT
GTCTCTGTCTGTTTCCTTCATATGACCTCTATTCTAATCTCATGTGATTTGGTCCAATTT
TGTGTTTTATCCACATTGAT**AATCA**TGTAGCATCACTCTTTACCCTTGCATCCTCTTTCT
GTTCCTTTTTGCTGCTGTAGACTATTACAGATGTAACATCACGCACGTGTCACTTCCTGT
AATAAAGTATCTATTTTGTTATTCATGTC

protein:

MVSVARLVFMLGLLLCLGAQLSSS**QHWSHGWYPG**GKRELDSFATSEISEEIKLCEAGECS
YLRPQRRNILKNIILDALVREFQKRK

Organism name: Coregonus sp. 'balchen' (whitefish)

>gi|1711366212|emb|LR664353.1|:47979796-47983276 Coregonus sp. 'balchen' genome assembly, chromosome: 10

TGCACCAACAGTTGTTGCCTTCTCACCAAGCTGCTTGCCTATTGTCCTGTAGCCCATCCC
AGCCTTGTGCAGGTCTACAATTGTATCCCTGATGTCCTTACACAGCTCTCTGGTCTTGGC
CATTGTGGAGAGGTTGGAGTCTGTTTGATTGAGTGTGTGGACAGGTGTCTTTTATACAGG
TAACGAGTTCAAACAGGTGCAGTTAATACAGGTAATGAGTGGAGAACAGGAGGGCTTCTT
AAAGAAAAACTAACAGGTCTGTGAGAGCTGGAATTCTTACTGGTTGGTAGGTGATCAAAT
ACTTATGTCATGCAATAAAATGCAAATTAATTACTTAAAATCATACAATGTGATTTTCTG
GATTTTCCCGACATGATACAATTCTAAATATCTTAAGAACTCTACTACACTATACAAAAG
AGCAAATAAATAAATGTTAAACAGGATGGATGACTCTTCTTCAAACACACATTGTGCTGA
GACCACCATTTGTATGTCGGTACTGTCAAATTGGGACACATTGATTGGTAAAGTGAGACA
CTTAATTATTTATTGTAAAGAAGGATACATTAGTGAGTATATGAGTAATTTCAATATTTA
TATGATAAACTTCAATTATTCATACAAATTAAATGTATTAAAACATTCTTTATGTATTTT
TGTGGAACATTAAAAATGATAATGTATTATTATTATTGTCATCATACTTGATTATAATTA
TATGATGTAGCCTATATGTAATTGTATAGCCTATAAAATAAAATAAAGAACTTAATCTAG
GCCTATCAGCTCAACATTCTAAACACTACACTCTAATGGACATTTTGTAGAATGAAACCA
TAAAGACCTTATCCCACAACAATTTCAATGACTTTTATTGTCACCAGAGTCAAATACAGC
AAAATATACCTGTATTGTTATGAATATACAGTAGCTAGCTAAGATTTTTTATGTTGGTGA
GGTAATTTTTTCTAAGTAAAATGCAGTTATGCTAGCTTTTTCTAGCAGGCTAGCTGATAG
CAGATTTGAGGAAAATGAAATGGAACATTTGATCTTATTTAGAAAATGGATTTCTGTTAA

GAAAACATGTATTTCATTAGATTAGTTTAGATATAATTTTTCTGCATTGAAATGGCCTAG
AGGAGTGTCCCAGTTTGACAGTAAGAGGGTGTCCCAGTTTGAGAGTAAGAGGGTGTCCCA
GTTTGAGAGTAGGCGATTGGCATACTGGGACACTGTCAAAATGTCAATATTCTAAAAAGG
GGAGAGTAGTAAGAAAGCCATTTACAGTCATTTTGATTTACAATTACATCCCATTACAAC
ATATGTTGTAATGAGACACTGAATATCCTTTCTTAGTTTTTTATACCCTTAACATTATTT
ACCTCAGAACCCTATGTCATTTTACTTACCTCTTGAGACACGTGTCATTACACGGGATGC
CGTGCCCACAATGATGACGTCATACCCATTGAACTGTTATTGTACACATGTAAAGATATC
CATTACAAAGCTTAAGTGTTCCACTTTGCCTGGTGTCCCACTTAACCAATACTCACCCTA
CGTCACTAAATGTGTTGATTTTGGTGGAAGGGGGCAGGGAGGGACAGTGAAATGGGGTAT
TAGATAGAGGACGAACATCCCAAGGGAGGTTAGGACTGTTTGGAGATGATAATTGGAATT
GAGCGGATCTTACGCACACCCCGCATCCCTATACACCGCTCAGCTGACCTCTGGGAGGGG
AAGGAGCAAATTGTTCAGCGATGGGCAGGGCGAGGTGACGTGGGGGAAGCATTTGAGGTG
GGGACATAAATTGAGGAGGAAGAGGAGGCTTC**AGAGATATCATCAGAGGCCAGCTCTCTA**
CAACACTCATCGTCGCCTCAGGAAAAGTAATGTGCCACTGAGTTTCTGTATACCAACAGG
TTAATAATAGGCAGAAGTGAACAGCGGGCACACAGACGTACCTGGCGGACATCAACAGTG
ATAACCGATTCCATACCACAGTGATTATCAGACTAAGGGTCTAAGGTAAGGATTGCAATG
ATTTCTGCTCTTGGGTATTAATGGCTATGAAGTGAGATCTTGGAGAGGATTATAGAATTT
GCTTTGGGTTTGATGCTGTCTATGAATAATACAAATAAATTGTTTTATAATGAAGATTAC
GCAGTTAACATGTTCTTTATTTTCAATCCTGCCTTGGTAAGTGGAATCTTGAGAAGAAAT
TACCTCAACAATTGCCTATGAGAAACTGATCTTTGAAACCCACTGCAAACTTTGTGGAGA
TCTTTCCCCCCTGCATAAATATTATTTGATTGTATGAAATATTTATCCTGCATTACAAGA
AAAACATATATTTTAAACTTTAAGGCTTGTTGCTGTTTTCCTGTCCAATCGTGCCTCTTC
ACCAACATTTGGATACCCTAATTTGCAAGCCTGGCCGATCAGAAGGCGCCATCACACTTC
TGACACCAATGTCACTACCAGCAGTAGTAGGACAGGCCACATGGGCTGCGTTTACACAGG
CAGCCTAATTCTGATCGTTTGCCCAATTATTGGAAAAAGAGCTGAACTGATTTGTCAAAA
GACCAATTTTGTGAGAAAAAAAAAAACGGATTTGGGCAGCCTGTGTTAAAGCAGCCTCTCG
CACTTTCTCTCGTTCCTGAGGCATTCATTATATAACCAGCCCGCATCCCTCCCTTTCTTT
CCTTTTGAAAGGTATGCCCTACTGCACAGACAGTATGCTCTCTTAACCCACACCCTCCCT
CTCCTTACATTTCTCCATCCCTCTCCACCCAG**CTCCAGTGATG**GTCAGTGTGGCTAGACT
GGTGTTTATGCTGGGGCTGCTGCTGTGTCTGGGAGCCCAGCTGTCCTCCTCC**CAGCACTG**
GTCCCATGGCTGGTACCCAGGAGGCAAGAGGGAGCTGGACTCATTTACCACCTCTGAG**GT**
GGGTCACAGTACTACAATCTCAATGACTCAAATGGATATTACATACAGCACTTTTAATCA
GGAATCAAGACAGGTGTTTAGCACCTGTCTGGGTAACACACAGTAGCCATTTGTGTCACC
ATCATGCGGTTCTCTCTCTCTCTCTCTCTCTCTCTCTCTCTCTCTCTCTCTCTCTCTCTC
TCTCTCTCTCTCTCT**CAG**AATTCAGAGGAGATTAAACTCTGTGAGGCAGGAGAATGCAGC
TACCTGAGACCCCAGCGAAGGAACATCCTTAAAAACATTATT**GT**GAGTGCAGGGGTGGGA
AGGGGGTTGTACATTCATGTAATGATGCTGTATCATGTGGCAATCATATAGGCCTGGGAT
AGCTCAAATCTGAGCCTGAGGGGCCGGAATATTCTCGCGTTCTTTTCTACATGGTCACTG
ATTAGCCACTCACCAGGGCCATGATCAATAGGCAAACATTGTGGAACATTGCAGATGTGA
TTCCTTATTCTGTGTCAGAGGCATGTTTGTTCTGCATCATGTATTTCTATCTGACATTTC
CATAATGTTGTGCCCTGATGAATGTAGCCCTGGTGTCCCAAGTCTAAATCAGTCCCTCAT
TCAGGGTGAATAATGAAATGCCACTAGTGTGTGAGCATAGTATTATTATTTATCTACAGG
TGTTACAAGCATGTTGCATATCCAGGGGTGGAAATAGTTGGGGGAACTGGGTTCCGGGAG
TACAATTATTTTGGATTTAAGGATTTTTCATGGGTGGGTTCCCTGTGTGACTTTCCTCAC
TCATCTCCTCCCTTGTTTAGACCATTTTTTGTGGGATAAATGAAGGCTAAAAATAATACT
GTACCTGTAATTCATGTCGTGTTACTCTCTTTG**CAG**TTGGATTCCCTGGCCAGAGAATTT
CAGAAGAGAAAGTAACTTTGGACAACCTATCAACCGTGGCTGATTTAAATCCTTCCTTGT
CTCTGTCTGTTTCCTTCATATGACCTCTATTCTAATCTCATGTGATTTGGTCCAATTTTG

CGGTTTATCCACA**AAATCA**TGTAGCATTTCTCTTTACCCTTGCATCCTCTTTCTGTCCCT
TTTTTGCTGCTGTAGACTATCAATTACAGATGTAACATCATGCACATGCCATTTCCTGT**A**
ATAAAGTTTCTATTTTGTTATGAATGTCTTCATCATTGTCCTCTCTTTAATTGATTTGAT
TCAAAGAAAGTATAGGTCAAGATAAATATCATGATGAGAGCAAGGTTATCTTTTTTGAAA
ACATGATTTGAAAATAAAAATAAAAATGTAAACACTAGCATAAACAATAGAGTAAAATGT
TCTCAGTTATTATATGAAAGTTGAGCACACAAAATTCGAACTTTACA

An unlikely poly(A) motif (AAATCA) is shown here in the position equivalent to that presented by Vickers et al. (2004) for the GnRH2 gene in *Coregonus clupeaformis*. However, a more canonical signal (AATAAA) is found approx. 100 bps downstream, which has been selected as the bona fide poly(A) site for both GnRH2 genes (also see GnRH2 Gene1). This compares more favourably to the end of the *gnrh2* mRNA described for rainbow trout (Penlington et al. 1998; von Schalburg et al. 1999a).

mRNA:

AGAGATATCATCAGAGGCCAGCTCTCTACAACACTCATCGTCGCCTCAGGAAAAGTAATG
TGCCACTGAGTTTCTGTATACCAACAGGTTAATAATAGGCAGAAGTGAACAGCGGGCACA
CAGACGTACCTGGCGGACATCAACAGTGATAACCGATTCCATACCACAGTGATTATCAGA
CTAAGGGTCTAAGCTCCAGTGATGGTCAGTGTGGCTAGACTGGTGTTTATGCTGGGGCTG
CTGCTGTGTCTGGGAGCCCAGCTGTCCTCCTCC**CAGCACTGGTCCCATGGCTGGTACCCA**
GGAGGCAAGAGGGAGCTGGACTCATTTACCACCTCTGAGAATTCAGAGGAGATTAAACTC
TGTGAGGCAGGAGAATGCAGCTACCTGAGACCCCAGCGAAGGAACATCCTTAAAAACATT
ATTTTGGATTCCCTGGCCAGAGAATTTCAGAAGAGAAAGTAACTTTGGACAACCTATCAA
CCGTGGCTGATTTAAATCCTTCCTTGTCTCTGTCTGTTTCCTTCATATGACCTCTATTCT
AATCTCATGTGATTTGGTCCAATTTTGCGGTTTATCCACA**AAATCA**TGTAGCATTTCTCT
TTACCCTTGCATCCTCTTTCTGTCCCTTTTTTGCTGCTGTAGACTATCAATTACAGATGT
AACATCATGCACATGCCATTTCCTGT**AATAAA**GTTTCTATTTTGTTATGAATGTC

protein:

MVSVARLVFMLGLLLCLGAQLSSS**QHWSHGWYPG**GKRELDSFTTSENSEEIKLCEAGECS
YLRPQRRNILKNIILDSLAREFQKRK

Organism name: Salmo salar (Atlantic salmon)

>gi|925216720|ref|NC_027311.1|:43589530-43592538 Salmo salar isolate Sally breed double haploid chromosome ssa12, ICSASG_v2, whole genome shotgun sequence

TCAACATTCCAAACACTACACTACACACTACAATGGACATTTTGTAGAATGAAACCAGAA
AGACCTTATCCCTCATCAATTTCAATGACTTTTATTGTCACCAGAGTCAAATCCAGCAAA
ATAAACTTTTTTTGTCGTCATGTACCTGTATGGTTATGAATATACAGTAGTTAGCTAGGA

GTTTTTATGTGGGTTAGGTTATATCTTTCTAAGTAAAATGCAGCTATGCAAGCTTTTTCT
AGCAAGCTAGCTGATAGCAGATTTGAGGAAAATGAAATTGAACATTTGATAATTCGCCTC
AGAACCCTATGTCATTTTACTTACCATTATGTCTAGAGACACGTGTCATTACACGGGATG
CCACGCCCATAGTGATGACATCATACCCATTGAACCATTTTTTTATCATCTCTAACACA
CTGGCAGTGATTGTTGGTTATGTTATTGTACACATGCAAAGATATCTATTGCAAAGCTTA
AGTGTCCCTCTTTGCCAGGTGTCCTACTTAACCAATACACACCCTACGTCACTAAATGTG
TTGATTTTGGTGTTAGGGAGCAGGGAGGGAGGGTGAAATGGGGGTATTAGATAGAGTAAG
GACGGACATCCCAAGGGAGGTCAGGACTGTTTGGAGATGATAATTGGAATTGAGCGGATC
TTACGCACACCCCGCGTCCCTAAACACCGCTCAGCTGACCTCTGGGAGGGGAAGGAGCAA
AGTGATCAGCGATGGGCAGGGCCAAGTGGCGTGGGGGTGGGGACATAAATTGAGGAGGAA
GAGGATGCTTC**AGAGATACCATAAGAGACCAGCTCTACAACACTCATTATCGGCTCAGGA**
AAAGTGATGTGCCACTGAGTTGCTGTATACCAACAGGTTAATAATAGGCAGAAGTGAACA
GTGGACACACAGACGTACCTGGGGGACATCAACAGTGACAACCGATTCCATACCACAGTG
ATTATCAGACTAAGGGTCTAAGGTAAGGATTGCTATGATTTTTGCTCTTGAGTATTAATG
GCTAGGAAGTGAGATCTTTGATAGGATTGTTGCATTTGCTTTGGGTTTGATGCTGTCTAT
GAATAATTCGGCGTAAAAAAAATCCTTGATATTTATCGTAATTTTCCAATAAATATTACG
CTGTTGACGTTCTTTATTTTCAATCCTGTCTTGGTGGAATCTTGAGAAGAAATTACCTCA
ACAATTGCCTATGAGAAACTGATCTTTAAAACCCACTGCAAAATGTGTTTGTGGAGATTT
TTTTTCCCCCTGCATAAATATTTTTTGATTGGGTCAAATATATCTCCTGCATTGGATGAG
CGGCACAAGAAAAATATTATATATATTTTTTTGATATTTTAAACTTTGAGGCTTGTTGTT
AAAGCTTGTTGTTGTGTGTTGTTGTTTGCCTGTCCAATTGTTCCTCTTCACCAACATTTG
GATACCCTAATTTGCAAGTCTGGCCGATCAGAAGGCGCCATCACACTTCTGACACCAATG
TCGCTACCAGCAGTAGTAGGACAGGCCACATGAGCTGCGTTTACACAGGCAGCCTAATTC
TGATCTTTTGCCCAATTATTGGGAAAAAGTCTGATTGGTCAACAACAACAAAAAATAATT
AAATACATTTTTTGGGGAATTGAGCAGCCTGTGTAAAAGCAGCCTCTCGCACTTTCTCTC
GTTCCTAAGGCATTAATTGTATAACCAGCATCCATCCCTCCCTTTCTTTCCTTGGGAAAG
GTATGCCTTATTGCACAGACGAGTGTTGCATGCTGTCTTAACCTACAACTTCCCTCTCCT
TACACCTCTCCATCCCTCTCCACTCAG**CTCCAGTGATG**GTGAGTGTGGCTAGACTAGTGT
TTATGTTGGGGTTGCTGCTGTGTCTGGGAGCCCAGCTGTCTTCCTCC**CAGCACTGGTCCC**
ATGGCTGGTACCCTGGAGGCAAGAGGGAGCTGGACTCATTTACCACCTCTGAG**GT**GGGTC
ACAGTACTACAATCTCAATGACTCAAATGGATATTACATACAGCACTTTTCATCAGGAGT
CAAGACGGGTGTTTAGTTCCTGTCTGGGTAACACACAGTAGTCATTTGTGTCACCATCAT
GCTGTTATCTCTCTGTCT**CAG**ATTTCAGAGGAGATTAAACTCTGTGAGGCAGGAGAATGC
AGCTACCTGAGACCCCAGCGAAGGAACATCCTTAAAAACATTATT**GT**GAGTGCAGGGGCG
GGAAGAAAGTAGTACATTCATGTGATGATACTGTATCAGGTGGCAATCATATACGGCAGG
GATAGCTCAGATCTGAGCCTGAGGGGCCGGAATACTTCTCGTTTTCTTTTCTACATGGTC
ACTGATTAGCCACTCACCAGGGCTATGATGAATAAGCAAATGTTGCGGAACACTGCATAT
GTGATGCCCTTTTCAGTGTCAGAGGCATGTTTGTTCTACATAATGTATTTCTGATATTTC
CATAATGCTGTGCCCTGATGAATGTAGCCCTGGTGTCCCAAGTCTAAATCAGTCCTTCAT
TCAGGGTGAATAATGAAATGCCACTAGTGGATGAGCATAGTATTATTATTCGTCTACAGG
TGTTACAAGCATGTTGCATATCCAGTGGTGGAAATGGTGGGGGGAACCGGGTCCCGGAAG
TAAAATTATTTTGGGTCCCGGAAGTAAAATTATTGTGTAATAAATGAAGGCAAAAAAAATA
CTGTATCTGTAATTCATGTCTTGTTACTCTCTTTG**CAG**TTGGATGTCCTGGCCAGAGAAT
TTCAGAAGAGAAAATAACCTTAGGCAACCTATCAACCGTGGCTGATTTAAATCCTCCCTT
GTCTCTGTCTGTTTCCTTCATGACCTCTAACCTCATGTGATTTGGTCCAATGTTGTGGTT
TATCCACTTTGAT**AATCA**TATAGCATCACCCTTTACCATTGCATCCTTTTCCTGTCCCTT
TTTGATGCTGTAGACTATCAATTACAGATGTAACATCATGCACATGCCATTTCCTTT**AAT**
AAAGTATCTATTTTGTTATTAATGTCTTCATCATTGTCCTCTCTTTAATTGATTTGTTTC

AACGAAAGAATAGGTTAAGAGAGATAGCATGATGAGAGCAAGGTTATCCTTTTTGAAAAC
ATGATTTGAAAATACAAATAAAAAGAAAAACAAACATGTTCTCAGTTATTATATGCAAGT
T

mRNA:

**AGAGATACCATAAGAGACCAGCTCTACAACACTCATTATCGGCTCAGGAAAAGTGATGTG
CCACTGAGTTGCTGTATACCAACAGGTTAATAATAGGCAGAAGTGAACAGTGGACACACA
GACGTACCTGGGGGACATCAACAGTGACAACCGATTCCATACCACAGTGATTATCAGACT
AAGGGTCTAAGCTCCAGTGATG**GTGAGTGTGGCTAGACTAGTGTTTATGTTGGGGTTGCT
GCTGTGTCTGGGAGCCCAGCTGTCTTCCTCC**CAGCACTGGTCCCATGGCTGGTACCCTGG
A**GGCAAGAGGGAGCTGGACTCATTTACCACCTCTGAGATTTCAGAGGAGATTAAACTCTG
TGAGGCAGGAGAATGCAGCTACCTGAGACCCCAGCGAAGGAACATCCTTAAAAACATTAT
TTTGGATGTCCTGGCCAGAGAATTTCAGAAGAGAAAATAACCTTAGGCAACCTATCAACC
GTGGCTGATTTAAATCCTCCCTTGTCTCTGTCTGTTTCCTTCATGACCTCTAACCTCATG
TGATTTGGTCCAATGTTGTGGTTTATCCACTTTGAT**AATCA**TATAGCATCACCCTTTACC
ATTGCATCCTTTTCCTGTCCCTTTTTGATGCTGTAGACTATCAATTACAGATGTAACATC
ATGCACATGCCATTTCCTTT**AATAAA**GTATCTATTTTGTTATTAATGTC

protein:

MVSVARLVFMLGLLLCLGAQLSSS**QHWSHGWYPG**GKRELDSFTTSEISEEIKLCEAGECS
YLRPQRRNILKNIILDVLAREFQKRK

Organism name: Salvelinus spp.

>gi|1340981081|ref|NC_036838.1|:22410468-22414091 Salvelinus
spp. isolate IW2-2015 linkage group LG1, ASM291031v2, whole
genome shotgun sequence

GAATATTTCATTCATTCAGATCTAGGATGTGTTATTTTTAGTGTTCCCTTTATTTTTTGA
GCAGTGTATATAAAAATCCAATGTTATGAATATCGCTAGCAAATACAGAAGAAACAACAT
TTTAATGACATACCTTCAGTAGCAAAGGGGAATATCTTCTTTCTAACAATGTCAACTTTA
TTTCTCAACTCCTAATAGAGTAAATTACACTAATTGGAACCAATTACACTCACTGGAGTA
TATGACATATGCTTTGAAAAAGCACCTGGAGCCTATACCAGTCACATGTGCCACTGGCTA
CTGGCAAGCTTTCTTGACTTGGATTCATTTTTGCCAACACAATGCTAACCTTTGTTTAAA
CAGCAGCTACAGTATTAGCGATTGTGTTTGGAGTTACTTTTCTATCTACTAGGCTATGTT
ACAGTTGACACTTATTCCAATTATAATGTGGGGAGGTCAAAATAATGCAAAACTATATAC
CAAGTCTATTCCATATAAAATGTTGGTTACCTGTCCTTGCTATTTATTATTCAGCAGATG
AGACAAGACGTGGGGGGAAAAAAACATGTTGCTGACTTGCCTAGTTAAATAAAGGTTAAA
TAAATAATATAAAATAAAAACATTATGGGGACGCTGGTTTGCCTGGGCAAGAAAACGTTG
AAGACCCCTATTTTAATCCATATCCAAATAATACATTATCTAAATTATACAGCTTGTRGT
GAGAACTTGACTGAGGCTACTCATGCAAGGATATGGATATCTGGACCTGGAAGGCCACTG
TGTGTGTAGGCTTCTATTCACTAACACACCTAATCCAACTAACCATGGTCTATTCCACGG
GTGGGAAAACAAACAAACTCATTATATCCTACTTTAAAATGACTGAAACCAAATGGAAAC
TGTAGAAATMATAGTGGACTTACATTCATAGAGATCCTTGACTGTGTCCAGCTCGCTAAT
AATCACCAAAATGAAAGCTAGACAGTCAGGGAGTATTGAAATTTCCCCAAATGATAGATT

TTTTMWRKAAWTTRKKCSGTGAGGAAATATAACCAATTTTCTGTGCAGCCCTCCAGACCT
CGTTGRGGGCCCCCAGMACAAAATGAGTTTGACGCCCCTAGTCTAAWAAGGATCAGGTCC
CTCCTGTTATTCATTATGGTTTAAAAGGCTAAACTGATCCTAGATCAGCTGATCATACAT
CCTACTCTAACATGTTTTGTGAATACYGGCCCAGGTTCTCCGGTTCCGTTGGCGCGTCTC
AAAGGTTTGGCGGACAAGGCAATGTCTCCTCGGTGACTCATGCAGTGCTCTGTCATCATG
TCCTTCTGCTTCCTGCTCTCCCCTCGCGCTGGCAGCACCGTGTCAGTCAGCAGATAAAGT
ATTTCTGTCTAAGTCAACAGAGAGACGACACTGTCAAACAACTGCCTGATAGGAACTCCC
CCTATGCATTTTAAACATGAAAATTAGCCACTCTCCAATTGATACCCCTTCCCCCAAGCA
AAAAGGCTGTCAGACGGGAAATGAGAGTGATGMTGGCGGCTGGTATGTTTGATCGTGCTC
GTGTTTGCTCGTCTGGTGTCCCCATGGCAACCGTGTCCTGGCGGTCACTCCAGTGTGATG
GAGGCCGTGTGACAGGCCACGGCACCACTTTAAGCATGGGCAAATACAGAGGAAATGGGA
TTTGGCCTAGGTCAGTGATCCTCAACCTCCGGACTGCAGACCAACACTGATCCCTGGGAT
GATGTTGACTGGTCCCACATACTGTTAGCTAAATGACACCAATTTGGTCAGTTCTCAAGT
GATAGTTGTACCCGTCAGTGGCAATTTTAGCATTTAAATCTTGGTGGGGCAAAAAAAAAA
AAAAGTGGGATGAATGGCAGCAAAGCCACTACACAACACCAAACAATACATTAATTGCAR
TATACCAGTGACAAACGGTGCCTACAAACTGTTAGGGCCTATATAAAGCTATCCCAACAG
CAGAATCCCAACAGCAGTCCCAACACCTTACCAGTGCTACACKTGGCTATTAGCGGAGCC
TTGTCTGGCAACGAAACAGTTCATTCAGCCTCATTTACTGCCTTTTAAAAAAACATAGCT
GATATGGCTGACTTGCTTAAACAAATGTGGTTTCTACTGGCAATTGAGATGTACAAAAGG
GGACAACAAGCAGATAAGATGCTATCGATTAAGACATTAATGAGCGAACTAAGACGGACG
TAGTCAATATAACTATTTGTTCAGCACTTTTGAAATGTACAGCGACAGAATTCAGAACAT
GGGCTGTTCTTACAGTGTTCTCCCTGTACACCAAGTCAGAACCGTAGGATAAATAAAGAG
GGCATATAAGCATACAATGAAAGCTCTTACAATATTCAATGATTACATTTCTCAAAAACA
GGTTATAGGCTACATATGCACCACCAAGTCAGAACAGTAGGCAAAATTAAGAGGTGAAAA
CAGACCAAATTGTTAGGGATAGGCACATGGGCTACTAACAGCTTAGTTAGTAAAATTATA
AACTTGGATCAGCTAACACAACGTGCAACTGGAACACAGGGGTGATGGTTGCTGATAATG
GGCCTCTGTACGCCTATATAGATGTTCCTTTAAAAATCAGACGTTTCCAGCTACAATAGT
CATTTACAACATTAACAATATCTACACTGTATTTCTGATCAATTTGATGTTATGTTAATG
GACAAAATGTGCTTTTCTTTCAAATACAAGGACATTTCTAAGTGACCCCAAACTTTTGAA
CGGTAGTGTATGTGTATATACTGTAGGGTGAGTATCGGTAAAGTGGGACCCTTTACAGAA
CCACCCCATTGTCTTAACCCCCAACATGATACCATTCTAAATATCTTAAGAACTCTACTA
CACTATACAAAAGAGCAAAGAAAGAAATGTTAAACACAGMATGGATGACTCTTCTACAAA
CGCAAATTGTGCTGAGACCACATTTTTTACATTGATACTGTCAAATTGGGACACCTTGAT
TGGCGAAGTGGGACACTTAATTCTTTATTGTGAAGAAGGATAAATTAGAGGATATATGAG
AAATTTCAATATTATTGTGATAAACTTCAGTTATTCATACAAACATTCTTTATGTATTTT
TGTGGAACATTAAGAATGATAATTTGTTATTGTTATTATTATTATCATCATACTTGATTA
TAATTATATGATGTAGCCTATATGTAATTGTATAGACTATAAATAAAATGAACTGAATCT
CGGCCTATCAGCTCAACATTCTAAACACTACACTGTAATGGACATTTTGTAGAATYAAAC
CAGAAAGACCTTATCCCTCATCAATTTCAATGACTTTTATTGTCACCAGAGTCAAATAGA
GCAAAATAAACATTTTTTGTCGTCATATACCTGTATGGTTATGAATATACAGTAGTTAGC
TCGGAGTTTTTATGTTGGTTAGGTTATATCTTTCTAAGTAAAATGCAYCTATGCAAGCTT
TTTCTAGCAAGCTAGCTGATAGCAGATTTGAGGAAAATGAAATTGAACATTTGATAATTC
ACCTCAYAACCCTATGTCATTTTACTTACCATTATGTGTCTAGAGACATGTGTCATTACA
CGGGATGCCACGCCCGTAATGATGACGTCATACCCATTGAACCCTTTTTTTAAATAATCT
CTAACACACAGGCATTGATTGTTGGTTATGTTATTGTACACATGCAAAGATATCCATTAC
AAAGCTTAAGTGTCCCATTTTGCTAGGTGTCCCACTTAACCAATACTCACCCTACGTCAC
TAAATGTGTTGATTTTGGTGTWAGGGGGCAGGAAGGGAGGGTGAAATGGGGGTATTAGAT
AGAGTGAGGAAGGACATCCCAAGGGAGGTCAGGACTGTTTGGAGATGATAATTGGAATTG

AGCGGATCTTACGCACACCCCGCGTCCCTAAACACCGCTCAGCTGACCGCTGGGAGGGGA
AGGAGCAAAGGGGGCAGCGATGGGCAGGGCCAAGTGGCGTGGGGGTGGGGACATAAATTG
AGGAGGAAGAGGAGGCTTC**AGAGATACCATAAGAGACCAGCTCTACAACACTCATTATCG**
GCTCAGGAAAAGTGACGTGCCACTGAGTTGCTGTATACCAACAGGTTAATAATAGGCAGA
AGTGAACAGTGGACACACAGACGTACCTGGGGACATCAACAGTGACAACCGATTCCATA
CCACAGTGATTATCGGACTAAGGGTCTAGGGTAAGGATTTGATTTTTGCTATGATTTTTG
CTCTTGAGTATTAATGGGTATGAAGTGAGATCTTTGATAGGATTATTGCATTTGCTTTGG
GTTTGATGCTGTCTATGAATAATTTTGACGTAAAAAAAATCCTTGATATTTATCGTCATT
TTACAATAAATATTACGCTGTTAACATGTTCTTTATTTTCAATCCTGCCTTGGTGGAATC
TTGAGAAGAAATTACCTCAACAATTGCCTATGAGAAACTGATCTTTAAAACCCACTGCAA
AATGTGTTTGTGGAGTTTTTTTTCTCCCCCTGCATAAATATTATTTGATTGTGTCAAATA
TATCTCCTGCATTGGATAAGTTATGAATGGCACAAGAAACACATATTTTAAACTTTAAGG
CTTGTTGTTAAGGRTTGTTGTTAAGGCTTGTTGTTAAGGCTTGTTGTTGCGTGTTGCTGT
TTGCCTGTCCAATTGTTCCTCTTCACCAACATTTGGATACCCTAATTTGCAAGTCTGGCC
GATCAGAAGGCCCCATCACATGTCACTACCAGCAGTAGTAGGACAGGCCACATGAGCTGC
GTTTACACAGGCAGCCTAATTCTGATCTTTTGCCCAATTATTGGAAAAAGATCTGATTGG
TCAAAAGACACTTTTTTCTAAAAAAAAAAATGTAATTGAGCAGCCTGTGTTAAAGCAGCC
TCTCACACTTTCTCTCGTTRCTGAGGCATTAATTGTATAACCAGCCCCCATCCCTCCCTT
TCTTTCCTTGTGAAAGGTATGCCTTATTGCACAGACGAGTGTCGCATACTCTCTTAACCT
ACAACTTCCCTCTCCTTACACCTCTCCATCCCTCTCCACTCAG**CTTCAGTGATG**GTGAGT
GTGGCTAGACTGGTGTTTATGCTGGGGCTGCTGCTGTGTCTGGGAGCCCAGCTGTCRTCC
TCC**CAGCARTGGTCCCATGGCTGGTACCCTGGA**GGCAAGAGGGAGCTGGACTCATTTACC
ACCTCTGAG**GT**GGGTCACAGTACTACAAMCTCAATGACTCAAATGGATATTACATACAGC
ACTTTTCATCAGGAGTCAAGACGGGTGTTTAGCTCCTGTCTGGGTAACACACAGTAGCCA
TTTGTGTCACCATCATGCTGTTATCTCTCTGTCT**CAG**ATTTCAGAGGAGATTAAACTCTG
TGAGGCAGGAGAATGCAGCTACCTGAGACCCCAGCGAAGGAACATCCTTAAAAACGTTAT
T**GT**AAGTGCAGGGGCGGGAAGAAAGTAGTACATTCATGTAATGATACTGTATCAGGTGGC
AATCATATACGGCAGGGATAGCTCAAATCTGAGCCTGAGGGGCCGGAATACTTCTCGTTT
TCTTTTCTACATGGTCACTGATTAGCCACTCACCAGGGCTATGATGAATAGGCAAATGTT
GCGGAACATTYCATATGTGATGCCCTTTTCAGTGTCAGAGMCATGTTTGTTCTACATAAT
GTAGTTCTATCTGATATTTCCATAATGCTATGCCCTGATGAATGTAGCCCTGGTSTCCCA
AGTCTGAATCAGTCCCTCATTCAGGGTGAATAATGAAATGCCACTAGTGTGTGAGCATAG
TATTATTATTTGTCTACAGGTGTTACAAGCATGTTGCATATCCAGGGGTGGAAATGGTGG
GGGGAACCGGGTCCCGGAAATACAAATATTTTGATGTGTGGAATAAATGAAGGCAAAAAA
AATACTGTACCTGTAATTCATGTCATGTTACTCTRTTTG**CAG**TTGGATGTCCTGGCCAGA
GAATTTCAGAAGAGAAAATAACCTTAGACAACCTATCAACCGTGGCTGATTAAAATCCTC
CCTTGTCTCTGTCTGTTTCCTTCATGACCTCTATTCTAATCTCATGTGATTTGGTCCAAT
TTTGTGGTTTATCCACTTTGAT**AATCA**TATAGCATCACCCTTTACCATTGCATCCTTTTT
CTGTCCCTTTTTGCTGCTGTAGACTATAAATTACAGTTGTAACATCATGCACATGCCATT
TCATTT**AATAAA**GTATCTATTTGTTATTAATGTCTTCATCATTGTCCTCTCTTTAATTG
ATCTGTTTCAACGAAAGAATAGGTTAAGAGAAATATCATGATGAGAGCAACGTTATCCTT
TTTGAAAAAATTATTTGAAAATACAAATAAAAAGAAAAACAAACATGTTCTCAGTTATTA
TATGCAAGTTGAGTACAAAATTCTAACTTTACAGAGAGTAAAG

Two non-DNA letters are present in exon 2 of this assembly and were adjusted to permit translation of the ORF.

mRNA:

AGAGATACCATAAGAGACCAGCTCTACAACACTCATTATCGGCTCAGGAAAAGTGACGTG
CCACTGAGTTGCTGTATACCAACAGGTTAATAATAGGCAGAAGTGAACAGTGGACACACA
GACGTACCTGGGGGACATCAACAGTGACAACCGATTCCATACCACAGTGATTATCGGACT
AAGGGTCTAGGCTTCAGTGATGGTGAGTGTGGCTAGACTGGTGTTTATGCTGGGGCTGCT
GCTGTGTCTGGGAGCCCAGCTGTCTTCCTCC**CAGCACTGGTCCCATGGCTGGTACCCTGG**
AGGCAAGAGGGAGCTGGACTCATTTACCACCTCTGAGATTTCAGAGGAGATTAAACTCTG
TGAGGCAGGAGAATGCAGCTACCTGAGACCCCAGCGAAGGAACATCCTTAAAAACGTTAT
TTTGGATGTCCTGGCCAGAGAATTTCAGAAGAGAAAATAACCTTAGACAACCTATCAACC
GTGGCTGATTAAAATCCTCCCTTGTCTCTGTCTGTTTCCTTCATGACCTCTATTCTAATC
TCATGTGATTTGGTCCAATTTTGTGGTTTATCCACTTTGAT**AATCA**TATAGCATCACCCT
TTACCATTGCATCCTTTTTCTGTCCCTTTTTGCTGCTGTAGACTATAAATTACAGTTGTA
ACATCATGCACATGCCATTTCATTT**AATAAA**GTATCTATTTTGTTATTAATGTC

protein:

MVSVARLVFMLGLLLCLGAQLSSS**QHWSHGWYPG**GKRELDSFTTSEISEEIKLCEAGECS
YLRPQRRNILKNVILDVLAREFQKRK

Organism name: Oncorhynchus mykiss (rainbow trout)

>gi|1207596112|ref|NC_035093.1|:32516534-32519896
Oncorhynchus mykiss isolate Swanson chromosome 17, Omyk_1.0, whole genome shotgun sequence

ACTCATTATACCCTACTTTAAAATGACTGAAACCAAATGGAAACTGTAGAAATTATAGTG
GACTTACATTCATAGAGATCCTTGACTGTGTCCAGCTAGCTAATAATCACCTAAATGAAA
GCTAGACAGTCAATGAGTATCAACATTTCCCCAAATTATAGATTTTTTTAGCAAATTTCA
CCGGTGAGGAAATATAACCAATTTTCTGTGCAGCCCTCCAGACCTCGTTGCGGGCCCCCA
GGACAAAATGAGTTTTGACGCCCCTAGAGTAATAAGGATCAGGTCCCTCCTGTTATTCAT
TATGGTTTAAAAGGCTCAACTGATCCAAGATCAACTGATCATACATCCTACTCCTACATC
CTAATCCTACATGTTTTGTGAATACGGGCCCAGGTTCTCCGGTTCCGTTGGCGCGTCTCA
AAGGTTTGGCGGACAAGGCGATGTCTCCTCGGTGACTCATGCAGTGCTCTGTCATCATGT
CCTTCTGCTTCCTGCTCTCCCCTCGCGCTGTCAGCACCGTGTCAGTCAGCAGATAAAGTA
TTTCTGTCTAAGTCAACAGAGAGACGACACTGTCAAACAACTGCCTGATAGGAACTCCCC
CTATGCATTTTAAACATGAAAATTAGCCACTCTCCAATTGATACCCCTTCCCCCAAGCAA
AAAGGCCTGTCAGACCGGAAATGAGAGTGATGGTGGCGGCTGGTATGTTTGATCGTGCTC
GTGTTTGCTCGTCTGGTGTCCCCATGGTCACCGTGTCCTGGCGGTCACTCCAGTGTGATG
GAGGTCATGTGACAGGCCACGGCACCACTTTAAGCATGGGCAAATACAGAGGAAATGGGA
TTTGGCCTAGGTCAGTGATCCTCAACCCCCGGACTGCAGACCAACACTGACCCCTGGGAT
GATGTTGACTGGTCCCACATACTGTTAGCTAAATGACACCAATTTGGTCAGTTCTCAAGT
GATAGTTGTACTTGTCAGTGGCAATTTTAGCATGTAAATCTTGGTGGGGCTAAACAAACA
AAAAAGTGGGATGCAGGCCAGCAAAGCCACTACACAACACAACACCAAACAATACATTAA
TTGCACTATACCAGTAACAAACGGTGCCTACAAACTGTTAGGATCTACATAAAGCTATCC
CAACAGCAGTCCCAACACCTTACCAGTGCTACACCTGGTTATTAGCGGAGCCTTATCTGG
CAACGAAACAGTTCATTCAGCCTCATTTACTGCCTTTAAAAAAAACATAGCTGATATGGC

TGACTTGCTTAAACAAATGTGGTTTCTACTGACAATTGAGATGTACAAAAGGGGACGACA
AGCAGATATAATGCTATCGATTAAGACATTAATGAGCAAGCTAGGACGGACATAGTCAAT
ATAACTAACTATTTGTTCAGCACTTTTGAAATGTACAGCGACAGAATTCAGAACATGGGC
TGTTCTTACAGTGTTCTCCCTGTACACCAAGTCAGAACTGTAGGATAAATTAAGAGGTGA
AAACAGACCAAATTGTTAGGGATAGGCACATGGGCTACTAACAGCTTTTAAAATTATAAA
CTTGGGTCAGCTAACACAACGTGCCACTGGAACACAGGAGTGGTGGTTGCTGATAATGGG
CCTCTGTACGCCTATATAGATATTCCTTTAAAAATCTGCCGTTTCCAGCTACAATAGTCA
TTTACAACATTAACAATGTCTACACTGTATTTCTGATCAATTTGATGTTATGTTAATGGA
CAAAATGTGTTTTTCTTTCAAAAACAAAGACATTTCTAAGTCACCACAAACTTTTGAACG
GTAGTGTATGTGTATATACTGTAGGGTGAGTCGGTAAAGTGGGACACTTTCTGAAAATGT
GTTTTCTGGAACACCTTACAGAGCCACCCCATTGTCTTAACCTCCAACATGATACCATTC
TAAATATCTTAAGAACTCTAATACACTACAAAAGAGCAAAGCAAGAAATGTTAAACACAG
GATGGATGACTCTTCTACAAACGCAAATTGTGCTGAGACCACATTTTTTACGTTGATACT
GTCAAATTGGGACACCTTGATTGGCAAAGTGGGACACTTAATTCTTTATTGTGAAGAAGG
ATAAATTAGAGGATATATGAGTAATTTCATTATTTTTGTGATAAACTTAAATTATTCATA
CTAACATTCTTTATGTATTTTTGTGGAACATTAAGAATGATAATTTATTATTATTATTAT
CGTCATACTTTATTATAGTTATATGATGTAGCCTATATGTAATTGTATAGACTATAAATA
AAATTAACTTAATCTCGGCCTATCAGCTCAACATTCTAAACACTACACTCTAATGGACAT
TTTATAGAATGAAACCAGAAAGACCTTATCCCTCATCAGTTTCAATGACTTTTATTGTCA
CCAGAGTCAAATACAGCCAAATAAACGTTTTTGTTGTTGTCATATGTATGTATGGTTATG
AGTATACAGTAGTTAGCTAGGAATTTTTATGTTGGTTAGGTTATATTTTTCTTAGTAAAA
TGCAGCTATGCAAGCTTTTTCTAGCAAGGAAAACAAAATTGAACATTTGATTATTCACCT
CAGAACCCTATGTCATTTTACTAACCATTATGTGTCTAGAGACACGTGTCATTACACGGG
ATGCCACGGCCATAATGATGACATCATACCCACTGAACCCTTTTTTTATCATCTCTAACA
CACAGGCATTGATTGTTGGTTATGTTATTGTACACATGCAACGATATCCGTTACAAAGCT
AAGGTGTCCCACTTTGCCATGTGTCCCACTTAACCAATACTCACCCTACGTCACTAAATG
TGTTGATTTAGGTGTTAGGGGGCAGGGAGGGAGGGTGAAATGGGGGTATTAGATAGAGTG
AGGACGGACATCCCGAGGGAGGTCAGGACTGTTTGGCGATGATAATTGGAATTGAGCGGA
TCTTACGCACACGTCCCTGAACACCGCTCAGCTGACCGCTGGGAGGGGAAGGAGCAAAGG
GGTCAGCGATGGGCAGGGCCAAGTGGCGTGGGGGTGGGGACATAAATTGAGGAGGAAGAG
GATGCTTC**AGAGATGCCATAAGAGACCAGCTCTACAACACTCATTATCGGCTCAGGAAAA**
GTGACGTGCCACTGAGTTGCTGTATACCAACAGGTTAATAATAGGCAGAAGTGAACAGTG
GACACAGACGTACCTGGGGGACATCAACAGTGACAACCGATTTAAATACCACAGTGATTA
TCGAACTAAGGGTCTAAGGTAAGGTTTGCTATGATTTTTGCTCTTGAGTATTAATGGGTA
TGAAGTGAGATCTTGGATAGGATTATTGCATTTGCTTTGGGTTTGATGCTGTCTATGAAT
AATTCGACGTAAAAAAAATCCTTGATATTTATCGTTCTTTTACAATAAATATTCCGCTGT
TCACATATTCTTTATTTTCAATCCTGCCTTGGTGGAATCTTGAGAAGAAATTACCTCAAC
AATTGCCTATGAGAAACTAATCTTTAAAACCCACTGCAAAATGTGTTTGTGGAGAATTCC
CCCCCCCTGCATAAATATTATTTGATTGTGTCAAATACATCTCCTGCATTGGATAAGTTA
TGAGTGGCACAAGAAAAACACATTTTAAACTTTAAGGCTTGTTGTTGCGTGTTGCTGTTT
GCCTGTCCAATTGTTCCTCTTCGCCAACATTTGGATACCCTAATTTGCAAGTCTGGCCGA
TCAGAAGGCGCCATCACACTTCTGACACCAATGTCACTACCAGCAGGAGTAGGACAGGCC
ACATAATCTGCGTTTACACAGACAGCCTCATTCTGATCTTTTGCCCAATTATTGGAAAAA
TATGTGATTGGTCAAAAGACACATTTGTTTAGATAATTTTTGGGGGGAATTGAGCAGCCT
GTGTTAAAGCAGCCGCTCGCACTTTCTCTCGTTACTGAGGCATTAATTGTATAACCAGCC
CCCCATCCCTCCCTTTCTTTCCTTGTGAAAGGTACGCCTTATTGCACAGACAAGTGTCGC
ATGCTGTCTTCACCTACAACTTCCCTCTCCTTACACCTCTCCATCCCTCTCCACTCAG**AT**
CCAGTGATGGTGAGTGTGGCTAGACTGGTGTTTATGCTGGGGCTGCTGCTGTGTCTGGGA

GCCCAGCTGTCTTCCTCC**CAGCACTGGTCCCATGGCTGGTACCCTGGA**GGCAAGAGGGAG
CTGGACTCATTTACCACCTCTGAG**GT**GGGTCACAGTACTACAATCTCAATGACTCAAATG
GATATTACATACAGCACTTTTCATCAGGAGTCAAGACGGGTGTTTTTAGCACCTGTCTGG
GTAACACCCAGTAGCCATTTGTGTCACCATCATGCTGTTGTCTCTCTGTCT**CAG**ATTTCA
GAGGAGATTAAACTCTGTGAGGCAGGAGAATGCAGCTACCTGAGACCCCAGCGAAGGAAC
ATCCTTAAAAACGTTATT**GT**GAGTGCAGTGGCGGGACGAAAGTAGTACATTCATGTAATG
ATACTGTATCAGGTGGCAATCATATAGGGCAGGGATAGCTCAAATCTGAGCCTGAGGGGC
GGGAATACTTCTCTTTTTCTTTTCTACATGGTCGATGAATAGGCAAATGTTGCGGAACAT
TGCATATGTGATGCCCTTTTCACTGTCAGAGGCATGTTTGTTCTACATAATGTATTTCTA
TCTGATATTTCCATAATGTTGTGCCCTGATGATTGTAGCCCTGGTGTCCCAAGTCTAAAT
CAGTCCCTCATTCAGGGTGAATAATGAAATGTCACTAGTGTGTGAGCATAGTATTATTAT
TTGTCTACAGGTGTTACAAGCATGTTGCATATCCAGGGGTGGAAATGGTGGGCGGAACTG
GGTCCGGAAGTAAAATAACTTTGACATGTGGAATAAATTAAGCCCCAAAAAAAAATACTGT
ACCTGTAATTCATGTCATGTTACTCTCTTTG**CAG**TTGGATGCCCTGGCCAGAGAATTTCA
GAAGCGAAAATAACCTTAGACAACCTATCAACCGTGGCTGATTTAAATCCTTCCTTGTCT
CTGTCTGTTTCCTTCATGACCTCTATTCTAATCTCATGTGATTTGGTCCAATTTTGTGGT
TCATCCACTTTGAT**AATCA**TATAGCATCACTCTTTACCATTGCATTCTCCTTCTGTCCCC
TTTTTGCTGCTGTAGACTATCAATTACAGATGCAACATCGTGCACATGCCATTTCCTTT**A**
ATAAAGTATCTATTTTGTTATTAATGTCTTCATCATTGTCCTCTCTTTAATTGATTTGTT
TCAACGAAAGAATGGGTCAAGAGACACATGATGAGAGCAATGTTATCCTTTTTGAAAACA
TGATTTGAAAATACAAAACAAAAAGAAAAACAAACATGTTCTCAGTTATTATATGCACAT
TGAGTACAAAATTCGAACTTTACAGAGAGTAAATAAGATCTCAAAAAGCAGAGAGGAAGA
GGCAAAGCGAGAAGGTTTACTCCGCATCAAGTCTGTCCACAAAAATAAGACCACTAAGGG
AGGAATATTTGTATGGTGGTCATTTACCTTCACCATTTGAAATATCAACTTCAATGGGGT
AAAGTCACATCCCAAGGAACCCAGACAACAAGGACCATTCTGCAACATATTTTTAGTTAC
CTATGCAATGCACATCCTCTGCTCAGATAGGAACAAAGGTGGAAGAGCAAA

mRNA:

AGAGATGCCATAAGAGACCAGCTCTACAACACTCATTATCGGCTCAGGAAAAGTGACGTG
CCACTGAGTTGCTGTATACCAACAGGTTAATAATAGGCAGAAGTGAACAGTGGACACAGA
CGTACCTGGGGGACATCAACAGTGACAACCGATTTAAATACCACAGTGATTATCGAACTA
AGGGTCTAAGATCCAGTGATGGTGAGTGTGGCTAGACTGGTGTTTATGCTGGGGCTGCTG
CTGTGTCTGGGAGCCCAGCTGTCTTCCTCC**CAGCACTGGTCCCATGGCTGGTACCCTGGA**
GGCAAGAGGGAGCTGGACTCATTTACCACCTCTGAGATTTCAGAGGAGATTAAACTCTGT
GAGGCAGGAGAATGCAGCTACCTGAGACCCCAGCGAAGGAACATCCTTAAAAACGTTATT
TTGGATGCCCTGGCCAGAGAATTTCAGAAGCGAAAATAACCTTAGACAACCTATCAACCG
TGGCTGATTTAAATCCTTCCTTGTCTCTGTCTGTTTCCTTCATGACCTCTATTCTAATCT
CATGTGATTTGGTCCAATTTTGTGGTTCATCCACTTTGAT**AATCA**TATAGCATCACTCTT
TACCATTGCATTCTCCTTCTGTCCCCTTTTTGCTGCTGTAGACTATCAATTACAGATGCA
ACATCGTGCACATGCCATTTCCTTT**AATAAA**GTATCTATTTTGTTATTAATGTC

protein:

MVSVARLVFMLGLLLCLGAQLSSS**QHWSHGWYPG**GKRELDSFTTSEISEEIKLCEAGECS
YLRPQRRNILKNVILDALAREFQKRK

Organism name: Oncorhynchus nerka (sockeye salmon)

>gi|1681300722|ref|NC_042549.1|:14074154-14077398
Oncorhynchus nerka isolate On170113-E2 linkage group LG15, Oner_1.0, whole genome shotgun sequence

TGTGGAAAATCTGTCATTGCCAACAAAGGGTATATAACAAAGTATTGAGATAAACTTTTG
TTATTGACCAAATACTTATTTTCCACCATAATTTGCAAATAAATTCATTAAAGATCCTAC
AATGTGATTTTCTGGAGAAAAAAAATCTCATTTTGTCTGTCATAGTTGAAGTGTACCTAT
GATGAAAATTACAGGCCTCTCTCATCTTTTTAAGTGTGAGAACTTGCACAATTGGTGGCT
GACTAAATACTTTTTTGCTGAATGGAAAAAAAAGCTGAAATAAATCATTCTCTCTACTAT
TATTCTGACATTTCACATTCTTTAAATAAAGTGGTGATCCTAACTGACCTAAGACAGGGA
ATTTTTACTAGGATTAAATGTCAGGAATTGTGTAAACTTTTGACTTCAACTGTATATATA
TATATATATATAAAAATCCAATGTTATGAATATTGCTAGCAAAAACAGAAGAAACAACAT
TTTAATTATATACCTTCAGTAGAAAAAGGGAATATCTTCTTTCTAACAATGTCAACTTTA
TTTCTCAACTCCTAATAGATTAATTACACTAATTGGAACCAATTACACTCACTGGAGTAT
CTGACATATGCTTTGAAAAAGCCCCTGCGAATACCAGTCACAAGTGCTGCTGGCAAGCTT
TCTTGACTTGGACATTCATTTTTGCCAACACAATGCTAACCTTTGTTTAAACAGCAGCTA
CAGTATTAGCGAACGCGTTTGGAGTTACTTTTCTATCTAATAGGCTATGTTACTGTTGAC
ACTTCTTCCAGTTATAATGTGGGGAGGTCAAAATAATGCTAAACTATATACCAAGTCTAT
TCCATATAAAATGTTGGTTACCTGTCCTTGCTATTTATTATTCAGCAGATGAGAGAAGAC
GTGGGGGGGGGGGGGGGGGGGAACATGTTACTGACTTGCCTAGTTAAATAAAGGTTAAA
TAAATAATATAAAATAAAAACATGATGGGGGACGCTGGTTTGCCTGGGCAAGAAAACGTT
GAAGACCCCTATTTTAATCCATATCCAAATAATCCATGATCTAAATTATGCAGCTTGTCG
TAAGAACTTGATTGAGCCTACTCATACAAGGATATGGATATCTGGACCTGGAAGGCCACT
GTGTGTGTAGGCTTTTATGCACTAACACACCTAAACCAACTAACCATGGTCTATCCCACG
GGTGGGCAAACAAACAAACTCATTATACCCTACTTTAAAAATGACTGAAACCAAATGGAA
ACTGTAGAAATCATAGTGGACTTACATTCATAGAGATCCTTGACTGTGTCCAGCTCGCTA
ATAATCACCTAAATGAAAGCTAGACAGTCAATGAGTATCAAAATTTTCCCAAATTATAGA
TTTTTTTTGCAAATTTCACCGGTGAGGAAATAGGAAATATAACCAATTTTCTGTGCAGCC
CTCCAGACCTCGTTGCGGGCCCCCAGGACAAAAATAGTTTTGACGCCCCTAGTCTAATAA
GGATCAGGTCCCTCCTGTTATTCATTATGGTTTAAAAGGCTCAACTGATCCAAGATCAAC
TGATCATACATCCTACTCCTACATCCTAATCCTACATGTTTTTTGAATACGGGCCCAGGT
TCTCCTGTTCCGTTGGCGCATCTCAGAAAAGTTTGGCGGACAAGGCGATGTCTCCTCGGT
GACTCATGCAGTGCTCTGTCATCATGTCCTTCTGCTTCCTGCTCTCCCCTCGCGCTGTCA
GTGCCGTGTCAGTCAGCAGATAAAGTATTTCTGTCTAAGTCAACAGAGAGACGACACTGT
CAAACAACTGCCTGATAGGAACTTCCCCTATGCATTTTAAACATGAAAATTAGCCACTCT
CCAATTGATACCCCTTCCCCCAATCAAAAAGGCCTGTCAGACCGGAAATGAGAGTGATGG
TGGCGGCTGGTATGTTTGATCGTGCTCGTGTTTGCTCGTCTGGTGTCCCCATGGTCACCG
TGTCCTGGCGGTCACTCCAGTGTGATGGAGGCCATGTGACAGGCCACGGCACCACTTTAA
GCATGGGCAAATACAGAGGAAATGGGATTTGGCCTAGGTCAGTGATCCTCAACCCCCGGA
CTGCAGACCAACACTGATCCCTGGGATGATGTTGACTGGTCCCACATACTGTTAGCTAAA
TGACACCAATTTGGTCAGTTCTCAAGTGATAGTTGTACTTGTCAGTGGCAATTTTAGCAT
GTAAATCTTGGTGAGGCTAAACATAAAAAAAATTGGGATGCATGCCAGCAAAGCCACTACA
CAACACAACACCAAACAATACATTAATTGCACTATACCAGTGACAAACGGTGCCTACAAA
CTGTTAGGATCTACATAAAGCTGTCCCAACAGCAGAATACCAACAGCAGTCCCAACACCT
TACCAGTGCTACACCTGGCTATTAGCGGAGCCTTGTCTGGCAACGAAACAGTTCATTCAG
CCTCATTTACTGCCTTTAAAAAAAACATAGCTGATATGGCTGACTTGCTTAAACAAATGT

GGTTTCTACTGGCAATTGAGATGTACAAAAGGGGACGACAAGCAGATATAATGCTATCGA
TTAAGACATTAATGAGCGACCTAGGACGAACGTAGTCAATATAACTAACTATTTGTTCAG
CACTTTTGAAATGTACAGCGACAGAATTCAGAACATGGGCTGTTCTTACAGTGTTCTCCC
TGTACACCAAGTCAGAACCGTAGGATAAATAAAGAGGGCATATAAGCATACAATGAAAGC
TCTTACAATATTCAATAATTACATTTCTCAAAAACAGGTTATAGGCTACATGTGCACCAC
CAAGTCAGAACAGTAGGCAAAATTAAGAGGTGAAAACAGACCAAATTGTTAGGGATAGGT
ACATGGGCTACTAACAGCTTTTAAAATGATAAACTTGGATCAGCTAACACAACGTGCCAC
TGGAACACAGGAGTGATGGTTGCTGATAATGGGCCTCTGTACGCCTATATAGATATTCCT
TTAAAATCAGCCGTTTCCAGCTACAATAGTCATTTACAACATTAGCAATGTCTACACTGT
ATTTCTGATCAATTTGATGTTATGTTAATGGACAAAATGTGTTTTTCTTTCAAAAACAAA
GACATTTCTAAGTGACCACAAACTTTTGAACGGTAGTGTATGTGTATATACTGTAGGGTG
AGTATCGGTAAAGTGGGACACTTTCTGAAAATGTGTTTTCTGGACACCTTACAGAGCCAC
CCCATTGTCTTAACCTCCAACATGATACCATTCTAAATATCTTAAGAACTCTAATACACT
TTACAAAAGAGCAAAGCAAGAAATGTTAAACACAGGATGGATGACTCTTCTACAAACGCA
AATTGTGCTGAGACCACATTTTTTACGTTGATACTGTCAAATTGAGACACCTTGATTGGC
AAAGTGGGACACTTAATTATTTATTGTGAAGAAGGATAAATTAGAGGATATATGAGTAAT
TTCAATATTTTTGTGATAAACTTCAATTATTCATACTAACATTCTTTATGTATTTTTGTG
GAACATTAAGATTGATAATTTACTATTATTATTATCATCATACTTTTTTATAGTTATATG
GTGTAGCCTATATGTAATTGTATAGACTATAAATAAAATGAACTTAATCTCGGCCTATCA
GCTCAACATTCTAAACACTACACTCTAATGGACATTTTGTAGAATGAAACCAGAAAGACC
TTATCCCTCATCAGTTTCAATGACATTTATTGTCACCAGAGTCAAATACAGCAAAATAAA
TGTTTTGTTGTCATATACCTGTATGGTTATGAGTATACAGTAGTTAGCTAGGAGTTTTTA
TGTTGGTTAGGTTATATTATTCTAAGTAAAATGCAGCTATGCAAGCTTTTTCTAGCAAGG
AAAATGAAATTGAACATTTGATTATTCACCTCAGAACCCTATGTCATTTTACTAACCATT
ATGTGTCTAGAGACACGTGTCATTACACGGGATGCCACGCCCATAATGATGACATCATAC
CCACTGAACCCTTTTTTAATCATTTCTAACACACAGGCATTGATTGTTGGTTATGTTATT
GTACACATGCAACGATATCCGTTACAAAGTGTCCCACTTAACCAATACTCACCCTGCATC
ACTAAATGTGTTGATTTTGGTGTTATAGGGGGCAGGGAGGGAGGGTGAAATGGGGGTATT
AGATAGAGTGAGGACGGACATCCCGAGGGAGGTCAGGACTGTTTGGCGATGATAATTGGA
ATTGAGCGGATCTTACGGACACACCATGCGTCCCTAAACACCGCTCAGCTGACCGCAGGG
AGGGGAAGGAGCAAAGGGGTCAGCGATGGGCAGGGCCAAGTGGCGTGGGGGTGGGGACAT
AAATTGAGGAGGAAGAGGATGCTTC**AGAGATGCCATAAGAGACCAGCTCTACAACACTCA**
TTATCGGCTCAGGAAAAGTGACGTGCCACTGAGTTGCTGTATACCAACAGGTTAATAATA
GGAAGCAGGGAACAGTGGACACAGACGTACCTGGGGGGCATCAACAGTGACAACCGATTA
AATACCACAGTGATTATCGAACTAAGGGTCTAAGGTAAGGATTGCTATGATTTTTGCTCT
TGAGTATCAATGGGTATGAAGTGAGATCTTGGATAGGATTATTGCATTTGCTTTGGGTTT
GATGCTGTCTATGAATAATTCAACGTAAAAAAAAATCCTTGATATTTATCGTCCTTTTAC
AATAAATATTACGCTGTTCACATATTCTTTATTTTCAATCCTGCCTTGGTGGAATCTTGA
GAAGAAATGACCTCAACAATTGCCTATGAGAAACTAATCTTTAAAACCCACTGCAAAATG
TGTTTGTGGAGATTCCCCCCCCCCCCCCTGCATAAATATTATTTGATTGTGTCAAATATA
TCTCCTGCGTTGGATAAGTTATGAGTGGCACAAAGAAAAACACATGTTAAACTTTAAGGC
TTGTTGCGTGTTGCTGTTTGCCTGTCCAATTGTTCCTCTTCGCCAACATTTGGATACCCT
GATTTGCAAGTCTGGCCGATCAGAAGGCGCCATCACACTTCTGACACCAATGTCACTACC
GGCAGTAGTAGGACAGGCCACATAATCTGCGTTTACACAGACAGCCTAATTCTGATCTTT
TGCCCAATTATTGGAAAAAGATGTGATTGGTCAAAAGACACATTTGTTTAGATAATTTTT
GGGGGGGGAATTGAGCAGCCTGTATTAAAGCAGCCTCTCGCACTTTCTCTCGTTACTGAG
GCATTCATTGTATAACCAGCCCCCATCCCTCCCTTTCTTTCCTTGTGAAGCCTTATTGCA
CAGACTAGTGTCGCATGCTGTCTTCACCTACAACTTCCCTCTCCTTACACCTCTCCATCC

CTCTCCACTCAG**ATCCAGTGATG**GTGAGTGTGGCTAGACTGGTGTTTATGCTGGGGCTGC
TGCTGTGTCTGGGAGCCCAGCTGTCTTCCTCC**CAGCACTGGTCCCATGGCTGGTACCCTG**
GAGGCAAGAGGGAGCTGGACTCATTTCCCACCTCTGAG**GT**GGGTCACAGTACTACAATCT
CAATGACTCAAATGGATATTACATACAGCACTTTTCATCAGGAGTCAAGACGGGTGTTTT
TAGCACCTGTCTGGGTAACACCCAGTAGCCATTTGTGTCACCATCATGCTGTTGTCTCTC
TGTCT**CAG**ATTTCAGACGAGATTAAACTCTGTGAGGCAGGAGAATGCAGCTACCTGAGAC
CCCAGCGAAGGAACATCCTTAAAAACGTTATT**GT**GAGTGCAGTGGCGGGACGAAAGTAAT
ACATTCATTTAATGATACTGTATCATGTGGCAATCATATAGGGCAGGGATAGCTCAAATC
TGAGCCTGAGGGGACGGAATACTTCTCGTTTTCTTTTCTACGTGGTCGATGAATAGGCAA
ATGTCGCGGAACATTGCATATGTGATGCCCTTTTCACTGTCAGAGGCATGTTTGTTCTAC
ATCATGTATTTCTATCTGATATTTCCATAATGTTGTGCCCTGATGAATGTAGCCCTGGTG
TCCCAAGTCTAAATCAGTCCCTCATTCAGGGTGAATAATGAAATGCCACTAGTGTGTGCT
CGAGCATAGTATTATTATTTGTCTACAGGTGTTACAAGCATGTTGCACATCCAGGGGTGG
AAATGGTGGAGGGAACCGGGTCCGGAAGTAAAATAACTTTGACATGTGGAATAAATTAAG
GCAAAAAAAAAATACTGTACCTGTAATTCTTGTCATGTTACTCTCTTTG**CAG**TTGGATGC
CCTGGCCAGAGAATTTCAGAAGCGAAAATAACCTTAGACAACTTATCAACCGTGGCTGAT
TTAAATCCTTCCTTGTCTCTGTCTGTTTCCTTCATGACCTCTATTCTAATCTCATGTGAT
TTGGTCCAATTTTGTGGTTCATCCACTTTGAT**AATCA**TATAGCATCACTCTTTACCATTT
GCATTCTCCTTCTGTCCCCTTTTTGCTGCTGTAGACTATCAATTACAGATGTAACATCAT
GCACATGCCATTTCCTTG**AATAAA**GTATCTATTTTGTTATTAATGTCTTCATCATTGTCC
TCTCTTTAATTGATTTGTTTCAACGAAAGAATAGGTCAAGAGGCATATGACGAGAGCAAT
GTTATCCTTTTTGAAAACATGATTTGAAAATGCAAACAAAAAGAAAAACAAACATGTGCT
CAGTTATTATATGCACATTGAGTACAAAATTCGAACTTTACAGAGAGTAAATAAGATCTC
AAAAAGCAGAGAGGAAGAGGCAAAGCGAGAAGGTTTACTCCGCTCAAAGTCTGTCCACAA
AAATAAGACCACTAAGGGAGGAATATTTGTATGGTGGTCATTTACCTTCACCATTTTTAC
CTTCACCATTTTAAATATCAATATCAAGTCAGAGTTGGGACATCCCAAGGAACCCAGACA
ACAAGGACCATTCTGCAACATATTGTTAGTTATCTATGCAATGCACATGCGCTGCTCAGG
TAGGAACAAAGATGGAAGAGCAAAGTATGTTCTCCGCTGCTATTGGAGTCAGATACTGAA
AATAACATTATTTCTACTCATAACGTGGGTTCTGTATAAACACTAATAATACCAATGTTT
CACAGATTGAAAGGGAGTTCAATAAGAATGTGAAATTACAGTTCGTAGC

mRNA:

AGAGATGCCATAAGAGACCAGCTCTACAACACTCATTATCGGCTCAGGAAAAGTGACGTG
CCACTGAGTTGCTGTATACCAACAGGTTAATAATAGGAAGCAGGGAACAGTGGACACAGA
CGTACCTGGGGGGCATCAACAGTGACAACCGATTAAATACCACAGTGATTATCGAACTAA
GGGTCTAAGATCCAGTGATGGTGAGTGTGGCTAGACTGGTGTTTATGCTGGGGCTGCTGC
TGTGTCTGGGAGCCCAGCTGTCTTCCTCC**CAGCACTGGTCCCATGGCTGGTACCCTGGA**G
GCAAGAGGGAGCTGGACTCATTTCCCACCTCTGAGATTTCAGACGAGATTAAACTCTGTG
AGGCAGGAGAATGCAGCTACCTGAGACCCCAGCGAAGGAACATCCTTAAAAACGTTATTT
TGGATGCCCTGGCCAGAGAATTTCAGAAGCGAAAATAACCTTAGACAACTTATCAACCGT
GGCTGATTTAAATCCTTCCTTGTCTCTGTCTGTTTCCTTCATGACCTCTATTCTAATCTC
ATGTGATTTGGTCCAATTTTGTGGTTCATCCACTTTGAT**AATCA**TATAGCATCACTCTTT
ACCATTTGCATTCTCCTTCTGTCCCCTTTTTGCTGCTGTAGACTATCAATTACAGATGTA
ACATCATGCACATGCCATTTCCTTG**AATAAA**GTATCTATTTTGTTATTAATGTC

protein:

MVSVARLVFMLGLLLCLGAQLSSS**QHWSHGWYPG**GKRELDSFPTSEISDEIKLCEAGECS
YLRPQRRNILKNVILDALAREFQKRK

Online Resource 3

The late-evolving salmon and trout join the GnRH1 club

Histochemistry and Cell Biology

Kristian R. von Schalburg, Brent E. Gowen, Kris A. Christensen, Eric H. Ignatz, Jennifer R. Hall, Matthew L. Rise
Corresponding author at: Department of Biology, Electron Microscopy Laboratory, University of Victoria, Victoria, British Columbia, Canada V8W 3N5

E-mail address: krvs@uvic.ca (K.R. von Schalburg)
