# Supplementary material for: The late-evolving salmon and trout join the GnRH1 club
Source: Histochem Cell Biol. 2023 Aug 11;160(6):517–39. doi: 10.1007/s00418-023-02227-z (PMC10700215; doi:10.1007/s00418-023-02227-z)
Supplement: Supplementary file 4 — Supplementary file4 (PDF 152 KB) [file 418_2023_2227_MOESM4_ESM.pdf]

Online Resource 4 Presentation of the GnRH3 genes for grayling, whitefish (*Coregonus* sp. 'balchen'), Atlantic salmon, a *Salvelinus* spp., rainbow trout and sockeye salmon. The 5'-utr, ATG start codons (green), GnRH-encoding sequence, exon/intron GT-AG donor/acceptor sites and poly(A) motifs are highlighted in bold for each gene of interest. Putative CREs and EREs in the proximal promoter of both the GnRH3 Genes 1 and 2 are underlined. We provide the putative mRNA and protein below each GnRH gene-type presented.

GnRH3 Gene1

Organism name: Thymallus thymallus (grayling)

>gi|1594667022|gb|CM015033.1|:37470642-37473334 Thymallus thymallus isolate TTM2012 chromosome 6A, whole genome shotgun sequence

TTGATACATCAGAAAAGCAGAACTTAACTTAATATATGATAATAAATGTAATATAATAC
AAATTCACATTACCTGGTTATTAAGTGTTGTAAGGCTATCATTTTCATACCAAAGGTAC
ATTTATGGATTCATAACCTCTTAGGAGTGGTCATCTGTAGCTCATTTGGTAGAGCATGT
CATTTGGGTTTGATTCTCATGACCACCTGTATTTAAAATGTTTGCACTAATGACTATAA
GACACTTTTGAAAAAGTGTTGGTTAAATGGCATACTGTGTTAGGTAAAGATATAAGGAT
TGTGTAATCATTTAATGGTATATATATTTTTTTAATATTTTCCATCTGGTCTCATCGCT
GCAACTCCCCAACGGGATCGGGAGAGGTGAAGGTGGAGTCATGTGTCCTCCGGAACATG
ACCCGCCTAACCGCGCTCCTTAACATCCGCCAGCTTAACCCGGAAGCCAGCCGCACCAA
TGTGTCACAGGATACACCTTTCAACAGGCAACCGGGGTCAGCCCGCAGGACACCCGGCC
CGCCACAAGGGTTCGCTTGAGCTTAATGAGCCAAGTAAAGCCCCACCAGCCAAACCCTC
CCCTAACCCGGACGGCGCTGGGCCAATTGTGCGTCGTCCTATGGGACTCCCGGCCACAG
CCGTTTGTGGCACAGCCCAGGATATGAACATTTGATGCAGTGCCTTAGACCACTGCTCC
ACTCGGAAGGCCCTAGAATCAATCTTATGTACAGAGTATGAAATTGAAGCCTAATGGAT
GGTGTAGCGCACAAGCATCTTGTTCCTGTGCTCTTTCTTGTCTGTATTAAGTTGATATT
TTATTAGCAAATTCTGACTCTAATAAATGCATTGAAAATAAACAAATTTGCCATTAATT
TCTGAGTATTTGCAAAAGATACAAAACATGGGTATCAAACAGTGGAGGCTGGTGGGAGG
AGCTATAGGAGGACCAGCTTAGTGTAATGGTTGAAATGGAATTAATTGAATGGTATAAA
AAATATGGAAACCACATGTTTGATTCTGTTCCTTTTATTCCATTCCAGCCATTACAATG
AGCCTGTCTTCCTATATCTCCTGCCACCAGCCTCCAATATGTTTACTGACTTCACCTCT
TTACACGTTACGAATATGTTTGTTTCCATCTAATTCAGTTTGAAGCTTATGTGCTAAGC
AGGTCCCGTTGGTGACATTTAGCGTCCATTAGGCACTTAGTGTGTCACACCTGTGGAGA
AGGGATTATAATCCTGATGACACAGACTGTTCTATGTTTAACGACCCCTATAAAATGGA
CCCATGATTATTCCC**ACCACACTGTAAGGAGGAATAGACAGAACAGAAACG**GTATGTGA
TTCATTCATATGCTTTCAAATACATTTAGATTTTTTAACTAATGTGCATTTATGTGTTG
ATAATATATACTAAGTGCATATGCAATAAGTAATCATTGCAAGACAATCCCAAATTATA
TTTGAGTTAAGATAAAATATTTTTGACCATATTTTATTTTACTGACTTTTTTTCCAG**CT**
TCCATGGATCTTAGCAGCAGAACAGTTGTGCAGGTGGTGGTGTTGGCGTTGATAGCTCA
GGTCACTTTCTCT**CAGCATTGGTCGTATGGGTGGCTACCTGGA**GGAAAGAGAAGTGTTG

GAGAGTTGGAGGCCACCATCAGG**GC**AAGTTATATATAACCTATGTTTACCTGTATGTAA
GTAACTATGTCTATCTGTACATGCATGTAATTGTCCTTTTAAGTAAATCTACTGTGTCT
TGAAAACCTGTGAACATGTGTGTATATATATACTCACGGGTCAGTTCCTGTTCACGAAA
ATGGTTTGTTCCTACAGACAGTGAGTCACATGGCCGTGGCTTTCTATATAAAGCAGGAA
GACAGGCATGTTCAGTTACTGTTCGATTTAATGTTAGAATGGGCAAAACGGGTGACCAA
AGCAAATTTGAGCATGGTTTGATCGTCGGTGCCAGGCGTATCAGATCCACCATCTCTGA
AATGGCCGTCCTTTTAGGTGTGGGATATTTGTTCAAAAAGCAGTCTTTTCAAGGGTACA
TAGGCAATGTTTATTCAGCTCTTTGAGGGTGTTGCATGTGGAGTATATGGGTAATAACT
TTGCGAATTCCTCTTACATATC**AAG**ATGATGGACACAGGAGGTGTAATGGCTCTTCCTG
AGGAGACTGATGCCCATGTCCTAGAGAGACTGAGACCATATGATGTA**GT**AAGTATTCAT
ATTCATATATTAGGTTGGATATAGAATTCATTGAATTTCTAATGGTCAATTCCTTGTTT
CGTATGAAGATATTGAATTGATGTCTCCTTATATTATGTTGTAAAAAGATATGCCAAAG
TATCTGAAAGAAATGTTAAGATGAGAGTATTGTCTGACAAATGGAAAGCTTCCCTGAAG
TGATACTTATAAATGTATGGCGACAATGTTGCTAATTCCTCGTACATCAGTATAATAAA
CTGTAGGTACAGTATTACACAACATAAAATAGACTTCTGAAGTTGACACTTTGTTTCCC
TAGATATCGAATAAACGGATGCCACATAAATAAAGAACTGTGAGACCATTCTTCACAAG
GAAGCAAGAAAACAAATACAGTTCTGTCCATTTCCAACTAAGATCACATTTAGATTGAT
GTCATTTACTTGAAGTATGAAACTTTAACCTCCTGTAAAATTGG**AATAAA**GAGTGATTT
GAAATATATGTCTCTTTGTATGTCCTTACAATACAGTAGCTGCGGTGTCCTCTTCAATT
TTGGATTAATCAATTAGATTGAGGAAGAGTTGAAAGACCATAAATTGATTATTCTTGTA
CATTTGTTTCCCACGACCAACAAGCATGATCAACTTGTAGTTCAATAACCTTAGCGTAA

mRNA:

ACCACACTGTAAGGAGGAATAGACAGAACAGAAACGCTTCCATGGATCTTAGCAGCAGA
ACAGTTGTGCAGGTGGTGGTGTTGGCGTTGATAGCTCAGGTCACTTTCTCT**CAGCATTG**
GTCGTATGGGTGGCTACCTGGAGGAAAGAGAAGTGTTGGAGAGTTGGAGGCCACCATCA
GGATGATGGACACAGGAGGTGTAATGGCTCTTCCTGAGGAGACTGATGCCCATGTCCTA
GAGAGACTGAGACCATATGATGTAATATCGAATAAACGGATGCCACATAAATAAAGAAC
TGTGAGACCATTCTTCACAAGGAAGCAAGAAAACAAATACAGTTCTGTCCATTTCCAAC
TAAGATCACATTTAGATTGATGTCATTTACTTGAAGTATGAAACTTTAACCTCCTGTAA
AATTGG**AATAAA**GAGTGATTTGAAATAT

protein:

MDLSSRTVVQVVVLALIAQVTFS**QHWSYGWLPG**GKRSVGELEATIRMMDTGGVMALPEE
TDAHVLERLRPYDVISNKRMPHK

Organism name: Coregonus sp. 'balchen' (whitefish)

>gi|1711365939|emb|LR664344.1|:54777960-54780734 Coregonus sp. 'balchen' genome assembly, chromosome: 1

AAAAATATATGCTTTATTCTCAACTTGAAAAGTGTAATTTAGAGACAGGCATCAATTTC
CTCTTGATATGGAGTGTTCAGTCACAGTGCCCTCATATCAATCACTAATTAACTTAATT
ATTATTTATTGTAATTTAGCAGACACTCTTATCCAGCGCAACTTACAGGAGCAATTATG

GTTAAGATTTTTCACCTAGCCGCCTCAGGGATTCAAAGCAGTGGTCTTTAATTTACTAG
CCCAACACTCTTAACCGCTAGGCAACACGTTAGGCTATCTGCCACCCGGTTATTGACAA
TAGCACATTTCAGGAAGCTAGTTTCTGAAATGTCTATGTTCTGTCATTTACTATGAAAT
ATATATACAGTATATGATCATAAATGTAATATAATAAACATTCAAATTACCTGGTTAAC
TGGTTATTAAGTGTTGGTAGGCTATCCTTTTCATACCAAAGGTACATTTATGGATTCAT
TACCTCTTTGGCTATCTGTAGCTCAGTTGGTAGAAAAATGCGCTTGCAACCCCAGTATA
GTGGGTTCGATTCTCAGGACCACCTGTATGTAAAATGTATGTACTAATGACTGTAAGAC
GCTTTGGAAAAAAGTGTCTGCCAAATGGCATACTGTATTAGGTAAAGAGATAAGGATTG
TGTAATCATATAATGGTCTTCATTTAGAATCAATCTTATGTACAGAGTATGGAATTGAG
GCCTAATGTAGTGCACAAGCATCTTGTTCCTGTACTGTCTCTTGTCATAAGTCTGTTTG
ACGTTTACATTTTATTTGCAAATTCTGATTGCAATAAATGCATTGAAAATAAACTAATT
AGCAATCAATTTCTGAATATTTGTAAAAGATACAAAACATGGGTATCAAACAGTGGAGG
CTGGTGGGAGGAGCTATAGGAGGACGAGCTCATTGTAATGGTTGAAATAAAAATAATGG
AACGTTATCAAACATATGGAAACCACAAGTTTGACTGTTGTTTTATTCCATTCTAGCCA
TTACAATGAGCCTGTCCTCCTATAGCTCCTCCCACCAGCCTCCACTTGTATCAAAGTAA
CTTTTTTATTGAGCAGAATATATTATTTCATTTACAAACATTACATATTTTATAATATG
TGCACTGACTTCACCTCTTAACACATTACGAATATGTTTGTTTCCATCGAATTCAGTTT
GAAGCTTATGCACTAAGCAGGTCCCGTTTGTGACATTTAGTGTCCATTAGGCACTTAGT
GTGTCACACCTGTGGAGAAGGGATTATAATCCTGATGACACAGACTGTTCCATGTCTAA
CGACCCCTATAAAAGGGACCCATGATTATTCCC**ACCACACTGTAAGAAGGAATAGACAG**
AACGGAGACAGTATGTGATTCATTCATGTGCTTTCAAATACATTTAAAATAGTTAACTA
TTGTGCATTTATGTGTTGATAATATATACTAGTTGCATATTCAATAGGTAATCAATGCA
AGACAATCCCAAGCTGTATTTGAGTTTAGATAAAATATTTTTGACTATCAAATGTAGCT
TGTATTTTACTGGCTTTTTTTCCAG**CTTCCATG**GATCTTAGCAGCAGAACGGTTGTGCA
GGTGGTGTTATTGGCGTTCATAGCTCAGGTCACTTTCTCT**CAGCATTGGTCATATGGGT**
GGCTACCTGGAGGGAAGAGAAGTGTTGGGGAGCTGGAGGCCACCATCAGG**GC**AAGTACT
ATTTACCCTATGTTTACCCGTATATAAGTAACTATGTCTACCTGTACATACATGTAATT
GTCCTTTAACTAAATCTATTGTGTTTTAAGTAAAAACCTGTGTACATGTATATATACAC
TCATGGGTCAGTTCCCCATTCACAAAAGTGGATCGGTCCTACAGACAGTGAGTCACGTG
GCCGTGGCTTGCTATATAAAGCAGGCAGACAAGCATCAAGGTATTCAGTTACTGTTCGA
TTAAACGTTAGAATGGGCAAAACAACTGACCAAAGCGACTTTGAACTTGGTATGATCGT
CGGTGCCAGGCGTGCCAGATCCAGTATCTCAGAAATGGCCGTCATTTTTGGTGTGGGAT
ATTTGTTCAAGAAGCAGTCTTTTCAAGAGTACATATGCAATGTTTATTAAGCTCTTTGA
GGGTGTTGCATATGGAGTATATGGGTAATAACATTACATATTCCTCTTACATATTGCTA
TC**AAG**ATGATGGACACAGGAGGTGTAATGGCTCTTCCTGAGGAGACAGGTGCCCATGTC
CCAGAGAGACTGAGACCATATGACGTA**GT**AAGTATTCATATTCATATATTAGGTTAGAT
ATTGTATTCATTGTATTTCTAATGGTCAATTCCTTGTTTCGTCTGAAGATATTGAATTG
ATGTCTCCTTGTAATATGTTGTAAAAAGATATGCCAAAAAGAAATGTTAAGATGAGAGT
ATTGGCTGACAAATAGAAAGCTTCCCTGAAGTGATACTTATAAATGTATGGATACAATG
TTGCTCATTTCTGATACATCAGTATAATATACTGTAGATACAGTATTATACAACATAAA
GTAGACTTCTGAAGTTGACACTTTGTTTCCCT**AG**ATATCGAAGAAACGCATGCCACATA
AATAAAGAACTGTGAGACCATTCTTCACAACGAAGCAAGAAGACAGCGATGATGGGGCA
TTCAAGTACAGTTCTGTCCATTTTCAACTAAGATCACATTTAGATTGATGTTATTTACT
TGAAGTATGACATTTTAACCTCCTGTAAAATTGT**AATAAA**GAGTGATTGAAAGATATGT
CTCTTTGTATCTCCTTACAATACAGTAGCTGCAGTGTCCTATCCAATTTTGGATTCATC
AATTAGTGACCCATCATAAGATTGAGATGCAGTTGACAGACCATAAATTGATTATTATT
GTACATTTGTTTTCCATGACCGACAAGCATGATCAACTTGTAGT

mRNA:

ACCACACTGTAAGAAGGAATAGACAGAACGGAGACACTTCCATGGATCTTAGCAGCAGA
ACGGTTGTGCAGGTGGTGTTATTGGCGTTCATAGCTCAGGTCACTTTCTCT**CAGCATTG**
GTCATATGGGTGGCTACCTGGAGGGAAGAGAAGTGTTGGGGAGCTGGAGGCCACCATCA
GGATGATGGACACAGGAGGTGTAATGGCTCTTCCTGAGGAGACAGGTGCCCATGTCCCA
GAGAGACTGAGACCATATGACGTAATATCGAAGAAACGCATGCCACATAAATAAAGAAC
TGTGAGACCATTCTTCACAACGAAGCAAGAAGACAGCGATGATGGGGCATTCAAGTACA
GTTCTGTCCATTTTCAACTAAGATCACATTTAGATTGATGTTATTTACTTGAAGTATGA
CATTTTAACCTCCTGTAAAATTGT**AATAAA**GAGTGATTGAAAGAT

protein:

MDLSSRTVVQVVLLAFIAQVTFS**QHWSYGWLPG**GKRSVGELEATIRMMDTGGVMALPEE
TGAHVPERLRPYDVISKKRMPHK

Organism name: Salmo salar (Atlantic salmon)

>gi|925216783|ref|NC_027300.1|:62421225-62424414 Salmo salar isolate Sally breed double haploid chromosome ssa01, ICSASG_v2, whole genome shotgun sequence

AAAGATTGGAAAACCTTTCTGATCAAGAATCAATTGTCGACAAGTGACTTCAACTTCAA
GTAGACAGTCTGCTAAGCCAGCAGACTGTTTTTATGTTTTTCAAGACTAAATTAAGAAG
TTAGATTTCAACAAAATATACATTTTACTATTCCCATATAGTTAATAAAACAGCCTTCA
TCTCTGTAATAAACAAGATTGGTGTTAAGGGAAATTATTATAATACCAATGTACCAAAA
ATATAGTATTTATTCTGTTTATAGAGTTCTCAACTTGAAAAGTATACATTAAAAACAGG
CATCAATTTCCTCTTGATAAGGAGTGTTCAGTCATAGTGCCCTCATATCAATCACTAAT
TAACTTGATTATTATTAGCATTTAGCAGACACTCTTATCCAGAGCAACTTACAGGAGCA
ATTATGGTGAAGTGTCTTGTTCAAGGGCACTTCCACCTAGTCGCCTCAGGGATTTCGAA
CCAGTGGTTTTTCTGTTACTAGCCCAACCGCTAGGCAACACGTTAGGCTACCTGCCACC
CGGTTATTGACAATAGCAAATTTCAGGAAGCTAGTTTCTGGAATTTCAATGGGAAATAT
ATACACAGTATATGATCATAAATGTAATATAACACAAATTTGAATTACTTGGTTACCTG
GTTATTAAGTGTTGTTAGACTATCCTTTTCATACCAAAGGTACATTTATGCATTCATAA
CCTCTAAGGAGTGGTTATCTGTAGCTCTGTTGGTAGAGGATGGCACTTGCAATGCCAGT
ATAGTAGTGGGTTCGATTCTCAGGACCACCTGTATGTACTAATAACTGTAAGCTAAATG
GCGAACTGTATTAGGTAAAGGTATAAGGATTGTGTCTTCATTTAGAATCAGTCTTATGT
ACTGAGTATGAAATTAAGGCTAGTGGTTCCTGTACAGTCTCTTATCATTTTTTTAATTT
TACCTTCATTTAACTAGGCAAGTCAGTTAAGAACAAATTCTTATTTTCAATGACGGCCT
AGGAACAGTGGGTTAACTGCCTTGTTCAGGGGCAGAACAACAGATTTTTACCTTGTCAG
CTCGGGGATTTGATCTTGCAACCTTTCAGTTACTGGTCCAACACTCTAACCACTAGGCT
ACCTGCCGCCCCAATCTTAAGTCTGTATAAAGTTTACATTTTTTAATTCTGAGTGCAAT
AAATGCAAAGAAAATAAACAAATTTGCCATTCATTTCTGCATATTTGTAAAAGATACAA
AACATGTGTATCAAACAGTGGAGGCTGGTGGGTGGAGCTATAGGAGGACAGGCTCATTG
TAATGGCTGGAATGGAATAAATGGAACGGTATCAAACACATCAAACATATGGAAACCAC

ATGTTTGACTCTGTTCCTTTTATTCCTTTCCAGCCAATACAATGAGCTTGTCCTCCTAT
AGCTCCTCCCACCAGCTGCCACTTGTATCAAAGTAACTTTTTTATTGAGACAAATATAT
AATTTAATTTACAAACATTACATATTTATAATATCTACACTGACGTCACCTCTTAACAC
ATTACAAATTTGTTTGCTTCCATCAAATTCAGTTTGAAGCTTATGCACTAAGCTGGTCC
CGTTGGTGACGTTTAGTGTCCATTAGGCACTTAGTGTGTCACACCTGTGGAGAAGGGAT
TATAATCCTAATGACTGTTCCATTTCTAACGACCCTTATAAAAAGGACCCATGATTATT
CCC**ACCACACTGTAAGGAATAGACGGAACGGAGACA**GTATGTGATTCATTCATATGCTT
TCAAGTACATTTAAAATTGTTAACTGATTTATGTGTTGATAATATCTACTAAGTGCATG
TTCAATAGGTAATCATTGCAAGACAATCCCAAACTATATTTGAGTTATGATAAAATATT
TTGGACTATCAAATGTAGCTTGTATTTTACTGGCTTTTTTTCCAG**CTTCCATG**GATCTT
AGCAGCAAAACGGTTGTGCAGGTGGTGATGTTGGCGTTGATAGCTCAGGTCACTTTCTC
T**CAGCATTGGTCGTATGGGTGGCTACCTGGA**GGAAAGAGAAGTGTTGGGGAGCTGGAGG
CCACCATCAGG**GC**AAGTACTATTTACCCTATGTTTACCTGTATGTAAGTAACTATGTCT
ACCTGAACATACATGTAATTGTCCTTTAAGTAAATCTACTGTGCCTTGAAAACCTGTGA
ACATGTATATATACACCCAGGGGTCAGTTCCCCTTTCACGTAAATGTATCGTTCCTACA
GACAGTGAGTCACACAGCCGTGGCTTGCTAGTCACGTGGCCGTGGTTTGCTATATAAAG
CAGGCAGACAAGCAACAAGGTATTCAGTTACTGTTCGATTGAACGTCAAAATGGGCAAA
ACAAGTGACCAAATCGACTTTGAGCATGGCATGGTGTCGTGCCAGGCGTGCCAGATCCA
GTATCTCAGAAATGACCGTCATTTTTGGAGTGGGATATTTGTTCAAGTAGCAGTCTTTT
CAAGAGTACATAGGCAAAGTTTATGAAGCTCTTTGAGGGTGTTGCATATCGAGTATATG
GGTAATAACATTACAAATTCCTCTTACATATTGCTATC**AAG**ATGATGGACACAGGTGGT
GTAATGGTTCTTCCTGAGGAGACAGGTGCCCATGTCCCAGAGAGACTGAGACCATATGA
TGTA**GT**AAGTATTCATATTCATATTATAGGTTAGATAATGTATTCATTGTATTTCTAAT
GGTAAATTCCTTGTTTCGTCTAAAGATATTGAATTAATGTCTCCTTGTAATATGTTGTA
AAAAGATATGCCAAAGTATCAGAAAGAAATGTTAAGATGAGAGTATTGGCTGACAAACG
GAAAGCTTTCCTGAAGTGGTACTTATAAATGTATGGAGACAATGTTGCTAATTTCTGAT
ACATCATTGTAATATACTGTAGATACAGTATTATACAACATAAAGTAGACTTCTGACGT
TTTCACTTTGTTTCCCT**AG**ATGTCAAAGAAACGGATGCCACATAAATAAAGAACTGTGA
GACAATTCACAAAAAGCAAGAAGACAACGATGATGGAGCATTCAAGAACAGTTCTGTCC
ATTTCCAACTAAGATCACATTTAGATTGATGTAATTTACTTGAAGTATGAAACACTAAC
CTCCTGTAAAATTGT**AATAAA**GAGTGATTGAAAGATATGTCTCTTTGTATGTCCTTACA
ATAC

mRNA:

ACCACACTGTAAGGAATAGACGGAACGGAGACACTTCCATGGATCTTAGCAGCAAAACG
GTTGTGCAGGTGGTGATGTTGGCGTTGATAGCTCAGGTCACTTTCTCT**CAGCATTGGTC**
GTATGGGTGGCTACCTGGAGGAAAGAGAAGTGTTGGGGAGCTGGAGGCCACCATCAGGA
TGATGGACACAGGTGGTGTAATGGTTCTTCCTGAGGAGACAGGTGCCCATGTCCCAGAG
AGACTGAGACCATATGATGTAATGTCAAAGAAACGGATGCCACATAAATAAAGAACTGT
GAGACAATTCACAAAAAGCAAGAAGACAACGATGATGGAGCATTCAAGAACAGTTCTGT
CCATTTCCAACTAAGATCACATTTAGATTGATGTAATTTACTTGAAGTATGAAACACTA
ACCTCCTGTAAAATTGT**AATAAA**GAGTGATTGAAAGAT

protein:

MDLSSKTVVQVVMLALIAQVTFS**QHWSYGWLPG**GKRSVGELEATIRMMDTGGVMVLPEE
TGAHVPERLRPYDVMSKKRMPHK

Organism name: Salvelinus spp.

>gi|1340979661|ref|NC_036858.1|:39913083-39916271
Salvelinus spp. isolate IW2-2015 linkage group LG18,
ASM291031v2, whole genome shotgun sequence

TTTTTATGTTTCTCAAGACTAAATAAGAAGTTAGATTTCAACAAAATATACATTTTACT
ATTTCCATATAGTTAATAAAACAGCCTTCATCTCTGTAATAAACATGATTGGTGTTAAG
GGAAATTATTATAATACCAAATATACCAAATATATATGATTTATTCTGTTTATAGAGTT
CTCAACTTGAAAAGTATCATTTAGAAACAGGCATCAAATTCCTCTTGATAAGGAGTGTT
CAGTCATAGTGCCCTCATATCAATCACTAATTAACTTGATTATTATTATCATTTAGCAG
ACACTCTTATCCAGAGCAACTTACAGGAGCAATTATGGTGAAATGTGTTGCTCAAGGGC
ACTTCGGCAGTTTTGTCACCTAGTCGCCTCAGGGATTTCGAACCAGTGATTTTTCAGTT
ACTAGCCCAATCGCTAGGGAACACGTTAGGCTACCTGCCACCTGGTTATTGACAATAGC
AAATTTCAGGAAGATAGTTTCTGGAATTTCAATGTACTGTCATTTACTGTGAAATATAT
ACACAGTATATGATCATAAATGTAATATAACACACATTTGAATTACTTGGTTACCTGGT
TATTAAGTGTTGTTAGGCTATCCTTTTCATACCAAAGGTACATTTATGCATTCATAACC
TCTAAGGAGTGGTTATCTGTAGCTCTGTTGGTAGAGGATGGCACTTGCAACGCCAGTAT
AGTAGTGGGTTCGATTCTCAGGACCACCTGTATGTACTAATAACTGTAAGCTAAATGGC
AAACTGTATTAGGTAAAGATATAAGGATTGTGTCTTCATTTAGAATCAGTCTCATGTAC
TGAGTATGAAATTAAGGCTAGTGGTTGCTGTAGTGCACAAACATCTTGTTCCTATACAG
TCTCTTATCATTTTTTTTTGCAGTTTTTTATTTTACCTTAATTTAACTAGGCAAGTCAG
TTGAGAACAAATTCTTATATTCAATGACGGCCTAGGAACAGTGGGTTAACTGCCTTGTT
CAGGGGCAGAACGACAGATTTTTACCTTGTCAGCTCAGGGATTTGATCTGGCAACCTTT
CGGTTACAAGTCCAACGCTCTAACCACTAGGCCACCTGGCCACCATAAGTCTGTATAAA
GTTTAAAATGTTTATTCTGAGTGCAATAAATGCAATGAAAATAAACTAATTTGCCATTA
ATTTCTGAATATTTGTAAAAGATACAAAACATGTGTATCAAACAGTGGAGGCTGGTGGG
TGGAGCTATAGGTGGACGAGCTCATTGTAATGGTTGTAATGGAATAAATGGAACGGTAT
CTAACCCATCAAATATATGGAAACCACATGTTTGACTCCTTTCCTTTTATTCCATTCCA
GCCATTACAATGAGCCTGTCATCCTATAGCTCCTCCCACCAGCTGCCACTTGTATCAAA
GTAACTTTTTTATTGAGACAAATATATTATTTATTTTACAAACATTACATATTTATAAT
ATCTACAC<u>TGACTTCA</u>CCTCTTAACACATTACGAATTTGTTTGTTTCCATCAAATTCAG
ATTGAAGCTTATGCACTAAGCAGGTCCCATTGG<u>TGACG</u>TTTAG<u>TGTCC</u>ATTAGGCACTT
AGTGTGTCACACCTGGGGAAAAGGATTATAATCCTAATGACACAGACTGTTCCATTTC
TAACGACCCTTATAAAAAGGACCCATGATTATTCCC**ACCACACTGTAAGAAGGAATAGA**
CAGAACGGAGACAGTATGTGATTCATTCATATGCTTTCAAGTACATTGAAAATTGTTAA
CTGATTTATGTGTTGATAATATCTACTAAGTGCATGTTCAATAGGTAATCATTGCAAGA
CAATCCCAAACTATATTTGAGTTACGATAAAATATTTTGGACTATCAAATGTAGCTTGT
ATTTTACTGGCTTTTTTCCCAG**CTTCCATG**GATCTTAGCAGCAAAACGGTTGTGCAGGT
GGTGATGTTGGCGTTGATAGCTCAGGTCACTTTATCT**CAGCATTGGTCGTATGGGTGGC**
TACCTGGAGGAAAGAGAAGTGTTGCGGAGCTGGAGGCCACCATCAGG**GC**AAGTACTATT
TACCCTATGTTTACCTGTATGTAAGTAACTATGTCTATCTGAACATACATGTAATTGTC
CTTTAAGTAAATCTACTGTGCCTTGAAAACCTGTGAACATGTATATATACACTCATGGG
TCAGTTCCCCGTTCACGAAAATGTATCGCTCCTACAGAAGGTTAGTCACGTGGCCTTGA
CTTGCTAGTCACGTGGCCGTGGTTTGCTATATAAAGCAGGCAGACAAGCAACAAGGTAT
TCAGTTACTGTTCGATTGAACGTCAGAATGGGCAAAAGAGTGACCAAATCGACTTTGAG
CATGGTATGATTGTCGTGCCAGGCGTGCCAGATCCAGTATCTCAGAAATGGCTGTCATT

TTTGGAGTGGGATATTTGTTCAAGAAGCAGTCTTTTCAAGAGTACATAGGCAAAGTTTA
TGAAACTCTTTGAGGGTGTTGCATATCGAGTATATGGGTAATAACATTACAAATTCCTC
TTACATATTGCTATC**AAG**ATGATGGACACAGGTGGTGCAATGGCTCTTCCTGAGGAGAC
AGGTGCCCATGTCCCAGAGAGACTGAGACCATATGATGTA**GT**AAGTATTCATATCATAT
TTTAGGTTAGATAATGTATTCATTGTATTTCAAATGGTAAATTCCTTGTTTCGTCTGAA
GATATTGAATTGATGTCTCCTTGTAATATGTTGTAAAAAGATATGCCAAAGTATCTGAA
AGAAATGTTAAGATGAGAGTATTGGCTGACAAACGGAAAGCTTTCCTGAAGTGGAACTT
ATAAATGTATGGAGACAATGTTGCTCATTTCTGATACATCATTGTAATATACTGTAGAT
ACAGTATTATACAACATAAAGTAGACTTCTGACGTTGACACTTTGTTTCCCT**AG**ATGTC
GAAGAAACGGATGCCACATAAATAAAGAACTGTGAGACAATTCTTCACAAAAAGCAAGA
AGACAACGATGATGGAGCATTCAAGAACAGTTGTGTCCATTTCCAACTAAGATCACATT
TAGATTGATGTAATTCACTTGAAGTATGAAACACTAACCTCCTGTCAAATTGT**AATAAA**
GACTGATTGAAAGATATGTCTCTTTGTATGTCTGTACAATACAGTATCTGCAGTGTACT
ATCCAATTTTGGAGTCATCAATTAGTGACCCATCATAAGATTGAGGTGGAGTTGACAAA
CCATGAATTGATTATTATCGTATATTTGTTTTCCATGACCGACAAGCATGATCAACTTG
TAATTCAACAACCTTAGCATGACAACAGCTGGTGATTAAGTAAATGTATTAAAATTCAA
TGGTGTCATTAGATTTGAAACAGTGGTCTATGTCCCTTTTATAACTAGATCGCATTGCT
TGTCAGTCATTTATAATTTCATCCATAAAAACCACTAGACCAATT

mRNA:

ACCACACTGTAAGAAGGAATAGACAGAACGGAGACACTTCCATGGATCTTAGCAGCAAA
ACGGTTGTGCAGGTGGTGATGTTGGCGTTGATAGCTCAGGTCACTTTATCT**CAGCATTG**
GTCGTATGGGTGGCTACCTGGAGGAAAGAGAAGTGTTGCGGAGCTGGAGGCCACCATCA
GGATGATGGACACAGGTGGTGCAATGGCTCTTCCTGAGGAGACAGGTGCCCATGTCCCA
GAGAGACTGAGACCATATGATGTAATGTCGAAGAAACGGATGCCACATAAATAAAGAAC
TGTGAGACAATTCTTCACAAAAAGCAAGAAGACAACGATGATGGAGCATTCAAGAACAG
TTGTGTCCATTTCCAACTAAGATCACATTTAGATTGATGTAATTCACTTGAAGTATGAA
ACACTAACCTCCTGTCAAATTGT**AATAAA**GACTGATTGAAAGAT

protein:

MDLSSKTVVQVVMLALIAQVTLS**QHWSYGWLPG**GKRSVAELEATIRMMDTGGAMALPEE
TGAHVPERLRPYDVMSKKRMPHK

Organism name: Oncorhynchus mykiss (rainbow trout)

>gi|1207595699|ref|NC_035099.1|:14937147-14940267
Oncorhynchus mykiss isolate Swanson chromosome 23,
Omyk_1.0, whole genome shotgun sequence

GTGAAGACATCAAAACTATAAAATAACACATGGAATCATGTCGTAACCATCAAATCAAA
ATATATTTGAGATTCTACAACCTTGATGACAGCTTTGCACACTCTTGGAAATCTCAAAT
ATATAATATATTTTGATTTGTTTATTTAACACTTTTTTGGTTACTACATGATTCTATGT
GTGTTATTTAATAGTGTTGATGTCTTCACTATTATTCTACAATGTAGAACATAGCACAA
ATAAAGAAACACCCTGGAATGAGTAGGTGTCTCCAAACTTTTGACTGGTACTGTACATA
CAGTTGTACTAACTTTTTAAAGGTAGAATATATAGCATTTGTATGCTTTTCAAAATAAC

TACAGTATATTGGACCTAACATTTCAGCGTTTGCATGAAGCAGTGCCATACAAAGATTG
GAAAACCTTTCTGATCAAGAAACAATTGTCAACAAGTGACTTCAACTTCAAGTAGACAT
AGATAAATTAAGAAGATAGATTCCAACAAAATATACATTTTACTGTTTCCATATAGTTA
ATAAAACAGCCTTCATCTCTGTAATAAACATGATTGGTGTTAAGGGAAATTATTATAAT
ACCAAAAATACATTATTTATTCTGTTTATAGAGTTCTCAACTTGAAAAGTACAATTTAG
AAACAGGCATCAATTTCCTCTTGATAAGGAGTGTTCAGTCATAGTGCCCTCATATCAAT
CACTAATTAACTTGATTATTAGTATCATTTAGCAGACACTCTTATACAGAGCAACTTAC
GGTGCAATTATAGTGAAGTGTGTTGCTCAAGGGCACTTTGACAGTTTTGTCACCTAGTT
GCCTCAGGGATTTCAAACCAGTGGTTTTTCAGTTACTAGCCCAACCGCTAGGCAACACG
TTCGGCTACCTGCCACCTGGTTATTGACAATAGCAAATTTCAGGATGCTAGTTTCTGGA
ATTTCAATGTACTGTCATTTACTGTGAAATATATACACAGTATATAAATGTAATTTAAC
AAAAATTTGAATTACTTGGTTACCTGGTTATTAAGTGTTGTTAGGCTATCCTTTTCATA
CCAAAGGTACATTTATGCATTCATAACCTCTAAGGAGTGGTTATCTGCAGCTCTGTTGG
TAGAGGATGGCACTTGCAACGCCAGTATATAAGTGGGTTCGATTCTCAGGACCACCTGT
ATGTACTGTAAGCTAAATGGCAAACTGTATTAGGTAAAGATATAAGGATTGTGTCTTCA
TTTAGAATCAGTCTTATGTACTGAGTATGAAATTAAGGCTAGTGGTTGCTGTATTGCAC
AAGCATCTTGTTCCTGTACAGCCTCTTATTTTTTTTACCTTAATTTAACTAGGCAAGTC
AGTTAAGAATAAATTCTTATTTTCAATGACAGCCTAGGAACAGTGGGTTAACTGCCTTG
TTCAGGGGAAGAACAACATATTTTTGTCAGCTTGGGGATTTGATCTTGCAACCTTCCGG
TTACTAGTCCAAAGCTCTAACCACTAGGTTACCTGCCGCCCCAATCATAAGTCTGTATA
AAGTTTACATTTTTATATTCTGAGTGCAATAAATGCAATGAAAATAAACTAATTTGCCA
TTCATTTCTGAATATTTGTAAAAGATACAAAACATTGTTTATCAAACAGTGGAGGCTGG
TGGGAGGAGCTATAGGAGGATAGGCTCATTGTAATGATTGGAATAGAATAAATGGAACG
GTATCTAACACATCAAACATATGGAAACCACATGTTTGACTCCGTTTCTTTCATTCCAT
TCAGCCATTACAATGAGCCTGTCCTCCTATAGCTCCTCCCACCAGCCATTACAATGAGC
CTGTCCTCCTATAGCTCCTCCCACCAGCCATTACAATGAGCCTGTCCTCCTATAGCTCC
TCCCACCAGCCATTACAATGAGACTGTCCTCCTATAGCTCCTCCCACCAGCTGCCACTT
GTATCAAAGTAACTTTTTTATTGAGACATATATTATTTAATTTACAAACATTACATATT
TATAATATCTACACTGACGTCAACTCTTAACACATTACGAATTTGTTTGTTTCCATTAA
ATTCAGTTTGAAGCTTATGCACTAAGCAGGTCCCGTTGGTGACGTTTCGTGTCCATTAG
GCACTTAGTGTGTCACACCTGTGGAGAAGGGATTATAATCCTAATGACACAGACTGTTC
CATTTCTAACGACCCTTATAAAAAGGGCCCGTGATTATTCCC**ACCACACTGTAAGAAGG**
AATAGACCGAACGGACAGTATGTGATTCATTCATATGCTTTCAAGTACATTTAAAATTG
TTAACTGATTTATGTGTTGATAATTTCTACTAAGTGCATGTTGAATAGGTAACCATTGC
AAGACAATCCCAAACTATATTTGATTTACGATAAAATACTTTGGACTATCAAATGTAGC
TTGTATTTTACTGCCTTTTTTTTCCAG**CTTCCATG**GATCTTAGCAGCAAAACGTTTGTG
CAGGTGGTGATGTTGGCGTTGATAGCTCAGGTCACTTTCTCT**CAGCATTGGTCGTATGG**
GTGGCTACCTGGAGGAAAGAGAAGTGTTGGGGAGCTGGAGGCCACCATCAGG**GC**AAGTA
CTATTTACCCTATGTTTACCTGTATGTAAGTAACTATGTCTAACTGAACATACATGTAA
TTGTCCTTTAAGTAAATCTACTGTGCCTTGAAAACCTGTGAACATGTGTATATATATAT
ATATATATGTATATATGTATATATATATATATATATATATATATATATATATATATGAC
CCACACTCATGGGTCAGTTCCCCGTTCACAAAAATGTATCGCTCCAACAGACAGTGAGT
CACGTGGCCGTGGCTTGCTAGTCACGTGGCCGTGGTTTGCTATATAATGCAGGCAGACA
AGCAACAAGGTATTCCGTTACTGTTCGATTGAACATCAGAATGGGCAAAACGAGTGACC
AAATCGATTTTGAGCATGGTATGATTGTCGTGCCAGGCTTGCAAGATCCAGTATCTCAG
AAATGGCCGTCATTTTTGGAGTGAGATATTTGTTCAAGAAGCAGTCTTTTCAAGAGTAC
ATAGGCAAAGTTTAAGAAGTTCTTTGAGGGTGTTGCATATCGAGTATATGGGTAATAAC
ATTACAAATTCCTCTTACATATTGCTATC**AAG**ATGATGGACACAGGTGGTGTAATGGCT

CTTCCTGAGGAGACAGGTGCCCATATCCCAGAGAGACTGAGACCATATGATGTA**GT**ACG
TATTCATATTCATATTTTAGGTTAGATAGTGTATTCATTGTATTTCTAATGGTAAAGCC
CTTGTTTCGTCTAAAGATATTAAATTGATGTCTCCTTGTAATATGTTGTAAAAAGATAT
GCCAAAGTATCTGAAAGAAATGTTAAGATGAGAGTATTGGCTGACAAACGGAAAGCTTT
CCTGAAGTGGTACTTATAAATGTATGGAGACAACGTTGCTAATTTCTGATGCATCATTG
TAATATACTGTAGACACAGTATTATGCAACATAAAGTAGACTTCGGACATTGACACTTT
GTTTCCCT**AG**ATGTCGAAGAAACGAATGCCACATAAATAAAGAACTGTGAGATAATTCT
TCACAAAAAGCAAGAAGACAGCGATGATGGAGCATTCAAGAACAGTTCTGTCCATTTCC
AACTAAGATCACATTTAGATTGATGTAATTTACTTGAAGTATGAAACACTAACCTCCTG
TAAAATTGT**AATAAA**GAGTGATTGAAAGATATGTCTCTTTGTATGTCCTTACAATACAG
TATCTGCAGTGTCGTATCCCATTTTGGATTCATCAATTAGTGACCCATCATAAGATTGA
GGTGGAGTTGATAAACCATCAATTGAAAATGATTGTCTATTTGTTTTCCATGACCGACA
AGCATGATCAACTTGTAATTCAACAACCTTAGCATGACAACAGCTGGTGATTAAGTAAA
TGTATTCAAATCCAATGGTGTCATTAGATTTGAAACAGTGGTCTATGTCCCTTTTATAA
CTAGATCGTATTGCTTGTCAGTCGTTTATAATTTCATCCATAAAAACCA

mRNA:

ACCACACTGTAAGAAGGAATAGACCGAACGGACACTTCCATGGATCTTAGCAGCAAAAC
GTTTGTGCAGGTGGTGATGTTGGCGTTGATAGCTCAGGTCACTTTCTCT**CAGCATTGGT**
CGTATGGGTGGCTACCTGGAGGAAAGAGAAGTGTTGGGGAGCTGGAGGCCACCATCAGG
ATGATGGACACAGGTGGTGTAATGGCTCTTCCTGAGGAGACAGGTGCCCATATCCCAGA
GAGACTGAGACCATATGATGTAATGTCGAAGAAACGAATGCCACATAAATAAAGAACTG
TGAGATAATTCTTCACAAAAAGCAAGAAGACAGCGATGATGGAGCATTCAAGAACAGTT
CTGTCCATTTCCAACTAAGATCACATTTAGATTGATGTAATTTACTTGAAGTATGAAAC
ACTAACCTCCTGTAAAATTGT**AATAAA**GAGTGATTGAAAGAT

protein:

MDLSSKTFVQVVMLALIAQVTFS**QHWSYGWLPG**GKRSVGELEATIRMMDTGGVMALPEE
TGAHIPERLRPYDVMSKKRMPHK

Organism name: Oncorhynchus nerka (sockeye salmon)

>gi|1681300709|ref|NC_042562.1|:27128556-27131977
Oncorhynchus nerka isolate On170113-E2 linkage group LG28,
Oner_1.0, whole genome shotgun sequence

TTACTGACCTTACAAGTGACTTAAGTGCCATTTAATATTTGCTGTGTATGATTTGTTTT
AATCAACAGTATTGTTTTTTTCTTTATTTATGCTATTTTCTACATTGTAGAATAATAGT
GAAGACATCAAAACTATGAAATAACACATATGGAATCGTGTTAATCAAATCAAAATATA
TTTGAGATTCTACAACCACCCTTTGCCTTGATGACAGCTTTGCACACTCTTGGAAATCT
CAAATATAAAATATATTTTGATTTGTTTTTTTAACACTTTTTTTTGGTTACTACATGAT
TCTATATGTGTTATTTAATAGTGTTGATGTCTTCACTATTATTCTACAATGTAGAAAAT
AGTACAAATAAAGAAACACCCTGGAATGAGTAGGTGTCTCCAAACTTTTGACTGGTACT
GTACATACAGTTGTAATAACTTTTTAAAGGTAGAATATATATATATATATATATATATA
TATATATATATATAGCATTTGTATGCTTTTCAAAATAACAGTATATTGGACCTAACATT

TCAGCGTTTGCATGAAGCAGTGCCATACAAAGATTGGAAAACCTTTCTGATCAAGAAAG
AATTGTCAACAAGTGACTTCAACTTCAAGTAGACATAGATAGCTAAGCCAGCAGACTGT
TTTTATGTTTTTCAAGACTAAATTAAGAAGTTAGATTTCAACAAAATATACATTTTACT
GTTTCCATATAGTTAATAAAACAGCCTTCATCTCTGTACATGATGTACATGAACATGAT
TGGTGTTAAGGGAAATTATTATAATAACCAAAAATATATTATTTATTCTGTTTAGAGTT
CTCAACTTGAAAAGTATAATTTAGAAACAGGCATCAATTTCCTCTTGATAAGGAGTGTT
CAGTCATAGTGCCCTCATATCAATCACTAATTAACTTGATTATTAGTATCATTTAGCAG
ACACTCTTATACAGAGCAACTTACGGTGCAATTATGGTGAAGTGTGTTGCTCAAGGGCA
CTTTAACAGTTTTGTCACCTAGTTGCCTCAGGGATTTCGAGCCAGTGGTTATTCAGTTA
CTAGCCCAACCGCTAGGCAACACGTTCGGCTACCTGCCACCTGGTTATTGACAATAGCA
AATTTCAGGATGCTAGTTTCTGGAATTTCAATGTACTGTGAAATATATACACAGTATAT
GATCATAAATGTAATTTAATACAAAATTGAATTACTTGGTTACCTTGTGTGTTGTGAGG
CTATCCTTTTCATACCAAAGGTACATTTATGCATTCATGACCTCTAAGAAGTGGTTATC
TGTAGCTCTGTTGGTAGAAGTTGGCACTTGCAACGCCAGTATAGTAGTGGGTTCGATTC
TCAGGACCACCTGTATGTACTAATAACTGTAAGCTAAATGGCAAACTGTATTAGGTAAA
GATATAAGGATTGTGTCTTCATTTAGAATCAGTCTTATGTACTGAGTATGAAATTAAGG
CTAGTGGTTGCTGTAGTGCACAAGCATCTTGTTCCTGTACAGCCTCTTATATTATTTTT
TTTACCTTAATTTAACTAGGCAAGTCAGTTAAGAATACATTCTTATTTTCAATGACAGC
CTAGGAACAGTGGGTTAACTGCCTTGTTCAGGGGAAGAACAACACATTTTTTACCTTGT
CAGCTTAGGGATTTGATCTTGCAACCTTTCGGTTACTAGTCCAAAGCTCTAACCACTAG
GTTACCTGCCGCCCCAATCATAAGTCTGTATAAAGTTTACAATTTTCTATTCTGAGTGC
AATAAATGCAATGAAAATAAACTAATTTGCCATTCATTTCTGAATATTTGTAAAAAAAA
AATACAAAACATGTGTATCAAACAGTGGAGGCTGGTGGGTGGAGCTATATGTGGACGAG
CTCATTTTAATGGTTGAAATGGAACTAATGGAACGGTATCAAACATATGGAAAACACAC
GTTTGACTCCGTTCCTTTTATTCCATTTCAGCCATTACATTTTTTTTATTATTATTTTT
TGAGCCTGTCATCCTATAGCGCCTCCCACCAGCTGCCACTTGTATCAAAGTAAGTTTTT
TTTATTGAGACAAATATATTATTTAATTTACAAACATTACATATTTATAATATCTACAC
TGACGTCACCTCTTAACACATTACGAATTTGTTTGTTTCCATTAAATTCAGTTTGAAGC
TTATGCACTAAGCAGGTCCCGTTGGTGACGGTTAGTGTCCATTAGGCACTTAGTGTGTC
ACACCTGTGGAGAAGGGATTCTAATCCTAATGACACAGACTGTTCCATTTCTAACGACC
CTTATAAAAAGGACCCATGATTATTCCC**ACCACACTGTAAGAAGGAATACACCGAACGG**
ACAGTATGTGATTCATTCATATGCTTTCAAGTACATTTCAAATTGTTAACTGATTTATG
TGTTGATAATATCTACTAAGTGCATGTTCAATAGGTAATCATTGCAAGACAATCCCAAA
TTACATTTGATTTACGATAAAATATTTTGGACTATCAAATGTAGCTTGTATTTTACTGC
CTTTTTTTTCCAG**CTTCCATG**GATCTTAGCAGCAAAACGGTTGTGCAGGTGGTGATGTT
GGCGTTGATAGCTCAGGTCACTTTCTCT**CAGCATTGGTCGTATGGGTGGCTACCTGGA**G
GAAAGAGAAGTGTTGGGGAGCTGGAGGCCACCATCAGG**GC**AAGTACTATTTACCCCATG
TTTACCTGTATGTAAGTAACTATGTCTACCTGAACATACATGTAATTGTCCTTTAAGTA
GATCTACTGTGCCTTAACCTGTGAAGATATATATATACATACACTCATGGGTCAGTTCC
CCGTTCACAAAAATGTATCGCTCCAACAGACATTGAGTCATGTGGCCGTGGTTTGCTAG
TCATGTGGCCGTGGTTTGCTAGTCATGCGGCCGTGGTTTGCTAGTCATGTGGCCGTGGC
TTGCTAGTCATGTGGCCGTGGCTTGCTAGTCATGTGGCCGTGGCTTGCTAGTCATGTGG
CCGTGGCTTGCTAGTCATGTGGCCGTGGCTTGCTAGTCATGTGGCCGTGGCTTGCTAGT
CATGTGGCCGTGGTTTGCTAGTCATGTGGCCGTGGCTTGATAGTCATGTGGCCGTTTGG
CTTGCTAGTCATGTGGCCGTGGCTTGCTAGTCATGTGGCCGTGGCTTGCTAGTCATGTG
GCCGTGGCTTGCTAGTCATGTGGCCGTGGCTTGCTAGTCATGTGGCCGTGGTTTGCTAG
TCATGTGGCCGTGGCTTGCTAGTCATGTGGCCGTGGTTTGCTATATAATGCAGGCAGAC
AAGCAACAAGGTATTCCGTTACTGTTCGATTGAACATCAGAATGGGCAAAACGAGTGAC

CAAATCGACTTTGAGCATGGTATGATTGTCGTGCCAGGCTTGCCAGATCCAGTATCTCA
GAAATGGCCGTCATTTTTGGAGTGGGATAATTGTTCAAGAAGCAGTCTTATCAAGAGTA
CGTAGGCAAAGTTTAAGAAGCTCTTTGAGGGTGATGCATATCGAGTATATGGGTAATAA
CATTACAAATTCCTCTTACATATTGCTATC**AAG**ATGATGGACACAGGTGGTGTAATGGC
TCTTCCTGAGGAGACAGATGCCCATATCCCAGAGAGACTGAGACCATATGATGTA**GT**AA
GTATTCATATTCATATTTTAGGTTAGATAATGTATTCATTGTATTTCTAATGGTAATGT
CCTTGTTTCGTCTAAAAAATATTGAATTGATGTCTCCTTGTAATATGTTGTAAAAAGAT
ATGCCAAAATATCTAAAAGAAATGTTAAGATGAGAGTATTGGCTGACAAACGGAAAGCT
TTCCTGAAGTGGTACTTATAAATGTATGGAGACAATGTTGCTCATTTCTGATACATCAT
TGTAATATACTGTAGACACAGTATTATACAACATAAAGTAGACTTCTGACATTGACACT
GTTTCCCT**AG**AAACGGATGCCACATAAATAAAGAACTGTGAGATAATTCTTCACAAAAA
GCAAGAAGACAGCGATGATGGAGCATTCAAGAACAGTTCTGTGCATTTCCAACTAAGAT
CACATTTAGATTGATGTAATTTACTTGAAGTATGAAACATTAACCTCCTGTAAAATTGT
AATAAAGAGTGATTGAAAGATATGTCTCTTTGTATGTCCTTACAATACAGTATCTGCAG
TGTCCTATCCCATTTTGGATTCATCAATTAGTGACCAGTCATAAGATTGAGGTGGAGTT
GATAAACCATCAATTGAATATTATCGTATATTTGTTTTCCATGACCGACAAGCATGATC
AACTTGTAATTCAACAACCTTAGCATGACAGCAGCTGGTGATTAAGTAAATGTATTCAA
ATTCAATGGTGTCATTAGATTTGAAACAGTGGTCTATGTCGCTTTTATAACGAGATCGT
ATTGCTTGTCAGTCGTTTCATCCATAAAAACCACAAGGAACATTTGCATTTTGAAACAG
GACATTTATTTTTACCTCAAATGTATGTGAGTATGCCACCTGTTGAATACTTGCATTTT
ATTTAGTTAAAAATAATTCTGAAATGTCAAGAGGGTTGACATTTTTCGAATAGTTTTGC
CAGGATCATAGACATCACTAAAAGACTGTGTAAAACTTTAATGTCGTTCAATAAATTGG
TTATACACATTTTCATTTCATATCAGGCAAGGAAAGGAATCTGCAGGGGGCATTTGTTT
CAAATAGAACTCATAATCTGGCAAAGCCCAACAGACATGGTACCTCACTAATCGCGTAG
TGCATTAGGGGCCCATGATTCATGAACTGTGCCAACACGAGGGAGACCCTTCATTGTTA
GCGTGTGGTTGTAAATGCCCACCAATGAACCCAAACCCGCCGGTACTTTAGGGAAAAGG
TGAACATTTTTAAAGCACATAGAATTAGAAAGAAATTAAACCACTTTCCATTGCACTTT
CAGAACAATGGTTCTTCATTTGTGATACCTAACATCGTTCGTTTAATATTCATGGTTAC
AATTTGCATAATGCAATCATTTTCTTGGATAAGGAAAAACTTCACTATTTTACTTCAAA
AAATTATTATTATTTTTTTTTTACTTGAGAACTTGCTCCAACTATTGCAACAAAGATAT
ACAGTACCAGTCAAAAGTTTGGACACACCTAATCAGTCAAGGGTTTTTCTTTATTCAGA
GACAGCAGGGGTTCAAACATACATTTCCTACATTTGAATATAAAATTTGATTTTATCAA
ACAAAACTATGCTACAATACCTTTTATCTCTGGGACCGTCAAGATGACAAATCAGAGCA
AGATTACTGAATGTAAGTATATTATTTACCTTCAGAGGTGAATGTATCAAACCAGTTGC
CGTGATAAAAGTGTTTTGTTGTTGTGCACTCTCAAACAATAGCATGGTATTTTTTCACT

mRNA:

ACCACACTGTAAGAAGGAATACACCGAACGGACACTTCCATGGATCTTAGCAGCAAAAC
GGTTGTGCAGGTGGTGATGTTGGCGTTGATAGCTCAGGTCACTTTCTCT**CAGCATTGGT**
CGTATGGGTGGCTACCTGGAGGAAAGAGAAGTGTTGGGGAGCTGGAGGCCACCATCAGG
ATGATGGACACAGGTGGTGTAATGGCTCTTCCTGAGGAGACAGATGCCCATATCCCAGA
GAGACTGAGACCATATGATGTAAAACGGATGCCACATAAATAAAGAACTGTGAGATAAT
TCTTCACAAAAAGCAAGAAGACAGCGATGATGGAGCATTCAAGAACAGTTCTGTGCATT
TCCAACTAAGATCACATTTAGATTGATGTAATTTACTTGAAGTATGAAACATTAACCTC
CTGTAAAATTGT**AATAAA**GAGTGATTGAAAGAT

protein:

MDLSSKTVVQVVMLALIAQVTFS**QHWSYGWLPG**GKRSVGELEATIRMMDTGGVMALPEE
TDAHIPERLRPYDVKRMPHK

GnRH3 Gene2

Organism name: Thymallus thymallus (grayling)

>gi|1594667021|gb|CM015034.1|:15191110-15193763 Thymallus thymallus isolate TTM2012 chromosome 6B, whole genome shotgun sequence

ATACACAACTTGTCATGAGAGTTTGTCATTGTATAACTGACCTTTCAAGTGAAAAGTGA
CTTAACAAATTAGCATTCAATATTTGCTGTGTATTTTGTCTGTTTCCTCATATCAACAG
TATATTGAGTATCCAATGGAAAACAATCAATTTGGACAACTTTGAATTGCCATCTGTTT
CAAACAGCAACTTTTTAAAAACTATATTGGACCTACCATTTCAGCATTTGATGTTTGAA
TGAAGCAGTGCCATACAAAGATTAGAAAAGCATTCTGATCAAGTTAAAATTGTCACCAA
GTGACTTCAACTTCAAATATACATAGCTAAGGCAGCAGATTGTTTTATGTTTTTCAAGA
CTAACCTAAAAAGGGAGAATATGTTGATTTTCATGATGTCCTTATAGTGTATAAAACAT
AAACATGATTGGGATTAAGGCAAAGGATTATAATATCAATATACCCCCCAAAATGTGTA
TTGAATGTATAGAGTTCTCAAATAGAAAAGTGTAATTTAGAGACTGGCAGCAATTTCCT
CTTGGTAAGGACTTGGTAGCCACGGTGCTCTCATATCACTAAATAACTGGGTTATTGAT
AATAACAATTGATTCATGGAAGTTTCAGGAAGATATTTTCTTGAATTTCAATGTTCTGT
ATTTTACTATGAAATCTATATTTTAGCCATGATCATGAATAAGTAGAGTTTAAATGACC
TGGTTACCCAGTTATTAAGTGTCAAGGCACTCTACAGGGCTATCCTTTTCATGGTAAAG
GTACGTTTATGGATTCATAGCCTGTTAGGTAAAGAGACAAGTGTAAGGATTATGTAATC
GTATAAGGGTCTTCATACAGTACAGAACATTTAGAATCAGTCTTATTTACAGAGTATGA
AATTGAAGCCTAATGTAGTGCTCAAGCATCTTGTTCCTGTATATCTCATCATAAGTCTG
TATGAAGTTGACATTTTATTTGTAAATGCTGGTCGCAATAAATGCATTGAAAATAAACT
AATTGGCCATTCATTTCTGAATATTTTTAAATGATACAAAACATGGGTATAAAAGTCAC
TTTTTTTTAGTTCTGTTTGATTTACAAACATGACATATTTTAGAATATGTACACTGACT
TCACCTCAACACATTCCGAATGTTTGTTTCCATCGAATGCAGTTTGAAGCTTATGCACT
AAGCAGGTCCCGTTAGTGACATTAAGTGTCCATTAGGCACTTAGAATTTCACACCTGTG
GAGTAGGGATTATAATCCTGATGACACAGACTGTTCCATGTCTAACGACCCCTTTAAAA
GGGACCCATGATTATTCCC**ACCACACTGTAGGAAGGAATACACAGAACGGAGACG**GTAT
GTGATTCATATTTAAAATGTTTAACTAATGTGCATTTGTGGGTAGTTCATATATACTGT
ACTATGTACATATTCAATAGGTAATCTTTGCAATACAATCCCAAACGTATCTTTGGTGT
AAAAATACCTTATTTTTTACTATCAAATGTAGCTTGTATTTGATTAGCTTTCTTTCTAG
CTCCCATGGATCTTAGCAGCAGAACGGTCGTGCAGGTGGTGGTGTTGGCGATGGTAGCT
CAGGTTACTCTCTCT**CAGCATTGGTCGTATGGGTGGCTACCTGGA**GGGAAGAGAAGTGT
TGGGGAGCTGGAGGCCACCATCAGG**GC**AAGTACTATTTACATCTACCTGTAACTACTGT
AAGTAACCATGTCTACATGTACATACTGTACATTTATTTGTCCTTTAAGTAAATCTGTG
TCTTGAAAACCTGTGAACATTTAAATGTAACATTTATAGGGATATATGAATATTTGTTC
AAGAAGCAGTCTTTTCAAGAGTACATAGGCAATGTTTATTAATTAAGCTCTGTAAGGTT
GTTGCATAATGGGTATATATATGGGTAATAGTATTAGAAATTCCTCTTACATATTTCTA
TC**AAG**ATGATGGACACAGGAGGTGTAGTGGCTCTTCCTGAGGAGACAAGCGCCCATGTC
TCAGAGAGACTGAGACCATATGATGTA**GT**AAGTATTCATATTAATATATTAGGTTAGAT

ATTGTCTTCATTGTATTCCTAATGGTCAGTTAATTATGTTTCGTCTGAAGATACTGAAT
TGATGTCTCCTTGTAAAATATGTTTTGAACAATATACCAAAGTATCTGAAATACATTTT
AAGATGAGAGTATTGGCTGACAAATGGAAAGCTCCCCTAAATTTAGACAAGTTAAACTT
ATAAATGTATGGAGACAATGTTGCTCATGTCTGACACATCAGTATAAAATAGATACAGT
ACTATACAACATAAAGTAGACTTCTGAAGCTAACACTTTGACTTCCCT**AG**ATATCGAAG
AAATGGATGCCCCGTAAATAAAGAACTGTGAGACCATTATTCACAGAAGAAGCAAGAAG
ACAACATCAGGCAGACATTCAACATCACTATCAACATCAATGTTGGAGCATTCAACTAC
AGTTCCGTCCATTTCCAACTAACATCACATTTAGATTGATGTTATTTACTTGAAGTATG
AAACTTAATCCTCCTGTAAAATTGT**AATAAA**GAGTGATTGAAAGATGTCTCTTTGTATG
TCTGTTGAACGTCCTTACAATATAGGAGCTTTACTGTCCTAATCAATTATTGACCCATC
ATGATATTGAGGTGGAGTTGACAGACCATACATTGATTATTACCATACATTTGTTTTCT
CTGACCGTCAAGCCTGATCAACATGTGATACAACAATCTTATCATGACAAAAGCTGGTG
ATTAAGTCAATTTATAAAAAAAAAAAAAATCTATGGTGTCATTAGATTTTAAACAGTGGT
TTATGTCCCTTTTGTAATTAGATTATAGTTAATGCTTGTCAGTCATGTGTAATTTCTGA
GAAAATCCACAATACCAGAACATTTGTTTCACCTCAAATTTATGTGAGAGTATGTCACT
TGTTGAATAATTGAATTTACATAGTTAAAAATAATTCAAGTGTCATGGGGGTTGACATT
TTTCTCCTAGTTTTGCCAGGATCATAGGCCTCACAAAAAAAAAAGTGTTCACTTTCATG
TAGTTCAATAAATTGCTGTTAATTATAAAAATTGGAATTGCATATCAGGCAAGGAAAGG
AATTAGCAGGGGCATATGTTTTAAAGAGAACTAATTTTCTGGCAAAGCCCCCCAGACAG
GGTACGGTACTAATCGCGTAGTGCATTAGGGGCCCATGATTCATGAACTGTACTGACAC

mRNA:

ACCACACTGTAGGAAGGAATACACAGAACGGAGACGCTCCCATGGATCTTAGCAGCAGA
ACGGTCGTGCAGGTGGTGGTGTTGGCGATGGTAGCTCAGGTTACTCTCTCT**CAGCATTG**
GTCGTATGGGTGGCTACCTGGAGGGAAGAGAAGTGTTGGGGAGCTGGAGGCCACCATCA
GGATGATGGACACAGGAGGTGTAGTGGCTCTTCCTGAGGAGACAAGCGCCCATGTCTCA
GAGAGACTGAGACCATATGATGTAATATCGAAGAAATGGATGCCCCGTAAATAAAGAAC
TGTGAGACCATTATTCACAGAAGAAGCAAGAAGACAACATCAGGCAGACATTCAACATC
ACTATCAACATCAATGTTGGAGCATTCAACTACAGTTCCGTCCATTTCCAACTAACATC
ACATTTAGATTGATGTTATTTACTTGAAGTATGAAACTTAATCCTCCTGTAAAATTGT**A**
ATAAAGAGTGATTGAAAGAT

protein:

MDLSSRTVVQVVVLAMVAQVTLS**QHWSYGWLPG**GKRSVGELEATIRMMDTGGVVALPEE
TSAHVSERLRPYDVISKKWMPRK

Organism name: Coregonus sp. 'balchen' (whitefish)

>gi|1711367472|emb|LR664380.1|:21754100-21756276 Coregonus sp. 'balchen' genome assembly, chromosome: 37

ATTTTCATTATGTCCGTATAGTGTATAAAACATAAACATGATCGGGATTAAGGCAAAGG
ATTATAATACCAATATACCCCACCCAAAAAATGTATATTGAATATATAGAGTTCTCAAC
TAGAAAAGTGTAATTTAGAGACGGGCAGCAATTTCCGCTTGGTAAGGACTTTATAGCCA
CAGTGCTCTCATATCACTAAATAACTGGGTTATTGATAATAGCAATTGATTCATGAAAG

TTATTGATTCTTGAATTTCAATGTACTGTAGTTTACTGTGAAATCTATATTTTAGCAAT
GATCATAAATACAAATTAATAGAATTTAAATTCAAATTACCTGGTTACCCAGTTATTAA
GTGTTAAGATACTCTACAGGGCTATCCTTTTCATGCCAAAGGTACATTTATGGATTCAT
AGCCTGTTAGGTGTAATGATTATGTGTAAGGATTATGTAAGGATTATACAAGTGTAAGG
ATTATGTAATCATATAAGGGTCTTCATACAGAACATTTAAAATCAGTCTTATGTACAGA
GTATGAAATTGAGGCCTAATGTAGTGCTCAAGCATCTTGCTCCTGTATATCTCTTGTCA
TAAGTCTGTATGAAGTTGACATTTTATTTGTAAATGCTGGTCGCAATAAATGCATTGAA
AATAAACTAATTTGCCATTCATTTCTGAATATTTGTAAAAGATACAAAACATGGGTATC
AAAGTCACTTTTTTGTTGACCTGCAGAAATTGTTTCATTTACAACATATTTTAGAATAT
GTACACTTACTTCACCTCTTAACACATTACAAATATGTTTGTTTCCATCGAATGCAGTT
TGAAGCTTATGCACTAAGCAGGTCCCGTTGGTGACATTTAGTGTCCATTAGGCACTTAG
TGTGTCACACCTGTGGAGAAGGGATTATAATCATGATGACTCAGACTGTTCCATGTCTT
AACGACCCCTATAAAAGGGACCCATGATTATTCCC**ACCACACTGTAGGAAGGAATATAC**
AGAACGGAGACAGTATGTGATTCATATTTAAAATAGTTAACTAATGTGCATTTGTGGGT
AGTTCATATACACTGTACTATGTGCATATTCAATAGGTAATCATCTTTGTTGTAAAAAT
ACAAAATGTAGCTTGTATTTGACTAGCTTTCTTTCCAG**CTCCAATG**GATCTTAGCAGCA
GAACGGTCGTGCAGGTGGTGGTGCTGGTATTGGTAGCTCAGGTCACTCTCTCT**CAGCAT**
TGGTCGTATGGGTGGCTACCTGGAGGGAAGAGAAGTGTTGGGGAGCTGGAGGCCACCAT
CAGG**GC**AAGTACTATTTACCTCTACCTGTAACTACTGTAAGTAACTATGTCTACATGTA
CATACTGTACATGTAATTGTCCTTTAAGTAAATCTACTGTGTCTTGAAAACCTGTGAAC
ATTTAAATGTAACATTTACTAATTAAGCTCTGTAAGGGTGTTGCATAAGGGGTATATGG
GTAATAGCATTACAAATTCCTCTTACATATTGCCATC**AAG**ATGATGGACACAGGAGGTG
AAGTGGCTCTTCCCGAGGAGACAAGCGCCCATGTCTCAGAAAGACTGAGACCATATGAT
GTA**GT**AAGTATTCATATTAATTTATTAGGTTAGATATTGTATTAATTGTATTCCTAATG
GTCAATTATTTATGTTTCGTCTGAAGATACTGAATTGATGTCTCCTTGTAAAATATGTT
GTAAACGATATACCAAAGTATCTGAAATACATTTTAAGATGAGAGTATTGTCTGACAAA
TGGAAAGCTTCCCTGAATTTAGAGAAGTGATACTTATAAATGTATGGAGACAATGTTGC
TCATGTCTGACACATCAGTACAATATAGATACAGGCCTATGCAACATAAAGTAGACTTC
TGAAGTTAACACTTTGATTTCCCT**AG**ATATCGAAGAAATGGATGCCCCATAAATAAAGA
ACTGAGACCATTATTCACAAAAGAAGCAAGAAGACAACATCAAGCAGACATTCAGCATC
ACTATCAACATCAATGATGGAGCATTCAACTACAGTTCTGTCCATTTACAACTAACATC
ACATTTAGATTTATGTTATTTACTTGAAGTATGAAACTTTAACCTCCTGTCAAATTGT**A**
ATAAAGAGTGATTGAAAGATAAGTCTCTTTGTATGTCTGTTGAACGTCCTTACAATACA
GGAGCTTTAGTGTCCTATCCAATTTTATTGAGGTGGAGTTGGCAGACCATAAATGGATT
ATTACCATACCATTTGTTTTCTATGACCGACAAGCATGATCAACTTGTGATACAACAAT
CTTAGCATGACAACAGCTGGTGATTAAGTCAATTTATAAAATATCTATGGTGTCCTTAG
ATTTGAAACAGTGGTTTATGTCCCTTTTGTAATTAGATTATAGTTATTGCTTGTCAGTC
ATGCATAATTTCAATGAGAAAATCCACAATACCAGGATATTTGTTTCACCTCAAATTTA
TGTGAGAGTATGTCACTTGTTGAATAATTGAATTTTGCATAGTTAAAAATAACTCTGAA
GTGTCATGAGGGTTGACATTTTTCTCCTAGTTTTGCCAGGATCATAGGCATCACAAAAA
GAAAATGTGTTAACTTTCATGTCGTTCAATAAATTGCTGTTAATTATAAAAATTGGAAT
TGCATATCAGGCTAGGAAAGGAATTAGCAGGGGCATATGTTTTAAAGAGAACTAATTTT
CTGGCAAAGCCCCACAGACAGGGTACCGTACTAATCGCGTAGTGCATTAGGGGCCCATG

mRNA:

ACCACACTGTAGGAAGGAATATACAGAACGGAGACACTCCAATGGATCTTAGCAGCAGA
ACGGTCGTGCAGGTGGTGGTGCTGGTATTGGTAGCTCAGGTCACTCTCTCT**CAGCATTG**

GTCGTATGGGTGGCTACCTGGAGGGAAGAGAAGTGTTGGGGAGCTGGAGGCCACCATCA
GGATGATGGACACAGGAGGTGAAGTGGCTCTTCCCGAGGAGACAAGCGCCCATGTCTCA
GAAAGACTGAGACCATATGATGTAATATCGAAGAAATGGATGCCCCATAAATAAAGAAC
TGAGACCATTATTCACAAAAGAAGCAAGAAGACAACATCAAGCAGACATTCAGCATCAC
TATCAACATCAATGATGGAGCATTCAACTACAGTTCTGTCCATTTACAACTAACATCAC
ATTTAGATTTATGTTATTTACTTGAAGTATGAAACTTTAACCTCCTGTCAAATTGT**AAT**
AAAGAGTGATTGAAAGAT

protein:

MDLSSRTVVQVVVLVLVAQVTLS**QHWSYGWLPG**GKRSVGELEATIRMMDTGGEVALPEE
TSAHVSERLRPYDVISKKWMPHK

Organism name: Salmo salar (Atlantic salmon)

>gi|925216706|ref|NC_027317.1|:19443955-19445994 Salmo salar isolate Sally breed double haploid chromosome ssa18, ICSASG_v2, whole genome shotgun sequence

GTTTTCTTTTGCTCATCTTAATCTTTTCTTTTTATTGGCCAATTCTGAGATATGGCTTT
TTCTTTGCAACTCTGTGTAGAAGGCCAGCATCCCGGAGTCGCCTCTTCACTGTTGACAT
TGAGACTGGTGTTTCTGGCCATTTTGAGCCTGTAATCGAACCCAGAAATACTGATGCTC
CAGATACTCAACTAGTCTAAAGAAGGACAGTTTTATTGCTTTTTTCACTAGGACAACAG
TTCTCAGCTGTGCTAACATAATTGCAAAAGGGTTTTCTAATGATCAATTAGCCTTTTAA
AATGAAAAACTTGGATTAGCTAACACAATGTGCCATTGGAACACAGGTGTGATGGTTGC
TGATAATGGGCCTTTTAACGCCTAAGTATGTAGATATTCCATAAAAAATGTGCAGTTTC
CAGCTACGATAGTCATGTACAACATTAACAATGTCTACACTGTATTTCTGATCAATTTG
ATGTTATTTTAATGGACAAAAATGTGCTTTTCTTTCAAAAACTAGGACATTTCTAAGTG
ACCCCAAACTGTTGAACGGTAGTGTTCACTGACTTCACCTCTTAACACATTACAAATAT
GTTTGTTTCCATCAAATGCAGTTTGAAGCTTATGCACTAAGCAGGTGCCGTTAGTGACA
TTTAGTGTCCATTAGGCACTTAGTGTGTCACACCTGTGGAGAAGGGATTCTAATCCTGA
TGACACAGACTGTTCCATGTCTAACGACCCCTATAAAAGGGACTCATGATTATTCCC**AC**
CACAGTGTATGAAGGAATATACAGAACGGAGAAAGTATGTGATTCATATAACTATATTT
AAAATTGTTAACTAATGTGCATTTGTGGGTAGTTCATATATACTGTACACTGTGCATAT
TCAATAGGTAATCATTGCAAGATGATCCCAAACTTATCTTTGATGTAAAAAATAAATAT
TTTTGACTATCACATGTAGCTTGTATTTCACTAGCTTTCTTTCCAG**CTCCCATG**GATCT
TAGCAACAGAACGGTTGTGCAGGTGGTGGTGTTGGCGTTGGTAGCTCAGGTCACTCTCT
CT**CAGCACTGGTCGTATGGCTGGCTACCTGGA**GGGAAGAGAAGTGTAGGGGAGCTGGAG
GCCAGCATCAAG**GC**AAGTACTATTTACCTCTACCTGTAACTACTGTAAGTAACTATGTC
TACATGTACATACTGTGCATGTAATTGTCCTTTAAGGAAATCTACTGTGTCTTGAAAAC
CTGTGAACATTTAAATGTAGCATTTATAGGGATAGTTTGTGTGGAATATTTGTTCAAGA
GGCCTTTTCAAGAGTACATAGGCAATGTATATTAATTAAACTCTGTAAGGGTGTTGCAT
AAAGGGTATATGGGTAATATTATTGCACATTCCTCCTACATATTGCTATCC**AG**ATGATG
GACACAGGAGGTGTAGTGGCTCTTCCTGAGGAGACAAGTGCCCATGTCTCAGAGAGGCT
GAGACCATATGATGTA**GT**AAGTAGTCATATTCATTTATTAGGTTAGATATTGTATTCAT
TGTATTCCTAATGGTCAATTATTTATGTTTCATCTGAAGATGCTAAATTGTCTCCTTGT
AAAATATGTTGTAAACGATATACCAAAGTATCTGAAATAAATGTTTAGATGAGAGTATT

GGCTGACAAATGGAAATCTTCCCTGAATTTAGAGAAGTGATAGCTATAAATGTAGGGAG
ATAATGTTGCTCATGCCTGACACATCAGTATAATATAGATATACAACATAAAGCAGACT
TCTGAAGTTAACACTTTGATTTCCC**TAG**ATATTGAAGAAATGGATGCCCCATAAATAAA
GAACTGTGAGACCATTATTCACAAAAGAAGCAAGAAGACAACATCAAGCAGACATTCAG
CATCACTATCAACATCAATGATGGAGCAACTACAGTTCTGTCCATTTCCAACAAACATC
ACATTTAGATTGATGTTATTTACTTGAAGTATAACACTTTAACCTTCTGTAAAATTGT**A**
ATAAAGAGTGATTCAAAAAGAGAAGTATCTTTGTGTGTCTATTGAACGTCCTTACAATA
CAGGAGCTTAAGTGTCATTTCCAATCTTGGATTAATCAATTCGTGATCCGTCCACATTT
TTAGGTGCAGTTGACAGACCATGAATTGATTATTACCATGCATTTCTTTTCTATGACCG
ACAAGCATGATCAACTTGTGATACAACAATCTTAGCATAACAACAGCTGGTAATTAAGT
CAATTTATAGAACATCTATGGTGTTATTAGATTGAAACAGGGGTTTATGTCCCTTTTGT
AATTAGATTATAGATTATTGCTTGTCAGTCATGCATCATTTCAAAGAGAAAATCCACAA
TACCAGGACATTTGTTTCACCTCAAATTTCTGTAAGAGTATATGTCACTTGTTGAATAA
TTTAATTTTGCATAGTTAAAAATAATTCTGA

mRNA:

ACCACAGTGTATGAAGGAATATACAGAACGGAGAAACTCCCATGGATCTTAGCAACAGA
ACGGTCGTGCAGGTGGTGGTGTTGGCGTTGGTAGCGCAGGTCACGCTCTCT**CAGCACTG**
GTCGTATGGCTGGCTACCTGGAGGGAAGAGAAGTGTTGGGGAGCTGGAGGCCACCATCA
AGATGATGGACACAGGAGGTGTAGTGGTTCTTCCTGAGGAGACAAGTGCACATGTCTCA
GAGAGACTGAGACCATATGATGTAATATTGAAGAAATGGATGCCCCATAAATAAAGAAC
TGTGAGACCATTATTCACAAAAGAAGCAAGAAGACAACATCAAGCAGACATTCAGCATC
ACTATCAACATCAATGATGGAGCAACTACAGTTCTGTCCATTTCCAACAAACATCACAT
TTAGATTGATGTTATTTACTTGAAGTATAACACTTTAACCTTCTGTAAAATTGT**AATAA**
AGAGTGATTCAAAAAGAG

protein:

MDLSNRTVVQVVVLALVAQVTLS**QHWSYGWLPG**GKRSVGELEATIKMMDTGGVVVLPEE
TSAHVSERLRPYDVILKKWMPHK

Organism name: Salvelinus spp.

>gi|1340979625|ref|NC_036865.1|:16779745-16782458
Salvelinus spp. isolate IW2-2015 linkage group LG25,
ASM291031v2, whole genome shotgun sequence

AACACCCTATTAAGACACTTTATGTTGGTGTTTCCTTTATTTTGGCAGTTACCTGTATA
TGTTTGCTTGTCTGTCTGTTTGTTAAAAAGAGCCCAAATGAACATTTATTATTATCTCT
ATTTAGTCACGGACGTTTCAACTAGCTTTGTCTCCTCAGCATTTGTATGTCAAATGTTT
TGAATTGAAAACGTAAGGCCAAAACCGGTGTGAAGGGTTTAATTGAATAGGTTCTCGGA
CTCAAGACAATACTGAAATGTATGTTAATATGTACATACTGTGTGTATTAATTAACTTA
TGCATATAATCTTTTTCTATTTATATAATGTAGAATTTTATGTACATTTTGAGTTTTAC
TGATAGTCTATGCTAAAAAAATGACCATGAGATTGCCTTTCGTTGCCTGTGGTCTGTAC
TGTAGCCATGGCACATACTGTAGAACTCCTGCTGTTGTAAAAATACACAACCTGTCATG

AGAGTTTGTGACAAGTGACTTAACAAATGAACATTCAGTATTTGGTGTGTATGTTGTCT
GTTTCATCATAACAGTCTATTGAGTATCCAATGGAAAACAATCAATTTGGACAACTTTG
AATTGCCATCTGTTTCAAACAGCAATTTTGATCTGACCTCACTAACAAATGGCTGTTCA
TATGGACACGGTTGTAATAACTTTTGTACAACTTTTTTTAAACTATATTGGACCTACC
ATTTCAGCATTTGCATGTTTGAATGAAACAGTGCCATACAAAGATTAGAAAAATGTTCT
GATCAAGTTAAAATTGTCACCAAGTGACTTCAACTTCAATATACACAAAATGTGTATTG
AATGTATAGAGTTCTCAACTAGAAAAGTGTAATTTAGAGCCAGGCAGCAATTTTCCACT
TGGTAAGTATTTATAGCCACAGTGCTCTCATATCACTAAATAACTGGGTTATTGATAAT
AGTAATTGATTCATGGAAGTTTCAGGAAGATATTTTATTGAATTTCATTGTGCTGTAGT
TTACTGTGAAATCTATATTTTAGCAATGGTCATAAATAAATAGAATTTAAATTCAAATT
ACCTGGTTACCCAGTTATTAAGTGTTAAGGCACTCCACAGGGCTATCCTTTTCATGCCA
AAGGTACATTTATGGATTCATAGCCTGTTAGGTAAAGATACAAGTGTAAGGATTATGTA
ATCACATAAGGGTCAGAACATTTAGAATCAGTCTTGTGTACAGAGTATGAAATTGAGGC
ATAATGTAGCGCTCAAGCATCTTGTTCCTGTATATCTCTTGTCATAAGTCTGTATGGAG
TTAACATTTAATTTGTAAAATCTGTCCGCAATAAATGCATTGAAAATAAACAAATTTGC
CATTAATTTCTGAATATTTGTAAAAGATACAAAACATGGGTATCAAAGTCACTTTTTTT
GTTGACCAGCAGATATTGTTTCATTTACAAACATTACATATTTTAGAATATGTACACTA
CCGTTCAAGTTTGGGGTCACTTAGAAATGTCCTTGTTTTTGAAAGAAAAGCACATTTAA
AGTCCATTAAAATAACATCAAATTGATCAGAAATACAGTGTAGACATTGTTAATGTTGT
ACTACCGTTGAACGGTAGTGTTCACTGACTTCACCTCTTAACACATTACAAATATGTTT
GTTTCCATCAAATGCAGTTTGAAGCTTATGCACTAAGCAGGTGCTGTTAGTGACATTTA
GTGTCCATTAGGCACTTAGTGTTTCACACCTGTGGAGAAGGGATTCTAATCCTGATGAC
ACAGACTGTTCCATGTCTAACGACCCTATAAAAGGGACTCATGATTATTCCC**ACCACAG**
TGTAGGAAGGAATACACAGAACGGAGAAAGTATGTGATTCATATAAATATATTTGAAAT
TGTTAACTAATGTGCATTTGTGGGTAGTTCATATATACTGTACAATGTGCATATTCAAT
AGGTAATCATTGCAAGATGATCGCAAGCTTATGTTTGATGTGAAAATACATTTTTGACT
ATCACATTTAGCTGGTATTTGACTAGCTTTCTTTCCAG**CTCCCATG**GATCTTAGCAACA
GAACGGTCGTGCAGGTGGTGGTGTTGGCGTTGGTAGCTCAGGTCACGCTCTCT**CAGCAC**
TGGTCGTATGGCTGGCTACCTGGAGGGAAGAGAAGTGTTGGGGAGCTGGAGGCCACCAT
CAAG**GC**AAGTACTATTTACCTCTACCTGTAACTACTGTAAGCAGCTATGTCTACATGTA
CATACTGTACATGTAATTGTCCTTTAAGGAAATCTACTATGTCTTGAAAAGCTGTGAAC
ATTTAAATATAGCATTTATAGGGATAGTTTGTGTGGAATATTTGTTCAAGAGGCCTTTT
CAAGAGTACATAGGCAATGTATATTAATTAAACTCTGTAAGGGTGTTGCATAAAGGGTA
TATGGGTAATAGCATTACAAATTCCTCCTACATATTGCTATTC**TAG**ATGATGGACACAG
GAGATGTAGTGGCTCTTCCTGAGGAGACAAGTGCCCATGTCTCAGAGAGACTGAGACCA
TATGATGTA**GT**AAGTAGTCATATTAATTTATTAGGTTAGATATTGTATTCATTGTATTC
CTAATGGTTTCATCTGAAGATGCTAAATTGTCTCCTTGTAAAATATGTTGTAAACAATA
TACCAAAGTATCTGAAATAAATGTTTAGATGAGAGTATTGGCTGACAAATGGAAATGTT
CCCTGAATTTAGCGAAGTGATACCTATAAATGTAGGGAGACAATGTTGCTCATGTCTGA
CACATCAGTATAATATAGATATACAACATAAAGTAGACTTCTGAAGTTAACGCTTTGAT
TTCCCT**AG**ATATTGAAGAAATGGATGCCCCATAAATAAAGAACTGTGAGACCATTATTC
ACAAAAGAAGCAAGAAGACAACATCAAGCAGACATTCAGCATCACTATCAACATCAATG
ATGGAGCATTCAACTACAGTTCTGTCCATTTCCAACAAACATCACATTTAGATTGATGT
TTATTTACTTGAAGTATAACACTTTAACCTTCTGTAAAATTGT**AATAAA**GAGTGATTCA
AAAAGAGAAGTATCTTTGTATGTCTATTGAACGTCCTTACAATACAGGAGCTTAAGTGT
CCTATCCAAATTTCAATTAATCAATTCGTGATCCATCCAAATTTTTAGGTGGAGTTGAC
AGACCATGAATTGATTATTACCATACATTTGCTTT

mRNA:

ACCACAGTGTAGGAAGGAATACACAGAACGGAGAAACTCCCATGGATCTTAGCAACAGA
ACGGTCGTGCAGGTGGTGGTGTTGGCGTTGGTAGCTCAGGTCACGCTCTCT**CAGCACTG**
GTCGTATGGCTGGCTACCTGGAGGGAAGAGAAGTGTTGGGGAGCTGGAGGCCACCATCA
AGATGATGGACACAGGAGATGTAGTGGCTCTTCCTGAGGAGACAAGTGCCCATGTCTCA
GAGAGACTGAGACCATATGATGTAATATTGAAGAAATGGATGCCCCATAAATAAAGAAC
TGTGAGACCATTATTCACAAAAGAAGCAAGAAGACAACATCAAGCAGACATTCAGCATC
ACTATCAACATCAATGATGGAGCATTCAACTACAGTTCTGTCCATTTCCAACAAACATC
ACATTTAGATTGATGTTTATTTACTTGAAGTATAACACTTTAACCTTCTGTAAAATTGT
AATAAAGAGTGATTCAAAAAGAG

protein:

MDLSNRTVVQVVVLALVAQVTLS**QHWSYGWLPG**GKRSVGELEATIKMMDTGDVVALPEE
TSAHVSERLRPYDVILKKWMPHK

Organism name: Oncorhynchus mykiss (rainbow trout)

>gi|1207596458|ref|NC_035077.1|:70464991-70467435
Oncorhynchus mykiss isolate Swanson chromosome 1, Omyk_1.0,
whole genome shotgun sequence

AATTTCTGAATATTTGTAAAAGATACAAAACATGGGTATCAAAGTCACTTTTTTGTTGA
CCAGCAGATATTGTTTCATTTACAAACATGAAATATTTTAGAATATGTACACTACCGTT
CAAGTTTGAGGTCACTTAGAAATGTCCTTGTTTTTGAAAGAAAAGCAACATTTTTGTCC
ATTAAAATAACATAAAATTGGATCAGAAATACAGTGTAGACATTGTTAATGTTGTAAAT
GACTATTGTAGCTGGAAACGGCAGATTTTTAATAGAATATCTACATAGGCGTACAGAGG
CCCATTATCAGCAACCATCATTCCTGTGTTCCAATGGTATGTTGTGTTAGCTAATCCAA
GTTTATAATTTTAAAAGGCTAGTTGATCATTAGAAAACCTTTTGCAAATATGTTAGCAC
AGCTGAAAACTGTTGTTCTGATTAAAGAAGCATTAAAACTGGCCTTCATTAGACTAGTT
GAGTATCTGGATCATCAGTATTTGTGGGTTCGATTACAGGCTCAAAATTGCCAGAAACA
AAGACTTTTCATCTGAAACTCATCAGTCTATTCTTGTTCTGAGAGATTAAGGCTATTCC
ATGCAAGAAATTGCCAAGAAACTGAAGATCCTGTACAACGCTGTGTTCTACTCTCTTCA
CAGAACAGCGCAAACTGGCTCTAACCAGAATAGAGTGGGAGGCCCCGGAGCACAACTGA
GCAAGAGGACAAGTACATTAGAGTGTCTAGTGTCTCACAGTGTCTCACAAGTCCTCAAC
TGGCAGCTTCATTAAATAGTACCCACAAAACACCTATCTCAACGTCAACAGTGAAGAGG
CGACTACGGGATGCTGGCCTTCTAGGCAGAGTTCCTCTGTCCAGTGTCTGTGTTCTTTT
GCCCATCTTAATCTTTTATTTTTATTGGCCAATTCTGAGATATGGCTTTTTCTTTGCAA
CTCTGCCTAGATGGCCAGCATCCCGGAGTCGCCTCTTCACTGCTGACGTTGAGACTGGT
GTTTCTGGCCATTTTGAGCCTGTAATCGATCCCACAAATGCTGATGCTCCAGATATTCA
ACTAGTCTAAAGAAGGCAAGTTGTATTGCTTCTTTCACTAGCACAACAGTTTTCAGCTG
TGCTAACATATTTGCAAAAGGGTTTTCTAATGATCAATTAGCCTTTTAAAATTATAAAC
TTGGATTAGCTAACACAATGTGCCATTGGAACACAGGTGTGACAGTTGCTGATAATGGG
CCTTTTAATGCCTATGTAGATATTCCATTTAAAAAATTGCCGTTTCCAGCTACAATAGT
CATGTACACTATCATTAACAATGTCTACACTGTATTTCTGATCAATTTGATGTTATTTT

AATGGACAAAAATGTTGCTTTTCTTTCAAAAACAAGGACAATTCTAAGTGACCTCAAAC
TGTTGAACGGTAGTGTACACTGACTTCACCTCTTAACACATTATAAATATGTTTGTTTC
CATCAAATGCAGTTTGAAGCTTATGCACTAAGCAGGTGCCATTAGTGACGTTTAGTGTC
CATTAGGCACTTAGTGTGTCACACCTGTGGAGAAGGGATTCTAATCCTGATGACACAGA
CTGTTCCATGTCTAACGACCCCTATAAAAGGGACTCATGATATTCCC**ACCACAGTGTAG**
GAAGGAATACACAGAACGGAGAAAGTATGTGATTCATATAAGTATATTTCAAATTGTTA
ACTAATGTGCATTTGTGGGTAGTTCATATATACTGTACAATGTGCATATTCAATAGGTA
ATCATTGCAAGATGATCGCAAACTTCTGCTTGATGTAAAAATACATTATTTTTGACGAT
CACATTTAGCTGATATTTGACTAGCTTTCTTTCCAG**CTCCCATG**GATCTTAGCAACAGA
ACGGTCGTGCAGGTGGTGGTGTTGGCGTTGGTAGCGCAGGTCACGCTCTCT**CAGCACTG**
GTCGTATGGCTGGCTACCTGGAGGGAAGAGAAGTGTTGGGGAGCTGGAGGCCACCATCA
AG**GC**AAGTACTATTTACCTCTACCTGTAACTACTGTTACAGCTATGTCTACATGTGCAT
ACTGTACATGTAATTGTCCTTTAAGGAAATCTACTGTGTCTTGAAAACCTGTGAACATT
TAAATGTGGCATTTATCGGGATAGTTTGTGTGGAATATTAGTTCAAGAGGCCTTTTCAA
GAGTATATTGGCAATGTATATTAATTAAACTCTGTAAGGGTGTTGCATAAATGGTATAT
GGGTAATAGCCTTACAAATTCCTCCTACATATTGCTATC**CAG**ATGATGGACACAGGAGG
TGTAGTGGTTCTTCCTGAGGAGACAAGTGCACATGTCTCAGAGAGACTGAGACCATATG
ATGTA**GT**AAGTAGTCATATTAATTTATTAGGTTAGATATTGTATTCACTGTATTCCTAA
TGGCCAATTATTTATGTTTCATCTGAAGATGCTACATTTTCTCCTGGTAAAATATGTTG
TAAACAATATACCAAAGTATCTGAAATAAATGTTTAGATAAGAGTAATGGCTGACAAAT
GGAAATCTTCCCTGAATTTAGCGAAGTGATACCTATAAATGTAGGGAGACAATGTTGCT
CATGTCTGACACATCAGTATAATATACAACATAAAGTAGACTTCTGAAGTTAACGCTTT
GATTTCCCT**AG**ATATTGAAGAAATGGATGCCCCATAAATAAACAACTGAGACCATTATT
CACGAAAGAAGCGAGAAGACAACATCAAGCAGACATACAGCATCACTATCAACATCAAT
GATGGAGCATTCAACTACAGTTCTGTCCATTTCCAACAAACATCACATTTAGATTGATG
TCATTTACTGAAAGTATAACACTTTAACCTTCTGTAAAATTGT**AATAAA**GAAGTGATTC
AAAAGGAGAAGTATCTTTGTATGTCT

mRNA:

ACCACAGTGTAGGAAGGAATACACAGAACGGAGAAACTCCCATGGATCTTAGCAACAGA
ACGGTCGTGCAGGTGGTGGTGTTGGCGTTGGTAGCGCAGGTCACGCTCTCT**CAGCACTG**
GTCGTATGGCTGGCTACCTGGAGGGAAGAGAAGTGTTGGGGAGCTGGAGGCCACCATCA
AGATGATGGACACAGGAGGTGTAGTGGTTCTTCCTGAGGAGACAAGTGCACATGTCTCA
GAGAGACTGAGACCATATGATGTAATATTGAAGAAATGGATGCCCCATAAATAAACAAC
TGAGACCATTATTCACGAAAGAAGCGAGAAGACAACATCAAGCAGACATACAGCATCAC
TATCAACATCAATGATGGAGCATTCAACTACAGTTCTGTCCATTTCCAACAAACATCAC
ATTTAGATTGATGTCATTTACTGAAAGTATAACACTTTAACCTTCTGTAAAATTGT**AAT**
AAAGAAGTGATTCAAAAGGAG

protein:

MDLSNRTVVQVVVLALVAQVTLS**QHWSYGWLPG**GKRSVGELEATIKMMDTGGVVVLPEE
TSAHVSERLRPYDVILKKWMPHK

Organism name: Oncorhynchus nerka (sockeye salmon)

>gi|1681300729|ref|NC_042544.1|:9181526-9184011
Oncorhynchus nerka isolate On170113-E2 linkage group LG10,
Oner_1.0, whole genome shotgun sequence

ACCCTATTAAGACACTTTATGTTGGTGTTTCCTTTATTTTGGCAGTTACCTGTATATGT
TTGCTTATCTGTCTGTTTGTTAAAAAGAGCCCAAATGAAAATGGATTATTATCTCTATT
TAGTCACGGACATTTCAACTAGCTTTGTCTCCTCAGCATTTGTATGTCAAATGTTTTGA
ATTGAAAACGTAAGACCAAAACCGGCGTGAAGGGTTTAATTGAATAGGTTCTCGGACTC
AAGACAATACTGAAAAGTATGTTATTATGTACATACTGTGTGTATTAATTAAGTTATGC
ATATAATCTTTTTCTATTTATATAATGTAGAATTCTATGTACATTTTGAGTTTTACTGA
TAGTCTATGTTGAAAAAATGACCATGAGATTGCCTTTCGTTGCCTGTGGTCTGTACTGT
AGACATGGCACATACTGTAGAACTCCTGCTGTTGTAAAAAATACACAACCTGTCATGAG
AGTTTGTGACAAGTGACTTAACAAATGAACATTCAGTTTTTGGTGTGTATGTTGTCTGT
TTCATCATAACAGTCTATTGAGTATCCAATGGAAAACAATCCATTTGGACAACTTTGAA
TTGCCATCTGTTTCAAACAGCAATTTTGATCTGACCTCACTAACAAATGGCTGTTCATA
TGTACACGGTTGTAATAACTTTTGTACAACTTTTTTTTTAAACTATGTTGGACCTTCCA
TTTCAGCATTTGCATGTTTGAATGAAACAGTGCCATACAAAGATTAGAAAAATGTTCTG
ATCAAGTTCAAATTGTCCCCAAGTGACTTCAACTTCAATATACATAGCTAAGGCAGCGG
ATTGTTTAATGTTTCTCAAGACTAACCTAAAAAGGAAGAAGATTTCAATGAAATGAGTT
GATTTTCATGATATCCTTATAGTGTATAGAACATGATTGGGATTAAAGCAAATGATTAT
AATGCTCATATACCCCCCAAAATGTGTATTGAATGTATAGAGTTCTCAATTAGAAAAGT
GTCATTTAGAGCCAGGCAGCAATTTTCCGCTTGGTAAGTATTTATAGCCACAGTGCTCT
CATACCACTAAATAACTGGGTTATTGATAATAGTAATTGATTTATGGAAGTTTCAGGAA
GATATTTTATTGAATTTCATCGTGCTGTAGTTTACTGTGAAATCTATATTTTAGCAATG
ATCATAAATAAATAGAATTTAAATTCAAATGACCTGGTTACCCAGTTATTAAGTGTTAA
GGCACTCCACAGGGCTATCCTTCATGCCAAAGGTACATTTATGGATTCATAGCCTGTTA
GGTAAAGATACAAGTGTAAGGATTATGTCTTCTTACAGAACATTTAGAATCAGTCTTGT
GTACAGAGTATGAAATTGAGCCATAATGTAGCGCTCAAGCATCTTGTTCCTGTATATCT
CTTGTCATAAGTCTGTATGAAGTTAAAATTTCATTTGTAAAATCTGTCCGCAATAAATG
CATTGAAAATAAACAAATTTGCCATTAATTTCTGAATATTTGTAAAAGATACAAAACAT
GGGTATCAAAGTCACTTTTTTTGTTGACCAGCAGATATTGTTTCATTTACAAACATGACA
TATTTTAGAATATGTACACTACTTGCCGTTTCCAGCTACAATAGTAATTTACAACATTA
ACAATGTCTACACTGTATTTCTGATCCAATTTGATGTTATTTTAATGGACAAAAATGTT
GCTTTTCTTTCAAAAACAAGGACATTTCTAAGTGACCTCAAACTGTTGAACGGTAGTGC
TCACTGACTTCACCTCTTAACACATTATAAATATGTTTGTTTCCATCAAATGCAGTTTG
AAGCTTATGCACTAAGCAGGTGCCATTAGTGACATTTAGTGTCCATTAGGCACTTAGTG
TGTCACACCTGTGGAGAAGGGATTCTAATCCTGATGACACAGACTGGTCCATGTCTAAC
GACCCCTATAAAAGGGACTCATGATATTCCC**ACCACAGTGTAGGAAGGAATACACAGAA**
CGGAGAAAGTATGTGATTCATATAAGTATATTTAAAATTGTTAACTAATGTGCATTTGT
GGGTAGTTCATATATACTGTACAATGTGCATATTCAATAGGTAATCATTGCAAGATGAT
CGCAAACTTCTGCTTGATGTAAAAATACATTATTTTTGACGATCACATTTAGCTGGTAT
TTGACTAGCTTTCTTTCCAG**CTCCCATG**GATCTTAGCAACAGAACGGTCGTGCAGGTTG
TGGTGTTGGCGTTGGTAGCGCAGGTCACGCTCTCT**CAGCACTGGTCGTATGGCTGGCTA**
CCTGGAGGGAAGAGAAGTGTTGGGGAGCTGGAGGCCACCATCAAG**GC**AAGTACTATTTA
CCTCTACCTGTAACTACTGTTACAGCTATGTCTACATGTGCATACTGTACATGTAATTG
TCCTTTAAGGAAATTACTGTGTCTTGAAAACCTGTGAACATTTAAATGTGGCATTTATA
GGGATAGTTTGTGTGTAATATTAGTTCAAGAGGCCTTGTATATTAATTCAACTCTGTAA
GGGTGTTGCATAAAGGGTATATGGGTAATAGCATTACAAATTCCTCCTACATATTGCTA
TC**CAG**ATGATGGACACAGGAGGTGTAGTGGCTCTTCCTGAGGAGACAAGTGCACATGTC

TCAGAGAGACTGAGACCATATGATGTA**GT**AAGTAGTCATATTAATTTATTAGGTTAGAT
AATGTATTCATTGTATTCCTAATGGCCAATTATTTATGTTTCATCTGAAGATGCTACAT
TTTCTCCTGGTAAAATATGTTGCAAACAATATTCCAAAGTATCTAAAATAAATGTTTAG
ATAAGAGTAATGGCTGACAAATGGAAATCTTCCCTGAATTTAGCGAAGTGATACCTATA
AATGTAGGGAGACAATGTTGCTCATGTCTGACACATCAGTATAATATAGATATACAACA
TAAAGTAGACTTCTGAAGTTAACGCTTTGATTTCCCT**AG**ATATTGAAGAAATGGATGCC
CCATAAATAAACAACTGAGACCATTATTCACAAAAGAAGCGAGAAGACAACATCAAGCA
GACATACAGCATCACTATCAACATCAATGATGGAGCATTCAACTACAGTTCTGTCCATA
TCCAACAAACATCACATTTAGATTGATGTTATTTACTTGAAGTATAACACTTTAACCTT
CTGTAAAATTGT**AATAAA**GAAGTGATTCAAAAGGAGAAGTGTCTTTGTATGTCTATTGA
ACGTCCTTACAATACAGGAGCTTAAGTGTCCTATCCCAAATTTTAATTGATCAATTCGT
GATCCATCCAAATCTTTAGGTGGAGTTGACAGACCATTAATTGATTATTAACATACATT
TGCTTGCTATGACCGACAAGCATGATCAACTTGTGATACAACAATCGTAGCATGACAAC
AGCTGGTAATTAAGTCAATTTATAAAACATCTATGGTGTCATTAGATTGAAACAGGGGT
TTATGTCCCTTTTGTAATTAGATTATAGATTATTGCTTGTCGGTCATGCATAATTTCAA
AGGGAAAATCCAC

mRNA:

ACCACAGTGTAGGAAGGAATACACAGAACGGAGAAACTCCCATGGATCTTAGCAACAGA
ACGGTCGTGCAGGTTGTGGTGTTGGCGTTGGTAGCGCAGGTCACGCTCTCT**CAGCACTG**
GTCGTATGGCTGGCTACCTGGAGGGAAGAGAAGTGTTGGGGAGCTGGAGGCCACCATCA
AGATGATGGACACAGGAGGTGTAGTGGCTCTTCCTGAGGAGACAAGTGCACATGTCTCA
GAGAGACTGAGACCATATGATGTAATATTGAAGAAATGGATGCCCCATAAATAAACAAC
TGAGACCATTATTCACAAAAGAAGCGAGAAGACAACATCAAGCAGACATACAGCATCAC
TATCAACATCAATGATGGAGCATTCAACTACAGTTCTGTCCATATCCAACAAACATCAC
ATTTAGATTGATGTTATTTACTTGAAGTATAACACTTTAACCTTCTGTAAAATTGT**AAT**
AAAGAAGTGATTCAAAAGGAG

protein:

MDLSNRTVVQVVVLALVAQVTLS**QHWSYGWLPG**GKRSVGELEATIKMMDTGGVVALPEE
TSAHVSERLRPYDVILKKWMPHK

Online Resource 4

The late-evolving salmon and trout join the GnRH1 club

Histochemistry and Cell Biology

Kristian R. von Schalburg, Brent E. Gowen, Kris A. Christensen, Eric H. Ignatz, Jennifer R. Hall, Matthew L. Rise

Corresponding author at: Department of Biology, Electron Microscopy Laboratory, University of Victoria, Victoria, British Columbia, Canada V8W 3N5

E-mail address: krvs@uvic.ca (K.R. von Schalburg)
