# Supplementary material for: The late-evolving salmon and trout join the GnRH1 club
Source: Histochem Cell Biol. 2023 Aug 11;160(6):517–39. doi: 10.1007/s00418-023-02227-z (PMC10700215; doi:10.1007/s00418-023-02227-z)
Supplement: Supplementary file 5 — Supplementary file5 (PDF 90 KB) [file 418_2023_2227_MOESM5_ESM.pdf]

***Coregonus* sp. 'balchen' GnRH1 viable GnRH1 gene**

TTCAATAAATTACTGTTCTCAATAAACAAAAGCCACTGTGTAATTACTTGTGCAAATGA
AATACTGGTATCCTTTCACTAACTTCCCAGAAGAACACCAAGACCCTGTGCCTAATTCA
TTTCATTGTTGCTGCTGTAAAAGTGCACAATCGTTCCCAAAGTATTGTTAAGCTTTTAA
AAGGCTCTTTGCGGTGCCTGAAGTGGAGGTGCTTAGTGCTGCTCTGTAATATATTTTTG
AGTGGAGCCTTTTTCTGTAATCCACTTTGTAACCTCCTGCAACTAGGTGATGGGGCTGG
GGGGTGGGGCACATGGGAACCAAGCTGCCTATAAAACCTCAAAGACACATGAGTTGCTC
AGCTAGAGTAATAAGGCTGACTTTGCAGAATGGAAGAGAAAAAGGTCCTGTTGTTGCTG

exon 1 **Q H W S Y G**
CTGCTTTTGGTGGTGGCTCTAGTGTCACAGGGTTGCTGTCAACATTGGTCCTATGGCAT

M N P G G K R **exon 1 end!**
GAACCCAGGGGGGAAAAGAGCGACTGGCAGCCTGTCTGACACCCAGGACAAT**GT**AAGTA
CTTTACCTATTCATAATGTGGAAGCAGAACTAGCTAGGTCTATTTACTAAAGGTTGAGA
TGGTAAAGTATACAATGATGGAAGGGATGTTCCTATATTTGCATAGTATAATCTGGCTA
exon 2
AATGACAATTCTA**CAG**ATGGCTGAAGACCTTCTGAAGATAGACCCTTGCAGTTTGTTTG
GCTGTGCTGATGTCTCACCTCATGCCAAAATGTACAGGCTGAGGGCATTACTT**GT**GAGT
GACATTTACAATTTTGCATATACATTTCCCTTCAAGAAGCTACCCAGAAGTTGAATATA
AAGCGAAATTGTGTTGGTAGTTCAATATTGCTACTTTATTTAATGTATGTGTTTTTTTC
TAAATTTCTTCAATTTTTATCCAAATTGTAAATTATGTTTTTCCTTTACTTTTTTG**TAG**
exon 3
GCAAGCCTCGCTGACAGACAAAGTGGACTCAATAATATATAGCAAATGTATGCTAGCTA
ACTCAATGAAACATTGCAGTGCCATCATTGTGTTTTATGGTGGTCTTTGTTGGGGGTCC
CATTTTTGTTGGTTTGCATATTACATAGTATGGCTTTAAAACAGTGGAACACGATGTCA
A**AATAAA**AGTGCCAACACATGATTGCG

The gene above would encode a viable full-length GnRH1 preprohormone. We only show the encoded GnRH decapeptide and the amidation/proteolytic tripeptide. The putative 5'-untranslated region and GT-AG exon-intron splice sites are shown in bold. Below we show the *Coregonus* sp. *gnrh1* nonviable gene which is the only pseudogene of the salmonids we examined that seems to have duplicated copies of the sequence that once encoded the GnRH1 decapeptide.

***Coregonus* sp. 'balchen' GnRH1 pseudogene examined in two coding frames**

a

CACAAAAATGTAATTCAATAAATTACTGTTATGAATTAAAAAATAAATTACTTGTGCAA
ATGAAATACTGGTATGTTTTCGCTAACTTTCCAACTAATAACTCTTAAGACCCTGTGCC
TAATTCATATAATAGTTGCTGCTGTAAAAGTGCACAATCATACCCAAAGTATTTTAGGC
TTTTAAAAGGCTCTTTGGGGTGCCTGAAGTGGAGGTGCTTAGTGCTGCTCTGCAAAATA
TTTTTGAGTGGATTATTTTTTCTGTAATCCCACTTTTTTGTGACCTGCAGCGAGGTGAT
GGGACTAAGGGGCCACATGGAAACCAAGCTTCCAATAAAACCTCAGAGACAAATGAACT

GCTCAGATAGAGTAAGGCTGACTATTGGTAGA**ATG**GAAGAGAACAATGTCCTGTTGTTG
CTGCTGCTCTTGGTGGTGGTACTAGTGTCACAGGGTTGCTGTCAACATTGGTACCATGA

Q H W S Y D M R P G E K R
CATGAGCCCGTCAACATTGGTCCTATGACATGAGACCAGGGGAGAAGAGAGAGACTGGC
AGCCTGTCTGACACTGTGGGAAAT**GT**AAGTACACTGCTCAAAAAAATAAAGGGAACACT
TAAACAACACAATGTAACTCCAAGTCAATCACACTTCTGTGAAATCAAACTGTCCACTT
AGGAAGCAACACTGATTGACAATACATTTCACATGCTGTTGTGCAAAAGACACCCCCAA
TAAAGGACTGGTTTTGCATGTGGTGACCACAGACCACTTCTCAGTTCCTATGCTTCCTG
GCTGATGTTTTGGTCACTTTTGAATGCTGGCGGTGCTTTCACTCTAGTGGTAGCATGAG
ACGGAGTCTATAACCCACACAAGTGGCTCAGGTAGTGCAGCTCATCCAGGATGGCACAT

b

CACAAAAATGTAATTCAATAAATTACTGTTATGAATTAAAAAATAAATTACTTGTGCAA
ATGAAATACTGGTATGTTTTCGCTAACTTTCCAACTAATAACTCTTAAGACCCTGTGCC
TAATTCATATAATAGTTGCTGCTGTAAAAGTGCACAATCATACCCAAAGTATTTTAGGC
TTTTAAAAGGCTCTTTGGGGTGCCTGAAGTGGAGGTGCTTAGTGCTGCTCTGCAAAATA
TTTTTGAGTGGATTATTTTTTCTGTAATCCCACTTTTTTGTGACCTGCAGCGAGGTGAT
GGGACTAAGGGGCCACATGGAAACCAAGCTTCCAATAAAACCTCAGAGACAAATGAACT
M E E N N V L L L
GCTCAGATAGAGTAAGGCTGACTATTGGTAGA**ATG**GAAGAGAACAATGTCCTGTTGTTG
L L L L V V V L V S Q G C C **Q H W Y H**
CTGCTGCTCTTGGTGGTGGTACTAGTGTCACAGGGTTGCTGTCAACATTGGTACCATGA

D M S P S T L V L *
CATGAGCCCGTCAACATTGGTCCTATGACATGAGACCAGGGGAGAAGAGAGAGACTGGC
AGCCTGTCTGACACTGTGGGAAAT**GT**AAGTACACTGCTCAAAAAAATAAAGGGAACACT
TAAACAACACAATGTAACTCCAAGTCAATCACACTTCTGTGAAATCAAACTGTCCACTT
AGGAAGCAACACTGATTGACAATACATTTCACATGCTGTTGTGCAAAAGACACCCCCAA
TAAAGGACTGGTTTTGCATGTGGTGACCACAGACCACTTCTCAGTTCCTATGCTTCCTG
GCTGATGTTTTGGTCACTTTTGAATGCTGGCGGTGCTTTCACTCTAGTGGTAGCATGAG
ACGGAGTCTATAACCCACACAAGTGGCTCAGGTAGTGCAGCTCATCCAGGATGGCACAT

It is unlikely the GnRH1 pseudogene is transcribed since the proximal promoter has lost many of the features that are present in the viable *gnrh1* promoters examined here. For example, the two potential TATA boxes are missing in the GnRH1 pseudogene (underlined in viable gene). Nevertheless, the remnants of GnRH1-encoding sequences exist in the *Coregonus* sp. nonviable *gnrh1* ohnolog. Interestingly, sometime in the past, the GnRH1 decapeptide-encoding sequence was duplicated (sequences highlighted in red and yellow} and then diverged (**a** and **b**). The sequences retain 73% (red) and 86% (yellow) identity compared to the viable GnRH1-encoding sequence.
a The sequence encoding QHWSYDMRPG (sequence in yellow) cannot be translated due to several upstream stop codons (underlined). **b** The second, likely 'original' GnRH1 signal and hormone encoding sequences could be translated using the upstream start codon (in green), but would be terminated four amino acids following the decapeptide

QHWYHDMSPS (sequence in red). As well, the decapeptide would remain inactive and unspliced in the prohormone, which lacks viable amidation/proteolytic residues in positions 11 to 13. Thus, even if the pseudogenic *gnrh1* mRNA was somehow transcribed, correctly spliced, processed and translated, it could not produce a viable GnRH hormone.

Online Resource 5

The late-evolving salmon and trout join the GnRH1 club

Histochemistry and Cell Biology

Kristian R. von Schalburg, Brent E. Gowen, Kris A. Christensen, Eric H. Ignatz, Jennifer R. Hall, Matthew L. Rise

Corresponding author at: Department of Biology, Electron Microscopy Laboratory, University of Victoria, Victoria, British Columbia, Canada V8W 3N5

E-mail address: krvs@uvic.ca (K.R. von Schalburg)
