# Supplementary material for: The late-evolving salmon and trout join the GnRH1 club
Source: Histochem Cell Biol. 2023 Aug 11;160(6):517–39. doi: 10.1007/s00418-023-02227-z (PMC10700215; doi:10.1007/s00418-023-02227-z)
Supplement: Supplementary file 6 — Supplementary file6 (PDF 98 KB) [file 418_2023_2227_MOESM6_ESM.pdf]

Organism name: *Oncorhynchus kisutch* (coho salmon)

>gi|1154410508|ref|NC_034181.1|:42296502-42299810 Oncorhynchus kisutch isolate 150728-3 linkage group LG8, Okis_V1, whole genome shotgun sequence

CATACTGTAGCATGATAAAAATGGCAAAAGAATTTGCTGTTATTGCACCACCTAATTATGCTGTGGATTT
CATTTTTCTTATAATAATGTGGCTGATGAAACAGAATTTCAAGAATGAAATGTACTCTTCATTGATAATC
ACTGAAGTCAGAAGCTGCCTGTTGTTTTGTTTGGTGTTCTCTGTGAATCTACCTCGTTTTTTTTTTTTAC
CACTTTCTGATTCATATCTAGTTCCATGCATTTCATGGTGTTTTTTGTCTCTTGTTGTATGAAAACCATT
ATTCATCATATCCAAATATGATGCGCAAATAATCAAAGTGAATAAGCTGTTTTTTAATAGTGTTACTGTC
ATTTTCAAGCTTGTGGATCACCCAGTTGTATGAGGAAGATGGTTGTATAACAACACTCCCATCACAATGA
ATTGCATATAATTCAATCATGTTTTGAAGTTTGCACAAATCTGTCTTAAATTAAACATTTTGTTCACTAA
CTTCTGTTTTACTTACTCGCAGGACACCAAAAAGTTGAATGTGGTGCTTGTAGTGTTTTTTATTTTATTT
TTTTAAATCCAGTGCTTACAAAACTTGTGCGTTTTATAAGGATAGCAAAACATCACTGCTGGACTGTGAT
TGTTAAAATCTGTCAGTTTGTTTAAGAAAATGTTTGCATGTTTAAAGACTGTATTTTACCAGTCTAAGGC
TGCTGAATTTTTTTCTAGGTGTAGCTGAAACCTTTTTTGAGATGCAATATTCTCTCTCTATACTCTATTC
CATAGTAGGTACTCTTTTGTCGTGATAATGAAGGCAATCATAAAGAATATTGCACCTTGAGTGTGGAGAT
CGTCAGTTTCAGGACCTTGTAGAAAAGAACAATGGTTATTTATTTTGATATAACATATCGACGAAATATG
GAATGAAAGTTAGTTAAAGACTGGGGGTGTTTCTCTTCCTCCCCTGTCTATAATTATTGCACTCAAGCAC
AAAAATGTATTTCAATAAATTACTGTTATCAATAAACAAAAGCCACTGTGTAATTAAAAATGAAATACTG
TTTTCACTAACTTTCCAGAAGAACACCAAGACCATGTGCCTAATTAATTTTATCGTTGCTGCTGTAAAAG
TGCACAATCATTCCCAAAGTGTTAATCTTTTAAAAGGCTATTAGGGGTGCCTGAAGTGGAGGTGCTTCAA
GCTGCTCTGTAATATATTTTTGAGTGGAGCCTTTTTCTGTAATCCACTTTGTCCTCCTGCAGCAGTGATG
GAACTGGTGGGTATGGCATATGGGAACTAAGCTGCCTATAAAACCTCAGACACCTGAGTTGCTT**ACCTAA**
AGTAATAAGGCTTACTTTGCAGAATGGAAGAGAAAAAGGTCTTGTTGCTGCTGCTTTTGGTAGCGCCTCT
AGTGTCACAGGGTTGCTGT**CAACATTGGTCCTATGGCTTGAACCCAGGG**GGGAAAAGAGTTACTGACAGC
CTGTCTGACACCCTGGACAATGTAA**GT**ACTTTACCTATTCATAATATGGAAGCAAAAGTAGCTAGGTCTT
TTCCTAAAGGTTGTGATGGTAAAGTATCCAATGATGGCAGGGATGTTCCTATAGTTGCATTGTAAAATC**T**
AGTTAAAATGACAATTCTACAGCTGGCTGAAGACCTTCCGAAGATAGACACATCTTGCAGTTTGTTTGGC
TGTGCTGATGTCTCACCTCATCCCGAAATGTACTGGCTGAGGGCATTACTTATGA**GT**GACATTTACAATT
TTAAAAATACATTTCCCTTCAAGAAGCTACCCAGAAGTTGCTCTTGCTGCGGGCGATTCCCTGATCCACC
TCTACGCAGACGACACCATTCTATATACTTCCGTCCCGTCCTTGGACACTGTGCTATCTAACCTCCAAAC
GAGCTTCAATGCCATACAACACTCCTTCCGTGGCCTCCAACTGCTCTTAAACGCTAGTAAAACCAAATGC
ATGCTTTTCAACCGTTCGCTGCCTGCACCCGCACGCCTGACTAGAATCACCACCCTGGATGGTTCTGACC
TTGAATATGTGGACATCTCTAAGTACCTAGGTGTCTGGCTAGACTGTAAACTCTCCTTCCAAACATCTCC
AATCGAAAATCAAATCTAGAGTCGGCTTTCTATTCCGCAACAAAGCCTCCTTCACTCACGCCGCCAAACT
TACCCTAGTAAAACTGACTATCCTACTGATCCTCGACTTCGGCGATGTCATCTACAAAATTGCTTCCAAC
ACTCTACTCAGCACACTGGATGCAGTTTATCACAGTGCCATCCGTTTTGTCACTAAAGCACCTTATACCA
CCCACCACTGCGACCTGTATGCTCTAGTCGGCTGGCCCTCGCTACATATTCGTCGCCAGACCCACTGGCT
CCAGGTCATCTACAAGTCCATGCTAGGTAAAGCTCCGCCTTATCTCAGTTCACTGGTCATGATGGAAACA
CCCACCCGTAGCACGCGCTCCAGCAGGTGTATCTCACTGATCATCCCTAAAGCCAACACCTCATTTGGCC
GCCTTTCGTTCCAGTTCTCTGCTGCCTGTGACTGGAATGAATTGCAAAAATCGCTGAAGTTGGAGAATTT
TATCTCCCTCACCAACTTCAAACATCTGCTATCTGAGCAGCTAACCGATCGCTGCAGCTGTACATAGTCT
ATCGGTAAATAGCCCACCCAATTTTACCTACCTCATCCCCATACTGTTTATATTTATTTACTTTTCTGCT
CTTTTGCACACCAGTATCTCTACCTGTACATGACCATCTGCTCATTTATCACTCCAGTGTTAATCTGCAA
AATTGTAATTATTCGCCTACCTCCTCATGCCTTTTGCACACAATGTATATAGACTCCCTTTTTTTCTCTA
CTGTGTTATTGACTTGTTAATTGTTTACTCCATGTGTAACTCTGTGTTGTCTGTTCACACTGCTATGCTT

TATCTTGGCCAGGTCGCAGTTGCAAATGAGAACTTGTTCTCAACTAGCCTACCTGGTTAAATAAAGGTGA
AATAAAAAATAAATAAAAAATAGTTTAATATAAAGGGAGGTTGTTTTGGTAGTTAAATATTGCAACTTTT
TATGTGTTTTTTTTCTCAATGTTTTAAACAATTTGTTTTAAACAATTTGTATCCAAATTGTAAGTAATGT
TTTTCATTTACTTTTTTC**TAG**GTAAGCCTCGC**TGA**CAGACAAAGTGGACTCAATAATATATAGCAACTGT
ATGCTAGCTAACTCT**ATTAAA**CATTGCCGTGCCATCATTGTGTTTTGTGGTGTCTGTTGGGGGACAATTT
TTGCTAGATCGCATATTACAGGGTATGGCTTTAAAACAGTGTAACACGATGTCAAAATACAATGACGGGT
AGGCCATCAT**AAATAA**GAATTTGTTCTTAATTAACTGACTTGCCTAGTTAACTAAATTCCAACAAATGAT
TGCCAGTATGAGTTACGTTGTCTACTCTTGTAGCAAACCTTTATTTTGAAAATGTTGACAGCATGGCTTT
AATGTCGAGTTCC

We show that the GT-AG borders for the coho *gnrh1* have shifted at the end of exon 1 and both ends of exon 2. The additional basepairs included in exon 1 and exon 2 due to these changes are underlined. The stop codon (**TGA**) follows the acceptor **TAG** site of exon 3. We join the three exons below to provide the likely complete mRNA and the protein it encodes.

mRNA:

ACCTAAAGTAATAAGGCTTACTTTGCAGAATGGAAGAGAAAAAGGTCTTGTTGCTGCTGCTTTTGGTAGC
GCCTCTAGTGTCACAGGGTTGCTGT**CAACATTGGTCCTATGGCTTGAACCCAGGG**GGGAAAAGAGTTACT
GACAGCCTGTCTGACACCCTGGACAATGTAATTAAAATGACAATTCTACAGCTGGCTGAAGACCTTCCGA
AGATAGACACATCTTGCAGTTTGTTTGGCTGTGCTGATGTCTCACCTCATCCCGAAATGTACTGGCTGAG
GGCATTACTTATGAGTAAGCCTCGC**TGA**CAGACAAAGTGGACTCAATAATATATAGCAACTGTATGCTAG
CTAACTCT**ATTAAA**CATTGCCGTGCCATCAT

protein:

MEEKKVLLLLLLVAPLVSQGCC**QHWSYGLNPG**GKRVTDSLSDTLDNVIKMTILQLAEDLPKIDTSCSLFG
CADVSPHPEMYWLRALLMSKPR

Organism name: *Salvelinus* spp.

>gi|1340979591|ref|NC_036872.1|:16608763-16610800 Salvelinus spp. isolate IW2-2015 linkage group LG33, ASM291031v2, whole genome shotgun sequence

ATTTGCACTAGAGCACATTCAGGGATATTTGTTTCTGTTATGTAGTGGACGCTTTTACCTGCCTTTCCTT
ATTTATCCCCAAAACCAAAATATTTCATGTATGTTTACTATTATGTCTGGATTCAATCCTACCTTATTAC
TATTTTTTTTAATTCTAGATGTATACACACTTGATTATTTAACGCAACATCTCTGAAAGTCAAAGATTAT
TAAACTAGTAAGTGCATGAAGGTTTAAAGTAACAATATAAGGGCATGGTGGGACTGTACATTCTTATCAA
TGTACTTAGTTTTATGCCAGATATGCTTTATGATTAGGATAATGTTTTTTTTTTCTAAGCCAAAGCCATA
CTGTAGCATGATAAAAATGGCAAAAGAGTTTGCTGTTATTGCACCACCTAATTATGCTGTGGATTTCATT
TTTCTTATAATACTGTGGCTGTTGAAACAGAATTTCAAGAATGCAATGTACTCTTCCTTGATAATCACTG
AAGTCAAAAGCTGCCTCTTGTGGTGTTTGGTGTTCTCTGTGAATCTACCTCCTTATTTTTTACCGCTTTC

TGATTTATATCTAGTTGCATGCATTTCATGGTGGTTTTTGCCTCTTGTTGTATGAAAACCATTATTCATC
ATATCCAAATATGATAATCAAAGTGAATAAGATATTTTTTAATAGTGTTACTGTCATGTTCAAGCTTGTG
GATCACCTACTTGTATGAGGAAGATGGTTGTATAACAAAACTCACACCACAATGAATTGCATATAATTCA
ATCATGTTTTGAAGTTATACTTTGCACAAATCTGTCTTAAATTAAACATTTTGTTCACTAACTTCTGTTT
TATTTACTCGCAGGACACCAAAAAGTTGAATGTGGTGCTTGTAGTGTTTTTTTTTTTTTTTATCCAGTGC
TTACAAAACTTGTGCGTTTTATAAGGATAGCAAAACATTAGCACTGCTGGACTGTGATTTTAACAATCTG
TCAGTTTGTTTTTGCATGTTTAAAGACTGTATTTTACCAGTCTACGACTGCAGAATTGTTTTCTAGGTGT
AGCTGAAACCTTTTTGAGATGCAATATTCTCTCTCTATACTCTATTCCACAGTAGGTACTCTTTTGTCAT
GATAATGAAGGCAATCATAAAGAATATAGCACCTTGAGTGTGGAGATCGTCAGTATCAAGACCTTGTAGA
AAAGAACAATGGTTATTTATTTTGATATAACATATTGAAGAAATATGGAATGAAAGTTAGTTCAAGACTG
GGGTGTTTCTCTTCCTCCCCTGTCTATAATTATTGCACTCAAGCACAAAAATGTATTTCAATAAACAAAA
GCCACTGTGTAATTACAAATGAAATACTGTTTTCTTTTCACTAACTTCCCAGAAGAACACAAAGACCATG
TGCCTAATTCATTTTATCGTTGCTGCTGTAAAAGTGCACAATCATTCCCAAAGTGTTAAGCTTTTAAAAG
GCTATTTGTGGTGCCTGAAGTGGAGGTGCTTAAAGCTGCTCTGTAATATATTTTTGAGTGGAGCCTTTTT
CTGTAATCCACTTCGTGTCCTCCTGCAGCAGTGATGGAACTGGTGGGTGTGGCACATGGGAACTAAGCTG
CCTATAAAACCTCAGACACCTGAGTTGCTT**ACCGAAAGTAATAAGGCTGACTTTGCAGAATG**GAAGAGAA
AAAGGTCTTGTTGCTGCTGCTTTTGGTAGCGGCTCTAGTGTCACAGGGTTGCTGT**CAACATTGGTCCTAT**
GTCTTGAACCCAGGGGGGAAAAGAGTGACTGACAGCCTGTCTGACACCCTGGACAAT**GT**AAGTACTTTAC
CTATTCATAATATGGAAGCAGAAGTAGCTAGGTCTTTTACTAAAGGTTGTGATGGTAAAGTATCCAATGA
TGGAAGGGATGTTCCTATATTTGCATTGTAAAATCTAGTTAAAATGACAATTCTA**CAG**ATGGCTGAAGAC
CTTCCGAAGATAGACACATCTTGCAGTTTGTTTGGCTGTGCTGATGTCTCACCTCATCCTCTTACTT**GT**G
AGTGACATTTACAATTTTGAATATACATTTCCCTTCAAGAAGCTACCCAGAAGTTGAATATAAAGGGACA
TTGTGTTGGTAGTTAAATATTGCAACTTTTTTAATGTATGTTTTTTTTCTCTCAATGTTTTAAACAATTT
GTATCCAAATTGTAAGTCATGTTTTTAATTTGCTTTTTTC**TAG**GCAAACCTCGC**TGA**CAGACAAAGTGGA
CTCAATAATATATAGCAAATGTATGCTAGCTAACTCT**ATTAAA**CATTGCGGTGCCATCATTGTGTTTTGT
GGTGTCTTTGTTGGGTGACAATTTTTGCTAGATCGCATATTACATTGTATGGCTTTAAAGCAGTGTAACA
CGATGTCAAAATACAATGACGGGTAGGCAATCATTGT**AAATAA**GAATTTGTTCTTAACTGACTTGCCTAG
TTAAAACAAAACAAAAAACTAAATGCCAACAAATGATTGCCAGTATGAGTTACGTTGTTGACTCTTGTAG
CAAACCTTTATTTTGAAAATGTTAACAGCATGGCTTTAATGTCGAGTTCCTGTGTACACATGTTCATCAC
GGGTTA

At least 20 basepairs are missing in the region underlined in the *Salvelinus* spp. *gnrh1* exon 2 in comparison to the other salmonids we examined. However, unlike for the coho, the GT-AG borders of exon 2 remain similar to those found for the other salmonids. The stop codon (**TGA**) follows the acceptor **TAG** site of exon 3. We join the three exons below to provide the likely complete mRNA and the protein it encodes.

mRNA:

ACCGAAAGTAATAAGGCTGACTTTGCAGAATGGAAGAGAAAAAGGTCTTGTTGCTGCTGCTTTTGGTAGC
GGCTCTAGTGTCACAGGGTTGCTGT**CAACATTGGTCCTATGTCTTGAACCCAGGG**GGGAAAAGAGTGACT
GACAGCCTGTCTGACACCCTGGACAATATGGCTGAAGACCTTCCGAAGATAGACACATCTTGCAGTTTGT
TTGGCTGTGCTGATGTCTCACCTCATCCTCTTACTTGCAAACCTCGC**TGA**CAGACAAAGTGGACTCAATA
ATATATAGCAAATGTATGCTAGCTAACTCT**ATTAAA**CATTGCGGTGCCATCAT

protein:

MEEKKVLLLLLLVAALVSQGCC**QHWSYVLNPG**GKRVTDSLSDTLDNMAEDLPKIDTSCSLFGCADVSPHP
LTCKPR

Online Resource 6

The late-evolving salmon and trout join the GnRH1 club

Histochemistry and Cell Biology

Kristian R. von Schalburg, Brent E. Gowen, Kris A. Christensen, Eric H. Ignatz, Jennifer R. Hall, Matthew L. Rise

Corresponding author at: Department of Biology, Electron Microscopy Laboratory, University of Victoria, Victoria, British Columbia, Canada V8W 3N5

E-mail address: krvs@uvic.ca (K.R. von Schalburg)
