# Supplementary material for: The late-evolving salmon and trout join the GnRH1 club
Source: Histochem Cell Biol. 2023 Aug 11;160(6):517–39. doi: 10.1007/s00418-023-02227-z (PMC10700215; doi:10.1007/s00418-023-02227-z)
Supplement: Supplementary file 7 — Supplementary file7 (PDF 947 KB) [file 418_2023_2227_MOESM7_ESM.pdf]

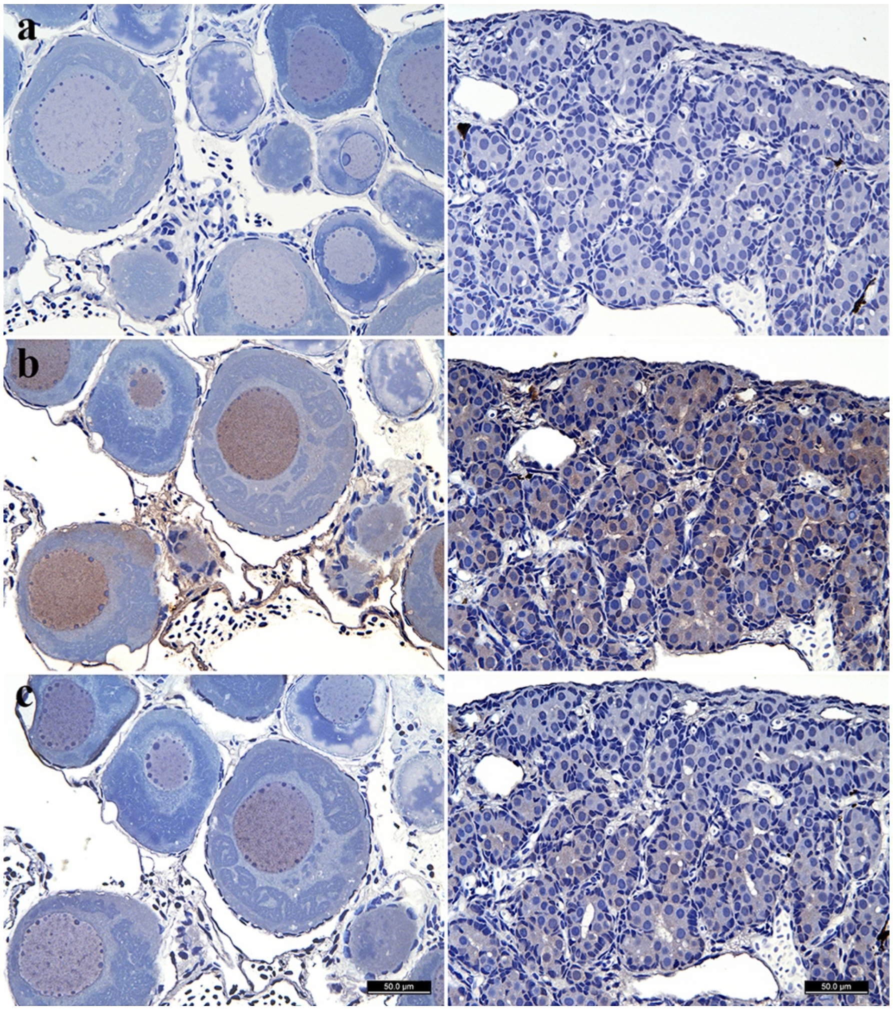

Plate: GF-6 Primary and GnRH Peptide for competitive assay in one-year-old gonads from Atlantic salmon:

a No primary
b Primary alone
c Primary pre-incubated with GnRH1 peptide

Note: in this plate, for each condition (**a to c**), the left panel = ovary and right panel = testis. The scale for all gonad types is the same, indicated on both the last ovary and testis panels within **c.**

Results:
a = no labeling present
b = ovary and testis label well
c = greatly reduced labeling

To test the specificity of the GF-6 primary to GnRH1, 4µm MBM sections of Atlantic salmon one-year-old ovary and testis were immunolabeled using the DAB method. Both the GF-6 primary and the primary pre-incubated with a Biomatik chemically synthesized peptide to GnRH1 were included in the immunolabeling test.

GF-6 and GF-6 incubated with peptide, both in PBS-BSA, were prepared the day before the immunolabeling (all final concentrations at 1:1000 for the primary). The aliquots were left in the fridge overnight and warmed up to room temperature for one hour before use.

(Biomatik Corporation, 4 Third Ave, Kitchener, Ontario, N2C1N6, Canada http://www.biomatik.com).

Biomatic Peptide

Peptide Name	Sequence (N to C)
GnRH1	QHWSYGMNPG

Online Resource 7

The late-evolving salmon and trout join the GnRH1 club

Histochemistry and Cell Biology

Kristian R. von Schalburg, Brent E. Gowen, Kris A. Christensen, Eric H. Ignatz, Jennifer R. Hall, Matthew L. Rise

Corresponding author at: Department of Biology, Electron Microscopy Laboratory, University of Victoria, Victoria, British Columbia, Canada V8W 3N5

E-mail address: krvs@uvic.ca (K.R. von Schalburg)
