# Supplementary material for: The late-evolving salmon and trout join the GnRH1 club
Source: Histochem Cell Biol. 2023 Aug 11;160(6):517–39. doi: 10.1007/s00418-023-02227-z (PMC10700215; doi:10.1007/s00418-023-02227-z)
Supplement: Supplementary file 8 — Supplementary file8 (PDF 72 KB) [file 418_2023_2227_MOESM8_ESM.pdf]

Online Resource 8 Gonadotropin-releasing hormone 1 (GnRH1) orthologs

	Distinct GnRH forms<br>Original name	Abbreviation	GnRH1 Orthologs (amidated)	# Species to date encoding GnRH1 with proven sequence
1	**Mammalian GnRH**	**mGnRH1**	**pQHWSYGLRPG**	Mammals: 181 species<br>Marsupial (koala): 1<br>Amphibians: 6<br>Teleosts: 6<br>Basal teleosts (eel): 3<br>Holostean (gar): 1<br>Sturgeon: 3<br>Cartilaginous (skate): 1
2	His[5] GnRH	**whGnRH1**	**pQHWSHGLRPG**	Whale, Rhino: 2
3	Guinea pig GnRH	**gpGnRH1**	**pQYWSYGVRPG**	Guinea pig: 1
4	Chicken GnRH1	**cGnRH1**	**pQHWSYGLQPG**	Birds: 79 Reptiles: 16
5	Cuckoo GnRH	**cuGnRH1**	**pQHWSFGLQPG**	Birds: 12
6	Egret GnRH	**egGnRH1**	**pQHWSYGLKPG**	Bird (Egret): 1
7	Snake GnRH	**snGnRH1**	**pQHWSYGFQPG**	Snakes: 6
8	Phe[7] GnRH	**flGnRH1**	**pQHWSYGFRPG**	Fence lizard: 1
9	Trp[8] GnRH	**fGnRH1**	**pQHWSYGLWPG**	Amphibians (frogs): 2
10	Seabream GnRH	**sbGnRH**	**pQHWSYGLSPG**	Teleosts: 48
11	Grouper GnRH	**grGnRH1**	**pQLWSYGLSPG**	Teleost (grouper): 1
12	Pejerry/Medaka GnRH	**pjGnRH1**	**pQHWSFGLSPG**	Teleosts: 11
13	Killifish GnRH	**kfGnRH1**	**pQHWSIGMNPG**	Teleost (killifish): 1
14	**Sockeye salmon GnRH1**	**ssGnRH1**	**pQHWFYGLNPG**	Teleost: **Sockeye salmon**
15	**Rainbow trout GnRH1**	**rtGnRH1**	**pQHWSYGLNPG**	Teleost: **Rainbow trout**<br>**Coho salmon**<br>**Chinook salmon**<br>Non-salmonids: 3
16	**Salvelinus spp. GnRH1**	**saGnRH1**	**pQHWSYVLNPG**	Teleost: 1

17	Whitefish GnRH	**wfGnRH1**	**pQHWSYGMNPG**	Teleost: Whitefish<br>**Grayling**<br>**River trout**<br>**Atlantic salmon**
18	Catfish GnRH	**cfGnRH1**	**pQHWSHGLNPG**	Teleost: catfish: 5
19	Herring GnRH	**hrGnRH1**	**pQHWSHGLSPG**	Teleost: (herring/anchovy)
20	Coelacanth GnRH	**coGnRH1**	**pQYWSYDLRPG**	Coelacanth: 1
21	Lungfish GnRH	**luGnRH1**	**pQHWSHGWMPS**	Lungfish: 1
22	Thorny skate GnRH	**skGnRH1**	**pQHWSHGWLPG**	thornyskate: 1
23	Elephant shark GnRH	**esGnRH1**	**pQHWSIDNRPG**	Cartilaginous (elephant shark): 1
24	Catshark GnRH	**csGnRH1**	**pQHWSFDLRPG**	Cartilaginous (catshark)

**Chicken GnRH2**	**GnRH2**	**pQHWSHGWYPG**
**Salmon GnRH**	**GnRH3**	**pQHWSYGWLPG**

Thirteen of the distinct GnRH1 forms presented here have been published (see text). The three novel salmonid GnRH1 types are shown in rows 14, 15 and 16. Additional unpublished data was obtained primarily from translation of genomic sequences found on NCBI. These peptides are presented in rows 2, 5, 6, 7, 8, 13, 21 and 22. They are Whale GnRH1 (H5), Cuckoo GnRH1 (F5/Q8), Egret GnRH1 (K8), Snake GnRH1 (F7/Q8), Fence lizard GnRH1 (F7), Killifish GnRH1 (I5/M7/N8), Lungfish GnRH1 (H5/W7/M8/S10) and Thorny skate GnRH1 (H5/W7), respectively. GnRH2 and GnRH3 peptides are added for comparison.

Online Resource 8 The late-evolving salmon and trout join the GnRH1 club

Histochemistry and Cell Biology

KR von Schalburg, BE Gowen, KA Christensen, EH Ignatz, JR Hall, ML Rise

Corresponding author at: Dept. of Biology, Electron Microscopy Lab, University of Victoria, Victoria, British Columbia, Canada V8W 3N5

E-mail address: krvs@uvic.ca (KR von Schalburg)
